# Reduced Retinal Microvascular Density, Improved Forepaw Reach, Comparative Microarray and Gene Set Enrichment Analysis with c-jun Targeting DNA Enzyme

**DOI:** 10.1371/journal.pone.0039160

**Published:** 2012-07-17

**Authors:** Cecilia W. S. Chan, Warren Kaplan, Christopher R. Parish, Levon M. Khachigian

**Affiliations:** 1 Centre for Vascular Research, University of New South Wales, Sydney, Australia; 2 Peter Wills Bioinformatics Centre, Garvan Institute of Medical Research, Sydney, Australia; 3 Centre for Vascular Research, John Curtin School of Medical Research, Australian National University, Canberra, Australia; Osaka University Graduate School of Medicine, Japan

## Abstract

Retinal neovascularization is a critical component in the pathogenesis of common ocular disorders that cause blindness, and treatment options are limited. We evaluated the therapeutic effect of a DNA enzyme targeting c-jun mRNA in mice with pre-existing retinal neovascularization. A single injection of Dz13 in a lipid formulation containing N-[1-(2,3-dioleoyloxy)propyl]-N,N,N-trimethylammonium methyl-sulfate and 1,2-dioleoyl-sn-glycero-3-phosphoethanolamine inhibited c-Jun expression and reduced retinal microvascular density. The DNAzyme inhibited retinal microvascular density as effectively as VEGF-A antibodies. Comparative microarray and gene expression analysis determined that Dz13 suppressed not only c-jun but a range of growth factors and matrix-degrading enzymes. Dz13 in this formulation inhibited microvascular endothelial cell proliferation, migration and tubule formation *in vitro*. Moreover, animals treated with Dz13 sensed the top of the cage in a modified forepaw reach model, unlike mice given a DNAzyme with scrambled RNA-binding arms that did not affect c-Jun expression. These findings demonstrate reduction of microvascular density and improvement in forepaw reach in mice administered catalytic DNA.

## Introduction

Neovascularization is the process by which new blood vessels are formed by the sprouting of endothelial cells from pre-existing vessels. New vessel formation is a key component of many pathologic conditions, and occurs in the retina in response to prolonged hypoxia, contributing to visual loss in retinopathy of prematurity (ROP), age-related macular degeneration (AMD) and diabetic retinopathy (DR). Together, these afflict people in all stages of life from birth through late adulthood and account for most instances of legal blindness.

ROP was reported at least 60 years ago in premature infants [1], [2] and there are 15,000 newborns in the United States that are affected each year [3], [4]. This represents the leading cause of blindness in children. Despite new therapeutic approaches that have improved the prognosis of newborns with ROP such as pegaptanib (Macugen), bevacizumab (Avastin) and ranibizumab (Lucentis), unmet need remains such as the need for multiple injections and affordability [5], [6]. The pathogenesis of ROP, or oxygen-induced retinopathy (OIR) is organized in two distinct phases. The first, ischemic phase, involves hyperoxia-induced arrest in vascular development and vaso-obliteration. The second, vaso-proliferative phase is thought to occur when the avascular retina becomes ischemic and triggers the release of pro-angiogenic factors [7]. This leads to aberrant retinal neovascularization characterizing human ischemic retinopathies.

OIR is regulated by key factors such as vascular endothelial growth factor (VEGF). Retinal neovascularization is suppressed by agents that bind VEGF or block VEGF receptors [8]. A range of other mediators have also been implicated in the pathogenesis of ocular neovascular disease, including fibroblast growth factor-2 (FGF-2), matrix metalloproteinase-2 (MMP-2), MMP-9, insulin-like growth factor (IGF)-1 and angiopoietin [9]–[12]. The 42 kDa basic region leucine zipper transcription factor c-Jun regulates the expression of VEGF and a range of pro-angiogenic genes and controls cellular processes such as proliferation, transformation and apoptosis [13].

“10–23” DNAzymes such as Dz13 that targets c-Jun are single-stranded synthetic all-DNA catalysts that comprise a cation-dependent catalytic core of 15 deoxyribonucleotides [14]–[17]. These bind a complementary sequence through Watson-Crick base pairing and cleave target mRNA via a de-esterification reaction. DNAzymes are relatively small, inexpensive to synthesize and resist nuclease degradation. Here for the first time we explored the ability of Dz13 to reduce retinal microvascular density in an OIR model in mice with pre-existing retinal angiogenesis and compared Dz13 efficacy against that of VEGF-A antibodies. We also interrogated the effect of the DNAzyme on visual behavior and its impact on changes in gene expression in the eye.

## Materials and Methods

### Cell Culture

Human microvascular endothelial cells (HMEC) (American Type Culture Collection, Manassas, VA) were maintained in MCDB131 media (Gibco) supplemented with 10% fetal bovine serum (FBS), hydrocortisone (500 µg/ml, Sigma), epidermal growth factor (0.01 µg/ml, GIBCO), L-glutamine (2 mM) and antibiotics [18]. The cells were seeded at 1×10^5^ cells/ml and sub-cultured after detachment with 0.05% trypsin/5 mM EDTA.

### Cell Transfection and Proliferation Assays

Typically, 3500 HMEC were seeded into 96-well plates. After approximately 24 h with 70% confluence, MCDB 131 media with 10% FBS was replaced with serum free MCDB 131 media for further 20 h for serum arrest. Cells were then transfected with DOTAP/DOPE [19] with Dz13, 5′-CGG GAG GAA GGC TAG CTA CAA CGA GAG GCG TTG (3′-3′ T)-3′ and Dz13scr, 5′-GCG ACG TGA GGC TAG CTA CAA CGA GTG GAG GAG (3′-3′ T) -3′ at a ratio of 3:1 (3 µl of 1 mg/ml DOTAP/DOPE to 1 µg DNA) at concentration of 0.2–0.4 µM. After 4 h of transfection, the media was replaced with 10% FBS MCDB 131 for further 48 h. Media was removed and cells were washed with PBS. Cells were trypsinized and the suspension per well was quantified in an automated Coulter counter (Z series, Coulter).

### Lactate Dehydrogenase (LDH) Assay

LDH activity in the supernatants of transfected HMEC was determined using the Cytotoxicity Detection Kit according to the manufacturer’s instructions (Roche).

### Endothelial Tubule Formation Assays

HMEC (30000 cells) were grown and transfected as described above in 6-well plates. The cells were trypsinized and 3000 cells seeded onto 100 µl Matrigel (BD Bioscience) in 96-well plates in medium with or without 5% FBS. Photographs of the tubules were taken at hourly intervals using a Nikon eclipse TE2000-S fluorescence microscope. Tubules >3 µm in 96 well plates were quantified in each of 4 random fields using NIH Image J software.

### *In vitro* Wound Repair

HMEC were grown and transfected as described in 6-well plates and serum free or 5% FBS conditioned MCDB 131 then placed on the cells and the cells cultured until a monolayer of cells was formed. The cells were injured by scraping with a sterile toothpick and were left for 48 h. The denuded zone was standard in every case and created in each well by the uniform linear stroke of a sterile toothpick. Uniformity of width in the denuded zone (300 µm) was confirmed under phase contrast microscopy immediately after scraping injury. Calcein (500 µM) was incubated with the cells for 20 min prior to fluorescence microscopy.

### Western Blotting

Protein concentrations in the extracts were determined using the micro-BCA assay (Pierce, Rockford, IL). Equal amounts of total protein were loaded onto 10–12% polyacrylamide gels. Western blotting was performed essentially as described [20], [21] using commercial rabbit or goat antibodies (Santa Cruz Biotechnology, Abcam, R&D Systems): rabbit monoclonal c-Jun antibodies were used at 1∶500 dilution, rabbit polyclonal VEGF-A antibodies were used at 1∶500, rabbit polyclonal FGF-2 antibodies were used at 1∶1000, and rabbit polyclonal MMP-2 antibodies were used at 1∶500. Secondary polyclonal swine anti-rabbit antibodies were obtained from Dako and used at 1∶1000. Unbiased loading was confirmed by probing for beta-actin (Sigma).

### Murine Model of Oxygen-induced Retinopathy

C57BL/6 mice and postnatal day 2 (P2) pups were purchased from Australian Resources Centre (Perth). Postnatal P6.5–P7 mice were exposed to hyperoxia (75% oxygen) for 4 d in Quantum-Air Maxi-Sealed cages (Hereford, UK) [20]. Following this period of hyperoxia, the mice were returned to normoxia for 5 d and intravitreal injections were performed (I-0). This is approximately a week after complete coverage of the retina by the superficial plexus [22], and after the hyperoxia/normoxia cycle that increases pathologic retinal angiogenesis and vascular density. Intravitreal injections of 20 µg Dz13 or Dz13scr (in 1.68 µl H_2_O containing 0.2 µl of 1 mg/ml N-[1-(2,3-dioleoyloxy)propyl]-N,N,N-trimethylammonium methyl-sulfate and 1,2-dioleoyl-sn-glycero-3-phosphoethanolamine, i.e., DOTAP and DOPE), its vehicle alone (H_2_O, DOTAP/DOPE) or 10 µg/µl VEGF-A antibodies (Roche CAS 216974-75-3) (in PBS, pH 7.4) [23] or its control (10 µg/µl IgG in PBS, pH 7.4) were performed with 26-gauge beveled needles attached to a micro-volume syringe (SGE Analytical Science syringe Melbourne, Australia). The mice were left at room oxygen for a further 10 d (I-10) and pup eyes were then enucleated and fixed in 10% formalin in PBS for immunohistochemistry or TRIzol (Invitrogen) for RNA extraction. Mice were deeply anesthetized with ketamine and xylazine for all procedures. Experiments were performed with and without operator blinding.

### DNAzyme Localization Studies

Twenty µg of FITC-DNAzyme (TriLink BioTechnologies) was injected intravitreally in anaesthetized pups in the OIR model as described above. Eyes were removed from the pups 2 d after the injections and were visualized under a fluorescence microscope (Olympus DP70).

### Histology and Immunohistochemical Analysis

Experimental and control mice were sacrificed at I-0 or I-10, eyes enucleated, fixed in 10% formalin overnight, and embedded in paraffin. Serial 5 µm cross sections of whole eyes were cut sagitally, at a fixed distance from the optic nerve (0, 50, 100 µm), and stained with H&E. Blood vessels from each group were quantified under light microscopy (3 sections per eye) as erythrocyte-filled vessels and vessels located on the vitreal side of the inner limiting membrane [24] were taken into consideration. With n = 6–8 eyes per group and 3 sections assessed per eye, each group represents 18–24 separate measurements. Immunohistochemical staining was performed on formalin-fixed paraffin-embedded tissues with rabbit polyclonal anti-mouse c-Jun antibodies at 1∶200 dilution or rat polyclonal anti-mouse CD34 antibodies at 1∶500 dilution (Santa Cruz and Abcam), and biotinylated goat anti-rabbit or rabbit anti-rat secondary antibodies at 1∶200 (Dako). Each specimen was processed together with its negative control that was IgG primary antibody (Santa Cruz).

### Visual Placement Test

Mice had their whiskers trimmed to a length of 1 cm from the skin at I-10 for the visual placement test. The animals were lifted by the base of their tails to 30 cm above the cage, then lowered vertically toward the cage top at a steady speed of 1 cm/s [25]. The ability of the mice to reach the cage top with their forepaws was determined 5 times for each mouse. Data is expressed as the number of forepaw-reaching episodes that did, or did not involve the whiskers touching the cage surface. Two independent visual placement experiments were performed, one under blinded conditions. There was no significant difference between these experiments.

### RNA Isolation

Eyes were enucleated and placed in TRIzol on dry ice according to the manufacturer’s instructions (Purelink RNA Minikit, Invitrogen).

### Quantitative Real-Time PCR

qPCR was carried out using Rotor-Gene 3000 (Corbett Life Science). The reaction was set in a final volume of 20 µl containing 0.5 µl of cDNA, 10 µl of SYBR Green Master Mix (Applied Biosystems), 0.6 µl of 10 µM of forward and reverse primer (Sigma), and 8.9 µl of DNase-free water. Murine c-jun primers were: forward, 5′-ACT CCG AGC TGG CAT CCA-3′, reverse, 5′- CCC ACT GTT AAC GTG GTT CAT G-3′. PCR conditions were: 95°C for 10 min followed by 35 cycles at 65°C for 20 sec, 72°C for 20 sec. Murine MMP-2 primers were: forward 5′-GGG ACA AGA ACC AGA TCA CAT AC-3′, reverse, 5′-CTT CTC AAA GTT GTA GGT GGT GG-3′. PCR conditions were: 95°C for 10 min followed by 28 cycles at 57°C for 30 sec and 72°C for 40 sec. Murine MMP-9 primers were: forward 5′-CCA AGG GTA CAG CCT GTT CCT-3′, reverse, 5′-GCA CGC TGG AAT GA C TAA GC-3′. PCR conditions were 95°C for 10 min followed by 30 cycles at 52°C for 20 sec and 72°C for 20 sec. Murine MMP-12 primers were: forward 5′–TTT GAC CCA CTT CGG CC-3′, reverse 5′–GTG ACA CGA CGG AAC AG-3′. PCR conditions were 95°C for 10 min followed by 30 cycles at 59°C for 20 sec and 72°C for 20 sec. Murine MMP-13 primers were: forward 5′–GTA ATC GCA TTG TGA GAG T-3′, reverse 5′–ATC AGG TGA TCC TTG GG-3′. PCR conditions were 95°C for 10 min followed by 30 cycles at 56°C for 20 sec and 72°C for 20 sec. Murine VEGF-A primers were: forward 5′–ATG AAC TTT TCT GCT GTC TTG GGT G-3′, reverse 5′–TCA CCG CCT CGG CTT GTC ACA T-3′. PCR conditions were 95°C for 10 min followed by 30 cycles at 68.5°C for 20 sec and 72°C for 20 sec. Murine VEGF-C primers were: forward 5′–AAC GTG TCC AAG AAA TCA GCC-3′, reverse 5′–AGT CCT CTC CCG CAG TAA TCC-3′. PCR conditions were 95°C for 10 min followed by 30 cycles at 65°C for 20 sec and 72°C for 20 sec. Murine FGF-2 primers were: forward 5′–GTC ACG GAA ATA CTC CAG TTG G-3′, reverse 5′–CCC GTT TTG GAT CCG AGT T-3′. PCR conditions were 95°C for 10 min followed by 30 cycles at 60°C for 20 sec and 72°C for 20 sec. Murine beta-actin primers were, forward, 5′-AGC CAT GTA CGT AGC CAT CC-3′, and reverse, 5′-CTC TCA GCT GTG GTG GTG AA-3′.

### Microarray Analysis and GSEA

Total RNA (10 µl at 50 ng/µl concentration) from 8–10 pooled whole eyes was used to prepare labeled probes for microarray analysis with a one-cycle protocol (Affymetrix Gene 1.0 ST arrays, University of New South Wales Ramaciotti Centre for Gene Function Analysis, Australia). Two independent experiments were performed, one under blinded conditions. Normalization and probe set summarization was performed using the robust multichip average [26] implemented in the Affy library [27] from R/Bioconductor [28], [29]. Control probe sets were removed leaving 28815 probe sets on the array. Differential gene expression was assessed for each probe set using Limma [30]. Gene Set Enrichment Analysis was run with the GenePattern tool GSEA preranked using a ranked list of fold-changes derived from Limma analysis. Of the 3272 gene sets in the curated gene set collection 786 gene sets were filtered out by the gene set size filter (min = 15, max = 500) leaving 475 gene sets upregulated in Dz13 and 2011 downregulated in Dz13. All analyses were performed using Gene Pattern software [31] and are available at http://pwbc.garvan.unsw.edu.au/gp. Microarray data are accessible from GEO: GSE37898.

### Animal Ethics and Statistical Analysis

Animal experiments were approved by the Animal Care and Ethics Committee, University of New South Wales. All values are expressed as the mean ± s.e.m. The data was analysed by one-way ANOVA followed by Bonferroni-Dunn post hoc analysis (GraphPad Prism 5) and/or Student’s t-test comparing individual groups.

## Results

### Dz13 in DOTAP/DOPE Inhibits Retinal Microvascular Density in a Modified OIR Model

**Figure 1:**
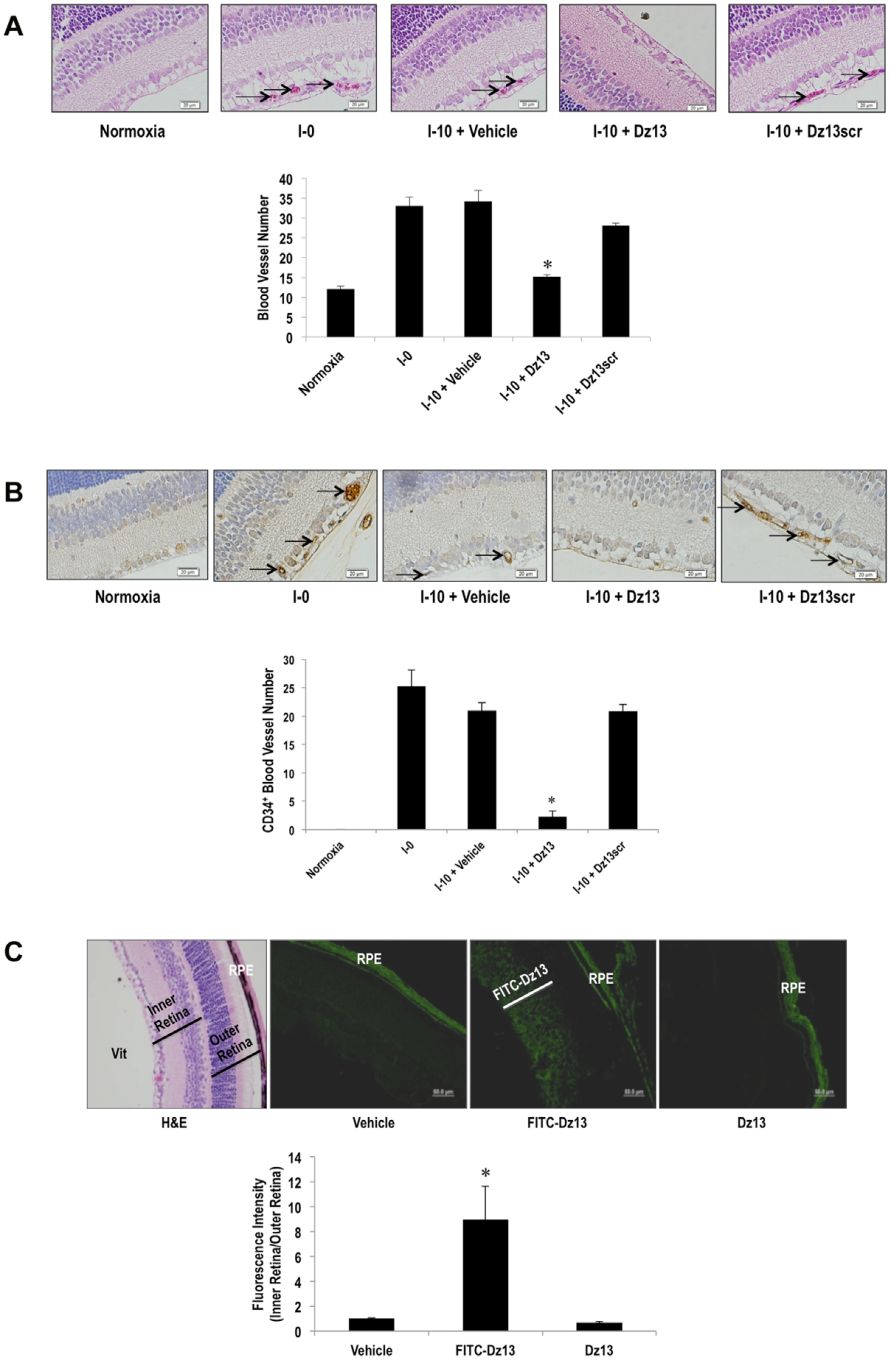
Dz13 reduces pre-existing retinal microvascular density and penetrates the subretinal space. C57BL/6 mice were exposed to 75% oxygen for 4 d and returned to room air for 5 d. Dz13 or Dz13scr (20 µg in DOTAP/DOPE) was injected intravitreally (I-0). After 10 d (I-10), the animals were sacrificed, the eyes enucleated, cross-sectioned and stained with H&E (**A**) or, alternatively, with antibodies to CD34 (**B**)**.** Blood vessels on the vitreal side of the inner limiting membrane were quantified by counting erythrocyte-filled vessels in H&E-stained sections or CD34-stained vessels per section. “Blood vessel number” represents the mean number of blood vessels in 3 parallel cross-sections (5 µm each) per retina at 3 distances from the optic nerve (0, 50, 100 µm). Blood vessels are denoted by arrows. n = 6–8 eyes per group. Data is representative of 2 or more experiments. FITC-Dz13 (20 µg) in DOTAP/DOPE was injected intravitreally on I-0 and fluorescence intensity at I-2 was determined using NIH Image J software (**C**). *denotes P<0.05 relative to vehicle or Dz13scr.

We evaluated the therapeutic effect of Dz13 in mice with pre-existing retinal angiogenesis. The OIR model has mainly been used to evaluate the effect of candidate molecules to inhibit the onset of retinal angiogenesis. Here we employed this system in a therapeutic “curative” rather than preventative setting. Dz13 (20 µg in 1.68 µl H_2_O containing DOTAP/DOPE) was administered once, intravitreally to I-0 C57BL/6 mice. These mice had previously been exposed to 75% oxygen for 4 d and returned to room air to render the inner retina relatively hypoxic [24]. Alternatively the mice were injected with Dz13scr (20 µg) or the vehicle alone. Ten days following DNAzyme administration, at I-10, the mice were sacrificed, the eyes enucleated, cross-sectioned and stained. Retinal microvascularity in H&E- (**Fig. 1A**) and CD34- (**Fig. 1B**) stained sections from control mice increased under these conditions at I-0, and levels remained elevated by I-10. CD34 has previously been used to quantify retinal microvascular density after OIR in C57BL/6J mice [32] and as an endothelial marker in human choroidal neovascularization [33]. At I-10, Dz13 dramatically reduced the number of retinal blood vessels (**Figs. 1A & B**). In contrast, the same load of its scrambled arm counterpart, Dz13scr, did not affect retinal vascular density compared with the vehicle group (**Figs. 1A & B**). Fluorescence microscopy was performed to determine whether the DNAzyme was absorbed into the subretinal space. FITC-Dz13 (20 µg) in DOTAP/DOPE was injected intravitreally on I-0 and fluorescence intensity was evaluated in cross-sections of the retina at I-2. FITC-Dz13 localized subretinally (**Fig. 1C**), whereas eyes injected with Dz13 without the label or DOTAP/DOPE showed no evidence of retinal fluorescence except for autofluorescence in the retinal pigment epithelium, as previously observed [34] (**Fig. 1C**).

### Dz13 Inhibits Expression of c-jun and c-Jun-dependent Genes in Eyes of OIR Mice

**Figure 2:**
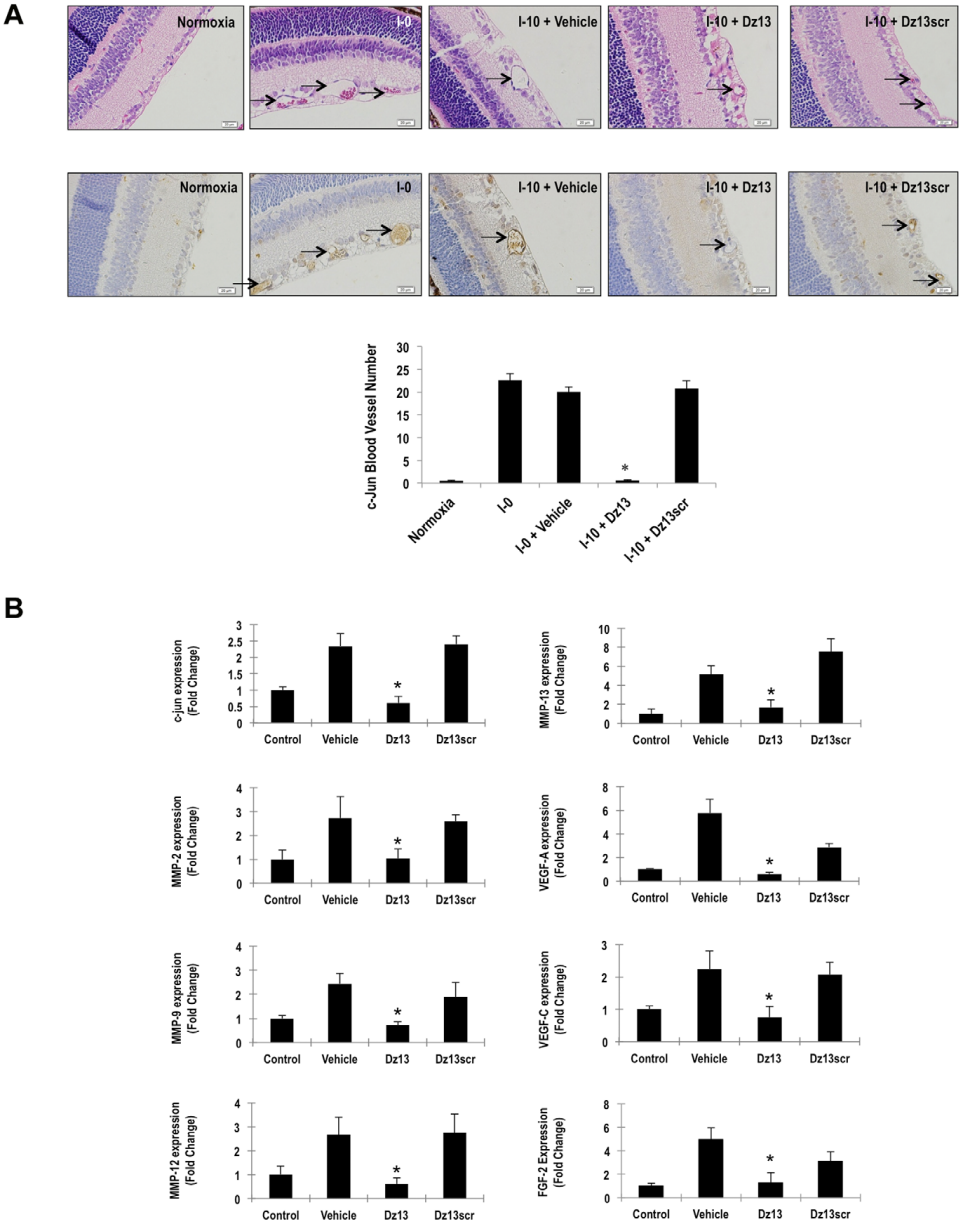
Dz13 inhibits c-Jun, FGF-2, VEGF-A, MMP-2 and MMP-9 expression in eyes of OIR mice. (**A**) Immunohistochemical analysis for c-Jun immunoreactivity in the retina of I-10 mice treated on I-0 with Dz13, Dz13scr or vehicle. Where indicated, IgG was used as the primary antibody as a negative control. Blood vessels are denoted by arrows. n = 6–8 eyes per group. (**B**) qPCR analysis on eyes of I-10 mice for c-jun, FGF-2, VEGF-A, VEGF-C, MMP-2, MMP-9, MMP-12 and MMP-13 mRNA. *denotes P<0.05 relative to vehicle and Dz13scr. Data is representative of 2 or more experiments.

Immunohistochemical staining revealed that c-Jun immunoreactivity in microvessels on the vitreal side of the inner limiting membrane in OIR mice was inhibited by Dz13. c-Jun expression at I-10 was observed in retinal microvessels in the Dz13scr and vehicle groups, but was barely detectable in the Dz13 group (**Fig. 2A**)**.** Since c-Jun, as a transcription factor, controls the expression of multiple other genes, we examined the effect of Dz13 on genes induced under conditions of OIR by comparative microarray analysis. This approach was used to compare the expression profile between Dz13, Dz13scr and vehicle (DOTAP/DOPE)-treated eyes at I-10 (**Fig. S1, right**). When comparing the Dz13 and Dz13scr groups we found 251 probe sets had a log2 fold-change >1.5 (**Fig. S1**, **upper left**). c-jun was one of the 187 probe sets downregulated by Dz13 whereas 64 probe sets were upregulated. Among other genes induced by OIR that were inhibited by Dz13 were a range of pro-angiogenic growth factors and matrix-degrading enzymes (**Fig. S1, right**). Quantitative real-time PCR (qPCR) analysis of eyes of I-10 mice revealed that Dz13 inhibited the OIR-inducible expression of not only c-jun mRNA relative to Dz13scr, but also that of FGF-2, VEGF-A, VEGF-C, MMP-2, MMP-9, MMP-12 and MMP-13 (**Fig. 2B**).

### Gene Set Enrichment Analysis of Eyes of OIR Mice Treated with DNAzyme

In order to understand the genome-wide effect of Dz13 vs Dz13scr the log2 fold-changes of all 28815 genes on the array were inputed into gene set enrichment analysis (GSEA) [35] and compared to the curated gene sets (c2all) from version 3 of the Molecular Signatures Database (MSigDB) [35]. The curated gene sets are sourced from pathway databases as well as results from published microarray studies. Unlike individual gene analysis GSEA shifts the emphasis from the role played by single genes to groups of genes (gene sets). It also offers the advantage that no thresholding of genes is performed and all genes on the array are used. At an FDR <25%, 765 gene sets were significantly downregulated by Dz13 while only 3 gene sets were significantly upregulated (**Table S1**). An overwhelming number of these downregulated gene sets, including those containing MMP-12 and MMP-13 (**Fig. S1, lower left**) are associated with cancer (**Table S2**)**.**

### Dz13 Inhibits Endothelial Cell Proliferation, Migration and Tubule Formation *in vitro*

**Figure 3:**
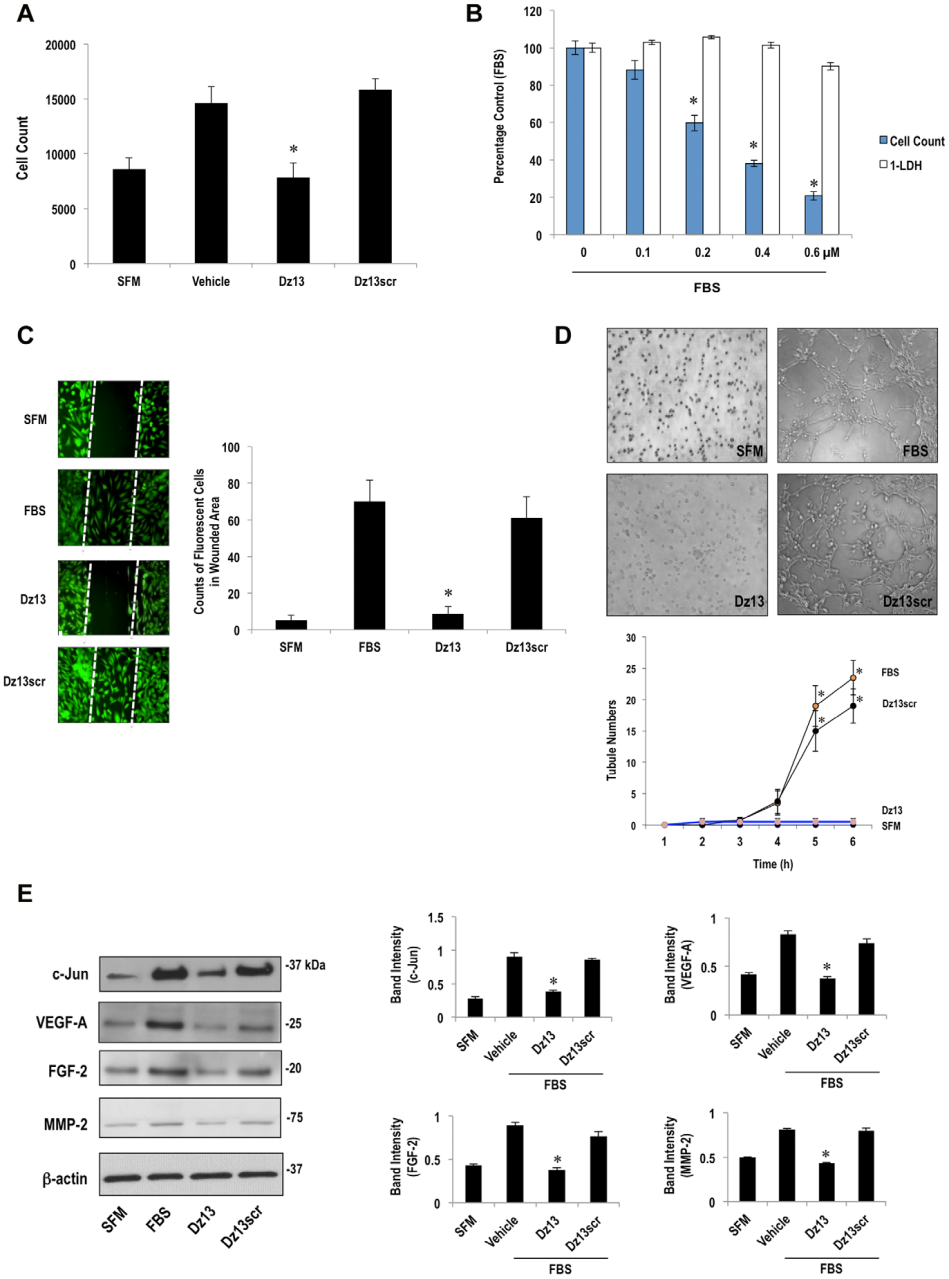
Inhibition of endothelial cell proliferation, migration and tubule formation by Dz13 *in vitro*, and suppression of c-Jun and c-Jun-dependent gene expression. (**A**) Effect of Dz13 (0.4 µM) on serum-inducible HMEC proliferation after 2 d. Endothelial cell proliferation was quantified as the total number of cells per well (triplicate wells per group) in 96 well plates. (**B**) Toxicity and cell count in HMEC after 2 d. LDH activity and cell counts were quantified in triplicate cells of a 96 well plate per group. The % Control (FBS) for the “1-LDH” plot was calculated by subtracting LDH values (% LDH positive) in the Dz13-treated groups from 100% and expressing these as a percentage of LDH activity in the FBS control, and for the proliferation plot, expressing cell numbers the Dz13-treated groups as a percentage of numbers in the FBS control. (**C**) Effect of Dz13 (0.4 µM) on HMEC migration into the denuded zone (of 300 µm width) 2 d after *in vitro* scraping injury. Counts represent number of cells that have migrated into the denuded zone in 3 random fields using NIH Image J software. Calcein was added for 20 min before quantification. (**D**) Effect of Dz13 (0.2 µM) on tubule formation by HMEC on Matrigel. Tubules >3 µm in 96 well plates were quantified hourly in each of 4 random fields using NIH Image J software. Representative images taken at the 6 h time point are shown in the figure. (**E**) Western blotting using extracts of growth-quiescent HMEC exposed to 5% FBS for 1 h. SFM denotes serum-free medium. Blots were quantified by scanning densitometry. Data is representative of 2 or more experiments. *denotes P<0.05 relative to control.

Angiogenesis is a complex process involving endothelial cell proliferation and migration. We next confirmed that Dz13 (in DOTAP/DOPE) could inhibit these key cellular processes in human microvascular endothelial cells (HMEC). Serum-inducible HMEC proliferation after 2 d was inhibited by 0.4 µM of Dz13, but not Dz13scr (**Fig. 3A**). The inhibitory effects of Dz13 on endothelial cell proliferation were not due to toxicity. We performed an assay for lactose dehydrogenase (LDH) over a range of concentrations and quantified cell numbers after trypsinization. **Fig. 3B** demonstrates that Dz13 inhibits endothelial proliferation at 0.4 µM without toxicity. Dz13 (0.4 µM) also blocked HMEC regrowth into the denuded zone 2 d following *in vitro* scraping injury, whereas Dz13scr had no effect (**Fig. 3C**). Angiogenesis involves the ordered assembly and alignment of endothelial cells. *In vitro*, endothelial cells spontaneously align and form a three-dimensional tubular capillary-like network within hours of plating on Matrigel, a commercial basement membrane matrix mixture. HMEC formed tubules within 4–6 h in this model (**Fig. 3D**). Dz13 in DOTAP/DOPE completely blocked tubule formation, whereas Dz13scr had no effect (**Fig. 3D**). Dz13 (0.4 µM) in DOTAP/DOPE inhibited the inducible c-Jun expression in HMEC exposed to serum for 1 h (**Fig. 3E**). Dz13 in this formulation also inhibited the expression of VEGF-A, FGF-2 and MMP-2 in these cells, whereas Dz13scr had no inhibitory effect (**Fig. 3E**).

### Comparison of Dz13 to VEGF-A Antibodies

**Figure 4:**
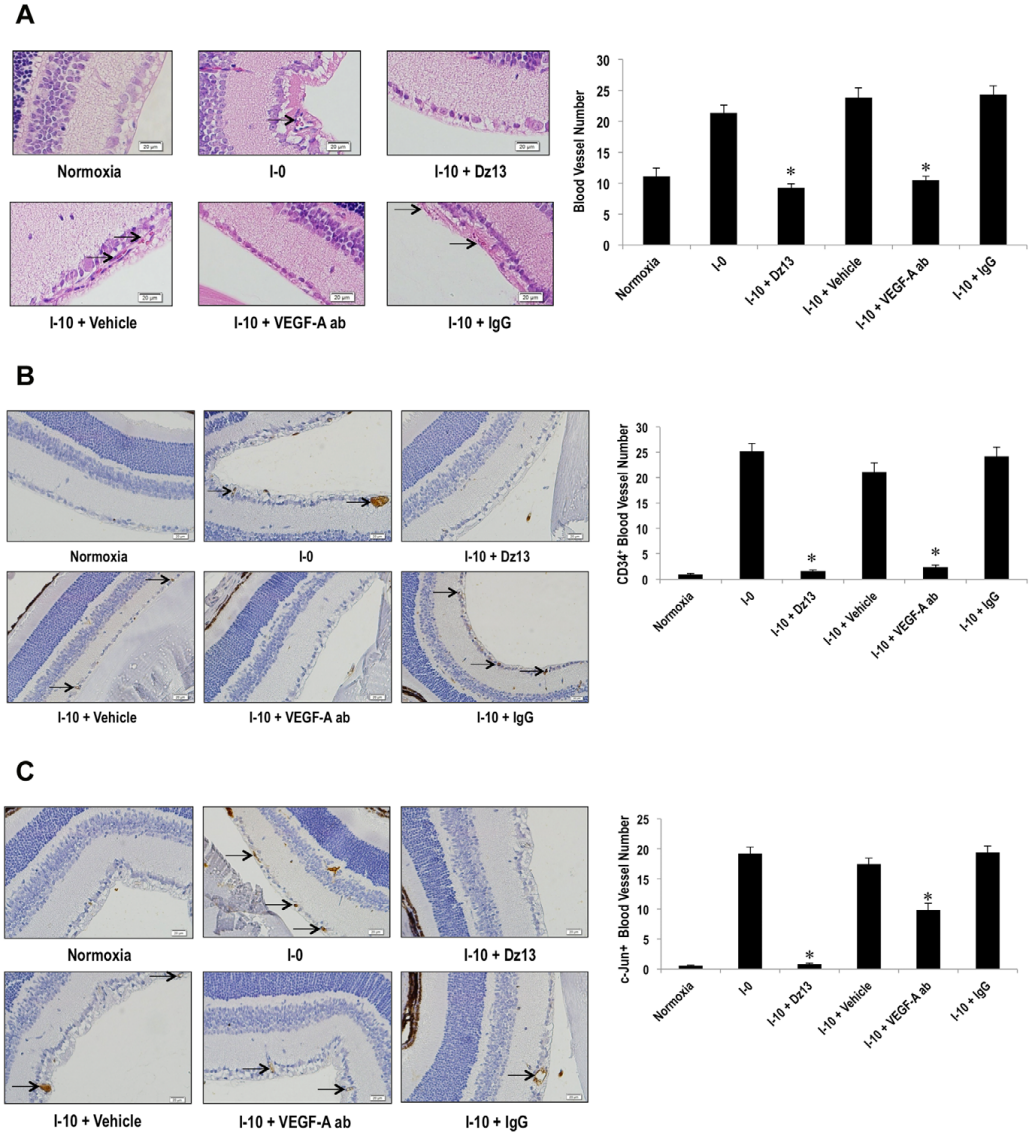
Comparison of Dz13 with VEGF-A antibodies in the modified OIR model. (**A**) C57BL/6 mice (I-0) were injected with Dz13 (20 µg) in DOTAP/DOPE or its vehicle control (H_2_O containing DOTAP/DOPE), VEGF-A antibodies (10 µg/µl) in PBS, pH 7.4 or IgG (10 µg/µl) in PBS, pH 7.4 and eyes removed at I-10. VEGF-A antibody concentration was as per Ref [23]. Blood vessels on the vitreal side of the inner limiting membrane were quantified by counting erythrocyte-filled vessels in H&E-stained sections (**A**), CD34-stained sections (**B**) or c-Jun-stained section (**C**). “Blood vessel number” represents the mean number of blood vessels in 3 parallel cross-sections (5 µm each) per retina at 3 distances from the optic nerve (0, 50, 100 µm). n = 6–8 eyes per group. Blood vessels are denoted by arrows. *denotes P<0.05 relative to vehicle or IgG.

We next compared the effect of Dz13 against a clinically-used approach such as VEGF-A antibodies (akin to Lucentis) in this model. I-0 mice were administered intravitreally with Dz13 (20 µg) in vehicle (H_2_O containing DOTAP/DOPE) or vehicle alone, or VEGF-A antibodies [23] (10 µg/µl in PBS, pH 7.4) or IgG in vehicle. Microvascular density at I-10 in all injected control groups did not differ from that of uninjected I-0 eyes (**Fig. 4A**). However, in eyes treated with Dz13 or VEGF-A antibodies, there was virtually complete inhibition of retinal vascularity relative to background (**Fig. 4A**). Importantly, Dz13 was as effective as VEGF-A antibodies in the inhibition of retinal microvascular density (**Fig. 4A**). This was further confirmed by immunohistochemical analysis indicating reduced CD34^+^ blood vessels in Dz13 and VEGF-A antibody-treated eyes compared with vehicle and IgG controls (**Fig. 4B**). Interestingly, although Dz13 reduced c-Jun immunoreactivity in the retina, c-Jun expression was still detected in VEGF-A antibody-treated eyes (**Fig. 4C**). This suggests that, unlike Dz13, c-Jun may not be the primary means by which VEGF-A targeting strategies effect inhibition of retinal neovascularization in this model.

### Effect of Dz13 Treatment in a Modified Forepaw Reach Model

**Figure 5:**
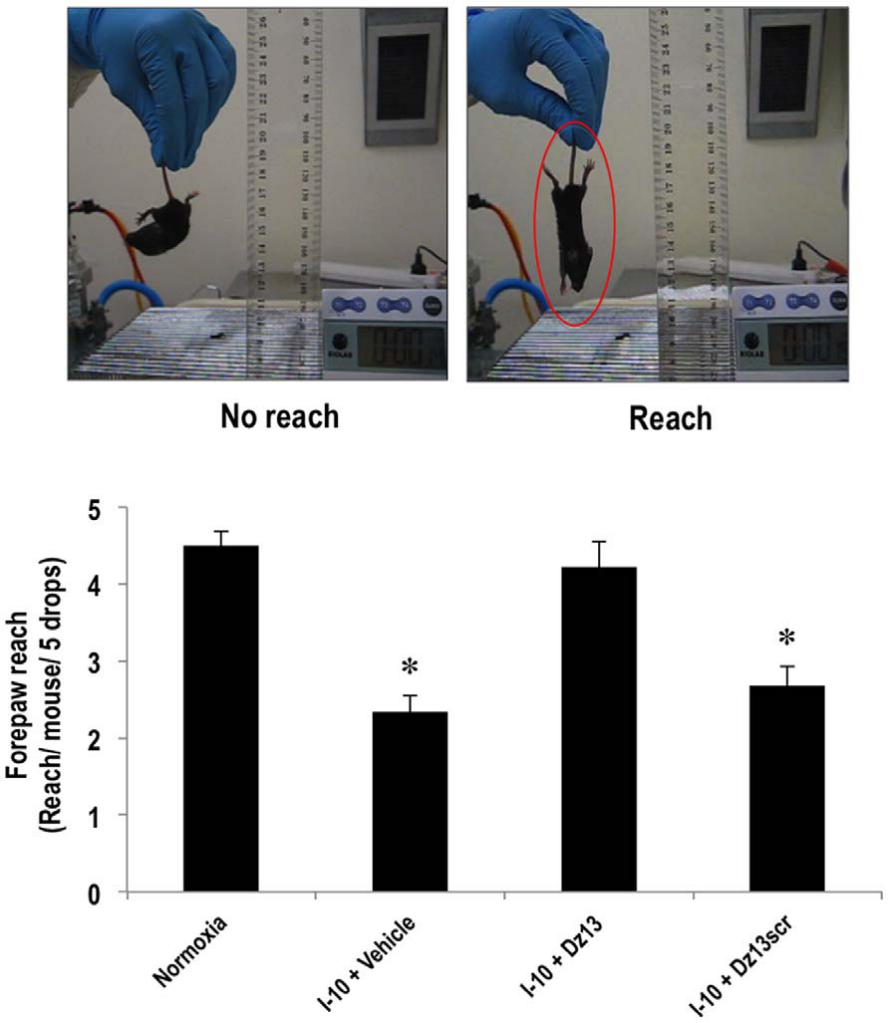
Effect of Dz13 treatment in a modified forepaw reach model. C57BL/6 mice (I-0) were injected with Dz13 or Dz13scr (20 µg) in H_2_O containing DOTAP/DOPE and on I-10, the animals were subjected to the forepaw reach reflex test. Mice forepaw-reaching reflex at the cage top was determined 5 times for each mouse. The observer was blinded to the groups. The circled mouse displays forepaw reach. n = 6–8 eyes per group. Data is representative of 2 or more experiments. * denotes P<0.05 relative to vehicle or Dz13scr.

The preceding data provide a compelling anatomical demonstration of the capacity of Dz13 in DOTAP/DOPE to reduce pre-existing microvascular networks in OIR. Lastly we determined whether Dz13 could provide a behavioral advantage to mice using a modified forepaw reach reflex model. This model has been used by others to indicate the presence of sight and involves animals being lowered from a fixed distance to the top of a cage [36] and evaluating forepaw reach for the bars of the cage when brought closer to the cage. Normal mice reached for the cage in almost every instance, whereas mice subjected to OIR injected with vehicle sensed the top of the cage poorly (**Fig. 5**). I-10 animals treated with Dz13 (20 µg) sensed the top of the cage, whereas mice injected with Dz13scr did not differ from the vehicle group (**Fig. 5**). Our findings above show that retinal microvascular density in the latter two groups was brought to background levels in Dz13 mice (**Figs. 1A & B** **,** **4A**). These findings thus demonstrate that a once-only intravitreal administration of Dz13 in DOTAP/DOPE to mice with pre-existing retinal microvascularity provides measurable anatomic and behavioral benefit.

## Discussion

Retinal neovascularization is hallmark in the pathogenesis of a range of common ocular disorders. The objective of this study was to determine the effect of Dz13 on pre-existing retinal microvascularity, and compare it to a clinically-used approach. We also aimed to demonstrate a behavioral consequence of Dz13 inhibition beyond retinal angiogenesis. In a modified OIR model, Dz13, in a formulation containing DOTAP/DOPE caused a marked reduction in retinal blood vessel density, whereas Dz13scr had no effect. Dz13 inhibited the expression of c-Jun, and a range of c-Jun dependent genes such as FGF-2, VEGF-A, MMP-2 and MMP-9. This was supported by comparative microarray analysis by GSEA, which shifts emphasis from individual genes to groups of genes (gene sets). When comparing Dz13 to Dz13scr many cancer gene sets were downregulated by Dz13. The DNAzyme formulation also inhibited microvascular endothelial cell growth, migration and tubule formation *in vitro*. Using a modified forepaw reach model, mice treated with Dz13 sensed the top of the cage unlike their vehicle or scrambled DNAzyme counterparts.

Dz13 was used in a clinically-relevant curative model in a liposomal formulation. Analysis of cross-sections of Dz13-treated retina by fluorescence microscopy revealed that the DNAzyme localized subretinally. Local intravitreal delivery makes amenable the use of a carrier with the DNAzyme. Interestingly, a carrier does not appear to be essential *in vivo.* Oligonucleotides such as DNAzymes have also been used in a range of animal models with and without a transfection agent. Indeed, clinical trials of antisense oligonucleotides have employed naked oligonucleotide with no carrier. Physiologic delivery *in vivo* may be achieved through endogenous mechanisms [37].

AMD and DR are leading causes of blindness in the Western world. VEGF-A antibodies, delivered intravitreally, represent an FDA-approved therapy in retinal diseases such as AMD and DR [38], [39] and include Lucentis (ranibizumab, Genentech) and Avastin (bevacizumab, Genentech). A recent study from the Comparison of AMD Treatments Trials (CATT) involving 1208 patients with neovascular age-related macular degeneration demonstrated that Avastin and Lucentis are equally effective in treating AMD, with mean change in visual acuity at 1 year the primary outcome [40]. VEGF siRNA (**bevasiranib,** Opko Health) used first in-class and delivered intravitreally, was found to be safe and well-tolerated following repeated administration until clinical trials were terminated in Phase III in 2009 due to the trial being unlikely to meet its primary endpoint. It also appeared that the siRNA approach did not inhibit through predicted mechanisms [6], [14], [41]. In the present study, Dz13 reduced retinal microvascular density as effectively as VEGF-A antibodies. Interestingly, although Dz13 inhibited c-Jun, this was not observed in the retina of mice treated with VEGF-A antibodies. This suggests that antibody strategies targeting VEGF-A may reduce retinal microvascular density by mechanisms independent of c-Jun, even though VEGF-A stimulates and is itself induced by c-Jun. Since it appears that mechanisms mediating the reduction in blood vessel density by Dz13 and anti-VEGF-A antibodies are likely to be different, future clinical strategies to reduce retinal vascular density in ocular pathologies may benefit from the combinatorial use of Dz13, VEGF-A antibodies and/or other agents. We did not observe any evidence of toxicity as a consequence of intravitreal Dz13 delivery or the OIR model. Histological examination of retinas treated with Dz13 revealed no discernable difference to Dz13scr- or vehicle-treated eyes, except for reduced vascular density in the Dz13 group. There was no evidence of retinal degeneration or retinal detachment. It is difficult to determine with certainty whether Dz13 has inhibitory effects on normal or pathologic vessels. It is nonetheless clear in this model of pre-existing retinal vascularity that Dz13 reduces vascular density stimulated by OIR to levels of the normal retina. A recent article from our group demonstrates that the inhibitory activity of Dz13 does not appear to involve CpG motifs and a Toll-like receptor response [19].

Dz13 offers a number of advantages over VEGF-A antibodies. DNAzymes being DNA are relatively small and inexpensive to synthesize and resistant to nuclease degradation. The 3′-3′ linked nucleotide modification to the DNAzyme prevents exonuclease degradation, increasing stability [42]. Limitations that have been reported in the use of VEGF-A antibodies include a lack of neovascular membrane regression [43], [44], besides the more general requirement of multiple injections and potential risk of complications from intraocular injections [45]. An emerging approach is the VEGF Trap-Eye (Regeneron) which like ranibizumab and bevacizumab, binds and neutralizes VEGF but requires repeated administration [46]. c-Jun DNAzymes provide a possible alternative to antibody-based therapy, especially if combined with recently described ocular reservoir devices for local drug delivery [47].

The pathogenic course in OIR progresses in a relatively uniform pattern and is controlled by key regulatory mediators. Dz13 suppressed the expression of VEGF-A, FGF-2, MMP-2 and MMP-9. It also inhibited MMP-12 and MMP-13. Both these proteinases play a crucial role in tumor angiogenesis and tissue remodeling [48], [49]. MMP-12 is involved in the breakdown of extracellular matrix and tissue remodeling, while MMP-13 is involved in the breakdown of extracellular matrix-collagen type II, which is important in tumor invasion and metastasis. Targeting a key transcription factor such as c-Jun over a single gene alone (e.g., VEGF-A) provides the additional advantage of suppressing the expression of multiple effector genes, like those demonstrated here, indirectly, through inhibition of an upstream transcriptional regulator that is switched on in this pathology. The modified OIR model used in a curative setting here contrasts with Dz13’s prevention of retinal angiogenesis [20]. The fact that GSEA suggests that cancer-related gene sets are downregulated by Dz13 is consistent with Dz13 inhibition of endothelial cell proliferation and migration, cellular processes that underpin retinal neovascularization. This study also provides the first evidence that intravitreal administration of a DNAzyme confers a behavioral advantage to mice through forepaw extension. Such testing takes advantage of the forepaw extension reflex that occurs when a suspended animal is faced with a surface [26], [36] and provides a simple surrogate measure of visual resolution. Dz13 treated mice reached for the cage as well as normal mice, whereas the Dz13scr mice only partially recognized the top of the cage. Our findings, taken together, suggest that Dz13 may serve as a possible therapeutic option in the management of retinal vascularization.

## Supporting Information

Figure S1 **Microarray analysis was performed comparing the gene expression profile between Dz13, Dz13scr and vehicle (DOTAP/DOPE) groups at I-10 among 28815 probe sets on the Affymetrix gene chip**. Heat map color blue represents low expression; red represents high expression. **Top left,** The plot demonstrates fold-change for all probe sets on the array. Fold-changes are ranked from the most upregulated (left in red) to the most downregulated by Dz13 (right in blue). Most genes fall within a log2 fold-change of <0.5 with the boxed regions showing 251 probe sets with fold-changes >1.5. **Lower left,** GSEA showing 2 enrichment plots of the Lee and Mueller gene sets with a selection of genes downregulated by Dz13 within each set listed, including MMP-12 and MMP-13 which are highlighted.
(TIFF)

Table S1 **Curated gene sets with a Q-value (<0.05) downregulated by Dz13.** 230 gene sets had a Q-value less than 0.05.
(PDF)

Table S2 **Summarized curated gene set terms with a Q-value (<0.05) downregulated by Dz13.** Since names given to gene sets can be uninformative summarizing themes in collections of gene sets can be difficult. Here we used unique words in the names of each gene set and summarized the frequency of word occurrence. A complete list of all curated downregulated gene sets identified in GSEA is provided in Table S1.
(PDF)
