# Correction: Reduced Retinal Microvascular Density, Improved Forepaw Reach, Comparative Microarray and Gene Set Enrichment Analysis with c-jun Targeting DNA Enzyme

**DOI:** 10.1371/journal.pone.0349921

**Published:** 2026-05-26

**Authors:** Cecilia W. S. Chan, Warren Kaplan, Christopher R. Parish, Levon M. Khachigian

After this article [1] was published, the corresponding author informed the journal of an omission in the Competing Interests statement. Based on information gathered in editorial follow-up, the corrected Competing Interests statement is: LMK invented the DNAzyme Dz13 and is listed as an inventor on patent application US 10/923197, owned by NewSouth Innovations Pty Ltd, the Technology Transfer and Innovation Office of UNSW Australia (The University of New South Wales). This does not alter our adherence to *PLOS One* policies on sharing data and materials.

## References

[1] Chan CWS, Kaplan W, Parish CR, Khachigian LM. Reduced retinal microvascular density, improved forepaw reach, comparative microarray and gene set enrichment analysis with c-jun targeting DNA enzyme. PLoS One. 2012;7(7):e39160. doi: 10.1371/journal.pone.0039160 22815700 PMC3398922

