# Supplementary figures and images for: Reduced Retinal Microvascular Density, Improved Forepaw Reach, Comparative Microarray and Gene Set Enrichment Analysis with c-jun Targeting DNA Enzyme
Source: PLoS One. 2012 Jul 17;7(7):e39160. doi: 10.1371/journal.pone.0039160 (PMC3398922; doi:10.1371/journal.pone.0039160)

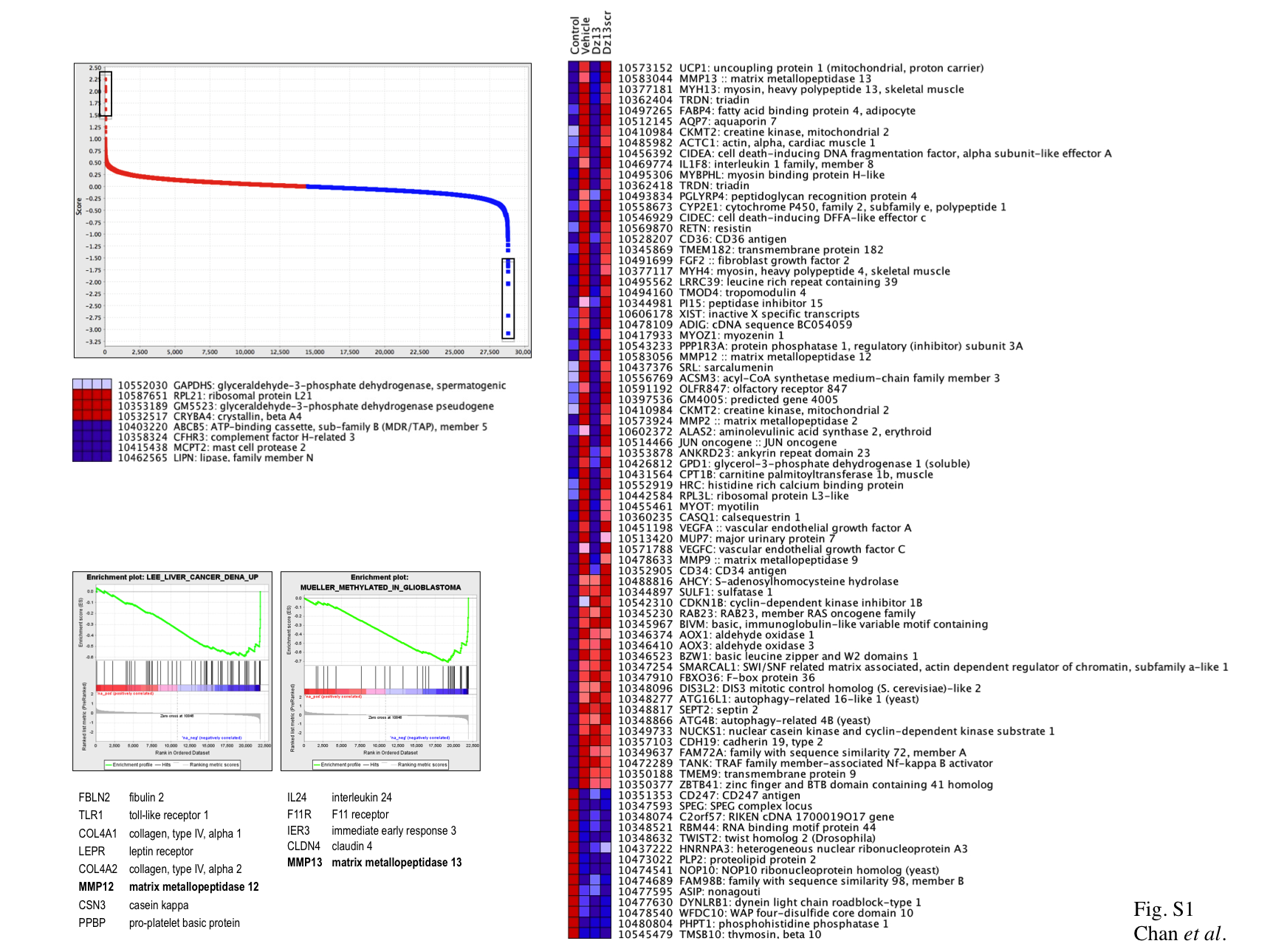

Supplement: Figure S1 — Microarray analysis was performed comparing the gene expression profile between Dz13, Dz13scr and vehicle (DOTAP/DOPE) groups at I-10 among 28815 probe sets on the Affymetrix gene chip. Heat map color blue represents low expression; red represents high expression. Top left, The plot demonstrates fold-change for all probe sets on the array. Fold-changes are ranked from the most upregulated (left in red) to the most downregulated by Dz13 (right in blue). Most genes fall within a log2 fold-change of <0.5 with the boxed regions showing 251 probe sets with fold-changes >1.5. Lower left, GSEA showing 2 enrichment plots of the Lee and Mueller gene sets with a selection of genes downregulated by Dz13 within each set listed, including MMP-12 and MMP-13 which are highlighted. (TIFF) [file pone.0039160.s001.tif]
