# Supplementary material for: Reduced Retinal Microvascular Density, Improved Forepaw Reach, Comparative Microarray and Gene Set Enrichment Analysis with c-jun Targeting DNA Enzyme
Source: PLoS One. 2012 Jul 17;7(7):e39160. doi: 10.1371/journal.pone.0039160 (PMC3398922; doi:10.1371/journal.pone.0039160)
Supplement: Table S1 — Curated gene sets with a Q-value (<0.05) downregulated by Dz13. 230 gene sets had a Q-value less than 0.05. (PDF) [file pone.0039160.s002.pdf]

Supplementary Table 1. **Curated gene sets with a Q-value (<0.05) downregulated by Dz13. 230 gene sets had a Q-value less than 0.05**.

NAME	GS<br>follow link to MSigDB	GS DETAILS	SIZE	ES	NES	NOM p-val	FDR q-val	FWER p-val	RANK AT MAX	LEADING EDGE
LEE_LIVER_CANCER_CIPROFIBRATE_UP	LEE_LIVER_CANCER_CIPROFIBRATE_UP	Details ...	55	-0.6948962	-2.620723	0	0	0	2042	tags=33%, list=9%, signal=36%
EBAUER_MYOGENIC_TARGETS_OF_PAX3_FOXO1_FUSION	EBAUER_MYOGENIC_TARGETS_OF_PAX3_FOXO1_	Details ...	48	-0.7161258	-2.5825858	0	0	0	3179	tags=58%, list=14%, signal=68%
RICKMAN_HEAD_AND_NECK_CANCER_F	RICKMAN_HEAD_AND_NECK_CANCER_F	Details ...	53	-0.7138477	-2.5638273	0	0	0	1709	tags=55%, list=8%, signal=59%
CHARAFE_BREAST_CANCER_BASAL_VS_MESENCHYMAL_U	CHARAFE_BREAST_CANCER_BASAL_VS_MESENCH	Details ...	109	-0.6166455	-2.4001365	0	0	0	4876	tags=57%, list=22%, signal=73%
CADWELL_ATG16L1_TARGETS_UP	CADWELL_ATG16L1_TARGETS_UP	Details ...	90	-0.5951139	-2.379971	0	0	0	1100	tags=21%, list=5%, signal=22%
HUPER_BREAST_BASAL_VS_LUMINAL_UP	HUPER_BREAST_BASAL_VS_LUMINAL_UP	Details ...	49	-0.6611484	-2.3502436	0	0	0	1667	tags=27%, list=8%, signal=29%
GRAHAM_CML_DIVIDING_VS_NORMAL_QUIESCENT_UP	GRAHAM_CML_DIVIDING_VS_NORMAL_QUIESCEN	Details ...	179	-0.5421623	-2.3466277	0	0	0	4334	tags=42%, list=20%, signal=52%
NAKAYAMA_SOFT_TISSUE_TUMORS_PCA2_DN	NAKAYAMA_SOFT_TISSUE_TUMORS_PCA2_DN	Details ...	74	-0.5811524	-2.3004036	0	0	0	3956	tags=39%, list=18%, signal=48%
JAEGER_METASTASIS_DN	JAEGER_METASTASIS_DN	Details ...	241	-0.4990002	-2.2863855	0	0	0	3039	tags=30%, list=14%, signal=34%
ROSTY_CERVICAL_CANCER_PROLIFERATION_CLUSTER	ROSTY_CERVICAL_CANCER_PROLIFERATION_CLUS	Details ...	140	-0.5319141	-2.281082	0	0	0	4160	tags=46%, list=19%, signal=56%
MUELLER_METHYLATED_IN_GLIOBLASTOMA	MUELLER_METHYLATED_IN_GLIOBLASTOMA	Details ...	34	-0.7076257	-2.2795048	0	0	0	2692	tags=53%, list=12%, signal=60%
CROMER_TUMORIGENESIS_DN	CROMER_TUMORIGENESIS_DN	Details ...	40	-0.7087664	-2.2759593	0	0	0	3611	tags=60%, list=16%, signal=72%
ICHIBA_GRAFT_VERSUS_HOST_DISEASE_35D_UP	ICHIBA_GRAFT_VERSUS_HOST_DISEASE_35D_U	Details ...	132	-0.5475313	-2.2735932	0	0	0	4723	tags=44%, list=22%, signal=56%
SMID_BREAST_CANCER_LUMINAL_A_UP	SMID_BREAST_CANCER_LUMINAL_A_UP	Details ...	80	-0.5594221	-2.254916	0	0	0	2363	tags=29%, list=11%, signal=32%
ONDER_CDH1_TARGETS_2_DN	ONDER_CDH1_TARGETS_2_DN	Details ...	443	-0.4622214	-2.2444956	0	0.00187395	0.02	4725	tags=38%, list=22%, signal=48%
LANDIS_ERBB2_BREAST_TUMORS_324_DN	LANDIS_ERBB2_BREAST_TUMORS_324_DN	Details ...	140	-0.5323506	-2.2429178	0	0.00175683	0.02	4720	tags=47%, list=22%, signal=60%
KUNINGER_IGF1_VS_PDGFB_TARGETS_UP	KUNINGER_IGF1_VS_PDGFB_TARGETS_UP	Details ...	42	-0.6764096	-2.23019	0	0.00165349	0.02	2973	tags=55%, list=14%, signal=63%
LIN_SILENCED_BY_TUMOR_MICROENVIRONMENT	LIN_SILENCED_BY_TUMOR_MICROENVIRONMENT	Details ...	91	-0.5753983	-2.2270133	0	0.00156163	0.02	4410	tags=49%, list=20%, signal=62%
LEE_LIVER_CANCER_DENA_UP	LEE_LIVER_CANCER_DENA_UP	Details ...	57	-0.5928865	-2.2191052	0	0.00147944	0.02	2042	tags=26%, list=9%, signal=29%
REACTOME_MUSCLE_CONTRACTION	REACTOME_MUSCLE_CONTRACTION	Details ...	50	-0.6169809	-2.2176723	0	0.00140547	0.02	4037	tags=50%, list=18%, signal=61%
MCBRYAN_PUBERTAL_BREAST_4_5WK_UP	MCBRYAN_PUBERTAL_BREAST_4_5WK_UP		249	-0.4856085	-2.2005122	0	0.00133854	0.02	2836	tags=30%, list=13%, signal=34%
YU_MYC_TARGETS_UP	YU_MYC_TARGETS_UP		37	-0.647736	-2.2004128	0	0.0012777	0.02	4604	tags=51%, list=21%, signal=65%
DOANE_BREAST_CANCER_ESR1_DN	DOANE_BREAST_CANCER_ESR1_DN		46	-0.6214032	-2.1977148	0	0.00122214	0.02	679	tags=15%, list=3%, signal=16%
CROONQUIST_IL6_DEPRIVATION_DN	CROONQUIST_IL6_DEPRIVATION_DN		78	-0.558601	-2.1967027	0	0.00117122	0.02	4531	tags=49%, list=21%, signal=61%
GRAHAM_NORMAL_QUIESCENT_VS_NORMAL_DIVIDING_D	GRAHAM_NORMAL_QUIESCENT_VS_NORMAL_DIVIDING_DN		86	-0.5671597	-2.1937878	0	0.00112437	0.02	5296	tags=56%, list=24%, signal=73%
KEGG_PPAR_SIGNALING_PATHWAY	KEGG_PPAR_SIGNALING_PATHWAY		68	-0.6274813	-2.192938	0	0.00108113	0.02	2481	tags=28%, list=11%, signal=31%
MCBRYAN_PUBERTAL_BREAST_4_5WK_DN	MCBRYAN_PUBERTAL_BREAST_4_5WK_DN		181	-0.4950546	-2.1849837	0	0.00104109	0.02	4391	tags=35%, list=20%, signal=44%
RICKMAN_METASTASIS_DN	RICKMAN_METASTASIS_DN		246	-0.4809478	-2.177633	0	0.0010039	0.02	5114	tags=46%, list=23%, signal=60%
LIAN_LIPA_TARGETS_6M	LIAN_LIPA_TARGETS_6M		78	-0.5913333	-2.1745753	0	9.69E-04	0.02	1980	tags=28%, list=9%, signal=31%
MCBRYAN_PUBERTAL_BREAST_3_4WK_UP	MCBRYAN_PUBERTAL_BREAST_3_4WK_UP		191	-0.5026783	-2.1654122	0	9.37E-04	0.02	4160	tags=36%, list=19%, signal=44%
GNATENKO_PLATELET_SIGNATURE	GNATENKO_PLATELET_SIGNATURE		32	-0.6573902	-2.1653821	0	9.07E-04	0.02	668	tags=16%, list=3%, signal=16%
REACTOME_STRIATED_MUSCLE_CONTRACTION	REACTOME_STRIATED_MUSCLE_CONTRACTION		31	-0.7135432	-2.160108	0	8.78E-04	0.02	2973	tags=58%, list=14%, signal=67%
RODWELL_AGING_KIDNEY_NO_BLOOD_UP	RODWELL_AGING_KIDNEY_NO_BLOOD_UP		142	-0.4852092	-2.149692	0	8.52E-04	0.02	4332	tags=38%, list=20%, signal=47%
CHIBA_RESPONSE_TO_TSA_UP	CHIBA_RESPONSE_TO_TSA_UP		42	-0.6335645	-2.1482043	0	8.27E-04	0.02	2414	tags=36%, list=11%, signal=40%
SOTIRIOU_BREAST_CANCER_GRADE_1_VS_3_UP	SOTIRIOU_BREAST_CANCER_GRADE_1_VS_3_UP		147	-0.5034542	-2.14118	0	8.03E-04	0.02	6339	tags=54%, list=29%, signal=75%
BIOCARTA_HSP27_PATHWAY	BIOCARTA_HSP27_PATHWAY		15	-0.7727583	-2.1373446	0	7.81E-04	0.02	3021	tags=67%, list=14%, signal=77%
LIU_VAV3_PROSTATE_CARCINOGENESIS_UP	LIU_VAV3_PROSTATE_CARCINOGENESIS_UP		85	-0.5482135	-2.1256077	0	7.60E-04	0.02	1699	tags=26%, list=8%, signal=28%
MCBRYAN_PUBERTAL_BREAST_6_7WK_DN	MCBRYAN_PUBERTAL_BREAST_6_7WK_DN		74	-0.5588515	-2.1237457	0	7.40E-04	0.02	1884	tags=28%, list=9%, signal=31%
HOFFMANN_LARGE_TO_SMALL_PRE_BII_LYMPHOCYTE_UP	HOFFMANN_LARGE_TO_SMALL_PRE_BII_LYMPHOCYTE_UP		95	-0.5251618	-2.119046	0	7.21E-04	0.02	5729	tags=48%, list=26%, signal=65%
ZHANG_RESPONSE_TO_IKK_INHIBITOR_AND_TNF_UP	ZHANG_RESPONSE_TO_IKK_INHIBITOR_AND_TNF_UP		204	-0.4600046	-2.1171916	0	7.03E-04	0.02	5151	tags=40%, list=23%, signal=51%
SENGUPTA_NASOPHARYNGEAL_CARCINOMA_WITH_LMP1_	SENGUPTA_NASOPHARYNGEAL_CARCINOMA_WITH_LMP1_DN		147	-0.484624	-2.1146939	0	6.86E-04	0.02	2121	tags=22%, list=10%, signal=25%
KORKOLA_TERATOMA	KORKOLA_TERATOMA		32	-0.641466	-2.1091242	0	6.69E-04	0.02	3069	tags=41%, list=14%, signal=47%
CHIANG_LIVER_CANCER_SUBCLASS_PROLIFERATION_UP	CHIANG_LIVER_CANCER_SUBCLASS_PROLIFERATION_UP		132	-0.5128451	-2.1032307	0	6.54E-04	0.02	4666	tags=44%, list=21%, signal=55%
GRAHAM_CML_QUIESCENT_VS_NORMAL_QUIESCENT_UP	GRAHAM_CML_QUIESCENT_VS_NORMAL_QUIESCENT_UP		85	-0.5573238	-2.0893452	0	0.00159954	0.05	3939	tags=42%, list=18%, signal=51%
ODONNELL_TARGETS_OF_MYC_AND_TFRC_DN	ODONNELL_TARGETS_OF_MYC_AND_TFRC_DN		44	-0.5482842	-2.0631504	0	0.00186656	0.06	3679	tags=43%, list=17%, signal=52%
WAMUNYOKOLI_OVARIAN_CANCER_LMP_UP	WAMUNYOKOLI_OVARIAN_CANCER_LMP_UP		254	-0.4530367	-2.0629504	0	0.00182598	0.06	5002	tags=39%, list=23%, signal=50%
SMID_BREAST_CANCER_ERBB2_UP	SMID_BREAST_CANCER_ERBB2_UP		128	-0.4909198	-2.0626252	0	0.00178713	0.06	4418	tags=38%, list=20%, signal=48%
MORI_LARGE_PRE_BII_LYMPHOCYTE_UP	MORI_LARGE_PRE_BII_LYMPHOCYTE_UP		51	-0.5848261	-2.0609012	0	0.0017499	0.06	5595	tags=55%, list=26%, signal=74%
EBAUER_TARGETS_OF_PAX3_FOXO1_FUSION_UP	EBAUER_TARGETS_OF_PAX3_FOXO1_FUSION_UP		196	-0.4665766	-2.0562062	0	0.00171418	0.06	3179	tags=32%, list=14%, signal=37%
KOBAYASHI_EGFR_SIGNALING_24HR_DN	KOBAYASHI_EGFR_SIGNALING_24HR_DN		246	-0.4479153	-2.0474267	0	0.00252517	0.09	5631	tags=43%, list=26%, signal=57%
LEE_LIVER_CANCER_ACOX1_UP	LEE_LIVER_CANCER_ACOX1_UP		56	-0.5766345	-2.0440943	0	0.00274245	0.1	2042	tags=21%, list=9%, signal=24%
TAKEDA_TARGETS_OF_NUP98_HOXA9_FUSION_3D_DN	TAKEDA_TARGETS_OF_NUP98_HOXA9_FUSION_3D_DN		26	-0.6376841	-2.0433555	0	0.00268971	0.1	1503	tags=19%, list=7%, signal=21%
CROONQUIST_NRAS_SIGNALING_DN	CROONQUIST_NRAS_SIGNALING_DN		61	-0.5359733	-2.040218	0	0.00263896	0.1	5194	tags=51%, list=24%, signal=66%
KANG_DOXORUBICIN_RESISTANCE_UP	KANG_DOXORUBICIN_RESISTANCE_UP		53	-0.566976	-2.0391934	0	0.00259009	0.1	6002	tags=60%, list=27%, signal=83%
MORI_IMMATURE_B_LYMPHOCYTE_DN	MORI_IMMATURE_B_LYMPHOCYTE_DN		53	-0.5811859	-2.03875	0	0.002543	0.1	5595	tags=57%, list=26%, signal=76%
LEE_LIVER_CANCER_MYC_E2F1_UP	LEE_LIVER_CANCER_MYC_E2F1_UP		53	-0.5448575	-2.0247128	0	0.0027444	0.11	2927	tags=28%, list=13%, signal=33%
LIN_APC_TARGETS	LIN_APC_TARGETS		51	-0.5902362	-2.020925	0	0.00269625	0.11	4751	tags=53%, list=22%, signal=67%
CREIGHTON_AKT1_SIGNALING_VIA_MTOR_UP	CREIGHTON_AKT1_SIGNALING_VIA_MTOR_UP		33	-0.5962808	-2.0177414	0	0.00288954	0.12	4929	tags=52%, list=22%, signal=66%
COLDREN_GEFITINIB_RESISTANCE_DN	COLDREN_GEFITINIB_RESISTANCE_DN		209	-0.4610066	-2.0161173	0	0.00284057	0.12	4887	tags=41%, list=22%, signal=52%
REACTOME_REGULATION_OF_LIPID_METABOLISM_BY_PER	REACTOME_REGULATION_OF_LIPID_METABOLISM_BY_PEROXIS		60	-0.5505033	-2.0140584	0	0.0030255	0.13	2301	tags=18%, list=10%, signal=20%
LEE_EARLY_T_LYMPHOCYTE_UP	LEE_EARLY_T_LYMPHOCYTE_UP		76	-0.5249588	-2.013262	0	0.0029759	0.13	3574	tags=42%, list=16%, signal=50%

CHANG_POU5F1_TARGETS_UP	CHANG_POU5F1_TARGETS_UP		15	-0.6562952	-2.0128117	0	0.0029279	0.13	3727	tags=47%, list=17%, signal=56%
WANG_BARRETTS_ESOPHAGUS_DN	WANG_BARRETTS_ESOPHAGUS_DN		25	-0.6301909	-2.0102649	0	0.00288142	0.13	2350	tags=36%, list=11%, signal=40%
CHIARADONNA_NEOPLASTIC_TRANSFORMATION_CDC25_D	CHIARADONNA_NEOPLASTIC_TRANSFORMATION_CDC25_DN		140	-0.4592807	-2.009273	0	0.0028364	0.13	4125	tags=34%, list=19%, signal=42%
SWEET_LUNG_CANCER_KRAS_DN	SWEET_LUNG_CANCER_KRAS_DN		410	-0.4240986	-2.007858	0	0.00279277	0.13	3381	tags=26%, list=15%, signal=30%
RHODES_UNDIFFERENTIATED_CANCER	RHODES_UNDIFFERENTIATED_CANCER		60	-0.5390779	-2.0059552	0	0.00296015	0.14	4808	tags=43%, list=22%, signal=55%
MARKEY_RB1_CHRONIC_LOF_DN	MARKEY_RB1_CHRONIC_LOF_DN		112	-0.4851702	-2.0047076	0	0.00291597	0.14	2717	tags=22%, list=12%, signal=25%
ENK_UV_RESPONSE_EPIDERMIS_UP	ENK_UV_RESPONSE_EPIDERMIS_UP		278	-0.4345448	-1.9774847	0	0.00453022	0.21	4336	tags=30%, list=20%, signal=37%
KOKKINAKIS_METHIONINE_DEPRIVATION_48HR_UP	KOKKINAKIS_METHIONINE_DEPRIVATION_48HR_UP		125	-0.4652236	-1.9732533	0	0.00467098	0.21	6164	tags=46%, list=28%, signal=64%
LANDIS_ERBB2_BREAST_PRENEOPLASTIC_DN	LANDIS_ERBB2_BREAST_PRENEOPLASTIC_DN		53	-0.5563756	-1.9724579	0	0.00460425	0.21	3846	tags=42%, list=18%, signal=50%
GOLDRATH_ANTIGEN_RESPONSE	GOLDRATH_ANTIGEN_RESPONSE		331	-0.415495	-1.9720473	0	0.00474361	0.22	4755	tags=37%, list=22%, signal=46%
SMID_BREAST_CANCER_LUMINAL_A_DN	SMID_BREAST_CANCER_LUMINAL_A_DN		18	-0.6962577	-1.9648211	0	0.00487182	0.23	2104	tags=28%, list=10%, signal=31%
VERRECCHIA_EARLY_RESPONSE_TO_TGFB1	VERRECCHIA_EARLY_RESPONSE_TO_TGFB1		50	-0.5809671	-1.9647548	0	0.00480508	0.23	5414	tags=56%, list=25%, signal=74%
TURASHVILI_BREAST_DUCTAL_CARCINOMA_VS_DUCTAL_N	TURASHVILI_BREAST_DUCTAL_CARCINOMA_VS_DUCTAL_NORMA		176	-0.4569757	-1.9640157	0	0.00493234	0.24	4106	tags=30%, list=19%, signal=37%
FARMER_BREAST_CANCER_CLUSTER_2	FARMER_BREAST_CANCER_CLUSTER_2		33	-0.5753829	-1.9586772	0	0.00523826	0.26	4334	tags=52%, list=20%, signal=64%
REACTOME_REGULATION_OF_INSULIN_LIKE_GROWTH_FAC	REACTOME_REGULATION_OF_INSULIN_LIKE_GROWTH_FACTOR_		15	-0.6859714	-1.957799	0	0.00516933	0.26	2088	tags=47%, list=10%, signal=52%
AIGNER_ZEB1_TARGETS	AIGNER_ZEB1_TARGETS		28	-0.6213976	-1.9531645	0	0.00584195	0.3	5077	tags=61%, list=23%, signal=79%
LANDIS_BREAST_CANCER_PROGRESSION_DN	LANDIS_BREAST_CANCER_PROGRESSION_DN		65	-0.5315996	-1.9528377	0	0.00576706	0.3	4029	tags=45%, list=18%, signal=54%
RICKMAN_TUMOR_DIFFERENTIATED_WELL_VS_MODERATE	RICKMAN_TUMOR_DIFFERENTIATED_WELL_VS_MODERATELY_DN		104	-0.4951286	-1.9512595	0	0.00569406	0.3	3057	tags=28%, list=14%, signal=32%
CROMER_TUMORIGENESIS_UP	CROMER_TUMORIGENESIS_UP		43	-0.5909309	-1.9507514	0	0.00598071	0.31	4331	tags=40%, list=20%, signal=49%
GARGALOVIC_RESPONSE_TO_OXIDIZED_PHOSPHOLIPIDS_	GARGALOVIC_RESPONSE_TO_OXIDIZED_PHOSPHOLIPIDS_TURQ		50	-0.5347695	-1.9476095	0	0.0069483	0.34	3280	tags=40%, list=15%, signal=47%
LE_EGR2_TARGETS_UP	LE_EGR2_TARGETS_UP		101	-0.4878051	-1.943116	0	0.0073747	0.36	4722	tags=41%, list=22%, signal=51%
KIM_RESPONSE_TO_TSA_AND_DECITABINE_UP	KIM_RESPONSE_TO_TSA_AND_DECITABINE_UP		120	-0.4794951	-1.9407797	0	0.0074583	0.36	3670	tags=34%, list=17%, signal=41%
RICKMAN_HEAD_AND_NECK_CANCER_E	RICKMAN_HEAD_AND_NECK_CANCER_E		82	-0.4962197	-1.9338189	0	0.00786862	0.38	3611	tags=35%, list=16%, signal=42%
DELYS_THYROID_CANCER_UP	DELYS_THYROID_CANCER_UP		383	-0.4052457	-1.9313258	0	0.00810592	0.39	3845	tags=30%, list=18%, signal=36%
ONDER_CDH1_TARGETS_3_DN	ONDER_CDH1_TARGETS_3_DN		49	-0.5327371	-1.930037	0	0.00801167	0.39	2621	tags=33%, list=12%, signal=37%
VECCHI_GASTRIC_CANCER_EARLY_UP	VECCHI_GASTRIC_CANCER_EARLY_UP		397	-0.4000905	-1.9255834	0	0.00840581	0.4	4334	tags=32%, list=20%, signal=40%
DASU_IL6_SIGNALING_UP	DASU_IL6_SIGNALING_UP		55	-0.5291644	-1.9252347	0	0.00831028	0.4	4578	tags=45%, list=21%, signal=57%
SMID_BREAST_CANCER_RELAPSE_IN_PLEURA_DN	SMID_BREAST_CANCER_RELAPSE_IN_PLEURA_DN		21	-0.6842411	-1.9242455	0	0.00837328	0.4	992	tags=29%, list=5%, signal=30%
KEGG_TIGHT_JUNCTION	KEGG_TIGHT_JUNCTION		128	-0.4733615	-1.9223193	0	0.0087443	0.41	5725	tags=44%, list=26%, signal=59%
YAO_TEMPORAL_RESPONSE_TO_PROGESTERONE_CLUSTER	YAO_TEMPORAL_RESPONSE_TO_PROGESTERONE_CLUSTER_1		72	-0.4981683	-1.9211732	0	0.00880242	0.41	4578	tags=36%, list=21%, signal=45%
CHARAFE_BREAST_CANCER_LUMINAL_VS_MESENCHYMAL_	CHARAFE_BREAST_CANCER_LUMINAL_VS_MESENCHYMAL_UP		421	-0.4111078	-1.9197247	0	0.00902133	0.42	4956	tags=39%, list=23%, signal=49%
AMIT_EGF_RESPONSE_480_HELA	AMIT_EGF_RESPONSE_480_HELA		156	-0.4515857	-1.9180748	0	0.00907978	0.43	4317	tags=37%, list=20%, signal=45%
VERRECCHIA_RESPONSE_TO_TGFB1_C1	VERRECCHIA_RESPONSE_TO_TGFB1_C1		18	-0.6717715	-1.9178269	0.018182	0.00913513	0.44	2459	tags=33%, list=11%, signal=38%
YAO_HOXA10_TARGETS_VIA_PROGESTERONE_UP	YAO_HOXA10_TARGETS_VIA_PROGESTERONE_UP		76	-0.5100758	-1.9172525	0	0.00918446	0.45	2306	tags=26%, list=11%, signal=29%
FOURNIER_ACINAR_DEVELOPMENT_LATE_2	FOURNIER_ACINAR_DEVELOPMENT_LATE_2		265	-0.4099003	-1.9162114	0	0.00923385	0.46	5595	tags=36%, list=26%, signal=48%
LEE_LIVER_CANCER_E2F1_UP	LEE_LIVER_CANCER_E2F1_UP		60	-0.5242515	-1.9095863	0	0.01029923	0.5	2839	tags=28%, list=13%, signal=32%
HAMAI_APOPTOSIS_VIA_TRAIL_DN	HAMAI_APOPTOSIS_VIA_TRAIL_DN		120	-0.4369467	-1.9055768	0	0.01105395	0.53	3806	tags=34%, list=17%, signal=41%
LY_AGING_MIDDLE_DN	LY_AGING_MIDDLE_DN		15	-0.7488517	-1.9008297	0	0.01136139	0.55	2371	tags=67%, list=11%, signal=75%
BERENJENO_TRANSFORMED_BY_RHOA_FOREVER_DN	BERENJENO_TRANSFORMED_BY_RHOA_FOREVER_DN		32	-0.5679145	-1.8925252	0	0.01223573	0.57	5811	tags=53%, list=26%, signal=72%
RICKMAN_HEAD_AND_NECK_CANCER_C	RICKMAN_HEAD_AND_NECK_CANCER_C		96	-0.4771103	-1.8903344	0	0.01238743	0.58	4589	tags=39%, list=21%, signal=49%
WU_CELL_MIGRATION	WU_CELL_MIGRATION		173	-0.4413008	-1.8841543	0	0.01348818	0.64	5183	tags=42%, list=24%, signal=54%
LIAN_LIPA_TARGETS_3M	LIAN_LIPA_TARGETS_3M		64	-0.5442935	-1.8821355	0	0.01349541	0.65	1980	tags=27%, list=9%, signal=29%
MCBRYAN_PUBERTAL_BREAST_5_6WK_UP	MCBRYAN_PUBERTAL_BREAST_5_6WK_UP		111	-0.4649934	-1.8815398	0	0.01363799	0.66	1953	tags=23%, list=9%, signal=25%
AMUNDSON_GAMMA_RADIATION_RESPONSE	AMUNDSON_GAMMA_RADIATION_RESPONSE		34	-0.5806825	-1.8802501	0	0.01377711	0.67	3848	tags=47%, list=18%, signal=57%
FRASOR_RESPONSE_TO_SERM_OR_FULVESTRANT_DN	FRASOR_RESPONSE_TO_SERM_OR_FULVESTRANT_DN		42	-0.5410633	-1.8778192	0	0.01404346	0.68	4263	tags=48%, list=19%, signal=59%
PARK_TRETINOIN_RESPONSE_AND_PML_RARA_FUSION	PARK_TRETINOIN_RESPONSE_AND_PML_RARA_FUSION		25	-0.6288276	-1.8688239	0	0.0150907	0.69	5468	tags=56%, list=25%, signal=75%
RHEIN_ALL_GLUCOCORTICOID_THERAPY_UP	RHEIN_ALL_GLUCOCORTICOID_THERAPY_UP		68	-0.5002174	-1.8687649	0	0.01495097	0.69	2363	tags=25%, list=11%, signal=28%
TANG_SENESCENCE_TP53_TARGETS_DN	TANG_SENESCENCE_TP53_TARGETS_DN		38	-0.5528093	-1.8675627	0	0.01506842	0.69	4666	tags=53%, list=21%, signal=67%
BIOCARTA_PML_PATHWAY	BIOCARTA_PML_PATHWAY		17	-0.6907986	-1.8667353	0	0.01532374	0.69	2581	tags=35%, list=12%, signal=40%
LINDSTEDT_DENDRITIC_CELL_MATURATION_D	LINDSTEDT_DENDRITIC_CELL_MATURATION_D		53	-0.4997129	-1.8633051	0	0.01543955	0.69	5831	tags=45%, list=27%, signal=62%
YAO_TEMPORAL_RESPONSE_TO_PROGESTERONE_CLUSTER	YAO_TEMPORAL_RESPONSE_TO_PROGESTERONE_CLUSTER_9		71	-0.4801607	-1.8606484	0	0.01567814	0.71	3717	tags=35%, list=17%, signal=42%
PAL_PRMT5_TARGETS_DN	PAL_PRMT5_TARGETS_DN		30	-0.6079	-1.8601867	0	0.01566129	0.71	312	tags=13%, list=1%, signal=14%
BERENJENO_TRANSFORMED_BY_RHOA_UP	BERENJENO_TRANSFORMED_BY_RHOA_UP		495	-0.3825739	-1.8594158	0	0.01589064	0.72	5208	tags=34%, list=24%, signal=43%
CROMER_METASTASIS_DN	CROMER_METASTASIS_DN		62	-0.505009	-1.8591939	0	0.01587595	0.73	5970	tags=47%, list=27%, signal=64%
NAKAYAMA_SOFT_TISSUE_TUMORS_PCA2_UP	NAKAYAMA_SOFT_TISSUE_TUMORS_PCA2_UP		86	-0.456615	-1.8587326	0	0.01586083	0.74	4049	tags=38%, list=18%, signal=47%
CROONQUIST_NRAS_VS_STROMAL_STIMULATION_DN	CROONQUIST_NRAS_VS_STROMAL_STIMULATION_DN		77	-0.4963014	-1.8559024	0	0.01620962	0.75	4350	tags=48%, list=20%, signal=60%
GAUSSMANN_MLL_AF4_FUSION_TARGETS_F_DN	GAUSSMANN_MLL_AF4_FUSION_TARGETS_F_DN		29	-0.5614895	-1.8527328	0	0.01702765	0.77	4820	tags=45%, list=22%, signal=57%
RICKMAN_TUMOR_DIFFERENTIATED_WELL_VS_POORLY_D	RICKMAN_TUMOR_DIFFERENTIATED_WELL_VS_POORLY_DN		357	-0.4106633	-1.8518978	0	0.01735375	0.79	4282	tags=28%, list=20%, signal=34%
MCMURRAY_TP53_HRAS_COOPERATION_RESPONSE_UP	MCMURRAY_TP53_HRAS_COOPERATION_RESPONSE_UP		27	-0.5633023	-1.8514661	0	0.01720914	0.79	3741	tags=41%, list=17%, signal=49%
DAIRKEE_CANCER_PRONE_RESPONSE_E2	DAIRKEE_CANCER_PRONE_RESPONSE_E2		27	-0.5643964	-1.851381	0	0.01706691	0.79	2422	tags=30%, list=11%, signal=33%
KUNINGER_IGF1_VS_PDGFB_TARGETS_DN	KUNINGER_IGF1_VS_PDGFB_TARGETS_DN		19	-0.6694082	-1.849774	0	0.01726912	0.79	4521	tags=58%, list=21%, signal=73%
SHEPARD_CRUSH_AND_BURN_MUTANT_UP	SHEPARD_CRUSH_AND_BURN_MUTANT_UP		138	-0.4475801	-1.84742	0	0.01747137	0.8	4748	tags=36%, list=22%, signal=46%
DACOSTA_UV_RESPONSE_VIA_ERCC3_UP	DACOSTA_UV_RESPONSE_VIA_ERCC3_UP		298	-0.4084904	-1.8467777	0	0.01744492	0.81	5374	tags=41%, list=25%, signal=53%
YAO_TEMPORAL_RESPONSE_TO_PROGESTERONE_CLUSTER	YAO_TEMPORAL_RESPONSE_TO_PROGESTERONE_CLUSTER_6		69	-0.4748433	-1.8400204	0	0.01832419	0.84	2379	tags=22%, list=11%, signal=24%

SCIAN_CELL_CYCLE_TARGETS_OF_TP53_AND_TP73_DN	SCIAN_CELL_CYCLE_TARGETS_OF_TP53_AND_TP73_DN	22	-0.635305	-1.8394359	0	0.01817875	0.84	4049	tags=45%, list=18%, signal=56%
KEGG_VIRAL_MYOCARDITIS	KEGG_VIRAL_MYOCARDITIS	62	-0.4937787	-1.8378111	0	0.01814232	0.84	1486	tags=18%, list=7%, signal=19%
SCHLOSSER_MYC_AND_SERUM_RESPONSE_SYNERGY	SCHLOSSER_MYC_AND_SERUM_RESPONSE_SYNERGY	31	-0.5820364	-1.8372325	0	0.01833307	0.84	2300	tags=29%, list=10%, signal=32%
TARTE_PLASMA_CELL_VS_B_LYMPHOCYTE_UP	TARTE_PLASMA_CELL_VS_B_LYMPHOCYTE_UP	71	-0.4736574	-1.8371071	0	0.01840703	0.84	4950	tags=37%, list=23%, signal=47%
LEE_LIVER_CANCER_ACOX1_DN	LEE_LIVER_CANCER_ACOX1_DN	61	-0.4792998	-1.8351978	0	0.01880322	0.86	1736	tags=16%, list=8%, signal=18%
KEGG_ADIPOCYTOKINE_SIGNALING_PATHWAY	KEGG_ADIPOCYTOKINE_SIGNALING_PATHWAY	67	-0.4782252	-1.8339154	0	0.01877281	0.86	2539	tags=22%, list=12%, signal=25%
POOLA_INVASIVE_BREAST_CANCER_UP	POOLA_INVASIVE_BREAST_CANCER_UP	255	-0.3908299	-1.8292438	0	0.01958526	0.88	5296	tags=38%, list=24%, signal=50%
FUJII_YBX1_TARGETS_DN	FUJII_YBX1_TARGETS_DN	136	-0.4234357	-1.8287392	0	0.01943801	0.88	4446	tags=32%, list=20%, signal=40%
LEE_LIVER_CANCER_MYC_TGFA_UP	LEE_LIVER_CANCER_MYC_TGFA_UP	58	-0.5014406	-1.8282932	0	0.01939901	0.88	4723	tags=36%, list=22%, signal=46%
KEGG_LEISHMANIA_INFECTION	KEGG_LEISHMANIA_INFECTION	60	-0.4844511	-1.8245859	0	0.01998179	0.9	4360	tags=43%, list=20%, signal=54%
WHITEFORD_PEDIATRIC_CANCER_MARKERS	WHITEFORD_PEDIATRIC_CANCER_MARKERS	91	-0.4905272	-1.8241837	0	0.01983486	0.9	4049	tags=40%, list=18%, signal=48%
REACTOME_RNA_POLYMERASE_I_PROMOTER_OPENING	REACTOME_RNA_POLYMERASE_I_PROMOTER_OPENING	34	-0.6008669	-1.8235235	0.018868	0.01969009	0.9	5584	tags=65%, list=25%, signal=87%
WU_HBX_TARGETS_2_UP	WU_HBX_TARGETS_2_UP	20	-0.6094332	-1.8207649	0	0.0203613	0.9	5838	tags=55%, list=27%, signal=75%
MISHRA_CARCINOMA_ASSOCIATED_FIBROBLAST_DN	MISHRA_CARCINOMA_ASSOCIATED_FIBROBLAST_DN	22	-0.5962562	-1.8205638	0	0.0203175	0.91	2256	tags=32%, list=10%, signal=35%
KERLEY_RESPONSE_TO_CISPLATIN_UP	KERLEY_RESPONSE_TO_CISPLATIN_UP	35	-0.5120994	-1.8180974	0	0.02077748	0.91	4838	tags=43%, list=22%, signal=55%
SCHUETZ_BREAST_CANCER_DUCTAL_INVASIVE_UP	SCHUETZ_BREAST_CANCER_DUCTAL_INVASIVE_UP	330	-0.384093	-1.814792	0	0.02123152	0.92	3987	tags=31%, list=18%, signal=37%
NING_CHRONIC_OBSTRUCTIVE_PULMONARY_DISEASE_UP	NING_CHRONIC_OBSTRUCTIVE_PULMONARY_DISEASE_UP	71	-0.469792	-1.8131789	0	0.02147454	0.92	4919	tags=41%, list=22%, signal=52%
SHEPARD_BMYB_TARGETS	SHEPARD_BMYB_TARGETS	59	-0.4930102	-1.813167	0	0.02132437	0.92	4133	tags=41%, list=19%, signal=50%
TSENG_ADIPOGENIC_POTENTIAL_DN	TSENG_ADIPOGENIC_POTENTIAL_DN	44	-0.5315586	-1.8122281	0	0.02146464	0.92	4597	tags=39%, list=21%, signal=49%
EGUCHI_CELL_CYCLE_RB1_TARGETS	EGUCHI_CELL_CYCLE_RB1_TARGETS	19	-0.6460689	-1.8104104	0	0.02199428	0.92	3574	tags=58%, list=16%, signal=69%
BOYLAN_MULTIPLE_MYELOMA_C_D_DN	BOYLAN_MULTIPLE_MYELOMA_C_D_DN	253	-0.4040485	-1.8081696	0	0.02251582	0.92	4396	tags=33%, list=20%, signal=41%
CHAUHAN_RESPONSE_TO_METHOXYESTRADIOL_UP	CHAUHAN_RESPONSE_TO_METHOXYESTRADIOL_UP	46	-0.5130003	-1.8054858	0	0.02331908	0.93	2181	tags=26%, list=10%, signal=29%
REACTOME_PACKAGING_OF_TELOMERE_ENDS	REACTOME_PACKAGING_OF_TELOMERE_ENDS	32	-0.540824	-1.8053216	0.016949	0.02325823	0.93	5584	tags=53%, list=25%, signal=71%
DACOSTA_UV_RESPONSE_VIA_ERCC3_COMMON_UP	DACOSTA_UV_RESPONSE_VIA_ERCC3_COMMON_UP	53	-0.502393	-1.8044643	0	0.02329025	0.93	3320	tags=40%, list=15%, signal=47%
SABATES_COLORECTAL_ADENOMA_UP	SABATES_COLORECTAL_ADENOMA_UP	120	-0.4295504	-1.8010019	0	0.02396927	0.95	4516	tags=37%, list=21%, signal=46%
KANG_IMMORTALIZED_BY_TERT_UP	KANG_IMMORTALIZED_BY_TERT_UP	81	-0.4541078	-1.8006117	0	0.02399627	0.95	1824	tags=25%, list=8%, signal=27%
MOOTHA_MITOCHONDRIA	MOOTHA_MITOCHONDRIA	432	-0.3831199	-1.8004367	0	0.02392933	0.95	7037	tags=44%, list=32%, signal=64%
FURUKAWA_DUSP6_TARGETS_PCI35_DN	FURUKAWA_DUSP6_TARGETS_PCI35_DN	66	-0.4647062	-1.7993988	0	0.02413928	0.95	4334	tags=41%, list=20%, signal=51%
SWEET_LUNG_CANCER_KRAS_UP	SWEET_LUNG_CANCER_KRAS_UP	458	-0.3653701	-1.798223	0	0.02434475	0.95	4781	tags=31%, list=22%, signal=39%
IVANOVA_HEMATOPOIESIS_LATE_PROGENITOR	IVANOVA_HEMATOPOIESIS_LATE_PROGENITOR	129	-0.4381229	-1.7973866	0	0.024644	0.95	5043	tags=36%, list=23%, signal=47%
WILCOX_PRESPONSE_TO_ROGESTERONE_DN	WILCOX_PRESPONSE_TO_ROGESTERONE_DN	59	-0.4623108	-1.7963349	0	0.02493938	0.96	2180	tags=29%, list=10%, signal=32%
YAMASHITA_METHYLATED_IN_PROSTATE_CANCER	YAMASHITA_METHYLATED_IN_PROSTATE_CANCER	58	-0.4710362	-1.7959155	0	0.02496366	0.96	2363	tags=19%, list=11%, signal=21%
NAKAMURA_CANCER_MICROENVIRONMENT_UP	NAKAMURA_CANCER_MICROENVIRONMENT_UP	22	-0.6183459	-1.7951951	0	0.02480566	0.96	3813	tags=59%, list=17%, signal=71%
BOQUEST_STEM_CELL_CULTURED_VS_FRESH_DN	BOQUEST_STEM_CELL_CULTURED_VS_FRESH_DN	30	-0.528938	-1.7947247	0	0.02491821	0.96	1856	tags=23%, list=8%, signal=25%
FLECHNER_BIOPSY_KIDNEY_TRANSPLANT_REJECTED_VS_	FLECHNER_BIOPSY_KIDNEY_TRANSPLANT_REJECTED_VS_OK_U	85	-0.4517129	-1.7933482	0	0.02537494	0.97	4818	tags=39%, list=22%, signal=50%
VALK_AML_CLUSTER_10	VALK_AML_CLUSTER_10	30	-0.523864	-1.7926456	0	0.02538832	0.97	2109	tags=27%, list=10%, signal=29%
DOANE_BREAST_CANCER_CLASSES_DN	DOANE_BREAST_CANCER_CLASSES_DN	33	-0.5423041	-1.7891815	0	0.02609639	0.97	5734	tags=48%, list=26%, signal=66%
MOOTHA_PGC	MOOTHA_PGC	322	-0.3822337	-1.7822748	0	0.02826628	0.97	4526	tags=30%, list=21%, signal=37%
RODWELL_AGING_KIDNEY_UP	RODWELL_AGING_KIDNEY_UP	314	-0.3877089	-1.7805219	0	0.02894403	0.97	4091	tags=31%, list=19%, signal=38%
LEE_METASTASIS_AND_ALTERNATIVE_SPLICING_UP	LEE_METASTASIS_AND_ALTERNATIVE_SPLICING_UP	73	-0.437889	-1.7787087	0	0.02961554	0.98	3619	tags=34%, list=17%, signal=41%
LY_AGING_OLD_DN	LY_AGING_OLD_DN	46	-0.5147099	-1.7737784	0	0.03147591	0.98	2509	tags=33%, list=11%, signal=37%
TONKS_TARGETS_OF_RUNX1_RUNX1T1_FUSION_HSC_DN	TONKS_TARGETS_OF_RUNX1_RUNX1T1_FUSION_HSC_DN	176	-0.3933319	-1.7709837	0	0.03238409	0.98	4923	tags=36%, list=22%, signal=46%
RASHI_RESPONSE_TO_IONIZING_RADIATION_2	RASHI_RESPONSE_TO_IONIZING_RADIATION_2	124	-0.4414028	-1.7701131	0	0.03260672	0.99	3833	tags=30%, list=17%, signal=36%
REACTOME_COMPLEMENT_CASCADE	REACTOME_COMPLEMENT_CASCADE	20	-0.6446277	-1.7670333	0.018868	0.03331976	0.99	4601	tags=60%, list=21%, signal=76%
KOBAYASHI_EGFR_SIGNALING_6HR_DN	KOBAYASHI_EGFR_SIGNALING_6HR_DN	18	-0.6078304	-1.7663103	0.023256	0.03337253	0.99	1007	tags=33%, list=5%, signal=35%
KEGG_ARACHIDONIC_ACID_METABOLISM	KEGG_ARACHIDONIC_ACID_METABOLISM	56	-0.4968942	-1.7656308	0	0.0333402	0.99	3476	tags=29%, list=16%, signal=34%
HESS_TARGETS_OF_HOXA9_AND_MEIS1_DN	HESS_TARGETS_OF_HOXA9_AND_MEIS1_DN	73	-0.4684164	-1.7635227	0	0.03363754	0.99	4468	tags=38%, list=20%, signal=48%
COWLING_MYCN_TARGETS	COWLING_MYCN_TARGETS	41	-0.5114936	-1.7629412	0	0.03376881	0.99	4331	tags=37%, list=20%, signal=46%
DAVICIONI_PAX_FOXO1_SIGNATURE_IN_ARMS_DN	DAVICIONI_PAX_FOXO1_SIGNATURE_IN_ARMS_DN	19	-0.6148139	-1.7602246	0.017857	0.03503295	0.99	3150	tags=47%, list=14%, signal=55%
CHEOK_RESPONSE_TO_HD_MTX_UP	CHEOK_RESPONSE_TO_HD_MTX_UP	17	-0.6318009	-1.759248	0	0.03531844	0.99	3410	tags=41%, list=16%, signal=49%
CASTELLANO_NRAS_TARGETS_UP	CASTELLANO_NRAS_TARGETS_UP	65	-0.4885145	-1.7574222	0	0.03567125	0.99	4248	tags=32%, list=19%, signal=40%
NIKOLSKY_BREAST_CANCER_1Q21_AMPLICON	NIKOLSKY_BREAST_CANCER_1Q21_AMPLICON	35	-0.5283982	-1.753945	0	0.03681611	0.99	5209	tags=49%, list=24%, signal=64%
ALCALAY_AML_BY_NPM1_LOCALIZATION_DN	ALCALAY_AML_BY_NPM1_LOCALIZATION_DN	178	-0.4000619	-1.7509282	0	0.03754605	0.99	4336	tags=32%, list=20%, signal=40%
REN_ALVEOLAR_RHABDOMYOSARCOMA_UP	REN_ALVEOLAR_RHABDOMYOSARCOMA_UP	98	-0.4437641	-1.7507967	0	0.03733629	0.99	1653	tags=20%, list=8%, signal=22%
VERHAAK_AML_WITH_NPM1_MUTATED_UP	VERHAAK_AML_WITH_NPM1_MUTATED_UP	164	-0.4082649	-1.7441844	0	0.0393128	0.99	4171	tags=29%, list=19%, signal=36%
BROWNE_HCMV_INFECTION_2HR_DN	BROWNE_HCMV_INFECTION_2HR_DN	49	-0.4923979	-1.7436711	0	0.03940635	0.99	3848	tags=37%, list=18%, signal=44%
DASU_IL6_SIGNALING_SCAR_UP	DASU_IL6_SIGNALING_SCAR_UP	29	-0.5702981	-1.7414205	0.019231	0.04004143	0.99	6991	tags=59%, list=32%, signal=86%
FINETTI_BREAST_CANCER_BASAL_VS_LUMINAL	FINETTI_BREAST_CANCER_BASAL_VS_LUMINAL	16	-0.6345744	-1.7412336	0	0.03997899	0.99	2869	tags=44%, list=13%, signal=50%
REN_ALVEOLAR_RHABDOMYOSARCOMA_DN	REN_ALVEOLAR_RHABDOMYOSARCOMA_DN	406	-0.3643292	-1.7398553	0	0.04014525	0.99	6849	tags=45%, list=31%, signal=64%
MARZEC_IL2_SIGNALING_UP	MARZEC_IL2_SIGNALING_UP	104	-0.4234864	-1.7396168	0	0.04000459	0.99	4765	tags=37%, list=22%, signal=46%
ICHIBA_GRAFT_VERSUS_HOST_DISEASE_D7_UP	ICHIBA_GRAFT_VERSUS_HOST_DISEASE_D7_UP	107	-0.4189917	-1.739307	0	0.03986397	0.99	4248	tags=36%, list=19%, signal=45%
KAAB_HEART_ATRIUM_VS_VENTRICLE_DN	KAAB_HEART_ATRIUM_VS_VENTRICLE_DN	250	-0.3844507	-1.7380205	0	0.04002832	0.99	3490	tags=26%, list=16%, signal=31%
ZHAN_MULTIPLE_MYELOMA_PR_UP	ZHAN_MULTIPLE_MYELOMA_PR_UP	43	-0.5431191	-1.7345871	0.018519	0.04093081	0.99	4246	tags=51%, list=19%, signal=63%
KOKKINAKIS_METHIONINE_DEPRIVATION_96HR_UP	KOKKINAKIS_METHIONINE_DEPRIVATION_96HR_UP	114	-0.4189214	-1.7333513	0	0.04116495	0.99	5372	tags=38%, list=24%, signal=50%

VALK_AML_CLUSTER_5	VALK_AML_CLUSTER_5		27	-0.5464405	-1.7328858	0.033898	0.04102185	0.99	6285	tags=59%, list=29%, signal=83%
ROY_WOUND_BLOOD_VESSEL_DN	ROY_WOUND_BLOOD_VESSEL_DN		18	-0.6459794	-1.7328453	0.039216	0.04080708	0.99	3570	tags=50%, list=16%, signal=60%
MCLACHLAN_DENTAL_CARIES_UP	MCLACHLAN_DENTAL_CARIES_UP		181	-0.3956396	-1.7313185	0	0.04125345	0.99	3782	tags=30%, list=17%, signal=36%
BIOCARTA_COMP_PATHWAY	BIOCARTA_COMP_PATHWAY		19	-0.5975098	-1.7304231	0.016667	0.04169307	0.99	4479	tags=58%, list=20%, signal=73%
DAZARD_UV_RESPONSE_CLUSTER_G1	DAZARD_UV_RESPONSE_CLUSTER_G1		36	-0.5118631	-1.7282398	0	0.04198641	0.99	2069	tags=19%, list=9%, signal=21%
BASAKI_YBX1_TARGETS_UP	BASAKI_YBX1_TARGETS_UP		273	-0.3804187	-1.7276001	0	0.04191285	0.99	5377	tags=38%, list=25%, signal=50%
LI_AMPLIFIED_IN_LUNG_CANCER	LI_AMPLIFIED_IN_LUNG_CANCER		161	-0.4232202	-1.7272344	0	0.04169901	0.99	2256	tags=19%, list=10%, signal=21%
GAL_LEUKEMIC_STEM_CELL_DN	GAL_LEUKEMIC_STEM_CELL_DN		221	-0.3796718	-1.7236679	0	0.04341928	0.99	4818	tags=34%, list=22%, signal=43%
BERTUCCI_INVASIVE_CARCINOMA_DUCTAL_VS_LOBULAR	BERTUCCI_INVASIVE_CARCINOMA_DUCTAL_VS_LOBULAR_DN		44	-0.4928179	-1.7224149	0	0.04362882	0.99	1366	tags=18%, list=6%, signal=19%
IVANOVA_HEMATOPOIESIS_MATURE_CELL	IVANOVA_HEMATOPOIESIS_MATURE_CELL		105	-0.41815	-1.7208514	0	0.0441201	0.99	4586	tags=33%, list=21%, signal=42%
KEGG_DILATED_CARDIOMYOPATHY	KEGG_DILATED_CARDIOMYOPATHY		88	-0.430304	-1.7206025	0	0.04418383	0.99	2123	tags=25%, list=10%, signal=28%
LANDIS_ERBB2_BREAST_TUMORS_65_DN	LANDIS_ERBB2_BREAST_TUMORS_65_DN		37	-0.5109888	-1.7203541	0	0.04396401	0.99	3283	tags=41%, list=15%, signal=48%
REACTOME_FORMATION_OF_PLATELET_PLUG	REACTOME_FORMATION_OF_PLATELET_PLUG		182	-0.3754033	-1.719107	0	0.04423761	0.99	4075	tags=28%, list=19%, signal=34%
SMID_BREAST_CANCER_RELAPSE_IN_BONE_DN	SMID_BREAST_CANCER_RELAPSE_IN_BONE_DN		285	-0.3707533	-1.7188836	0	0.04408667	0.99	3379	tags=21%, list=15%, signal=24%
REACTOME_TELOMERE_MAINTENANCE	REACTOME_TELOMERE_MAINTENANCE		60	-0.474831	-1.7157646	0.017544	0.0451082	0.99	5584	tags=48%, list=25%, signal=65%
FURUKAWA_DUSP6_TARGETS_PCI35_UP	FURUKAWA_DUSP6_TARGETS_PCI35_UP		57	-0.46913	-1.7151325	0	0.04516181	0.99	3103	tags=32%, list=14%, signal=37%
ODONNELL_TFRC_TARGETS_DN	ODONNELL_TFRC_TARGETS_DN		127	-0.4197095	-1.7142467	0	0.0451468	0.99	5016	tags=40%, list=23%, signal=52%
JAZAERI_BREAST_CANCER_BRCA1_VS_BRCA2_UP	JAZAERI_BREAST_CANCER_BRCA1_VS_BRCA2_UP		42	-0.510319	-1.7133039	0.019231	0.04513157	0.99	6509	tags=50%, list=30%, signal=71%
HEDENFALK_BREAST_CANCER_BRCA1_VS_BRCA2	HEDENFALK_BREAST_CANCER_BRCA1_VS_BRCA2		28	-0.5344072	-1.7130088	0	0.04505096	0.99	3006	tags=29%, list=14%, signal=33%
AMIT_SERUM_RESPONSE_240_MCF10A	AMIT_SERUM_RESPONSE_240_MCF10A		56	-0.4912737	-1.7122649	0	0.04530769	0.99	3710	tags=38%, list=17%, signal=45%
HUPER_BREAST_BASAL_VS_LUMINAL_DN	HUPER_BREAST_BASAL_VS_LUMINAL_DN		56	-0.4575947	-1.7120162	0	0.04523003	0.99	4301	tags=38%, list=20%, signal=47%
DORN_ADENOVIRUS_INFECTION_32HR_DN	DORN_ADENOVIRUS_INFECTION_32HR_DN		34	-0.5200323	-1.7088763	0	0.04613967	0.99	2638	tags=24%, list=12%, signal=27%
LENAOUR_DENDRITIC_CELL_MATURATION_UP	LENAOUR_DENDRITIC_CELL_MATURATION_UP		87	-0.4587148	-1.7085317	0	0.04612116	0.99	3042	tags=28%, list=14%, signal=32%
RIGGI_EWING_SARCOMA_PROGENITOR_DN	RIGGI_EWING_SARCOMA_PROGENITOR_DN		175	-0.3910086	-1.7061878	0	0.04708605	0.99	4383	tags=35%, list=20%, signal=43%
MARKEY_RB1_ACUTE_LOF_DN	MARKEY_RB1_ACUTE_LOF_DN		212	-0.4016326	-1.7052153	0	0.04732816	0.99	5538	tags=38%, list=25%, signal=51%
CREIGHTON_AKT1_SIGNALING_VIA_MTOR_DN	CREIGHTON_AKT1_SIGNALING_VIA_MTOR_DN		23	-0.5523239	-1.7051196	0.018868	0.04710803	0.99	3976	tags=35%, list=18%, signal=42%
WONG_ENDMETRIUM_CANCER_UP	WONG_ENDMETRIUM_CANCER_UP		17	-0.57072	-1.703398	0	0.04787283	0.99	1607	tags=24%, list=7%, signal=25%
VANTVEER_BREAST_CANCER_ESR1_DN	VANTVEER_BREAST_CANCER_ESR1_DN		219	-0.3872089	-1.7033114	0	0.04765222	0.99	5595	tags=32%, list=26%, signal=42%
GILDEA_METASTASIS	GILDEA_METASTASIS		28	-0.5265635	-1.7029132	0	0.04788341	0.99	4247	tags=46%, list=19%, signal=58%
SHEPARD_BMYB_MORPHOLINO_DN	SHEPARD_BMYB_MORPHOLINO_DN		157	-0.3940985	-1.7021046	0	0.04779232	0.99	4133	tags=32%, list=19%, signal=40%
MOOTHA_HUMAN_MITODB_6_2002	MOOTHA_HUMAN_MITODB_6_2002		416	-0.3540045	-1.7018571	0	0.04763982	0.99	7037	tags=45%, list=32%, signal=65%
BILD_HRAS_ONCOGENIC_SIGNATURE	BILD_HRAS_ONCOGENIC_SIGNATURE		235	-0.3853384	-1.698974	0	0.04831602	1	4794	tags=31%, list=22%, signal=40%
GU_PDEF_TARGETS_UP	GU_PDEF_TARGETS_UP		68	-0.442445	-1.6966476	0	0.0489853	1	3619	tags=32%, list=17%, signal=39%
DAVICIONI_MOLECULAR_ARMS_VS_ERMS_DN	DAVICIONI_MOLECULAR_ARMS_VS_ERMS_DN		173	-0.3940994	-1.6965241	0	0.04882857	1	5467	tags=39%, list=25%, signal=52%
SATO_SILENCED_BY_METHYLATION_IN_PANCREATIC_CANC	SATO_SILENCED_BY_METHYLATION_IN_PANCREATIC_CANCER_2		41	-0.5179186	-1.6961997	0	0.04873734	1	4740	tags=49%, list=22%, signal=62%
KEGG_SMALL_CELL_LUNG_CANCER	KEGG_SMALL_CELL_LUNG_CANCER		83	-0.4360434	-1.6961428	0	0.04852073	1	6434	tags=46%, list=29%, signal=65%
ZHAN_MULTIPLE_MYELOMA_DN	ZHAN_MULTIPLE_MYELOMA_DN		26	-0.496219	-1.6956998	0	0.04854939	1	3573	tags=35%, list=16%, signal=41%
BIOCARTA_CYTOKINE_PATHWAY	BIOCARTA_CYTOKINE_PATHWAY		20	-0.6006618	-1.6956407	0.020833	0.04833552	1	2135	tags=35%, list=10%, signal=39%
CHARAFE_BREAST_CANCER_LUMINAL_VS_BASAL_DN	CHARAFE_BREAST_CANCER_LUMINAL_VS_BASAL_DN		419	-0.3578853	-1.6955885	0	0.04812352	1	4594	tags=27%, list=21%, signal=33%
VERRECCHIA_RESPONSE_TO_TGFB1_C2	VERRECCHIA_RESPONSE_TO_TGFB1_C2		18	-0.6112106	-1.6942312	0	0.04889668	1	5414	tags=78%, list=25%, signal=103%
HINATA_NFKB_TARGETS_KERATINOCYTE_UP	HINATA_NFKB_TARGETS_KERATINOCYTE_UP		69	-0.4323836	-1.691984	0	0.04929102	1	5414	tags=38%, list=25%, signal=50%
HOFMANN_MYELODYSPLASTIC_SYNDROM_RISK_UP	HOFMANN_MYELODYSPLASTIC_SYNDROM_RISK_UP		18	-0.597379	-1.6893494	0.018868	0.05040591	1	5171	tags=56%, list=24%, signal=73%
HORIUCHI_WTAP_TARGETS_DN	HORIUCHI_WTAP_TARGETS_DN		295	-0.367333	-1.6888995	0	0.05060529	1	4760	tags=31%, list=22%, signal=38%
WANG_ESOPHAGUS_CANCER_VS_NORMAL_UP	WANG_ESOPHAGUS_CANCER_VS_NORMAL_UP		106	-0.3986946	-1.6878406	0	0.05080914	1	4899	tags=38%, list=22%, signal=48%
FAELT_B_CLL_WITH_VH3_21_UP	FAELT_B_CLL_WITH_VH3_21_UP		44	-0.478468	-1.6876241	0	0.05071059	1	6322	tags=59%, list=29%, signal=83%
FOURNIER_ACINAR_DEVELOPMENT_LATE_DN	FOURNIER_ACINAR_DEVELOPMENT_LATE_DN		21	-0.5696225	-1.686736	0	0.05109352	1	4334	tags=38%, list=20%, signal=47%
LI_LUNG_CANCER	LI_LUNG_CANCER		41	-0.4691536	-1.6864575	0	0.05087702	1	1142	tags=12%, list=5%, signal=13%
RIZ_ERYTHROID_DIFFERENTIATION_12HR	RIZ_ERYTHROID_DIFFERENTIATION_12HR		39	-0.4999355	-1.6843929	0.015873	0.05178451	1	3571	tags=36%, list=16%, signal=43%
DIAZ_CHRONIC_MEYLOGENOUS_LEUKEMIA_DN	DIAZ_CHRONIC_MEYLOGENOUS_LEUKEMIA_DN		107	-0.4250723	-1.6843854	0	0.05156693	1	3655	tags=30%, list=17%, signal=36%
FUJII_YBX1_TARGETS_UP	FUJII_YBX1_TARGETS_UP		24	-0.5420686	-1.6827148	0	0.05199318	1	4740	tags=42%, list=22%, signal=53%
WANG_BARRETTS_ESOPHAGUS_UP	WANG_BARRETTS_ESOPHAGUS_UP		40	-0.482012	-1.6826321	0	0.05177654	1	3191	tags=33%, list=15%, signal=38%
ENK_UV_RESPONSE_EPIDERMIS_DN	ENK_UV_RESPONSE_EPIDERMIS_DN		493	-0.3486018	-1.6812401	0	0.0521404	1	4057	tags=24%, list=19%, signal=29%
SIMBULAN_UV_RESPONSE_NORMAL_DN	SIMBULAN_UV_RESPONSE_NORMAL_DN		28	-0.539336	-1.6778613	0.016667	0.05366848	1	3946	tags=46%, list=18%, signal=57%
SHEPARD_CRUSH_AND_BURN_MUTANT_DN	SHEPARD_CRUSH_AND_BURN_MUTANT_DN		144	-0.3941511	-1.6776164	0	0.05373723	1	4531	tags=34%, list=21%, signal=43%
HOFMANN_MYELODYSPLASTIC_SYNDROM_LOW_RISK_DN	HOFMANN_MYELODYSPLASTIC_SYNDROM_LOW_RISK_DN		25	-0.5588273	-1.6753024	0	0.05437968	1	7281	tags=72%, list=33%, signal=108%
MCBRYAN_PUBERTAL_BREAST_3_4WK_DN	MCBRYAN_PUBERTAL_BREAST_3_4WK_DN		39	-0.4996161	-1.6744175	0	0.05449803	1	938	tags=21%, list=4%, signal=21%
WEIGEL_OXIDATIVE_STRESS_BY_HNE_AND_H2O2	WEIGEL_OXIDATIVE_STRESS_BY_HNE_AND_H2O2		34	-0.5035197	-1.6738374	0	0.05473119	1	5218	tags=41%, list=24%, signal=54%
LINDGREN_BLADDER_CANCER_CLUSTER_1_DN	LINDGREN_BLADDER_CANCER_CLUSTER_1_DN		359	-0.3545336	-1.6692654	0	0.05735074	1	5445	tags=33%, list=25%, signal=43%
YAUCH_HEDGEHOG_SIGNALING_PARACRINE_DN	YAUCH_HEDGEHOG_SIGNALING_PARACRINE_DN		258	-0.3647355	-1.6679338	0	0.05762877	1	3102	tags=23%, list=14%, signal=26%
AMIT_SERUM_RESPONSE_480_MCF10A	AMIT_SERUM_RESPONSE_480_MCF10A		35	-0.5335646	-1.6674265	0.0625	0.05779171	1	4906	tags=46%, list=22%, signal=59%
TSENG_IRS1_TARGETS_DN	TSENG_IRS1_TARGETS_DN		128	-0.3991358	-1.667063	0	0.05790028	1	4854	tags=30%, list=22%, signal=39%
BIOCARTA_GATA3_PATHWAY	BIOCARTA_GATA3_PATHWAY		15	-0.6337317	-1.6666616	0.018182	0.05778297	1	4795	tags=53%, list=22%, signal=68%
MACLACHLAN_BRCA1_TARGETS_UP	MACLACHLAN_BRCA1_TARGETS_UP		17	-0.6301429	-1.6665875	0	0.05755367	1	5162	tags=53%, list=24%, signal=69%
KEGG_ADHERENS_JUNCTION	KEGG_ADHERENS_JUNCTION		73	-0.4274643	-1.6663748	0.016949	0.05743897	1	5301	tags=41%, list=24%, signal=54%

REACTOME_HEMOSTASIS	REACTOME_HEMOSTASIS		266	-0.3526111	-1.6639918	0	0.05903444	1	4980	tags=31%, list=23%, signal=40%
BOYAULT_LIVER_CANCER_SUBCLASS_G23_UP	BOYAULT_LIVER_CANCER_SUBCLASS_G23_UP		52	-0.4799479	-1.6634345	0	0.05896694	1	4436	tags=40%, list=20%, signal=51%
WEIGEL_OXIDATIVE_STRESS_RESPONSE	WEIGEL_OXIDATIVE_STRESS_RESPONSE		29	-0.5287786	-1.6628497	0	0.05912412	1	3946	tags=34%, list=18%, signal=42%
WU_HBX_TARGETS_3_UP	WU_HBX_TARGETS_3_UP		17	-0.5488579	-1.6603527	0.071429	0.06037144	1	6373	tags=59%, list=29%, signal=83%
FINETTI_BREAST_CANCER_KINOME_RED	FINETTI_BREAST_CANCER_KINOME_RED		15	-0.6025277	-1.6602765	0.015385	0.06013744	1	2869	tags=40%, list=13%, signal=46%
LEE_LIVER_CANCER_CIPROFIBRATE_DN	LEE_LIVER_CANCER_CIPROFIBRATE_DN		62	-0.4492036	-1.6592641	0	0.06034101	1	471	tags=15%, list=2%, signal=15%
DUTTA_APOPTOSIS_VIA_NFKB	DUTTA_APOPTOSIS_VIA_NFKB		29	-0.5430533	-1.657946	0	0.06075361	1	4654	tags=38%, list=21%, signal=48%
SMID_BREAST_CANCER_RELAPSE_IN_LUNG_DN	SMID_BREAST_CANCER_RELAPSE_IN_LUNG_DN		32	-0.5113667	-1.6578327	0.016949	0.06057423	1	2238	tags=22%, list=10%, signal=24%
MULLIGHAN_MLL_SIGNATURE_1_UP	MULLIGHAN_MLL_SIGNATURE_1_UP		360	-0.3530844	-1.6575519	0	0.06055536	1	5386	tags=36%, list=25%, signal=47%
LIU_CDX2_TARGETS_UP	LIU_CDX2_TARGETS_UP		35	-0.467888	-1.6571758	0.016667	0.06064446	1	2810	tags=29%, list=13%, signal=33%
OLSSON_E2F3_TARGETS_UP	OLSSON_E2F3_TARGETS_UP		20	-0.5683253	-1.6564269	0	0.06084064	1	5372	tags=55%, list=24%, signal=73%
GERY_CEBP_TARGETS	GERY_CEBP_TARGETS		123	-0.4047171	-1.6558379	0	0.06087508	1	2873	tags=24%, list=13%, signal=27%
MARKS_HDAC_TARGETS_UP	MARKS_HDAC_TARGETS_UP		20	-0.5720867	-1.6551316	0.019231	0.06090873	1	5497	tags=60%, list=25%, signal=80%
KEGG_SYSTEMIC_LUPUS_ERYTHEMATOSUS	KEGG_SYSTEMIC_LUPUS_ERYTHEMATOSUS		88	-0.4239562	-1.6544522	0.016129	0.06089154	1	4676	tags=42%, list=21%, signal=53%
KENNY_CTNNB1_TARGETS_UP	KENNY_CTNNB1_TARGETS_UP		45	-0.4748687	-1.6540669	0	0.06082228	1	5008	tags=33%, list=23%, signal=43%
NAKAYAMA_SOFT_TISSUE_TUMORS_PCA1_UP	NAKAYAMA_SOFT_TISSUE_TUMORS_PCA1_UP		64	-0.4356445	-1.6538297	0	0.060649	1	4187	tags=38%, list=19%, signal=46%
LIANG_SILENCED_BY_METHYLATION_UP	LIANG_SILENCED_BY_METHYLATION_UP		19	-0.5884918	-1.6534016	0.037736	0.06068077	1	3568	tags=47%, list=16%, signal=57%
YOKOE_CANCER_TESTIS_ANTIGENS	YOKOE_CANCER_TESTIS_ANTIGENS		17	-0.6090589	-1.6533811	0	0.06045685	1	3206	tags=35%, list=15%, signal=41%
SPIELMAN_LYMPHOBLAST_EUROPEAN_VS_ASIAN_UP	SPIELMAN_LYMPHOBLAST_EUROPEAN_VS_ASIAN_UP		461	-0.336482	-1.653005	0	0.06038833	1	5666	tags=33%, list=26%, signal=43%
SWEET_KRAS_ONCOGENIC_SIGNATURE	SWEET_KRAS_ONCOGENIC_SIGNATURE		43	-0.471509	-1.652625	0	0.06031774	1	4740	tags=42%, list=22%, signal=53%
RAGHAVACHARI_PLATELET_SPECIFIC_GENES	RAGHAVACHARI_PLATELET_SPECIFIC_GENES		64	-0.4556654	-1.6524764	0.02	0.06009761	1	3550	tags=27%, list=16%, signal=32%
MOLENAAR_TARGETS_OF_CCND1_AND_CDK4_DN	MOLENAAR_TARGETS_OF_CCND1_AND_CDK4_DN		57	-0.4433067	-1.6516644	0.018868	0.06018477	1	5194	tags=39%, list=24%, signal=50%
NAGASHIMA_NRG1_SIGNALING_UP	NAGASHIMA_NRG1_SIGNALING_UP		168	-0.373046	-1.6474984	0	0.06190225	1	4578	tags=29%, list=21%, signal=37%
SCHLOSSER_SERUM_RESPONSE_AUGMENTED_BY_MYC	SCHLOSSER_SERUM_RESPONSE_AUGMENTED_BY_MYC		104	-0.4117623	-1.646905	0	0.06198216	1	4358	tags=32%, list=20%, signal=39%
CHANG_CYCLING_GENES	CHANG_CYCLING_GENES		46	-0.472462	-1.646428	0	0.06196073	1	5112	tags=43%, list=23%, signal=57%
BYSTRYKH_HEMATOPOIESIS_STEM_CELL_AND_BRAIN_QTL	BYSTRYKH_HEMATOPOIESIS_STEM_CELL_AND_BRAIN_QTL_TRA		169	-0.3726567	-1.6455172	0	0.06254306	1	3842	tags=21%, list=18%, signal=26%
REACTOME_ACTIVATED_AMPK_STIMULATES_FATTY_ACID_(	REACTOME_ACTIVATED_AMPK_STIMULATES_FATTY_ACID_OXIDA		17	-0.5764181	-1.6427019	0.017857	0.0635221	1	1341	tags=24%, list=6%, signal=25%
COATES_MACROPHAGE_M1_VS_M2_DN	COATES_MACROPHAGE_M1_VS_M2_DN		71	-0.4295417	-1.6426891	0.017857	0.06329604	1	3859	tags=34%, list=18%, signal=41%
TAKEDA_TARGETS_OF_NUP98_HOXA9_FUSION_8D_DN	TAKEDA_TARGETS_OF_NUP98_HOXA9_FUSION_8D_DN		179	-0.3721933	-1.6407243	0	0.06426057	1	2812	tags=19%, list=13%, signal=22%
NING_CHRONIC_OBSTRUCTIVE_PULMONARY_DISEASE_DN	NING_CHRONIC_OBSTRUCTIVE_PULMONARY_DISEASE_DN		59	-0.4549091	-1.64018	0.015873	0.06432993	1	2559	tags=22%, list=12%, signal=25%
CHANDRAN_METASTASIS_DN	CHANDRAN_METASTASIS_DN		88	-0.4138623	-1.6398693	0	0.06430579	1	3845	tags=26%, list=18%, signal=32%
ZHAN_V1_LATE_DIFFERENTIATION_GENES_UP	ZHAN_V1_LATE_DIFFERENTIATION_GENES_UP		30	-0.5193133	-1.638056	0	0.06536692	1	3489	tags=27%, list=16%, signal=32%
KEGG_GLUTATHIONE_METABOLISM	KEGG_GLUTATHIONE_METABOLISM		48	-0.477944	-1.6371744	0	0.06552792	1	4405	tags=40%, list=20%, signal=49%
MULLIGHAN_MLL_SIGNATURE_2_UP	MULLIGHAN_MLL_SIGNATURE_2_UP		396	-0.3363435	-1.6362661	0	0.0655396	1	5695	tags=37%, list=26%, signal=49%
NADERI_BREAST_CANCER_PROGNOSIS_UP	NADERI_BREAST_CANCER_PROGNOSIS_UP		36	-0.4830875	-1.6358175	0	0.06550883	1	4334	tags=42%, list=20%, signal=52%
BIOCARTA_MTA3_PATHWAY	BIOCARTA_MTA3_PATHWAY		17	-0.5718561	-1.634325	0	0.06591199	1	3145	tags=29%, list=14%, signal=34%
XU_HGF_SIGNALING_NOT_VIA_AKT1_6HR	XU_HGF_SIGNALING_NOT_VIA_AKT1_6HR		23	-0.529343	-1.6318991	0	0.06699193	1	738	tags=17%, list=3%, signal=18%
KEGG_APOPTOSIS	KEGG_APOPTOSIS		82	-0.4168232	-1.6311635	0	0.0673442	1	4177	tags=28%, list=19%, signal=35%
BARIS_THYROID_CANCER_DN	BARIS_THYROID_CANCER_DN		51	-0.4405959	-1.6297059	0	0.06778954	1	4037	tags=35%, list=18%, signal=43%
BAELDE_DIABETIC_NEPHROPATHY_UP	BAELDE_DIABETIC_NEPHROPATHY_UP		30	-0.5015437	-1.6289653	0.017241	0.06808056	1	4718	tags=50%, list=22%, signal=64%
VECCHI_GASTRIC_CANCER_ADVANCED_VS_EARLY_DN	VECCHI_GASTRIC_CANCER_ADVANCED_VS_EARLY_DN		124	-0.3968367	-1.628064	0	0.06861946	1	3191	tags=29%, list=15%, signal=34%
SHEPARD_BMYB_MORPHOLINO_UP	SHEPARD_BMYB_MORPHOLINO_UP		144	-0.3827425	-1.6246146	0	0.07042699	1	6672	tags=43%, list=30%, signal=61%
DAVICIONI_TARGETS_OF_PAX_FOXO1_FUSIONS_DN	DAVICIONI_TARGETS_OF_PAX_FOXO1_FUSIONS_DN		67	-0.4305574	-1.623609	0.018868	0.07094394	1	3350	tags=27%, list=15%, signal=32%
JEON_SMAD6_TARGETS_DN	JEON_SMAD6_TARGETS_DN		18	-0.581287	-1.6230668	0	0.0708482	1	2326	tags=39%, list=11%, signal=43%
TURASHVILI_BREAST_LOBULAR_CARCINOMA_VS_LOBULAR	TURASHVILI_BREAST_LOBULAR_CARCINOMA_VS_LOBULAR_NOR		90	-0.4073256	-1.6225624	0	0.07103328	1	4106	tags=29%, list=19%, signal=35%
DORN_ADENOVIRUS_INFECTION_48HR_DN	DORN_ADENOVIRUS_INFECTION_48HR_DN		34	-0.5159876	-1.6216522	0.019231	0.07117273	1	1007	tags=18%, list=5%, signal=18%
DORN_ADENOVIRUS_INFECTION_12HR_UP	DORN_ADENOVIRUS_INFECTION_12HR_UP		24	-0.5367147	-1.6190007	0	0.07220122	1	799	tags=17%, list=4%, signal=17%
URS_ADIPOCYTE_DIFFERENTIATION_UP	URS_ADIPOCYTE_DIFFERENTIATION_UP		61	-0.4342515	-1.6184621	0	0.07233694	1	3987	tags=31%, list=18%, signal=38%
BIOCARTA_NTHI_PATHWAY	BIOCARTA_NTHI_PATHWAY		23	-0.5760951	-1.6181554	0.017544	0.07228711	1	4938	tags=43%, list=23%, signal=56%
REACTOME_METABOLISM_OF_LIPIDS_AND_LIPOPROTEINS	REACTOME_METABOLISM_OF_LIPIDS_AND_LIPOPROTEINS		223	-0.3507079	-1.6181445	0	0.07204853	1	4293	tags=26%, list=20%, signal=33%
KAYO_CALORIE_RESTRICTION_MUSCLE_UP	KAYO_CALORIE_RESTRICTION_MUSCLE_UP		66	-0.4267524	-1.6174843	0	0.07213805	1	5580	tags=44%, list=25%, signal=59%
CAFFAREL_RESPONSE_TO_THC_24HR_5_DN	CAFFAREL_RESPONSE_TO_THC_24HR_5_DN		43	-0.4751873	-1.6158953	0.016129	0.07309841	1	4980	tags=44%, list=23%, signal=57%
SWEET_KRAS_TARGETS_DN	SWEET_KRAS_TARGETS_DN		25	-0.497595	-1.6157738	0	0.07290513	1	4740	tags=48%, list=22%, signal=61%
ACEVEDO_LIVER_CANCER_WITH_H3K9ME3_UP	ACEVEDO_LIVER_CANCER_WITH_H3K9ME3_UP		117	-0.3879732	-1.6132867	0.017544	0.07462783	1	3572	tags=24%, list=16%, signal=28%
BIOCARTA_PPARA_PATHWAY	BIOCARTA_PPARA_PATHWAY		56	-0.4545253	-1.6123949	0	0.07479337	1	2861	tags=23%, list=13%, signal=27%
LY_AGING_PREMATURE_DN	LY_AGING_PREMATURE_DN		23	-0.540278	-1.6110457	0	0.07518902	1	2371	tags=35%, list=11%, signal=39%
REACTOME_PLATELET_ACTIVATION	REACTOME_PLATELET_ACTIVATION		163	-0.3787869	-1.6097714	0	0.07548687	1	4075	tags=28%, list=19%, signal=34%
KEGG_P53_SIGNALING_PATHWAY	KEGG_P53_SIGNALING_PATHWAY		64	-0.440888	-1.6095762	0	0.07533301	1	5734	tags=45%, list=26%, signal=61%
WILENSKY_RESPONSE_TO_DARAPLADIB	WILENSKY_RESPONSE_TO_DARAPLADIB		26	-0.5344607	-1.6093937	0.036364	0.07522832	1	7658	tags=69%, list=35%, signal=106%
LEE_LIVER_CANCER_HEPATOBLAST	LEE_LIVER_CANCER_HEPATOBLAST		15	-0.6264007	-1.6092528	0	0.07503337	1	2250	tags=33%, list=10%, signal=37%
KLEIN_TARGETS_OF_BCR_ABL1_FUSION	KLEIN_TARGETS_OF_BCR_ABL1_FUSION		29	-0.4901676	-1.6090976	0.017544	0.0748832	1	2749	tags=31%, list=13%, signal=35%
WELCSH_BRCA1_TARGETS_1_DN	WELCSH_BRCA1_TARGETS_1_DN		122	-0.3759288	-1.608764	0	0.07482112	1	4332	tags=30%, list=20%, signal=38%
DORSAM_HOXA9_TARGETS_DN	DORSAM_HOXA9_TARGETS_DN		30	-0.496708	-1.6082308	0.035088	0.07485219	1	5664	tags=40%, list=26%, signal=54%
OUYANG_PROSTATE_CANCER_MARKERS	OUYANG_PROSTATE_CANCER_MARKERS		20	-0.5394219	-1.6077894	0.018519	0.07488026	1	5281	tags=50%, list=24%, signal=66%

LANDIS_ERBB2_BREAST_TUMORS_324_UP	LANDIS_ERBB2_BREAST_TUMORS_324_UP		135	-0.3811098	-1.6073058	0	0.07490879	1	6441	tags=41%, list=29%, signal=57%
LENAOUR_DENDRITIC_CELL_MATURATION_DN	LENAOUR_DENDRITIC_CELL_MATURATION_DN		96	-0.3902676	-1.606735	0	0.07507007	1	6827	tags=46%, list=31%, signal=66%
HOSHIDA_LIVER_CANCER_SUBCLASS_S1	HOSHIDA_LIVER_CANCER_SUBCLASS_S1		224	-0.3586507	-1.6063838	0	0.07492228	1	4833	tags=32%, list=22%, signal=40%
TSAI_RESPONSE_TO_IONIZING_RADIATION	TSAI_RESPONSE_TO_IONIZING_RADIATION		116	-0.3893074	-1.60612	0	0.07473198	1	4765	tags=34%, list=22%, signal=43%
MISSIAGLIA_REGULATED_BY_METHYLATION_UP	MISSIAGLIA_REGULATED_BY_METHYLATION_UP		90	-0.4146782	-1.6057116	0	0.07458928	1	6848	tags=53%, list=31%, signal=77%
KHETCHOUMIAN_TRIM24_TARGETS_UP	KHETCHOUMIAN_TRIM24_TARGETS_UP		45	-0.4552615	-1.6053584	0.020408	0.07448854	1	5413	tags=47%, list=25%, signal=62%
MORI_MATURE_B_LYMPHOCYTE_DN	MORI_MATURE_B_LYMPHOCYTE_DN		56	-0.4424461	-1.6050315	0	0.07438892	1	4764	tags=38%, list=22%, signal=48%
WEIGEL_OXIDATIVE_STRESS_BY_HNE_AND_TBH	WEIGEL_OXIDATIVE_STRESS_BY_HNE_AND_TBH		56	-0.4512036	-1.6042197	0	0.0745474	1	4752	tags=36%, list=22%, signal=45%
REACTOME_PLATELET_DEGRANULATION	REACTOME_PLATELET_DEGRANULATION		84	-0.4223388	-1.6036692	0	0.07470503	1	4265	tags=30%, list=19%, signal=37%
BERENJENO_TRANSFORMED_BY_RHOA_DN	BERENJENO_TRANSFORMED_BY_RHOA_DN		357	-0.3480214	-1.6030959	0	0.07469046	1	7147	tags=44%, list=33%, signal=64%
REN_BOUND_BY_E2F	REN_BOUND_BY_E2F		47	-0.4616551	-1.6020547	0.021739	0.07501742	1	5631	tags=47%, list=26%, signal=63%
CHIARADONNA_NEOPLASTIC_TRANSFORMATION_KRAS_DN	CHIARADONNA_NEOPLASTIC_TRANSFORMATION_KRAS_DN		133	-0.3721216	-1.6010853	0	0.07529713	1	4130	tags=30%, list=19%, signal=37%
CHIARADONNA_NEOPLASTIC_TRANSFORMATION_CDC25_U	CHIARADONNA_NEOPLASTIC_TRANSFORMATION_CDC25_UP		115	-0.3939295	-1.5995884	0	0.07579422	1	4362	tags=34%, list=20%, signal=42%
LEE_TARGETS_OF_PTCH1_AND_SUFU_DN	LEE_TARGETS_OF_PTCH1_AND_SUFU_DN		54	-0.4402514	-1.5989648	0	0.07586432	1	6193	tags=44%, list=28%, signal=62%
BIDUS_METASTASIS_DN	BIDUS_METASTASIS_DN		145	-0.3795213	-1.5951686	0	0.07790888	1	5235	tags=32%, list=24%, signal=41%
MCLACHLAN_DENTAL_CARIES_DN	MCLACHLAN_DENTAL_CARIES_DN		205	-0.3564415	-1.5948849	0	0.07784341	1	3782	tags=28%, list=17%, signal=33%
ROSS_AML_WITH_CBFB_MYH11_FUSION	ROSS_AML_WITH_CBFB_MYH11_FUSION		48	-0.4432333	-1.594757	0.02	0.07769375	1	4195	tags=35%, list=19%, signal=44%
MOREAUX_MULTIPLE_MYELOMA_BY_TACI_UP	MOREAUX_MULTIPLE_MYELOMA_BY_TACI_UP		338	-0.3369661	-1.5939941	0	0.07779682	1	3709	tags=25%, list=17%, signal=29%
TURASHVILI_BREAST_CARCINOMA_DUCTAL_VS_LOBULAR	TURASHVILI_BREAST_CARCINOMA_DUCTAL_VS_LOBULAR_UP		17	-0.6043949	-1.5935075	0.033898	0.07781683	1	2615	tags=41%, list=12%, signal=47%
KEGG_ARGININE_AND_PROLINE_METABOLISM	KEGG_ARGININE_AND_PROLINE_METABOLISM		51	-0.4232642	-1.5931112	0	0.07766917	1	2540	tags=20%, list=12%, signal=22%
SMIRNOV_CIRCULATING_ENDOTHELIOCYTES_IN_CANCER	SMIRNOV_CIRCULATING_ENDOTHELIOCYTES_IN_CANCER_UP		150	-0.3646616	-1.5928273	0	0.0776908	1	4771	tags=31%, list=22%, signal=40%
WEINMANN_ADAPTATION_TO_HYPOXIA_DN	WEINMANN_ADAPTATION_TO_HYPOXIA_DN		35	-0.5030525	-1.5924951	0.019608	0.07779598	1	3275	tags=31%, list=15%, signal=37%
GAUSSMANN_MLL_AF4_FUSION_TARGETS_E_UP	GAUSSMANN_MLL_AF4_FUSION_TARGETS_E_UP		73	-0.4164898	-1.5918231	0.017241	0.07794035	1	4240	tags=38%, list=19%, signal=47%
BOHN_PRIMARY_IMMUNODEFICIENCY_SYNDROM_UP	BOHN_PRIMARY_IMMUNODEFICIENCY_SYNDROM_UP		35	-0.4819099	-1.5916148	0.017544	0.0779163	1	5212	tags=46%, list=24%, signal=60%
DACOSTA_ERCC3_ALLELE_XPCS_VS_TTD_UP	DACOSTA_ERCC3_ALLELE_XPCS_VS_TTD_UP		22	-0.5200729	-1.5915277	0.033898	0.07772889	1	604	tags=23%, list=3%, signal=23%
REACTOME_TIGHT_JUNCTION_INTERACTIONS	REACTOME_TIGHT_JUNCTION_INTERACTIONS		30	-0.4839443	-1.5912955	0	0.07778814	1	2910	tags=33%, list=13%, signal=38%
OUILLETTE_CLL_13Q14_DELETION_DN	OUILLETTE_CLL_13Q14_DELETION_DN		57	-0.4121019	-1.5911494	0.017241	0.0776854	1	4381	tags=35%, list=20%, signal=44%
TONKS_TARGETS_OF_RUNX1_RUNX1T1_FUSION_ERYTHRO	TONKS_TARGETS_OF_RUNX1_RUNX1T1_FUSION_ERYTHROCYTE		15	-0.5843275	-1.5903813	0.019231	0.07790797	1	3968	tags=33%, list=18%, signal=41%
WU_HBX_TARGETS_1_DN	WU_HBX_TARGETS_1_DN		21	-0.5754934	-1.5899271	0.036364	0.0778469	1	5838	tags=48%, list=27%, signal=65%
MATTIOLI_MGUS_VS_PCL	MATTIOLI_MGUS_VS_PCL		98	-0.4008104	-1.5879681	0.018868	0.07859853	1	5595	tags=27%, list=26%, signal=35%
REACTOME_G2_M_CHECKPOINTS	REACTOME_G2_M_CHECKPOINTS		41	-0.4561769	-1.5873755	0	0.07881689	1	6002	tags=41%, list=27%, signal=57%
WOOD_EBV_EBNA1_TARGETS_UP	WOOD_EBV_EBNA1_TARGETS_UP		102	-0.3961461	-1.5873038	0	0.07859106	1	7408	tags=52%, list=34%, signal=78%
REACTOME_MITOTIC_M_M_G1_PHASES	REACTOME_MITOTIC_M_M_G1_PHASES		154	-0.3722184	-1.5872949	0	0.0783665	1	3849	tags=28%, list=18%, signal=34%
MARCHINI_TRABECTEDIN_RESISTANCE_DN	MARCHINI_TRABECTEDIN_RESISTANCE_DN		44	-0.4660974	-1.5857319	0.018868	0.07914443	1	3905	tags=39%, list=18%, signal=47%
JAATINEN_HEMATOPOIETIC_STEM_CELL_DN	JAATINEN_HEMATOPOIETIC_STEM_CELL_DN		204	-0.3624817	-1.5854342	0	0.07907949	1	2562	tags=17%, list=12%, signal=19%
DANG_MYC_TARGETS_DN	DANG_MYC_TARGETS_DN		31	-0.4888082	-1.5839182	0.021277	0.07973128	1	7447	tags=58%, list=34%, signal=88%
SATO_SILENCED_BY_METHYLATION_IN_PANCREATIC_CANC	SATO_SILENCED_BY_METHYLATION_IN_PANCREATIC_CANCER_1		374	-0.340312	-1.5835623	0	0.07962693	1	5472	tags=35%, list=25%, signal=46%
WINNEPENNINCKX_MELANOMA_METASTASIS_DN	WINNEPENNINCKX_MELANOMA_METASTASIS_DN		39	-0.4688937	-1.5824893	0.016129	0.08011186	1	3059	tags=33%, list=14%, signal=39%
REACTOME_PURINE_METABOLISM	REACTOME_PURINE_METABOLISM		29	-0.5045965	-1.5820702	0	0.08008385	1	837	tags=17%, list=4%, signal=18%
MOREIRA_RESPONSE_TO_TSA_UP	MOREIRA_RESPONSE_TO_TSA_UP		26	-0.4886947	-1.5818702	0.018868	0.07985954	1	2243	tags=27%, list=10%, signal=30%
WONG_EMBRYONIC_STEM_CELL_CORE	WONG_EMBRYONIC_STEM_CELL_CORE		325	-0.3419875	-1.5805916	0	0.08061645	1	5702	tags=34%, list=26%, signal=45%
SHAFFER_IRF4_MULTIPLE_MYELOMA_PROGRAM	SHAFFER_IRF4_MULTIPLE_MYELOMA_PROGRAM		36	-0.4725533	-1.5795443	0.016129	0.08128545	1	3597	tags=28%, list=16%, signal=33%
REACTOME_APOPTOTIC_EXECUTION_PHASE	REACTOME_APOPTOTIC_EXECUTION_PHASE		46	-0.4713647	-1.5788217	0.015873	0.08156669	1	5372	tags=35%, list=24%, signal=46%
AMIT_EGF_RESPONSE_120_MCF10A	AMIT_EGF_RESPONSE_120_MCF10A		40	-0.4597257	-1.5785347	0.017857	0.08157545	1	2506	tags=25%, list=11%, signal=28%
BEIER_GLIOMA_STEM_CELL_UP	BEIER_GLIOMA_STEM_CELL_UP		34	-0.4666204	-1.5774888	0.051724	0.08177548	1	1087	tags=18%, list=5%, signal=19%
COULOUARN_TEMPORAL_TGFB1_SIGNATURE_DN	COULOUARN_TEMPORAL_TGFB1_SIGNATURE_DN		115	-0.3773117	-1.5771745	0.02	0.08170585	1	4817	tags=37%, list=22%, signal=47%
BARRIER_CANCER_RELAPSE_TUMOR_SAMPLE_UP	BARRIER_CANCER_RELAPSE_TUMOR_SAMPLE_UP		15	-0.5797829	-1.576734	0.021739	0.08175623	1	1323	tags=20%, list=6%, signal=21%
DAZARD_UV_RESPONSE_CLUSTER_G24	DAZARD_UV_RESPONSE_CLUSTER_G24		16	-0.5498956	-1.5762851	0.030769	0.08187819	1	5831	tags=50%, list=27%, signal=68%
UDAYAKUMAR_MED1_TARGETS_DN	UDAYAKUMAR_MED1_TARGETS_DN		230	-0.3484972	-1.5736097	0	0.08334159	1	5262	tags=31%, list=24%, signal=40%
KEGG_DNA_REPLICATION	KEGG_DNA_REPLICATION		35	-0.4669795	-1.5733236	0.04	0.0832291	1	4086	tags=40%, list=19%, signal=49%
TARTE_PLASMA_CELL_VS_PLASMABLAST_UP	TARTE_PLASMA_CELL_VS_PLASMABLAST_UP		347	-0.3325334	-1.5718542	0	0.08376893	1	3603	tags=23%, list=16%, signal=27%
REACTOME_CELL_CYCLE_CHECKPOINTS	REACTOME_CELL_CYCLE_CHECKPOINTS		108	-0.3782568	-1.5696844	0	0.08540446	1	6002	tags=36%, list=27%, signal=49%
GARY_CD5_TARGETS_UP	GARY_CD5_TARGETS_UP		450	-0.3300604	-1.5691134	0	0.08555543	1	4980	tags=30%, list=23%, signal=39%
WATTEL_AUTONOMOUS_THYROID_ADENOMA_UP	WATTEL_AUTONOMOUS_THYROID_ADENOMA_UP		21	-0.551676	-1.5690652	0.019608	0.08536343	1	2931	tags=33%, list=13%, signal=38%
MISSIAGLIA_REGULATED_BY_METHYLATION_DN	MISSIAGLIA_REGULATED_BY_METHYLATION_DN		95	-0.386059	-1.567611	0	0.08615124	1	6400	tags=44%, list=29%, signal=62%
REACTOME_ACTIVATION_OF_ATR_IN_RESPONSE_TO_REPL	REACTOME_ACTIVATION_OF_ATR_IN_RESPONSE_TO_REPLICATI		36	-0.4544988	-1.567406	0.018182	0.08607348	1	7933	tags=58%, list=36%, signal=91%
BIOCARTA_IL10_PATHWAY	BIOCARTA_IL10_PATHWAY		17	-0.5525343	-1.5660036	0.035714	0.08678635	1	3255	tags=35%, list=15%, signal=41%
ABRAHAM_ALPC_VS_MULTIPLE_MYELOMA_UP	ABRAHAM_ALPC_VS_MULTIPLE_MYELOMA_UP		25	-0.5133309	-1.565986	0.0625	0.08666804	1	1179	tags=16%, list=5%, signal=17%
RUIZ_TNC_TARGETS_DN	RUIZ_TNC_TARGETS_DN		138	-0.3690686	-1.5653154	0	0.08669949	1	5631	tags=41%, list=26%, signal=54%
ROZANOV_MMP14_TARGETS_UP	ROZANOV_MMP14_TARGETS_UP		243	-0.3436785	-1.562653	0	0.08784337	1	4177	tags=26%, list=19%, signal=31%
BENPORATH_PROLIFERATION	BENPORATH_PROLIFERATION		142	-0.3851209	-1.562349	0	0.08772226	1	4246	tags=26%, list=19%, signal=32%
REACTOME_S_PHASE	REACTOME_S_PHASE		101	-0.3876984	-1.5622821	0	0.0874908	1	6834	tags=46%, list=31%, signal=66%
VERHAAK_AML_WITH_NPM1_MUTATED_DN	VERHAAK_AML_WITH_NPM1_MUTATED_DN		234	-0.3567287	-1.5617316	0	0.08748231	1	3448	tags=23%, list=16%, signal=27%
NUNODA_RESPONSE_TO_DASATINIB_IMATINIB_UP	NUNODA_RESPONSE_TO_DASATINIB_IMATINIB_UP		28	-0.4965439	-1.5617094	0.052632	0.0872527	1	6780	tags=50%, list=31%, signal=72%

WAMUNYOKOLI_OVARIAN_CANCER_GRADES_1_2_UP	WAMUNYOKOLI_OVARIAN_CANCER_GRADES_1_2_UP	128	-0.374114	-1.5617093	0	0.08702429	1	2350	tags=19%, list=11%, signal=21%	
MARSON_FOXP3_TARGETS_UP	MARSON_FOXP3_TARGETS_UP	57	-0.4285118	-1.5588076	0	0.08855365	1	4329	tags=35%, list=20%, signal=44%	
CHIARADONNA_NEOPLASTIC_TRANSFORMATION_KRAS_CD	CHIARADONNA_NEOPLASTIC_TRANSFORMATION_KRAS_CDC25	50	-0.4396248	-1.5586846	0.016393	0.08836024	1	3381	tags=28%, list=15%, signal=33%	
SHEDDEN_LUNG_CANCER_POOR_SURVIVAL_A6	SHEDDEN_LUNG_CANCER_POOR_SURVIVAL_A6	437	-0.3262212	-1.556329	0	0.08980976	1	4906	tags=29%, list=22%, signal=36%	
MORI_SMALL_PRE_BII_LYMPHOCYTE_DN	MORI_SMALL_PRE_BII_LYMPHOCYTE_DN	64	-0.4004	-1.5551661	0.017241	0.09034479	1	4163	tags=23%, list=19%, signal=29%	
KEGG_STEROID_BIOSYNTHESIS	KEGG_STEROID_BIOSYNTHESIS	17	-0.5470167	-1.5531638	0.020833	0.09163466	1	5312	tags=65%, list=24%, signal=85%	
MANALO_HYPOXIA_DN	MANALO_HYPOXIA_DN	281	-0.3336015	-1.5529068	0	0.09147045	1	4500	tags=27%, list=21%, signal=33%	
NUTT_GBM_VS_AO_GLIOMA_UP	NUTT_GBM_VS_AO_GLIOMA_UP	44	-0.4377721	-1.552298	0	0.09152607	1	6761	tags=52%, list=31%, signal=75%	
REACTOME_APOPTOSIS	REACTOME_APOPTOSIS	125	-0.3828697	-1.551171	0	0.09183221	1	5430	tags=30%, list=25%, signal=40%	
YAO_TEMPORAL_RESPONSE_TO_PROGESTERONE_CLUSTER	YAO_TEMPORAL_RESPONSE_TO_PROGESTERONE_CLUSTER_14	131	-0.3613466	-1.5511265	0.015385	0.09159734	1	7109	tags=40%, list=32%, signal=60%	
VALK_AML_WITH_EVI1	VALK_AML_WITH_EVI1	22	-0.5572723	-1.5500126	0.040816	0.09215016	1	4034	tags=41%, list=18%, signal=50%	
PAL_PRMT5_TARGETS_UP	PAL_PRMT5_TARGETS_UP	184	-0.3496427	-1.5497977	0	0.09202344	1	3905	tags=27%, list=18%, signal=32%	
SMID_BREAST_CANCER_RELAPSE_IN_BRAIN_UP	SMID_BREAST_CANCER_RELAPSE_IN_BRAIN_UP	39	-0.4652824	-1.5497954	0.039216	0.09178988	1	3956	tags=21%, list=18%, signal=25%	
YAO_TEMPORAL_RESPONSE_TO_PROGESTERONE_CLUSTER	YAO_TEMPORAL_RESPONSE_TO_PROGESTERONE_CLUSTER_16	76	-0.4082801	-1.5494794	0.017544	0.09187879	1	4184	tags=34%, list=19%, signal=42%	
MOOTHA_GLYCOGEN_METABOLISM	MOOTHA_GLYCOGEN_METABOLISM	20	-0.5132639	-1.5482247	0.035714	0.09268004	1	2186	tags=20%, list=10%, signal=22%	
KANG_FLUOROURACIL_RESISTANCE_DN	KANG_FLUOROURACIL_RESISTANCE_DN	15	-0.5523281	-1.5475974	0.039216	0.09297891	1	5592	tags=60%, list=26%, signal=80%	
YAO_TEMPORAL_RESPONSE_TO_PROGESTERONE_CLUSTER	YAO_TEMPORAL_RESPONSE_TO_PROGESTERONE_CLUSTER_7	66	-0.4128651	-1.5469393	0	0.09320276	1	4179	tags=27%, list=19%, signal=34%	
REACTOME_METABLISM_OF_NUCLEOTIDES	REACTOME_METABLISM_OF_NUCLEOTIDES	70	-0.4178277	-1.5465057	0.018868	0.09335841	1	3844	tags=30%, list=18%, signal=36%	
AIYAR_COBRA1_TARGETS_DN	AIYAR_COBRA1_TARGETS_DN	27	-0.4826549	-1.546339	0.037037	0.09322996	1	2421	tags=26%, list=11%, signal=29%	
LOCKWOOD_AMPLIFIED_IN_LUNG_CANCER	LOCKWOOD_AMPLIFIED_IN_LUNG_CANCER	169	-0.3502605	-1.5457544	0	0.09334656	1	6102	tags=37%, list=28%, signal=51%	
RIZKI_TUMOR_INVASIVENESS_2D_DN	RIZKI_TUMOR_INVASIVENESS_2D_DN	56	-0.4333549	-1.545213	0	0.09335773	1	3445	tags=27%, list=16%, signal=32%	
AMIT_EGF_RESPONSE_60_MCF10A	AMIT_EGF_RESPONSE_60_MCF10A	38	-0.4771335	-1.5434299	0.017544	0.09470093	1	3955	tags=37%, list=18%, signal=45%	
AMIT_EGF_RESPONSE_120_HELA	AMIT_EGF_RESPONSE_120_HELA	61	-0.397487	-1.5433939	0	0.09450143	1	2909	tags=25%, list=13%, signal=28%	
CHEN_LUNG_CANCER_SURVIVAL	CHEN_LUNG_CANCER_SURVIVAL	21	-0.5363538	-1.5394788	0.056604	0.09745649	1	4391	tags=33%, list=20%, signal=42%	
CHIARADONNA_NEOPLASTIC_TRANSFORMATION_KRAS_UP	CHIARADONNA_NEOPLASTIC_TRANSFORMATION_KRAS_UP	117	-0.3787637	-1.5393889	0.015152	0.09721645	1	5146	tags=32%, list=23%, signal=42%	
IZADPANAH_STEM_CELL_ADIPOSE_VS_BONE_UP	IZADPANAH_STEM_CELL_ADIPOSE_VS_BONE_UP	120	-0.3692805	-1.5388066	0	0.09738818	1	3880	tags=27%, list=18%, signal=32%	
SCIBETTA_KDM5B_TARGETS_DN	SCIBETTA_KDM5B_TARGETS_DN	73	-0.3794549	-1.5377189	0	0.09784193	1	4935	tags=32%, list=23%, signal=41%	
KEGG_HYPERTROPHIC_CARDIOMYOPATHY_HCM	KEGG_HYPERTROPHIC_CARDIOMYOPATHY_HCM	82	-0.3833072	-1.5366675	0.036364	0.09821957	1	2123	tags=22%, list=10%, signal=24%	
AMIT_EGF_RESPONSE_480_MCF10A	AMIT_EGF_RESPONSE_480_MCF10A	43	-0.4573049	-1.5366521	0.060606	0.09798002	1	4358	tags=37%, list=20%, signal=46%	
CHIARADONNA_NEOPLASTIC_TRANSFORMATION_KRAS_CD	CHIARADONNA_NEOPLASTIC_TRANSFORMATION_KRAS_CDC25	49	-0.4167625	-1.5356262	0.036364	0.09835504	1	5886	tags=51%, list=27%, signal=70%	
CHIN_BREAST_CANCER_COPY_NUMBER_UP	CHIN_BREAST_CANCER_COPY_NUMBER_UP	20	-0.5174229	-1.5350705	0.1	0.09832071	1	6816	tags=45%, list=31%, signal=65%	
SCHAVOLT_TARGETS_OF_TP53_AND_TP63	SCHAVOLT_TARGETS_OF_TP53_AND_TP63	15	-0.6037586	-1.5338683	0.083333	0.09900016	1	2165	tags=40%, list=10%, signal=44%	
REACTOME_SIGNALING_IN_IMMUNE_SYSTEM	REACTOME_SIGNALING_IN_IMMUNE_SYSTEM	299	-0.3281424	-1.532638	0	0.09980879	1	4961	tags=32%, list=23%, signal=41%	
YOSHIOKA_LIVER_CANCER_EARLY_RECURRENCE_UP	YOSHIOKA_LIVER_CANCER_EARLY_RECURRENCE_UP	33	-0.4559686	-1.5319604	0.037037	0.10000636	1	668	tags=12%, list=3%, signal=12%	
SASAKI_ADULT_T_CELL_LEUKEMIA	SASAKI_ADULT_T_CELL_LEUKEMIA	131	-0.3660121	-1.5315294	0.019231	0.10010807	1	4307	tags=31%, list=20%, signal=39%	
CHANG_IMMORTALIZED_BY_HPV31_DN	CHANG_IMMORTALIZED_BY_HPV31_DN	44	-0.4477967	-1.5309	0.017241	0.10040799	1	3448	tags=34%, list=16%, signal=40%	
FLECHNER_BIOPSY_KIDNEY_TRANSPLANT_OK_VS_DONOR	FLECHNER_BIOPSY_KIDNEY_TRANSPLANT_OK_VS_DONOR_DN	22	-0.5164414	-1.5308014	0.018868	0.10020196	1	3997	tags=41%, list=18%, signal=50%	
ACEVEDO_NORMAL_TISSUE_ADJACENT_TO_LIVER_TUMOR	ACEVEDO_NORMAL_TISSUE_ADJACENT_TO_LIVER_TUMOR_DN	337	-0.333477	-1.5297548	0	0.10063306	1	6284	tags=38%, list=29%, signal=52%	
PROVENZANI_METASTASIS_DN	PROVENZANI_METASTASIS_DN	132	-0.3634068	-1.5297245	0	0.10039345	1	5008	tags=28%, list=23%, signal=36%	
WANG_METHYLATED_IN_BREAST_CANCER	WANG_METHYLATED_IN_BREAST_CANCER	29	-0.5164414	-1.5286307	0.015385	0.10072231	1	3041	tags=34%, list=14%, signal=40%	
ALCALAY_AML_BY_NPM1_LOCALIZATION_UP	ALCALAY_AML_BY_NPM1_LOCALIZATION_UP	135	-0.375471	-1.5274111	0.016667	0.10131689	1	4143	tags=30%, list=19%, signal=37%	
NAKAMURA_TUMOR_ZONE_PERIPHERAL_VS_CENTRAL_UP	NAKAMURA_TUMOR_ZONE_PERIPHERAL_VS_CENTRAL_UP	266	-0.3316197	-1.5273573	0	0.10114384	1	5078	tags=31%, list=23%, signal=40%	
DAZARD_RESPONSE_TO_UV_NHEK_UP	DAZARD_RESPONSE_TO_UV_NHEK_UP	150	-0.356271	-1.5261166	0	0.10166837	1	4276	tags=27%, list=19%, signal=34%	
NAKAYAMA_SOFT_TISSUE_TUMORS_PCA1_DN	NAKAYAMA_SOFT_TISSUE_TUMORS_PCA1_DN	73	-0.4087043	-1.5250595	0.016667	0.10231995	1	3779	tags=29%, list=17%, signal=35%	
SESTO_RESPONSE_TO_UV_C6	SESTO_RESPONSE_TO_UV_C6	38	-0.4696638	-1.5249813	0	0.10221083	1	5452	tags=45%, list=25%, signal=59%	
KEGG_ASTHMA	KEGG_ASTHMA	23	-0.4850909	-1.5238483	0.056604	0.10285083	1	2673	tags=26%, list=12%, signal=30%	
SESTO_RESPONSE_TO_UV_C1	SESTO_RESPONSE_TO_UV_C1	70	-0.3928773	-1.5238193	0.051724	0.10261052	1	2610	tags=17%, list=12%, signal=19%	
JAZAERI_BREAST_CANCER_BRCA1_VS_BRCA2_DN	JAZAERI_BREAST_CANCER_BRCA1_VS_BRCA2_DN	38	-0.458159	-1.5221398	0.058824	0.10358233	1	224	tags=8%, list=1%, signal=8%	
KEGG_PATHWAYS_IN_CANCER	KEGG_PATHWAYS_IN_CANCER	321	-0.325152	-1.5217246	0	0.10356648	1	5162	tags=34%, list=24%, signal=43%	
ZHONG_RESPONSE_TO_AZACITIDINE_AND_TSA_UP	ZHONG_RESPONSE_TO_AZACITIDINE_AND_TSA_UP	165	-0.3533392	-1.5215138	0	0.1034551	1	5867	tags=36%, list=27%, signal=49%	
REACTOME_GLUCOSE_METABOLISM	REACTOME_GLUCOSE_METABOLISM	55	-0.4154988	-1.5206748	0	0.10409236	1	5452	tags=38%, list=25%, signal=51%	
ROME_INSULIN_TARGETS_IN_MUSCLE_UP	ROME_INSULIN_TARGETS_IN_MUSCLE_UP	86	-0.3787202	-1.518994	0.017857	0.10488927	1	4463	tags=28%, list=20%, signal=35%	
SENESE_HDAC2_TARGETS_DN	SENESE_HDAC2_TARGETS_DN	116	-0.3753396	-1.5177962	0	0.10554857	1	2899	tags=22%, list=13%, signal=26%	
BIOCARTA_41BB_PATHWAY	BIOCARTA_41BB_PATHWAY	17	-0.5258658	-1.5177778	0.016667	0.10533904	1	4171	tags=41%, list=19%, signal=51%	
WANG_BARRETTS_ESOPHAGUS_AND_ESOPHAGUS_CANCE	WANG_BARRETTS_ESOPHAGUS_AND_ESOPHAGUS_CANCER_DN	32	-0.470833	-1.5160466	0.046154	0.10638918	1	4019	tags=41%, list=18%, signal=50%	
BIOCARTA_DEATH_PATHWAY	BIOCARTA_DEATH_PATHWAY	30	-0.4709399	-1.5154037	0.033898	0.10672447	1	7118	tags=50%, list=32%, signal=74%	
CHANG_CORE_SERUM_RESPONSE_DN	CHANG_CORE_SERUM_RESPONSE_DN	28	-0.4483876	-1.515207	0.057692	0.10674028	1	4604	tags=36%, list=21%, signal=45%	
HOFFMANN_SMALL_PRE_BII_TO_IMMATURE_B_LYMPHOCY	HOFFMANN_SMALL_PRE_BII_TO_IMMATURE_B_LYMPHOCYTE_DN	22	-0.4910511	-1.5151365	0.055556	0.1065293	1	3001	tags=23%, list=14%, signal=26%	
LI_WILMS_TUMOR_VS_FETAL_KIDNEY_1_DN	LI_WILMS_TUMOR_VS_FETAL_KIDNEY_1_DN	159	-0.3457656	-1.5125207	0	0.10859311	1	5595	tags=31%, list=26%, signal=42%	
WEST_ADRENOCORTICAL_TUMOR_UP	WEST_ADRENOCORTICAL_TUMOR_UP	290	-0.3237195	-1.5121605	0	0.10859951	1	6149	tags=35%, list=28%, signal=48%	
KEGG_HEMATOPOIETIC_CELL_LINEAGE	KEGG_HEMATOPOIETIC_CELL_LINEAGE	78	-0.3995328	-1.5101128	0	0.10996915	1	3563	tags=33%, list=16%, signal=40%	
DANG_REGULATED_BY_MYC_DN	DANG_REGULATED_BY_MYC_DN	235	-0.3342463	-1.509613	0	0.11016389	1	5445	tags=34%, list=25%, signal=45%	
GESERICK_TERT_TARGETS_DN	GESERICK_TERT_TARGETS_DN	20	-0.5365907	-1.509236	0.070175	0.11007505	1	6742	tags=60%, list=31%, signal=87%	
HADDAD_T_LYMPHOCYTE_AND_NK_PROGENITOR_DN	HADDAD_T_LYMPHOCYTE_AND_NK_PROGENITOR_DN	59	-0.4045037	-1.5092243	0.05	0.10982769	1	3860	tags=29%, list=18%, signal=35%	

REACTOME_MITOTIC_PROMETAPHASE	REACTOME_MITOTIC_PROMETAPHASE		89	-0.3847508	-1.5082759	0.016667	0.11046141	1	3550	tags=28%, list=16%, signal=33%
GAJATE_RESPONSE_TO_TRABECTEDIN_DN	GAJATE_RESPONSE_TO_TRABECTEDIN_DN		15	-0.5582714	-1.5077385	0.037736	0.11068795	1	6085	tags=60%, list=28%, signal=83%
ROZANOV_MMP14_TARGETS_SUBSET	ROZANOV_MMP14_TARGETS_SUBSET		32	-0.4720938	-1.5056782	0.033333	0.11219478	1	4971	tags=38%, list=23%, signal=48%
MOOTHA_VOXPHOS	MOOTHA_VOXPHOS		83	-0.3809587	-1.505513	0.015152	0.11200824	1	6220	tags=43%, list=28%, signal=60%
GRADE_COLON_VS_RECTAL_CANCER_UP	GRADE_COLON_VS_RECTAL_CANCER_UP		28	-0.4674906	-1.5046705	0.036364	0.11225889	1	5418	tags=46%, list=25%, signal=62%
REACTOME_METABOLISM_OF_AMINO_ACIDS	REACTOME_METABOLISM_OF_AMINO_ACIDS		159	-0.3555568	-1.5041071	0	0.1124422	1	3838	tags=25%, list=18%, signal=30%
REACTOME_DNA_STRAND_ELONGATION	REACTOME_DNA_STRAND_ELONGATION		30	-0.4676982	-1.5034435	0.035714	0.11259467	1	4086	tags=37%, list=19%, signal=45%
ROSS_AML_WITH_PML_RARA_FUSION	ROSS_AML_WITH_PML_RARA_FUSION		76	-0.3886096	-1.502938	0.067797	0.11290411	1	5554	tags=33%, list=25%, signal=44%
JI_RESPONSE_TO_FSH_UP	JI_RESPONSE_TO_FSH_UP		52	-0.437037	-1.5023645	0.017857	0.11333466	1	5218	tags=38%, list=24%, signal=50%
RUTELLA_RESPONSE_TO_HGF_UP	RUTELLA_RESPONSE_TO_HGF_UP		394	-0.3194781	-1.5017395	0	0.11385965	1	4838	tags=27%, list=22%, signal=34%
LIU_CMYB_TARGETS_UP	LIU_CMYB_TARGETS_UP		154	-0.3444674	-1.5013161	0	0.11391695	1	2775	tags=18%, list=13%, signal=20%
THUM_MIR21_TARGETS_HEART_DISEASE_UP	THUM_MIR21_TARGETS_HEART_DISEASE_UP		18	-0.5321441	-1.5010825	0.040816	0.11385135	1	3198	tags=50%, list=15%, signal=58%
DAIRKEE_TERT_TARGETS_UP	DAIRKEE_TERT_TARGETS_UP		308	-0.3238503	-1.5010419	0	0.11363266	1	5005	tags=31%, list=23%, signal=39%
BEGUM_TARGETS_OF_PAX3_FOXO1_FUSION_DN	BEGUM_TARGETS_OF_PAX3_FOXO1_FUSION_DN		36	-0.4589572	-1.5009227	0	0.11347716	1	4838	tags=39%, list=22%, signal=50%
TOOKER_GEMCITABINE_RESISTANCE_DN	TOOKER_GEMCITABINE_RESISTANCE_DN		121	-0.3470633	-1.4979639	0.017857	0.11545213	1	4201	tags=24%, list=19%, signal=29%
DAIRKEE_CANCER_PRONE_RESPONSE_BPA	DAIRKEE_CANCER_PRONE_RESPONSE_BPA		45	-0.4529868	-1.4979568	0.016949	0.11520168	1	4118	tags=29%, list=19%, signal=35%
SESTO_RESPONSE_TO_UV_C3	SESTO_RESPONSE_TO_UV_C3		18	-0.5610339	-1.4978157	0.037736	0.11510388	1	3282	tags=44%, list=15%, signal=52%
FERNANDEZ_BOUND_BY_MYC	FERNANDEZ_BOUND_BY_MYC		174	-0.3620812	-1.4977283	0.015873	0.1149478	1	4899	tags=30%, list=22%, signal=39%
REACTOME_SYNTHESIS_OF_DNA	REACTOME_SYNTHESIS_OF_DNA		88	-0.367378	-1.4969627	0.017241	0.11542266	1	6834	tags=43%, list=31%, signal=62%
KYNG_DNA_DAMAGE_BY_4NQO	KYNG_DNA_DAMAGE_BY_4NQO		17	-0.5377929	-1.4942925	0.072727	0.11765044	1	4293	tags=53%, list=20%, signal=66%
KEGG_LEUKOCYTE_TRANSENDOTHELIAL_MIGRATION	KEGG_LEUKOCYTE_TRANSENDOTHELIAL_MIGRATION		112	-0.3629177	-1.4940213	0	0.11772663	1	4836	tags=34%, list=22%, signal=43%
GRAHAM_CML_QUIESCENT_VS_NORMAL_DIVIDING_UP	GRAHAM_CML_QUIESCENT_VS_NORMAL_DIVIDING_UP		54	-0.4083866	-1.4910712	0	0.12017593	1	3515	tags=30%, list=16%, signal=35%
CAIRO_LIVER_DEVELOPMENT_DN	CAIRO_LIVER_DEVELOPMENT_DN		206	-0.3349532	-1.490522	0	0.12048805	1	4720	tags=27%, list=22%, signal=34%
TURASHVILI_BREAST_DUCTAL_CARCINOMA_VS_LOBULAR	TURASHVILI_BREAST_DUCTAL_CARCINOMA_VS_LOBULAR_NORM		60	-0.4204366	-1.490049	0.048387	0.12076891	1	4106	tags=28%, list=19%, signal=35%
GRUETZMANN_PANCREATIC_CANCER_UP	GRUETZMANN_PANCREATIC_CANCER_UP		333	-0.3285394	-1.4898659	0	0.12066086	1	6149	tags=37%, list=28%, signal=50%
BIOCARTA_IL1R_PATHWAY	BIOCARTA_IL1R_PATHWAY		32	-0.4346641	-1.4897075	0.050847	0.12058463	1	4177	tags=31%, list=19%, signal=39%
DEBIASI_APOPTOSIS_BY_REOVIRUS_INFECTION_DN	DEBIASI_APOPTOSIS_BY_REOVIRUS_INFECTION_DN		220	-0.3262126	-1.4893037	0	0.12065602	1	6535	tags=36%, list=30%, signal=51%
REACTOME_CHOLESTEROL_BIOSYNTHESIS	REACTOME_CHOLESTEROL_BIOSYNTHESIS		21	-0.5179735	-1.4877201	0.084746	0.12197606	1	5312	tags=52%, list=24%, signal=69%
LI_PROSTATE_CANCER_EPIGENETIC	LI_PROSTATE_CANCER_EPIGENETIC		28	-0.4739736	-1.4848944	0.06383	0.12438314	1	4016	tags=39%, list=18%, signal=48%
KEGG_OTHER_GLYCAN_DEGRADATION	KEGG_OTHER_GLYCAN_DEGRADATION		16	-0.5241641	-1.483661	0.052632	0.12553464	1	3604	tags=31%, list=16%, signal=37%
STEIN_ESRRA_TARGETS_RESPONSIVE_TO_ESTROGEN_DN	STEIN_ESRRA_TARGETS_RESPONSIVE_TO_ESTROGEN_DN		41	-0.445889	-1.4817784	0.032787	0.12713158	1	5595	tags=44%, list=26%, signal=59%
CHARAFE_BREAST_CANCER_LUMINAL_VS_MESENCHYMAL	CHARAFE_BREAST_CANCER_LUMINAL_VS_MESENCHYMAL_DN		440	-0.3048217	-1.4813406	0	0.12721902	1	4464	tags=25%, list=20%, signal=31%
GU_PDEF_TARGETS_DN	GU_PDEF_TARGETS_DN		28	-0.4731866	-1.4810145	0.041667	0.1274219	1	1080	tags=25%, list=5%, signal=26%
MURAKAMI_UV_RESPONSE_6HR_UP	MURAKAMI_UV_RESPONSE_6HR_UP		30	-0.4918119	-1.4808744	0.050847	0.12724341	1	4740	tags=37%, list=22%, signal=47%
HELLER_HDAC_TARGETS_UP	HELLER_HDAC_TARGETS_UP		246	-0.3271343	-1.4805849	0	0.12727004	1	5568	tags=37%, list=25%, signal=48%
RODRIGUES_NTN1_TARGETS_DN	RODRIGUES_NTN1_TARGETS_DN		152	-0.348899	-1.4797165	0.015385	0.12797041	1	3946	tags=27%, list=18%, signal=33%
REACTOME_GLUCOSE_AND_OTHER_SUGAR_SLC_TRANSPO	REACTOME_GLUCOSE_AND_OTHER_SUGAR_SLC_TRANSPORTER		81	-0.3876698	-1.4791255	0	0.12831722	1	4247	tags=28%, list=19%, signal=35%
WIKMAN_ASBESTOS_LUNG_CANCER_UP	WIKMAN_ASBESTOS_LUNG_CANCER_UP		16	-0.5261217	-1.4787436	0.060606	0.12851708	1	883	tags=19%, list=4%, signal=20%
WANG_CISPLATIN_RESPONSE_AND_XPC_UP	WANG_CISPLATIN_RESPONSE_AND_XPC_UP		127	-0.3469868	-1.4783398	0	0.12857479	1	4876	tags=29%, list=22%, signal=37%
MOOTHA_FFA_OXYDATION	MOOTHA_FFA_OXYDATION		22	-0.5231276	-1.4775252	0.075472	0.12908812	1	7010	tags=73%, list=32%, signal=107%
LINDGREN_BLADDER_CANCER_CLUSTER_3_UP	LINDGREN_BLADDER_CANCER_CLUSTER_3_UP		305	-0.3237431	-1.4765469	0	0.12983422	1	7010	tags=41%, list=32%, signal=59%
LANDIS_ERBB2_BREAST_PRENEOPLASTIC_UP	LANDIS_ERBB2_BREAST_PRENEOPLASTIC_UP		18	-0.5275514	-1.473855	0.06	0.13198872	1	2197	tags=33%, list=10%, signal=37%
AMIT_SERUM_RESPONSE_60_MCF10A	AMIT_SERUM_RESPONSE_60_MCF10A		56	-0.4193135	-1.4730667	0.017857	0.13237591	1	2713	tags=29%, list=12%, signal=33%
GARCIA_TARGETS_OF_FLI1_AND_DAX1_DN	GARCIA_TARGETS_OF_FLI1_AND_DAX1_DN		141	-0.353932	-1.4723809	0.017544	0.13261925	1	3982	tags=28%, list=18%, signal=34%
DACOSTA_UV_RESPONSE_VIA_ERCC3_TTD_UP	DACOSTA_UV_RESPONSE_VIA_ERCC3_TTD_UP		45	-0.4158411	-1.4720539	0.048387	0.13257658	1	4156	tags=29%, list=19%, signal=36%
GOZGIT_ESR1_TARGETS_UP	GOZGIT_ESR1_TARGETS_UP		134	-0.3534028	-1.4719806	0.018868	0.1323353	1	3443	tags=23%, list=16%, signal=27%
GAJATE_RESPONSE_TO_TRABECTEDIN_UP	GAJATE_RESPONSE_TO_TRABECTEDIN_UP		51	-0.4122468	-1.4711881	0.037736	0.13283883	1	3311	tags=27%, list=15%, signal=32%
STREICHER_LSM1_TARGETS_DN	STREICHER_LSM1_TARGETS_DN		17	-0.548041	-1.4711711	0.075472	0.13256937	1	1310	tags=35%, list=6%, signal=38%
URS_ADIPOCYTE_DIFFERENTIATION_DN	URS_ADIPOCYTE_DIFFERENTIATION_DN		29	-0.4643662	-1.4708478	0.05	0.1325557	1	4464	tags=45%, list=20%, signal=56%
KEGG_CARDIAC_MUSCLE_CONTRACTION	KEGG_CARDIAC_MUSCLE_CONTRACTION		71	-0.3879035	-1.4702002	0.030769	0.13296932	1	2235	tags=23%, list=10%, signal=25%
ZHAN_VARIABLE_EARLY_DIFFERENTIATION_GENES_DN	ZHAN_VARIABLE_EARLY_DIFFERENTIATION_GENES_DN		27	-0.4514224	-1.4693092	0.08	0.13349803	1	6767	tags=56%, list=31%, signal=80%
ST_P38_MAPK_PATHWAY	ST_P38_MAPK_PATHWAY		35	-0.4488824	-1.4691046	0.058824	0.1334265	1	2819	tags=29%, list=13%, signal=33%
REACTOME_INNATE_IMMUNITY_SIGNALING	REACTOME_INNATE_IMMUNITY_SIGNALING		101	-0.3738394	-1.4689009	0.030769	0.1333264	1	5102	tags=37%, list=23%, signal=48%
VART_KSHV_INFECTION_ANGIOGENIC_MARKERS_DN	VART_KSHV_INFECTION_ANGIOGENIC_MARKERS_DN		130	-0.3392535	-1.4686309	0	0.13333891	1	2517	tags=22%, list=11%, signal=24%
REACTOME_ACTIVATION_OF_THE_PRE_REPLICATIVE_COM	REACTOME_ACTIVATION_OF_THE_PRE_REPLICATIVE_COMPLEX		28	-0.4478555	-1.4683667	0.081967	0.1333245	1	5631	tags=46%, list=26%, signal=62%
YAMASHITA_LIVER_CANCER_STEM_CELL_UP	YAMASHITA_LIVER_CANCER_STEM_CELL_UP		46	-0.4260616	-1.4680867	0.066667	0.13322735	1	3811	tags=35%, list=17%, signal=42%
PARK_TRETINOIN_RESPONSE_AND_RARA_PLZF_FUSION	PARK_TRETINOIN_RESPONSE_AND_RARA_PLZF_FUSION		18	-0.5067962	-1.4678982	0.019608	0.13304596	1	2949	tags=33%, list=13%, signal=38%
DAIRKEE_CANCER_PRONE_RESPONSE_BPA_E2	DAIRKEE_CANCER_PRONE_RESPONSE_BPA_E2		111	-0.3508594	-1.4665269	0.016129	0.13378376	1	3020	tags=21%, list=14%, signal=24%
KRIGE_AMINO_ACID_DEPRIVATION	KRIGE_AMINO_ACID_DEPRIVATION		25	-0.4810366	-1.4658834	0.04	0.13407132	1	4740	tags=40%, list=22%, signal=51%
MARKEY_RB1_CHRONIC_LOF_UP	MARKEY_RB1_CHRONIC_LOF_UP		107	-0.3787844	-1.4655349	0.016393	0.13400099	1	4195	tags=30%, list=19%, signal=37%
SHAFFER_IRF4_TARGETS_IN_PLASMA_CELL_VS_MATURE_	SHAFFER_IRF4_TARGETS_IN_PLASMA_CELL_VS_MATURE_B_LYM		65	-0.3864888	-1.4643027	0.016393	0.13482001	1	4918	tags=29%, list=22%, signal=38%
CHIANG_LIVER_CANCER_SUBCLASS_CTNNB1_DN	CHIANG_LIVER_CANCER_SUBCLASS_CTNNB1_DN		136	-0.3552066	-1.4635476	0.016129	0.13522036	1	5850	tags=40%, list=27%, signal=54%
FERREIRA_EWINGS_SARCOMA_UNSTABLE_VS_STABLE_DN	FERREIRA_EWINGS_SARCOMA_UNSTABLE_VS_STABLE_DN		88	-0.3665028	-1.4629724	0.017857	0.13534111	1	4623	tags=31%, list=21%, signal=39%
PENG_LEUCINE_DEPRIVATION_DN	PENG_LEUCINE_DEPRIVATION_DN		49	-0.4044606	-1.4624308	0.017241	0.13570637	1	6788	tags=43%, list=31%, signal=62%

DAVIES_MULTIPLE_MYELOMA_VS_MGUS_DN	DAVIES_MULTIPLE_MYELOMA_VS_MGUS_DN		20	-0.5234745	-1.4619426	0.072727	0.13587922	1	1692	tags=15%, list=8%, signal=16%
TAKEDA_TARGETS_OF_NUP98_HOXA9_FUSION_10D_DN	TAKEDA_TARGETS_OF_NUP98_HOXA9_FUSION_10D_DN		123	-0.3698149	-1.4614164	0.015873	0.13613151	1	1813	tags=15%, list=8%, signal=16%
APPEL_IMATINIB_RESPONSE	APPEL_IMATINIB_RESPONSE		32	-0.431435	-1.4606686	0.086207	0.13655333	1	6250	tags=38%, list=29%, signal=52%
BROWNE_HCMV_INFECTION_16HR_DN	BROWNE_HCMV_INFECTION_16HR_DN		76	-0.3864907	-1.4601493	0.054545	0.13689218	1	3956	tags=34%, list=18%, signal=42%
UEDA_CENTRAL_CLOCK	UEDA_CENTRAL_CLOCK		85	-0.3800371	-1.4595122	0.033898	0.1373364	1	3357	tags=24%, list=15%, signal=28%
FARMER_BREAST_CANCER_BASAL_VS_LULMINAL	FARMER_BREAST_CANCER_BASAL_VS_LULMINAL		318	-0.3110166	-1.45929	0	0.13731597	1	4672	tags=25%, list=21%, signal=31%
OSADA_ASCL1_TARGETS_DN	OSADA_ASCL1_TARGETS_DN		24	-0.5013028	-1.4589479	0.061538	0.1375163	1	3367	tags=38%, list=15%, signal=44%
SMITH_TERT_TARGETS_DN	SMITH_TERT_TARGETS_DN		71	-0.3936962	-1.4577905	0.018868	0.13847822	1	4265	tags=28%, list=19%, signal=35%
CHANDRAN_METASTASIS_UP	CHANDRAN_METASTASIS_UP		79	-0.3495803	-1.4559584	0	0.13978213	1	4681	tags=27%, list=21%, signal=34%
REACTOME_EXTENSION_OF_TELOMERES	REACTOME_EXTENSION_OF_TELOMERES		28	-0.4713381	-1.4554948	0.038462	0.13997014	1	4161	tags=43%, list=19%, signal=53%
GENTILE_UV_LOW_DOSE_UP	GENTILE_UV_LOW_DOSE_UP		18	-0.5179235	-1.4549013	0.052632	0.14029455	1	5460	tags=61%, list=25%, signal=81%
LIEN_BREAST_CARCINOMA_METAPLASTIC_VS_DUCTAL_DN	LIEN_BREAST_CARCINOMA_METAPLASTIC_VS_DUCTAL_DN		93	-0.3847169	-1.4547647	0.028986	0.14010812	1	3406	tags=29%, list=16%, signal=34%
JIANG_HYPOXIA_NORMAL	JIANG_HYPOXIA_NORMAL		209	-0.3322066	-1.4534777	0.016667	0.14118108	1	5208	tags=30%, list=24%, signal=39%
FALVELLA_SMOKERS_WITH_LUNG_CANCER	FALVELLA_SMOKERS_WITH_LUNG_CANCER		65	-0.395029	-1.4526322	0.033898	0.14160615	1	3031	tags=22%, list=14%, signal=25%
WONG_ENDMETRIUM_CANCER_DN	WONG_ENDMETRIUM_CANCER_DN		42	-0.3903118	-1.4520241	0.035714	0.14187291	1	5367	tags=48%, list=24%, signal=63%
REACTOME_AMINO_ACID_AND_OLIGOPEPTIDE_SLC_TRAN	REACTOME_AMINO_ACID_AND_OLIGOPEPTIDE_SLC_TRANSPORT		48	-0.4159157	-1.4518716	0.018182	0.14178929	1	3856	tags=29%, list=18%, signal=35%
WINTER_HYPOXIA_METAGENE	WINTER_HYPOXIA_METAGENE		212	-0.3244471	-1.4517173	0	0.14181322	1	4414	tags=24%, list=20%, signal=29%
CHEMNITZ_RESPONSE_TO_PROSTAGLANDIN_E2_UP	CHEMNITZ_RESPONSE_TO_PROSTAGLANDIN_E2_UP		133	-0.343055	-1.4515859	0	0.14165169	1	5105	tags=34%, list=23%, signal=44%
SENESE_HDAC1_AND_HDAC2_TARGETS_DN	SENESE_HDAC1_AND_HDAC2_TARGETS_DN		212	-0.3271478	-1.4509363	0	0.14194232	1	4367	tags=28%, list=20%, signal=35%
WANG_ESOPHAGUS_CANCER_VS_NORMAL_DN	WANG_ESOPHAGUS_CANCER_VS_NORMAL_DN		79	-0.3613843	-1.4505322	0	0.14201668	1	5781	tags=35%, list=26%, signal=48%
ROSS_AML_WITH_AML1_ETO_FUSION	ROSS_AML_WITH_AML1_ETO_FUSION		71	-0.3900458	-1.4486488	0.044776	0.14389245	1	5122	tags=39%, list=23%, signal=51%
INGA_TP53_TARGETS	INGA_TP53_TARGETS		15	-0.5439805	-1.4476508	0.067797	0.14436378	1	1896	tags=40%, list=9%, signal=44%
VARELA_ZMPSTE24_TARGETS_DN	VARELA_ZMPSTE24_TARGETS_DN		39	-0.409859	-1.4472376	0.04918	0.14467192	1	2823	tags=28%, list=13%, signal=32%
MOREAUX_B_LYMPHOCYTE_MATURATION_BY_TACI_UP	MOREAUX_B_LYMPHOCYTE_MATURATION_BY_TACI_UP		69	-0.3767291	-1.447001	0	0.14458546	1	1409	tags=16%, list=6%, signal=17%
PODAR_RESPONSE_TO_ADAPHOSTIN_DN	PODAR_RESPONSE_TO_ADAPHOSTIN_DN		18	-0.531569	-1.4445047	0.076923	0.14694381	1	6193	tags=50%, list=28%, signal=70%
KAAB_FAILED_HEART_ATRIUM_UP	KAAB_FAILED_HEART_ATRIUM_UP		35	-0.4361596	-1.4442934	0.018519	0.14695506	1	3085	tags=31%, list=14%, signal=37%
DAZARD_UV_RESPONSE_CLUSTER_G2	DAZARD_UV_RESPONSE_CLUSTER_G2		20	-0.5147147	-1.4442027	0.022222	0.14683728	1	3692	tags=50%, list=17%, signal=60%
GAURNIER_PSMD4_TARGETS	GAURNIER_PSMD4_TARGETS		46	-0.4213065	-1.4433211	0.046875	0.14763376	1	4360	tags=35%, list=20%, signal=43%
KANG_CISPLATIN_RESISTANCE_UP	KANG_CISPLATIN_RESISTANCE_UP		17	-0.5336057	-1.4426526	0.137255	0.14806116	1	4740	tags=53%, list=22%, signal=67%
TOOKER_RESPONSE_TO_BEXAROTENE_UP	TOOKER_RESPONSE_TO_BEXAROTENE_UP		121	-0.3470633	-1.4420264	0.014493	0.14846516	1	4201	tags=24%, list=19%, signal=29%
LIANG_HEMATOPOIESIS_STEM_CELL_NUMBER_LARGE_VS	LIANG_HEMATOPOIESIS_STEM_CELL_NUMBER_LARGE_VS_TINY		37	-0.4264487	-1.4411062	0.066667	0.14906935	1	7043	tags=51%, list=32%, signal=76%
PARK_APL_PATHOGENESIS_DN	PARK_APL_PATHOGENESIS_DN		33	-0.434148	-1.4406766	0.09434	0.1494735	1	4332	tags=42%, list=20%, signal=53%
NAKAJIMA_MAST_CELL	NAKAJIMA_MAST_CELL		29	-0.4936975	-1.4405929	0.065574	0.1492498	1	4320	tags=38%, list=20%, signal=47%
WANG_PROSTATE_CANCER_ANDROGEN_INDEPENDENT	WANG_PROSTATE_CANCER_ANDROGEN_INDEPENDENT		61	-0.3712891	-1.4405648	0.016949	0.14900097	1	4784	tags=31%, list=22%, signal=40%
RIZKI_TUMOR_INVASIVENESS_3D_UP	RIZKI_TUMOR_INVASIVENESS_3D_UP		179	-0.324757	-1.4405596	0	0.14872706	1	4713	tags=28%, list=21%, signal=35%
REACTOME_GLYCOGEN_BREAKDOWN_GLYCOGENOLYSIS	REACTOME_GLYCOGEN_BREAKDOWN_GLYCOGENOLYSIS		15	-0.4949331	-1.4395468	0.075472	0.14933164	1	5452	tags=33%, list=25%, signal=44%
SENGUPTA_NASOPHARYNGEAL_CARCINOMA_UP	SENGUPTA_NASOPHARYNGEAL_CARCINOMA_UP		264	-0.3155429	-1.4380926	0	0.15057	1	4383	tags=29%, list=20%, signal=36%
GENTILE_UV_LOW_DOSE_DN	GENTILE_UV_LOW_DOSE_DN		18	-0.5179235	-1.4367943	0.078125	0.15157785	1	5460	tags=61%, list=25%, signal=81%
AGUIRRE_PANCREATIC_CANCER_COPY_NUMBER_UP	AGUIRRE_PANCREATIC_CANCER_COPY_NUMBER_UP		275	-0.3215245	-1.4355309	0	0.15260883	1	7150	tags=42%, list=33%, signal=62%
SATO_SILENCED_EPIGENETICALLY_IN_PANCREATIC_CANC	SATO_SILENCED_EPIGENETICALLY_IN_PANCREATIC_CANCER		37	-0.4228265	-1.4353235	0.032258	0.15269066	1	4396	tags=41%, list=20%, signal=51%
KEGG_PRION_DISEASES	KEGG_PRION_DISEASES		34	-0.4571754	-1.4351329	0.04	0.15256527	1	5171	tags=44%, list=24%, signal=58%
KUMAR_TARGETS_OF_MLL_AF9_FUSION	KUMAR_TARGETS_OF_MLL_AF9_FUSION		402	-0.2953076	-1.433972	0	0.15348426	1	4389	tags=26%, list=20%, signal=32%
REACTOME_COLLAGEN_MEDIATED_ACTIVATION_CASCADE	REACTOME_COLLAGEN_MEDIATED_ACTIVATION_CASCADE		22	-0.4978257	-1.4332606	0.052632	0.15391545	1	3466	tags=36%, list=16%, signal=43%
ONDER_CDH1_TARGETS_1_UP	ONDER_CDH1_TARGETS_1_UP		128	-0.3446939	-1.43312	0	0.15371391	1	2421	tags=17%, list=11%, signal=19%
HOFFMANN_PRE_BI_TO_LARGE_PRE_BII_LYMPHOCYTE_DN	HOFFMANN_PRE_BI_TO_LARGE_PRE_BII_LYMPHOCYTE_DN		56	-0.3738719	-1.4326282	0.116667	0.15404308	1	4317	tags=27%, list=20%, signal=33%
DORN_ADENOVIRUS_INFECTION_24HR_DN	DORN_ADENOVIRUS_INFECTION_24HR_DN		38	-0.4148741	-1.4323089	0.018519	0.15401591	1	5420	tags=32%, list=25%, signal=42%
SWEET_KRAS_TARGETS_UP	SWEET_KRAS_TARGETS_UP		19	-0.4947102	-1.4323072	0.075472	0.15373892	1	3976	tags=37%, list=18%, signal=45%
CROONQUIST_STROMAL_STIMULATION_UP	CROONQUIST_STROMAL_STIMULATION_UP		44	-0.4309432	-1.4311022	0.068966	0.15482599	1	5379	tags=52%, list=25%, signal=69%
REACTOME_CHEMOKINE_RECEPTORS_BIND_CHEMOKINES	REACTOME_CHEMOKINE_RECEPTORS_BIND_CHEMOKINES		45	-0.4310243	-1.4310766	0.1	0.15454851	1	992	tags=13%, list=5%, signal=14%
BOYLAN_MULTIPLE_MYELOMA_C_CLUSTER_UP	BOYLAN_MULTIPLE_MYELOMA_C_CLUSTER_UP		34	-0.4449159	-1.4287808	0.066667	0.1565589	1	3661	tags=32%, list=17%, signal=39%
BASSO_CD40_SIGNALING_UP	BASSO_CD40_SIGNALING_UP		95	-0.3634463	-1.4285399	0.018868	0.15652978	1	4780	tags=35%, list=22%, signal=44%
BROWNE_HCMV_INFECTION_8HR_UP	BROWNE_HCMV_INFECTION_8HR_UP		93	-0.3563663	-1.4284513	0.015873	0.15627563	1	3358	tags=27%, list=15%, signal=32%
SA_MMP_CYTOKINE_CONNECTION	SA_MMP_CYTOKINE_CONNECTION		15	-0.5370923	-1.4280967	0.071429	0.15637329	1	3964	tags=47%, list=18%, signal=57%
DAVICIONI_RHABDOMYOSARCOMA_PAX_FOXO1_FUSION_I	DAVICIONI_RHABDOMYOSARCOMA_PAX_FOXO1_FUSION_DN		15	-0.5414868	-1.4278538	0.08	0.15639399	1	2414	tags=33%, list=11%, signal=37%
NAKAMURA_CANCER_MICROENVIRONMENT_DN	NAKAMURA_CANCER_MICROENVIRONMENT_DN		45	-0.424378	-1.4273106	0.087719	0.15669237	1	6002	tags=36%, list=27%, signal=49%
MORI_IMMATURE_B_LYMPHOCYTE_UP	MORI_IMMATURE_B_LYMPHOCYTE_UP		41	-0.439076	-1.426777	0.054545	0.15706049	1	5468	tags=44%, list=25%, signal=58%
NAGASHIMA_EGF_SIGNALING_UP	NAGASHIMA_EGF_SIGNALING_UP		56	-0.3784985	-1.4264725	0.034483	0.15745212	1	3031	tags=23%, list=14%, signal=27%
ONDER_CDH1_TARGETS_1_DN	ONDER_CDH1_TARGETS_1_DN		159	-0.3303567	-1.4260186	0	0.15771922	1	3320	tags=19%, list=15%, signal=22%
HOFMANN_CELL_LYMPHOMA_DN	HOFMANN_CELL_LYMPHOMA_DN		32	-0.4464765	-1.4256203	0.035088	0.15800893	1	4725	tags=38%, list=22%, signal=48%
HUANG_DASATINIB_RESISTANCE_UP	HUANG_DASATINIB_RESISTANCE_UP		69	-0.3846774	-1.4253439	0.016129	0.15795131	1	4306	tags=29%, list=20%, signal=36%
MATSUDA_NATURAL_KILLER_DIFFERENTIATION	MATSUDA_NATURAL_KILLER_DIFFERENTIATION		462	-0.2958639	-1.4240996	0	0.15897308	1	4366	tags=23%, list=20%, signal=28%
YAO_TEMPORAL_RESPONSE_TO_PROGESTERONE_CLUSTEF	YAO_TEMPORAL_RESPONSE_TO_PROGESTERONE_CLUSTER_17		168	-0.3331754	-1.4227798	0.017544	0.16043243	1	6394	tags=38%, list=29%, signal=53%
KEGG_PENTOSE_PHOSPHATE_PATHWAY	KEGG_PENTOSE_PHOSPHATE_PATHWAY		26	-0.4685508	-1.4223459	0.058824	0.1604472	1	5159	tags=42%, list=24%, signal=55%
DORN_ADENOVIRUS_INFECTION_12HR_DN	DORN_ADENOVIRUS_INFECTION_12HR_DN		28	-0.4573375	-1.4220772	0.081967	0.16036154	1	4982	tags=36%, list=23%, signal=46%

ROSS_ACUTE_MYELOID_LEUKEMIA_CBF	ROSS_ACUTE_MYELOID_LEUKEMIA_CBF		77	-0.3774391	-1.4220282	0.032258	0.16010688	1	5068	tags=42%, list=23%, signal=54%
ZHANG_RESPONSE_TO_CANTHARIDIN_DN	ZHANG_RESPONSE_TO_CANTHARIDIN_DN		58	-0.4000071	-1.4213277	0.074074	0.16060534	1	4263	tags=29%, list=19%, signal=36%
KEGG_ECM_RECEPTOR_INTERACTION	KEGG_ECM_RECEPTOR_INTERACTION		81	-0.3668665	-1.4210699	0.052632	0.16054493	1	2672	tags=26%, list=12%, signal=29%
RADMACHER_AML_PROGNOSIS	RADMACHER_AML_PROGNOSIS		75	-0.3629891	-1.4209447	0	0.1604369	1	3670	tags=27%, list=17%, signal=32%
APPIERTO_RESPONSE_TO_FENRETINIDE_DN	APPIERTO_RESPONSE_TO_FENRETINIDE_DN		40	-0.4400888	-1.4188658	0.055556	0.16230193	1	4594	tags=30%, list=21%, signal=38%
BILD_SRC_ONCOGENIC_SIGNATURE	BILD_SRC_ONCOGENIC_SIGNATURE		56	-0.4037577	-1.4186633	0.016393	0.16216512	1	5725	tags=34%, list=26%, signal=46%
TANG_SENESCENCE_TP53_TARGETS_UP	TANG_SENESCENCE_TP53_TARGETS_UP		24	-0.4499044	-1.4168395	0.112903	0.16362861	1	2101	tags=25%, list=10%, signal=28%
WANG_HCP_PROSTATE_CANCER	WANG_HCP_PROSTATE_CANCER		73	-0.3788549	-1.4157606	0.030303	0.16462995	1	4283	tags=30%, list=20%, signal=37%
KEGG_FOCAL_ADHESION	KEGG_FOCAL_ADHESION		195	-0.3176714	-1.4153192	0	0.16480786	1	5426	tags=36%, list=25%, signal=47%
FARMER_BREAST_CANCER_APOCRINE VS LUMINAL	FARMER_BREAST_CANCER_APOCRINE VS LUMINAL		311	-0.2949263	-1.4146581	0	0.16541556	1	4418	tags=24%, list=20%, signal=29%
CAIRO_HEPATOBLASTOMA_CLASSES_DN	CAIRO_HEPATOBLASTOMA_CLASSES_DN		198	-0.3138323	-1.4143022	0	0.16556679	1	5253	tags=34%, list=24%, signal=45%
REACTOME_SLC_MEDIATED_TRANSMEMBRANE_TRANSPOR	REACTOME_SLC_MEDIATED_TRANSMEMBRANE_TRANSPORT		168	-0.339145	-1.4128438	0.034483	0.16691428	1	4247	tags=26%, list=19%, signal=32%
REACTOME_CELLEXTRACELLULAR_MATRIX_INTERACTIONS	REACTOME_CELLEXTRACELLULAR_MATRIX_INTERACTIONS		16	-0.4852765	-1.4120482	0.1	0.16761334	1	4922	tags=44%, list=22%, signal=56%
VALK_AML_CLUSTER_2	VALK_AML_CLUSTER_2		26	-0.4597279	-1.4109086	0.153846	0.1686686	1	4350	tags=46%, list=20%, signal=58%
GOLUB_ALL_VS_AML_DN	GOLUB_ALL_VS_AML_DN		16	-0.5171525	-1.4104224	0.086957	0.16895355	1	4413	tags=44%, list=20%, signal=55%
KOKKINAKIS_METHIONINE_DEPRIVATION_96HR_DN	KOKKINAKIS_METHIONINE_DEPRIVATION_96HR_DN		71	-0.374718	-1.4098295	0.031746	0.16940325	1	6036	tags=42%, list=28%, signal=58%
CAFFAREL_RESPONSE_TO_THC_DN	CAFFAREL_RESPONSE_TO_THC_DN		26	-0.4581295	-1.4093912	0.038462	0.1695668	1	2409	tags=31%, list=11%, signal=35%
TARTE_PLASMA_CELL_VS_PLASMABLAST_DN	TARTE_PLASMA_CELL_VS_PLASMABLAST_DN		299	-0.3078968	-1.4092907	0	0.16932671	1	5631	tags=30%, list=26%, signal=40%
POOLA_INVASIVE_BREAST_CANCER_DN	POOLA_INVASIVE_BREAST_CANCER_DN		132	-0.326807	-1.4092566	0.032258	0.16906434	1	3626	tags=23%, list=17%, signal=27%
RUTELLA_RESPONSE_TO_HGF_VS_CSF2RB_AND_IL4_UP	RUTELLA_RESPONSE_TO_HGF_VS_CSF2RB_AND_IL4_UP		380	-0.2943772	-1.4081042	0	0.16979885	1	4838	tags=26%, list=22%, signal=33%
BERENJENO_TRANSFORMED_BY_RHOA_FOREVER_UP	BERENJENO_TRANSFORMED_BY_RHOA_FOREVER_UP		18	-0.5380738	-1.4075344	0.111111	0.1700812	1	2974	tags=33%, list=14%, signal=39%
AMUNDSON_POOR_SURVIVAL_AFTER_GAMMA_RADIATION	AMUNDSON_POOR_SURVIVAL_AFTER_GAMMA_RADIATION_2G		150	-0.3343576	-1.4073972	0	0.1698427	1	6592	tags=39%, list=30%, signal=55%
KIM_GASTRIC_CANCER_CHEMOSENSITIVITY	KIM_GASTRIC_CANCER_CHEMOSENSITIVITY		93	-0.356004	-1.4073089	0.014493	0.16972296	1	3446	tags=25%, list=16%, signal=29%
REACTOME_CELL_CYCLE_MITOTIC	REACTOME_CELL_CYCLE_MITOTIC		297	-0.2966337	-1.4070494	0	0.16965042	1	3849	tags=22%, list=18%, signal=27%
PODAR_RESPONSE_TO_ADAPHOSTIN_UP	PODAR_RESPONSE_TO_ADAPHOSTIN_UP		130	-0.3417046	-1.4063913	0	0.17002364	1	4413	tags=26%, list=20%, signal=33%
POTTI_CYTOXAN_SENSITIVITY	POTTI_CYTOXAN_SENSITIVITY		32	-0.4240803	-1.4053663	0.033333	0.17131227	1	3660	tags=28%, list=17%, signal=34%
PEREZ_TP63_TARGETS	PEREZ_TP63_TARGETS		318	-0.3022031	-1.4041657	0	0.1721255	1	4437	tags=29%, list=20%, signal=35%
SCHUHMACHER_MYC_TARGETS_UP	SCHUHMACHER_MYC_TARGETS_UP		67	-0.379654	-1.4040738	0.034483	0.17193228	1	5087	tags=30%, list=23%, signal=39%
KEGG_TYPE_II_DIABETES_MELLITUS	KEGG_TYPE_II_DIABETES_MELLITUS		47	-0.3970343	-1.4004903	0.032258	0.17626159	1	2047	tags=15%, list=9%, signal=16%
MORI_PLASMA_CELL_UP	MORI_PLASMA_CELL_UP		39	-0.4223946	-1.3999023	0.055556	0.17678419	1	8109	tags=62%, list=37%, signal=97%
KORKOLA_EMBRYONIC_CARCINOMA_VS_SEMINOMA_DN	KORKOLA_EMBRYONIC_CARCINOMA_VS_SEMINOMA_DN		24	-0.4472166	-1.399838	0.089286	0.17653842	1	1857	tags=21%, list=8%, signal=23%
RUGO_RESPONSE_TO_4NQO	RUGO_RESPONSE_TO_4NQO		17	-0.5377929	-1.3996527	0.065574	0.17650126	1	4293	tags=53%, list=20%, signal=66%
HOSHIDA_LIVER_CANCER_SURVIVAL_UP	HOSHIDA_LIVER_CANCER_SURVIVAL_UP		72	-0.3731946	-1.39665	0.064516	0.1796399	1	3838	tags=31%, list=18%, signal=37%
KAN_RESPONSE_TO_ARSENIC_TRIOXIDE	KAN_RESPONSE_TO_ARSENIC_TRIOXIDE		108	-0.3232354	-1.39532	0.033898	0.18126738	1	4329	tags=24%, list=20%, signal=30%
KINSEY_TARGETS_OF_EWSR1_FLII_FUSION_DN	KINSEY_TARGETS_OF_EWSR1_FLII_FUSION_DN		303	-0.3002785	-1.3950005	0	0.18155132	1	4570	tags=27%, list=21%, signal=34%
REACTOME_ORC1_REMOVAL_FROM_CHROMATIN	REACTOME_ORC1_REMOVAL_FROM_CHROMATIN		63	-0.359867	-1.3937087	0.017241	0.18261497	1	5920	tags=37%, list=27%, signal=50%
DELLA_RESPONSE_TO_TSA_AND_BUTYRATE	DELLA_RESPONSE_TO_TSA_AND_BUTYRATE		20	-0.4774218	-1.3925463	0.080645	0.18360582	1	7408	tags=60%, list=34%, signal=91%
BENPORATH_ES_1	BENPORATH_ES_1		361	-0.2947364	-1.3922372	0	0.1836502	1	4160	tags=23%, list=19%, signal=28%
HELLER_SILENCED_BY_METHYLATION_UP	HELLER_SILENCED_BY_METHYLATION_UP		220	-0.3018065	-1.3916612	0.025641	0.18401702	1	4301	tags=29%, list=20%, signal=35%
HOFMANN_CELL_LYMPHOMA_UP	HOFMANN_CELL_LYMPHOMA_UP		40	-0.3923617	-1.3907387	0.04	0.18508774	1	5001	tags=30%, list=23%, signal=39%
HELLER_SILENCED_BY_METHYLATION_DN	HELLER_SILENCED_BY_METHYLATION_DN		87	-0.3565722	-1.3899853	0.016949	0.18584031	1	4342	tags=32%, list=20%, signal=40%
REACTOME_METABOLISM_OF_CARBOHYDRATES	REACTOME_METABOLISM_OF_CARBOHYDRATES		114	-0.3371306	-1.3897238	0.034483	0.1859497	1	3769	tags=21%, list=17%, signal=25%
NOJIMA_SFRP2_TARGETS_UP	NOJIMA_SFRP2_TARGETS_UP		27	-0.4504967	-1.3894012	0.048387	0.18619111	1	4704	tags=44%, list=21%, signal=57%
KEGG_B_CELL_RECEPTOR_SIGNALING_PATHWAY	KEGG_B_CELL_RECEPTOR_SIGNALING_PATHWAY		75	-0.3570691	-1.388996	0.019231	0.18634444	1	4961	tags=32%, list=23%, signal=41%
WINTER_HYPOXIA_UP	WINTER_HYPOXIA_UP		90	-0.3469227	-1.3876343	0.042553	0.18804294	1	6614	tags=42%, list=30%, signal=60%
MILI_PSEUDOPODIA_CHEMOTAXIS_DN	MILI_PSEUDOPODIA_CHEMOTAXIS_DN		438	-0.2865666	-1.3872832	0	0.18808001	1	6280	tags=35%, list=29%, signal=48%
REACTOME_PLATELET_ACTIVATION_TRIGGERS	REACTOME_PLATELET_ACTIVATION_TRIGGERS		58	-0.3859286	-1.3866264	0.042857	0.18863897	1	4665	tags=34%, list=21%, signal=44%
MITSIADES_RESPONSE_TO_APLIDIN_DN	MITSIADES_RESPONSE_TO_APLIDIN_DN		241	-0.3160512	-1.3858757	0	0.18930532	1	6149	tags=34%, list=28%, signal=46%
BIOCARTA_FAS_PATHWAY	BIOCARTA_FAS_PATHWAY		29	-0.4222214	-1.3850138	0.075472	0.18974383	1	5372	tags=31%, list=24%, signal=41%
MOHANKUMAR_TLX1_TARGETS_UP	MOHANKUMAR_TLX1_TARGETS_UP		379	-0.2892329	-1.38484	0.043478	0.18961953	1	4435	tags=22%, list=20%, signal=28%
WANG_SMARCE1_TARGETS_UP	WANG_SMARCE1_TARGETS_UP		152	-0.3208389	-1.3847077	0.050847	0.18951789	1	3956	tags=23%, list=18%, signal=28%
REACTOME_G1_S_TRANSITION	REACTOME_G1_S_TRANSITION		98	-0.3462588	-1.3846624	0.017857	0.18928213	1	6834	tags=43%, list=31%, signal=62%
NIKOLSKY_BREAST_CANCER_15Q26_AMPLICON	NIKOLSKY_BREAST_CANCER_15Q26_AMPLICON		22	-0.4648594	-1.384657	0.06383	0.18897977	1	3364	tags=27%, list=15%, signal=32%
KIM_WT1_TARGETS_12HR_UP	KIM_WT1_TARGETS_12HR_UP		151	-0.3181428	-1.3846273	0.031746	0.18867835	1	5348	tags=32%, list=24%, signal=42%
ENGELMANN_CANCER_PROGENITORS_DN	ENGELMANN_CANCER_PROGENITORS_DN		60	-0.384261	-1.3838162	0.079365	0.18946925	1	5388	tags=45%, list=25%, signal=59%
WEST_ADRENOCORTICAL_TUMOR_MARKERS_UP	WEST_ADRENOCORTICAL_TUMOR_MARKERS_UP		20	-0.4831405	-1.3829566	0.063492	0.19017243	1	4510	tags=45%, list=21%, signal=57%
HELLER_HDAC_TARGETS_SILENCED_BY_METHYLATION_DN	HELLER_HDAC_TARGETS_SILENCED_BY_METHYLATION_DN		229	-0.3154196	-1.382533	0	0.19026828	1	4583	tags=30%, list=21%, signal=37%
CUI_TCF21_TARGETS_UP	CUI_TCF21_TARGETS_UP		36	-0.4225147	-1.3824683	0.071429	0.18998945	1	4502	tags=33%, list=21%, signal=42%
BEIER_GLIOMA_STEM_CELL_DN	BEIER_GLIOMA_STEM_CELL_DN		56	-0.3771445	-1.3818558	0.05	0.19038129	1	6077	tags=48%, list=28%, signal=67%
KIM_WT1_TARGETS_UP	KIM_WT1_TARGETS_UP		209	-0.3130491	-1.3816941	0	0.1903476	1	4965	tags=29%, list=23%, signal=37%
ZHAN_MULTIPLE_MYELOMA_UP	ZHAN_MULTIPLE_MYELOMA_UP		50	-0.389167	-1.381632	0.066667	0.1900919	1	5159	tags=30%, list=24%, signal=39%
HAN_SATB1_TARGETS_UP	HAN_SATB1_TARGETS_UP		296	-0.302212	-1.3808793	0	0.19070004	1	3568	tags=24%, list=16%, signal=28%
LEE_LIVER_CANCER_MYC_E2F1_DN	LEE_LIVER_CANCER_MYC_E2F1_DN		60	-0.3657902	-1.3803878	0.032258	0.19110548	1	3183	tags=22%, list=15%, signal=25%
FONTAINE_FOLLICULAR_THYROID_ADENOMA_UP	FONTAINE_FOLLICULAR_THYROID_ADENOMA_UP		61	-0.3552093	-1.3803059	0.054545	0.19093756	1	4788	tags=31%, list=22%, signal=40%

BROCKE_APOPTOSIS_REVERSED_BY_IL6	BROCKE_APOPTOSIS_REVERSED_BY_IL6		135	-0.3282916	-1.3802634	0.035714	0.19070356	1	4748	tags=27%, list=22%, signal=35%
RUTELLA_RESPONSE_TO_CSF2RB_AND_IL4_UP	RUTELLA_RESPONSE_TO_CSF2RB_AND_IL4_UP		323	-0.2873403	-1.3800993	0	0.19060417	1	6004	tags=33%, list=27%, signal=44%
ZHU_CMV_24_HR_DN	ZHU_CMV_24_HR_DN		54	-0.3771496	-1.3800195	0.015873	0.19037244	1	3868	tags=33%, list=18%, signal=40%
CHUNG_BLISTER_CYTOTOXICITY_UP	CHUNG_BLISTER_CYTOTOXICITY_UP		122	-0.3465441	-1.3799905	0.072727	0.19007543	1	6465	tags=43%, list=29%, signal=60%
REACTOME_METAL_ION_SLC_TRANSPORTERS	REACTOME_METAL_ION_SLC_TRANSPORTERS		23	-0.4620485	-1.3792614	0.086207	0.19071499	1	4966	tags=35%, list=23%, signal=45%
BIOCARTA_LAIR_PATHWAY	BIOCARTA_LAIR_PATHWAY		16	-0.501789	-1.3788285	0.101695	0.19094148	1	1654	tags=25%, list=8%, signal=27%
ST_WNT_BETA_CATENIN_PATHWAY	ST_WNT_BETA_CATENIN_PATHWAY		31	-0.4220253	-1.3787507	0.043478	0.19075368	1	5472	tags=45%, list=25%, signal=60%
LUCAS_HNF4A_TARGETS_UP	LUCAS_HNF4A_TARGETS_UP		49	-0.388018	-1.3782105	0.04918	0.19113246	1	3643	tags=24%, list=17%, signal=29%
PENG_GLUTAMINE_DEPRIVATION_DN	PENG_GLUTAMINE_DEPRIVATION_DN		82	-0.3570701	-1.3767794	0.016949	0.192119	1	4835	tags=29%, list=22%, signal=37%
WALLACE_PROSTATE_CANCER_UP	WALLACE_PROSTATE_CANCER_UP		18	-0.4983814	-1.3765782	0.074074	0.19206148	1	2301	tags=28%, list=10%, signal=31%
BROWNE_HCMV_INFECTION_48HR_DN	BROWNE_HCMV_INFECTION_48HR_DN		467	-0.2861255	-1.3756646	0	0.19286564	1	4484	tags=24%, list=20%, signal=30%
SONG_TARGETS_OF_IE86_CMV_PROTEIN	SONG_TARGETS_OF_IE86_CMV_PROTEIN		46	-0.4018695	-1.3746145	0.101695	0.19362499	1	6421	tags=48%, list=29%, signal=67%
GENTILE_RESPONSE_CLUSTER_D3	GENTILE_RESPONSE_CLUSTER_D3		44	-0.4123108	-1.3745486	0.084746	0.19345598	1	4531	tags=34%, list=21%, signal=43%
UEDA_PERIFERAL_CLOCK	UEDA_PERIFERAL_CLOCK		149	-0.3315935	-1.373421	0	0.19461806	1	3309	tags=19%, list=15%, signal=23%
PICCALUGA_ANGIOIMMUNOBLASTIC_LYMPHOMA_UP	PICCALUGA_ANGIOIMMUNOBLASTIC_LYMPHOMA_UP		193	-0.3165224	-1.3722739	0.015625	0.19590811	1	3963	tags=25%, list=18%, signal=31%
LEE_NEURAL_CREST_STEM_CELL_UP	LEE_NEURAL_CREST_STEM_CELL_UP		143	-0.3298766	-1.3714272	0.032258	0.19691633	1	3561	tags=27%, list=16%, signal=32%
REACTOME_CELL_JUNCTION_ORGANIZATION	REACTOME_CELL_JUNCTION_ORGANIZATION		83	-0.3636896	-1.3709269	0.0625	0.19730106	1	5301	tags=42%, list=24%, signal=55%
VALK_AML_WITH_CEBPA	VALK_AML_WITH_CEBPA		34	-0.4267749	-1.3708384	0.04918	0.19706339	1	5333	tags=35%, list=24%, signal=47%
TAKEDA_TARGETS_OF_NUP98_HOXA9_FUSION_16D_DN	TAKEDA_TARGETS_OF_NUP98_HOXA9_FUSION_16D_DN		115	-0.3417556	-1.3702118	0.03125	0.19746707	1	1479	tags=11%, list=7%, signal=12%
KEGG_PARKINSONS_DISEASE	KEGG_PARKINSONS_DISEASE		116	-0.3262675	-1.3664588	0	0.20236035	1	5204	tags=30%, list=24%, signal=39%
YAO_TEMPORAL_RESPONSE_TO_PROGESTERONE_CLUSTEF	YAO_TEMPORAL_RESPONSE_TO_PROGESTERONE_CLUSTER_5		29	-0.438855	-1.3664088	0.111111	0.2020737	1	6742	tags=52%, list=31%, signal=75%
LI_WILMS_TUMOR	LI_WILMS_TUMOR		27	-0.4483121	-1.3663999	0.034483	0.2017879	1	1067	tags=22%, list=5%, signal=23%
SEKI_INFLAMMATORY_RESPONSE_LPS_UP	SEKI_INFLAMMATORY_RESPONSE_LPS_UP		76	-0.3439484	-1.3662674	0.04918	0.20167387	1	5426	tags=37%, list=25%, signal=49%
REACTOME_INTEGRATION_OF_ENERGY_METABOLISM	REACTOME_INTEGRATION_OF_ENERGY_METABOLISM		219	-0.3021504	-1.3661473	0	0.20162332	1	6220	tags=35%, list=28%, signal=49%
BROWNE_HCMV_INFECTION_18HR_DN	BROWNE_HCMV_INFECTION_18HR_DN		167	-0.3071829	-1.3660824	0.016949	0.20138325	1	4110	tags=24%, list=19%, signal=29%
STEARMAN_TUMOR_FIELD_EFFECT_UP	STEARMAN_TUMOR_FIELD_EFFECT_UP		36	-0.4115803	-1.3657537	0.072727	0.20139626	1	6077	tags=42%, list=28%, signal=58%
REACTOME_SIGNALING_BY_PDGF	REACTOME_SIGNALING_BY_PDGF		63	-0.3736832	-1.3655038	0.084746	0.20140891	1	5379	tags=41%, list=25%, signal=55%
SESTO_RESPONSE_TO_UV_C7	SESTO_RESPONSE_TO_UV_C7		67	-0.3752006	-1.3650049	0.09375	0.20173915	1	4555	tags=31%, list=21%, signal=39%
OUYANG_PROSTATE_CANCER_PROGRESSION_UP	OUYANG_PROSTATE_CANCER_PROGRESSION_UP		20	-0.4864365	-1.364914	0.083333	0.20152062	1	2776	tags=30%, list=13%, signal=34%
CERVERA_SDHB_TARGETS_1_UP	CERVERA_SDHB_TARGETS_1_UP		111	-0.3297355	-1.3638245	0.04918	0.20244089	1	4058	tags=24%, list=19%, signal=30%
HARRIS_HYPOXIA	HARRIS_HYPOXIA		77	-0.3575093	-1.3637205	0.064516	0.20228714	1	1409	tags=12%, list=6%, signal=12%
LOPEZ_MBD_TARGETS_IMPRINTED_AND_X_LINKED	LOPEZ_MBD_TARGETS_IMPRINTED_AND_X_LINKED		16	-0.4823427	-1.3632436	0.178571	0.20273738	1	2807	tags=38%, list=13%, signal=43%
FARMER_BREAST_CANCER_CLUSTER_7	FARMER_BREAST_CANCER_CLUSTER_7		20	-0.4826493	-1.3631562	0.0625	0.2026233	1	4418	tags=50%, list=20%, signal=63%
HUANG_FOXA2_TARGETS_DN	HUANG_FOXA2_TARGETS_DN		34	-0.4238583	-1.362265	0.067797	0.20355578	1	5649	tags=44%, list=26%, signal=59%
PAPASPYRIDONOS_UNSTABLE_ATEROSCLEROTIC_PLAQUE	PAPASPYRIDONOS_UNSTABLE_ATEROSCLEROTIC_PLAQUE_DN		42	-0.4124508	-1.3617675	0.096154	0.20400138	1	5159	tags=31%, list=24%, signal=40%
BHATI_G2M_ARREST_BY_2METHOXYESTRADIOL_DN	BHATI_G2M_ARREST_BY_2METHOXYESTRADIOL_DN		116	-0.3388943	-1.3604887	0.032787	0.20520644	1	3981	tags=25%, list=18%, signal=30%
LINDSTEDT_DENDRITIC_CELL_MATURATION_B	LINDSTEDT_DENDRITIC_CELL_MATURATION_B		45	-0.3895264	-1.359806	0.089286	0.20598362	1	3393	tags=33%, list=15%, signal=39%
REACTOME_RNA_POLYMERASE_I_PROMOTER_CLEARANCE	REACTOME_RNA_POLYMERASE_I_PROMOTER_CLEARANCE		57	-0.3783621	-1.3595324	0.090909	0.20619921	1	5584	tags=42%, list=25%, signal=56%
BARRIER_CANCER_RELAPSE_NORMAL_SAMPLE_DN	BARRIER_CANCER_RELAPSE_NORMAL_SAMPLE_DN		30	-0.4133227	-1.3582788	0.125	0.2077204	1	7690	tags=67%, list=35%, signal=103%
STEARMAN_LUNG_CANCER_EARLY_VS_LATE_DN	STEARMAN_LUNG_CANCER_EARLY_VS_LATE_DN		58	-0.3630003	-1.3575516	0.072727	0.20838721	1	8505	tags=57%, list=39%, signal=93%
ACEVEDO_LIVER_TUMOR_VS_NORMAL_ADJACENT_TISSUE	ACEVEDO_LIVER_TUMOR_VS_NORMAL_ADJACENT_TISSUE_DN		254	-0.2937037	-1.3574415	0.030303	0.20826545	1	2751	tags=19%, list=13%, signal=21%
KEGG_CITRATE_CYCLE_TCA_CYCLE	KEGG_CITRATE_CYCLE_TCA_CYCLE		30	-0.4295494	-1.3572063	0.135593	0.20822667	1	6595	tags=60%, list=30%, signal=86%
SANA_RESPONSE_TO_IFNG_UP	SANA_RESPONSE_TO_IFNG_UP		56	-0.3821873	-1.3562825	0.0625	0.20928237	1	4599	tags=36%, list=21%, signal=45%
WATTEL_AUTONOMOUS_THYROID_ADENOMA_DN	WATTEL_AUTONOMOUS_THYROID_ADENOMA_DN		23	-0.4570569	-1.3559147	0.137255	0.20934395	1	2336	tags=26%, list=11%, signal=29%
CHEMNITZ_RESPONSE_TO_PROSTAGLANDIN_E2_DN	CHEMNITZ_RESPONSE_TO_PROSTAGLANDIN_E2_DN		331	-0.2888607	-1.3555439	0.013699	0.20957184	1	3118	tags=19%, list=14%, signal=22%
REACTOME_AMINO_ACID_TRANSPORT_ACROSS_THE_PLAS	REACTOME_AMINO_ACID_TRANSPORT_ACROSS_THE_PLASMA_M		31	-0.4324964	-1.3553802	0.089286	0.20949253	1	3041	tags=26%, list=14%, signal=30%
TONKS_TARGETS_OF_RUNX1_RUNX1T1_FUSION_ERYTHRO	TONKS_TARGETS_OF_RUNX1_RUNX1T1_FUSION_ERYTHROCYTE		147	-0.3264784	-1.3551428	0.050847	0.20935088	1	5470	tags=33%, list=25%, signal=43%
SASSON_RESPONSE_TO_GONADOTROPHINS_DN	SASSON_RESPONSE_TO_GONADOTROPHINS_DN		67	-0.3446213	-1.3550825	0.033898	0.20910768	1	4922	tags=37%, list=22%, signal=48%
BIOCARTA_MPR_PATHWAY	BIOCARTA_MPR_PATHWAY		32	-0.391356	-1.3548105	0.114754	0.20935224	1	6105	tags=44%, list=28%, signal=61%
LEE_LIVER_CANCER_DENA_DN	LEE_LIVER_CANCER_DENA_DN		71	-0.3592981	-1.3531054	0	0.21143933	1	3097	tags=18%, list=14%, signal=21%
BORCZUK_MALIGNANT_MESOTHELIOMA_DN	BORCZUK_MALIGNANT_MESOTHELIOMA_DN		93	-0.3519407	-1.3527958	0.048387	0.2115618	1	4526	tags=31%, list=21%, signal=39%
MARKEY_RB1_ACUTE_LOF_UP	MARKEY_RB1_ACUTE_LOF_UP		221	-0.3055386	-1.3520954	0	0.21215165	1	4416	tags=31%, list=20%, signal=38%
MULLIGHAN_NPM1_MUTATED_SIGNATURE_1_DN	MULLIGHAN_NPM1_MUTATED_SIGNATURE_1_DN		118	-0.3276118	-1.3517356	0.048387	0.2123708	1	4785	tags=28%, list=22%, signal=36%
BIOCARTA_KERATINOCYTE_PATHWAY	BIOCARTA_KERATINOCYTE_PATHWAY		45	-0.3766491	-1.3515632	0.086957	0.21224554	1	4961	tags=31%, list=23%, signal=40%
HAHTOLA_SEZARY_SYNDROM_UP	HAHTOLA_SEZARY_SYNDROM_UP		94	-0.3369543	-1.34662	0.044776	0.21874636	1	3407	tags=22%, list=16%, signal=26%
ZHOU_INFLAMMATORY_RESPONSE_FIMA_UP	ZHOU_INFLAMMATORY_RESPONSE_FIMA_UP		461	-0.2714953	-1.3464594	0	0.21877517	1	4762	tags=26%, list=22%, signal=33%
SHAFFER_IRF4_TARGETS_IN_ACTIVATED_B_LYMPHOCYTE	SHAFFER_IRF4_TARGETS_IN_ACTIVATED_B_LYMPHOCYTE		78	-0.3412852	-1.3462902	0.015625	0.21870136	1	5004	tags=27%, list=23%, signal=35%
WILCOX_PRESPONSE_TO_ROGESTERONE_UP	WILCOX_PRESPONSE_TO_ROGESTERONE_UP		140	-0.3063108	-1.3459349	0.032787	0.21881086	1	4684	tags=29%, list=21%, signal=37%
BILD_E2F3_ONCOGENIC_SIGNATURE	BILD_E2F3_ONCOGENIC_SIGNATURE		224	-0.306703	-1.3457599	0.017241	0.21880157	1	5286	tags=32%, list=24%, signal=41%
REACTOME_DNA_REPLICATION_PRE_INITIATION	REACTOME_DNA_REPLICATION_PRE_INITIATION		74	-0.3429309	-1.345345	0.029412	0.21917218	1	6834	tags=42%, list=31%, signal=61%
REACTOME_SIGNALING_BY_TGF_BETA	REACTOME_SIGNALING_BY_TGF_BETA		15	-0.5457745	-1.3446934	0.12963	0.21976513	1	4938	tags=40%, list=23%, signal=52%
BIOCARTA_INFLAM_PATHWAY	BIOCARTA_INFLAM_PATHWAY		28	-0.4022037	-1.3439629	0.107143	0.22051239	1	2761	tags=25%, list=13%, signal=29%
MOOTHA_GLUCONEOGENESIS	MOOTHA_GLUCONEOGENESIS		31	-0.4475397	-1.3438789	0.067797	0.22029595	1	3333	tags=29%, list=15%, signal=34%
DOANE_RESPONSE_TO_ANDROGEN_DN	DOANE_RESPONSE_TO_ANDROGEN_DN		235	-0.3045488	-1.3438208	0.016393	0.22012247	1	3970	tags=24%, list=18%, signal=29%

VART_KSHV_INFECTION_ANGIOGENIC_MARKERS_UP	VART_KSHV_INFECTION_ANGIOGENIC_MARKERS_UP		162	-0.3046807	-1.3427215	0	0.22126539	1	5026	tags=35%, list=23%, signal=45%
WIELAND_UP_BY_HBV_INFECTION	WIELAND_UP_BY_HBV_INFECTION		86	-0.3531738	-1.3424406	0.059701	0.22141261	1	6713	tags=48%, list=31%, signal=68%
LIAO_METASTASIS	LIAO_METASTASIS		498	-0.2693743	-1.3419447	0	0.22183128	1	5636	tags=31%, list=26%, signal=40%
FONTAINE_PAPILLARY_THYROID_CARCINOMA_UP	FONTAINE_PAPILLARY_THYROID_CARCINOMA_UP		58	-0.3699874	-1.3403584	0.034483	0.22340405	1	5789	tags=45%, list=26%, signal=61%
STEIN_ESRRA_TARGETS_UP	STEIN_ESRRA_TARGETS_UP		364	-0.2794417	-1.3391745	0.014925	0.22479558	1	5477	tags=29%, list=25%, signal=39%
LIU_NASOPHARYNGEAL_CARCINOMA	LIU_NASOPHARYNGEAL_CARCINOMA		59	-0.3616864	-1.3386718	0.066667	0.22499317	1	4163	tags=27%, list=19%, signal=33%
BIOCARTA_MCM_PATHWAY	BIOCARTA_MCM_PATHWAY		18	-0.4991374	-1.3385438	0.076923	0.2248545	1	5631	tags=50%, list=26%, signal=67%
MUELLER_COMMON_TARGETS_OF_AML_FUSIONS_DN	MUELLER_COMMON_TARGETS_OF_AML_FUSIONS_DN		22	-0.4784732	-1.3378303	0.148148	0.22539055	1	6272	tags=55%, list=29%, signal=76%
RIGGI_EWING_SARCOMA_PROGENITOR_UP	RIGGI_EWING_SARCOMA_PROGENITOR_UP		393	-0.2817654	-1.3378043	0	0.22509257	1	2839	tags=18%, list=13%, signal=20%
MCCABE_HOXC6_TARGETS_CANCER_DN	MCCABE_HOXC6_TARGETS_CANCER_DN		20	-0.4511075	-1.3367937	0.075472	0.22624145	1	922	tags=15%, list=4%, signal=16%
FARMER_BREAST_CANCER_APOCRINE_VS_BASAL	FARMER_BREAST_CANCER_APOCRINE_VS_BASAL		317	-0.2798086	-1.3355793	0	0.22765574	1	4753	tags=25%, list=22%, signal=31%
VARELA_ZMPSTE24_TARGETS_UP	VARELA_ZMPSTE24_TARGETS_UP		39	-0.3812576	-1.3345504	0.115385	0.22871973	1	3334	tags=23%, list=15%, signal=27%
MULLIGHAN_NPM1_SIGNATURE_3_DN	MULLIGHAN_NPM1_SIGNATURE_3_DN		154	-0.3122019	-1.3344083	0.037736	0.22865716	1	5986	tags=36%, list=27%, signal=49%
FLECHNER_PBL_KIDNEY_TRANSPLANT_REJECTED_VS_OK_	FLECHNER_PBL_KIDNEY_TRANSPLANT_REJECTED_VS_OK_UP		61	-0.3406923	-1.3343141	0.086207	0.22845474	1	3511	tags=18%, list=16%, signal=21%
PARENT_MTOR_SIGNALING_DN	PARENT_MTOR_SIGNALING_DN		43	-0.3917261	-1.33422	0.107143	0.22825521	1	4106	tags=33%, list=19%, signal=40%
REACTOME_TRANSMEMBRANE_TRANSPORT_OF_SMALL_MC	REACTOME_TRANSMEMBRANE_TRANSPORT_OF_SMALL_MOLECU		216	-0.3012838	-1.3339068	0.015873	0.22854489	1	3041	tags=19%, list=14%, signal=21%
HOQUE_METHYLATED_IN_CANCER	HOQUE_METHYLATED_IN_CANCER		55	-0.3727732	-1.3337034	0.084746	0.22848013	1	5001	tags=38%, list=23%, signal=49%
HWANG_PROSTATE_CANCER_MARKERS	HWANG_PROSTATE_CANCER_MARKERS		27	-0.4302675	-1.3333267	0.040816	0.22878873	1	1143	tags=11%, list=5%, signal=12%
SENGUPTA_NASOPHARYNGEAL_CARCINOMA_DN	SENGUPTA_NASOPHARYNGEAL_CARCINOMA_DN		319	-0.2843737	-1.3331134	0.015625	0.22872446	1	4538	tags=25%, list=21%, signal=31%
DOUGLAS_BMI1_TARGETS_UP	DOUGLAS_BMI1_TARGETS_UP		491	-0.275184	-1.3330619	0	0.22846639	1	5429	tags=29%, list=25%, signal=38%
LIEN_BREAST_CARCINOMA_METAPLASTIC_VS_DUCTAL_UP	LIEN_BREAST_CARCINOMA_METAPLASTIC_VS_DUCTAL_UP		77	-0.3417617	-1.3328784	0.079365	0.22840194	1	4967	tags=34%, list=23%, signal=44%
MEINHOLD_OVARIAN_CANCER_LOW_GRADE_DN	MEINHOLD_OVARIAN_CANCER_LOW_GRADE_DN		18	-0.4636286	-1.3323265	0.122449	0.22907603	1	4049	tags=39%, list=18%, signal=48%
BIOCARTA_P53_PATHWAY	BIOCARTA_P53_PATHWAY		16	-0.5076199	-1.3321873	0.071429	0.22895397	1	6598	tags=50%, list=30%, signal=71%
LINDSTEDT_DENDRITIC_CELL_MATURATION_C	LINDSTEDT_DENDRITIC_CELL_MATURATION_C		56	-0.3630804	-1.3313779	0.066667	0.22962457	1	3550	tags=23%, list=16%, signal=28%
KYNG_DNA_DAMAGE_UP	KYNG_DNA_DAMAGE_UP		97	-0.3286965	-1.3311919	0.065574	0.22961818	1	4360	tags=31%, list=20%, signal=38%
AMUNDSON_POOR_SURVIVAL_AFTER_GAMMA_RADIATION	AMUNDSON_POOR_SURVIVAL_AFTER_GAMMA_RADIATION_8G		83	-0.3519059	-1.3309008	0.065574	0.22966814	1	6592	tags=36%, list=30%, signal=51%
LE_EGR2_TARGETS_DN	LE_EGR2_TARGETS_DN		100	-0.3292307	-1.3308742	0.032787	0.2293915	1	5836	tags=30%, list=27%, signal=41%
LI_CISPLATIN_RESISTANCE_DN	LI_CISPLATIN_RESISTANCE_DN		32	-0.3997607	-1.3298932	0.114754	0.2302159	1	2807	tags=28%, list=13%, signal=32%
FRASOR_TAMOXIFEN_RESPONSE_UP	FRASOR_TAMOXIFEN_RESPONSE_UP		48	-0.3747555	-1.3291831	0.103448	0.23103908	1	5549	tags=38%, list=25%, signal=50%
ZIRN_TRETINOIN_RESPONSE_WT1_UP	ZIRN_TRETINOIN_RESPONSE_WT1_UP		18	-0.4708737	-1.3277143	0.166667	0.23302704	1	3619	tags=44%, list=17%, signal=53%
LEE_LIVER_CANCER_SURVIVAL_DN	LEE_LIVER_CANCER_SURVIVAL_DN		119	-0.3218432	-1.327396	0.0625	0.23332332	1	4037	tags=22%, list=18%, signal=27%
KEGG_GLYOXYLATE_AND_DICARBOXYLATE_METABOLISM	KEGG_GLYOXYLATE_AND_DICARBOXYLATE_METABOLISM		16	-0.4940974	-1.3272868	0.140351	0.23321556	1	4594	tags=38%, list=21%, signal=47%
FONTAINE_THYROID_TUMOR_UNCERTAIN_MALIGNANCY_U	FONTAINE_THYROID_TUMOR_UNCERTAIN_MALIGNANCY_UP		31	-0.4111542	-1.3244835	0.101695	0.23700204	1	4788	tags=35%, list=22%, signal=45%
WNT_SIGNALING	WNT_SIGNALING		89	-0.3322672	-1.3238341	0.084746	0.23765235	1	5472	tags=35%, list=25%, signal=46%
LIU_PROSTATE_CANCER_DN	LIU_PROSTATE_CANCER_DN		443	-0.2708224	-1.3237774	0.028571	0.23734893	1	4362	tags=23%, list=20%, signal=28%
YAO_TEMPORAL_RESPONSE_TO_PROGESTERONE_CLUSTEF	YAO_TEMPORAL_RESPONSE_TO_PROGESTERONE_CLUSTER_15		31	-0.4278236	-1.3235012	0.109091	0.23736987	1	2899	tags=29%, list=13%, signal=33%
BIOCARTA_TNFR1_PATHWAY	BIOCARTA_TNFR1_PATHWAY		29	-0.4144193	-1.3234017	0.147059	0.23720078	1	543	tags=10%, list=2%, signal=11%
KEGG_AMINO_SUGAR_AND_NUCLEOTIDE_SUGAR_METABC	KEGG_AMINO_SUGAR_AND_NUCLEOTIDE_SUGAR_METABOLISM		44	-0.3871863	-1.3229078	0.090909	0.23775363	1	7364	tags=52%, list=34%, signal=79%
KEGG_INSULIN_SIGNALING_PATHWAY	KEGG_INSULIN_SIGNALING_PATHWAY		135	-0.316873	-1.3223257	0.0625	0.23832439	1	5837	tags=33%, list=27%, signal=45%
KEGG_CELL_CYCLE	KEGG_CELL_CYCLE		121	-0.3174299	-1.3216547	0.04918	0.23916021	1	4063	tags=22%, list=19%, signal=27%
KEGG_FC_EPSILON_RI_SIGNALING_PATHWAY	KEGG_FC_EPSILON_RI_SIGNALING_PATHWAY		78	-0.3674631	-1.3204818	0.118644	0.24050501	1	4961	tags=33%, list=23%, signal=43%
MANALO_HYPOXIA_UP	MANALO_HYPOXIA_UP		198	-0.2926371	-1.3202342	0.014085	0.24052133	1	5348	tags=28%, list=24%, signal=37%
SA_PTEN_PATHWAY	SA_PTEN_PATHWAY		16	-0.4545694	-1.3194506	0.086957	0.2411202	1	4961	tags=50%, list=23%, signal=65%
HOFFMANN_PRE_BI_TO_LARGE_PRE_BII_LYMPHOCYTE_UP	HOFFMANN_PRE_BI_TO_LARGE_PRE_BII_LYMPHOCYTE_UP		19	-0.4626589	-1.3193674	0.155172	0.24089117	1	3670	tags=42%, list=17%, signal=51%
CLASPER_LYMPHATIC_VESSELS_DURING_METASTASIS_UP	CLASPER_LYMPHATIC_VESSELS_DURING_METASTASIS_UP		19	-0.4822244	-1.3176446	0.137255	0.24312592	1	2658	tags=37%, list=12%, signal=42%
KANNAN_TP53_TARGETS_UP	KANNAN_TP53_TARGETS_UP		45	-0.3860395	-1.3168741	0.078431	0.24394976	1	7069	tags=47%, list=32%, signal=69%
HAHTOLA_MYCOSIS_FUNGOIDES_SKIN_DN	HAHTOLA_MYCOSIS_FUNGOIDES_SKIN_DN		27	-0.4458506	-1.3165629	0.117647	0.24409178	1	3042	tags=26%, list=14%, signal=30%
TONKS_TARGETS_OF_RUNX1_RUNX1T1_FUSION_SUSTAIN	TONKS_TARGETS_OF_RUNX1_RUNX1T1_FUSION_SUSTAINED_IN		20	-0.4853618	-1.3162825	0.173913	0.24413837	1	4965	tags=50%, list=23%, signal=65%
GRADE_COLON_VS_RECTAL_CANCER_DN	GRADE_COLON_VS_RECTAL_CANCER_DN		39	-0.3686369	-1.3155504	0.04918	0.24487433	1	2479	tags=21%, list=11%, signal=23%
XU_CREBBP_TARGETS_DN	XU_CREBBP_TARGETS_DN		42	-0.3933645	-1.3155024	0.142857	0.24458611	1	6713	tags=55%, list=31%, signal=79%
BLUM_RESPONSE_TO_SALIRASIB_DN	BLUM_RESPONSE_TO_SALIRASIB_DN		331	-0.2800288	-1.3147526	0	0.24543868	1	4531	tags=26%, list=21%, signal=32%
WILLIAMS_ESR2_TARGETS_UP	WILLIAMS_ESR2_TARGETS_UP		19	-0.4740635	-1.3141351	0.14	0.2460442	1	3523	tags=32%, list=16%, signal=38%
REACTOME_PKA_ACTIVATION	REACTOME_PKA_ACTIVATION		16	-0.5164042	-1.3141173	0.127273	0.24581106	1	5767	tags=56%, list=26%, signal=76%
BIOCARTA_MCALPAIN_PATHWAY	BIOCARTA_MCALPAIN_PATHWAY		24	-0.4278183	-1.3134996	0.092593	0.24630626	1	4961	tags=50%, list=23%, signal=65%
BYSTRYKH_HEMATOPOIESIS_STEM_CELL_QTL_CIS	BYSTRYKH_HEMATOPOIESIS_STEM_CELL_QTL_CIS		126	-0.3113518	-1.3134891	0	0.2459994	1	6380	tags=38%, list=29%, signal=53%
CHIANG_LIVER_CANCER_SUBCLASS_POLYSOMY7_UP	CHIANG_LIVER_CANCER_SUBCLASS_POLYSOMY7_UP		59	-0.3651578	-1.3127325	0.127273	0.24684714	1	6512	tags=47%, list=30%, signal=67%
MOOTHA_GLYCOLYSIS	MOOTHA_GLYCOLYSIS		21	-0.4398826	-1.3125561	0.066667	0.2467988	1	2761	tags=24%, list=13%, signal=27%
PUJANA_BRCA2_PCC_NETWORK	PUJANA_BRCA2_PCC_NETWORK		403	-0.2698135	-1.3121771	0	0.24700749	1	4548	tags=23%, list=21%, signal=29%
KEGG_CYTOKINE_CYTOKINE_RECEPTOR_INTERACTION	KEGG_CYTOKINE_CYTOKINE_RECEPTOR_INTERACTION		231	-0.2981003	-1.3115202	0.057971	0.2475875	1	2842	tags=19%, list=13%, signal=21%
AMIT_SERUM_RESPONSE_120_MCF10A	AMIT_SERUM_RESPONSE_120_MCF10A		65	-0.3606587	-1.311426	0.125	0.2473726	1	7222	tags=48%, list=33%, signal=71%
OZEN_MIR125B1_TARGETS	OZEN_MIR125B1_TARGETS		15	-0.5064374	-1.3110509	0.140351	0.24772787	1	4921	tags=60%, list=22%, signal=77%
PEREZ_TP53_AND_TP63_TARGETS	PEREZ_TP53_AND_TP63_TARGETS		185	-0.2973852	-1.3103081	0.016949	0.24845468	1	4059	tags=27%, list=19%, signal=33%
KANG_IMMORTALIZED_BY_TERT_DN	KANG_IMMORTALIZED_BY_TERT_DN		98	-0.3360227	-1.3100208	0.055556	0.24844001	1	4071	tags=30%, list=19%, signal=36%
STEIN_ESRRA_TARGETS	STEIN_ESRRA_TARGETS		499	-0.2637495	-1.309224	0	0.249091	1	6595	tags=35%, list=30%, signal=49%

YAO_TEMPORAL_RESPONSE_TO_PROGESTERONE_CLUSTEF	YAO_TEMPORAL_RESPONSE_TO_PROGESTERONE_CLUSTER_13		160	-0.3089937	-1.3082416	0.035714	0.2503226	1	5401	tags=29%, list=25%, signal=39%
KEGG_GLYCEROPHOSPHOLIPID_METABOLISM	KEGG_GLYCEROPHOSPHOLIPID_METABOLISM		76	-0.3429627	-1.30774	0.072727	0.25074536	1	3275	tags=24%, list=15%, signal=28%
BERTUCCI_INVASIVE_CARCINOMA_DUCTAL_VS_LOBULAR	BERTUCCI_INVASIVE_CARCINOMA_DUCTAL_VS_LOBULAR_UP		24	-0.4284105	-1.307087	0.15	0.25149253	1	5262	tags=50%, list=24%, signal=66%
LI_CYTIDINE_ANALOG_PATHWAY	LI_CYTIDINE_ANALOG_PATHWAY		16	-0.4815294	-1.3058822	0.178571	0.25304618	1	4263	tags=44%, list=19%, signal=54%
MORI_PRE_BI_LYMPHOCYTE_DN	MORI_PRE_BI_LYMPHOCYTE_DN		65	-0.3526362	-1.3049253	0.075758	0.253937	1	4962	tags=35%, list=23%, signal=46%
HELLER_HDAC_TARGETS_DN	HELLER_HDAC_TARGETS_DN		238	-0.2895376	-1.3046238	0.044776	0.25395334	1	4578	tags=27%, list=21%, signal=34%
SUNG_METASTASIS_STROMA_UP	SUNG_METASTASIS_STROMA_UP		103	-0.3218002	-1.3045883	0.066667	0.25366122	1	4757	tags=30%, list=22%, signal=38%
MARTORIATI_MDM4_TARGETS_FETAL_LIVER_DN	MARTORIATI_MDM4_TARGETS_FETAL_LIVER_DN		263	-0.2791348	-1.3044732	0.059701	0.25356907	1	4840	tags=25%, list=22%, signal=32%
COATES_MACROPHAGE_M1_VS_M2_UP	COATES_MACROPHAGE_M1_VS_M2_UP		77	-0.3412059	-1.3033273	0.096154	0.2549614	1	4518	tags=26%, list=21%, signal=33%
VECCHI_GASTRIC_CANCER_EARLY_DN	VECCHI_GASTRIC_CANCER_EARLY_DN		319	-0.2750062	-1.3017838	0.032787	0.2573435	1	1795	tags=12%, list=8%, signal=13%
CHEN_LVAD_SUPPORT_OF_FAILING_HEART_UP	CHEN_LVAD_SUPPORT_OF_FAILING_HEART_UP		97	-0.3261648	-1.3011338	0.076923	0.25806704	1	5186	tags=36%, list=24%, signal=47%
PENG_LEUCINE_DEPRIVATION_UP	PENG_LEUCINE_DEPRIVATION_UP		21	-0.4321292	-1.3010843	0.185185	0.25780714	1	4835	tags=48%, list=22%, signal=61%
LEE_LIVER_CANCER_SURVIVAL_UP	LEE_LIVER_CANCER_SURVIVAL_UP		120	-0.2994421	-1.3008944	0.036364	0.25783947	1	5126	tags=31%, list=23%, signal=40%
MACLACHLAN_BRCA1_TARGETS_DN	MACLACHLAN_BRCA1_TARGETS_DN		16	-0.4645314	-1.299664	0.1875	0.25929832	1	4623	tags=38%, list=21%, signal=47%
SESTO_RESPONSE_TO_UV_C4	SESTO_RESPONSE_TO_UV_C4		20	-0.4753124	-1.2995219	0.189655	0.25914517	1	2454	tags=25%, list=11%, signal=28%
LAIHO_COLORECTAL_CANCER_SERRATED_DN	LAIHO_COLORECTAL_CANCER_SERRATED_DN		74	-0.3213672	-1.2990441	0.096154	0.25951132	1	3542	tags=23%, list=16%, signal=27%
KIM_WT1_TARGETS_8HR_UP	KIM_WT1_TARGETS_8HR_UP		159	-0.3065223	-1.2982548	0.066667	0.26029086	1	4868	tags=26%, list=22%, signal=34%
BIOCARTA_HIVNEF_PATHWAY	BIOCARTA_HIVNEF_PATHWAY		56	-0.3423568	-1.2979914	0.073529	0.26040667	1	7244	tags=39%, list=33%, signal=59%
JIANG_VHL_TARGETS	JIANG_VHL_TARGETS		88	-0.3264784	-1.2969942	0.104167	0.26161414	1	5629	tags=34%, list=26%, signal=46%
TAKEDA_TARGETS_OF_NUP98_HOXA9_FUSION_6HR_DN	TAKEDA_TARGETS_OF_NUP98_HOXA9_FUSION_6HR_DN		36	-0.4105007	-1.2955922	0.08	0.2636232	1	2780	tags=28%, list=13%, signal=32%
SMID_BREAST_CANCER_NORMAL_LIKE_UP	SMID_BREAST_CANCER_NORMAL_LIKE_UP		427	-0.2767451	-1.2946047	0	0.2647133	1	3570	tags=22%, list=16%, signal=26%
KEGG_ALZHEIMERS_DISEASE	KEGG_ALZHEIMERS_DISEASE		155	-0.2976625	-1.2944028	0.060606	0.26462883	1	5204	tags=31%, list=24%, signal=40%
LINDGREN_BLADDER_CANCER_HIGH_RECURRENCE	LINDGREN_BLADDER_CANCER_HIGH_RECURRENCE		42	-0.3845278	-1.2937769	0.114754	0.2654655	1	5594	tags=40%, list=26%, signal=54%
GENTILE_UV_RESPONSE_CLUSTER_D8	GENTILE_UV_RESPONSE_CLUSTER_D8		27	-0.4136095	-1.2936947	0.078431	0.2653251	1	5608	tags=41%, list=26%, signal=55%
LEE_DIFFERENTIATING_T_LYMPHOCYTE	LEE_DIFFERENTIATING_T_LYMPHOCYTE		125	-0.3130592	-1.293461	0.095238	0.26530883	1	6905	tags=42%, list=31%, signal=60%
WATANABE_RECTAL_CANCER_RADIOTHERAPY_RESPONSIV	WATANABE_RECTAL_CANCER_RADIOTHERAPY_RESPONSIVE_DN		87	-0.3364374	-1.2928003	0.067797	0.265772	1	5842	tags=37%, list=27%, signal=50%
HOFFMANN_SMALL_PRE_BII_TO_IMMATURE_B_LYMPHOCY	HOFFMANN_SMALL_PRE_BII_TO_IMMATURE_B_LYMPHOCYTE_UP		40	-0.4081624	-1.2926067	0.066667	0.2657893	1	5961	tags=45%, list=27%, signal=62%
OUELLET_CULTURED_OVARIAN_CANCER_INVASIVE_VS_LM	OUELLET_CULTURED_OVARIAN_CANCER_INVASIVE_VS_LMP_UP		66	-0.3606082	-1.2922033	0.107143	0.26603824	1	7193	tags=44%, list=33%, signal=65%
RASHI_RESPONSE_TO_IONIZING_RADIATION_6	RASHI_RESPONSE_TO_IONIZING_RADIATION_6		94	-0.3159889	-1.2921797	0.0625	0.26575676	1	4723	tags=30%, list=22%, signal=38%
HINATA_NFKB_TARGETS_FIBROBLAST_UP	HINATA_NFKB_TARGETS_FIBROBLAST_UP		64	-0.3506377	-1.29008	0.12	0.26883382	1	4307	tags=28%, list=20%, signal=35%
VERRECCHIA_DELAYED_RESPONSE_TO_TGFB1	VERRECCHIA_DELAYED_RESPONSE_TO_TGFB1		36	-0.370533	-1.289813	0.118644	0.26888433	1	3587	tags=28%, list=16%, signal=33%
CLASPER_LYMPHATIC_VESSELS_DURING_METASTASIS_DN	CLASPER_LYMPHATIC_VESSELS_DURING_METASTASIS_DN		35	-0.3846242	-1.288545	0.2	0.2709411	1	2002	tags=26%, list=9%, signal=28%
WU_APOPTOSIS_BY_CDKN1A_VIA_TP53	WU_APOPTOSIS_BY_CDKN1A_VIA_TP53		36	-0.3831026	-1.2878335	0.218182	0.27201104	1	4049	tags=31%, list=18%, signal=37%
CAIRO_HEPATOBLASTOMA_POOR_SURVIVAL	CAIRO_HEPATOBLASTOMA_POOR_SURVIVAL		16	-0.5072897	-1.2876565	0.226415	0.27205604	1	4654	tags=44%, list=21%, signal=55%
GINESTIER_BREAST_CANCER_ZNF217_AMPLIFIED_DN	GINESTIER_BREAST_CANCER_ZNF217_AMPLIFIED_DN		308	-0.2817927	-1.2873574	0.086957	0.27241933	1	5963	tags=36%, list=27%, signal=48%
RUTELLA_RESPONSE_TO_CSF2RB_AND_IL4_DN	RUTELLA_RESPONSE_TO_CSF2RB_AND_IL4_DN		291	-0.2780023	-1.2867733	0.014493	0.27299255	1	4375	tags=23%, list=20%, signal=28%
BROWNE_HCMV_INFECTION_48HR_UP	BROWNE_HCMV_INFECTION_48HR_UP		167	-0.2886143	-1.2866918	0.074627	0.27277505	1	7310	tags=43%, list=33%, signal=64%
PENG_RAPAMYCIN_RESPONSE_DN	PENG_RAPAMYCIN_RESPONSE_DN		67	-0.3270355	-1.2864487	0.111111	0.27281907	1	6834	tags=40%, list=31%, signal=58%
SANA_TNF_SIGNALING_UP	SANA_TNF_SIGNALING_UP		71	-0.3303209	-1.2863983	0.163636	0.27258486	1	5426	tags=39%, list=25%, signal=52%
ZHOU_INFLAMMATORY_RESPONSE_LPS_UP	ZHOU_INFLAMMATORY_RESPONSE_LPS_UP		362	-0.2791495	-1.2862817	0.048387	0.27247328	1	4255	tags=27%, list=19%, signal=33%
REACTOME_INORGANIC_CATION_ANION_SLC_TRANSPORT	REACTOME_INORGANIC_CATION_ANION_SLC_TRANSPORTERS		94	-0.3189484	-1.2862414	0.111111	0.27220577	1	3041	tags=20%, list=14%, signal=23%
THEILGAARD_NEUTROPHIL_AT_SKIN_WOUND_DN	THEILGAARD_NEUTROPHIL_AT_SKIN_WOUND_DN		219	-0.2835608	-1.2841486	0.055556	0.27543688	1	5905	tags=32%, list=27%, signal=43%
DOANE_RESPONSE_TO_ANDROGEN_UP	DOANE_RESPONSE_TO_ANDROGEN_UP		159	-0.2984988	-1.2841159	0.04918	0.27514857	1	5691	tags=38%, list=26%, signal=51%
HESS_TARGETS_OF_HOXA9_AND_MEIS1_UP	HESS_TARGETS_OF_HOXA9_AND_MEIS1_UP		60	-0.3467574	-1.2835641	0.096154	0.27583224	1	4263	tags=28%, list=19%, signal=35%
REACTOME_CELL_SURFACE_INTERACTIONS_AT_THE_VASC	REACTOME_CELL_SURFACE_INTERACTIONS_AT_THE_VASCULAR		90	-0.3320246	-1.2835586	0.086207	0.2754917	1	4921	tags=32%, list=22%, signal=41%
VANTVEER_BREAST_CANCER_METASTASIS_DN	VANTVEER_BREAST_CANCER_METASTASIS_DN		105	-0.3321193	-1.2829032	0.076923	0.27605286	1	4070	tags=23%, list=19%, signal=28%
BIOCARTA_TOB1_PATHWAY	BIOCARTA_TOB1_PATHWAY		19	-0.4480805	-1.2827384	0.226415	0.275921	1	3619	tags=32%, list=17%, signal=38%
ELVIDGE_HYPOXIA_UP	ELVIDGE_HYPOXIA_UP		165	-0.2944699	-1.282372	0.047619	0.2761875	1	5208	tags=27%, list=24%, signal=35%
REACTOME_GENES_INVOLVED_IN_APOPTOTIC_CLEAVAGE	REACTOME_GENES_INVOLVED_IN_APOPTOTIC_CLEAVAGE_OF_C		35	-0.390552	-1.2816807	0.211538	0.2768303	1	6433	tags=37%, list=29%, signal=52%
TURASHVILI_BREAST_LOBULAR_CARCINOMA_VS_LOBULAF	TURASHVILI_BREAST_LOBULAR_CARCINOMA_VS_LOBULAR_NOF		65	-0.3451949	-1.2815909	0.061224	0.2766454	1	4331	tags=31%, list=20%, signal=38%
TONKS_TARGETS_OF_RUNX1_RUNX1T1_FUSION_MONOCY	TONKS_TARGETS_OF_RUNX1_RUNX1T1_FUSION_MONOCYTE_UF		195	-0.276094	-1.2813281	0.045455	0.27687415	1	5612	tags=33%, list=26%, signal=44%
HUTTMANN_B_CLL_POOR_SURVIVAL_UP	HUTTMANN_B_CLL_POOR_SURVIVAL_UP		259	-0.2745609	-1.280881	0.047619	0.27732664	1	5003	tags=30%, list=23%, signal=39%
REACTOME_REPAIR_SYNTHESIS_OF_PATCH_27_30_BASES	REACTOME_REPAIR_SYNTHESIS_OF_PATCH_27_30_BASES_LON		15	-0.5103508	-1.280827	0.173077	0.277073	1	2870	tags=33%, list=13%, signal=38%
CROONQUIST_NRAS_VS_STROMAL_STIMULATION_UP	CROONQUIST_NRAS_VS_STROMAL_STIMULATION_UP		34	-0.3971682	-1.2806598	0.145833	0.2769561	1	3934	tags=29%, list=18%, signal=36%
DACOSTA_ERCC3_ALLELE_XPCS_VS_TTD_DN	DACOSTA_ERCC3_ALLELE_XPCS_VS_TTD_DN		27	-0.4314725	-1.2802628	0.169492	0.27722028	1	2619	tags=22%, list=12%, signal=25%
NIKOLSKY_BREAST_CANCER_16P13_AMPLICON	NIKOLSKY_BREAST_CANCER_16P13_AMPLICON		106	-0.3292896	-1.2802305	0.084746	0.27695045	1	3345	tags=24%, list=15%, signal=28%
MCBRYAN_PUBERTAL_BREAST_6_7WK_UP	MCBRYAN_PUBERTAL_BREAST_6_7WK_UP		180	-0.3080428	-1.2799386	0.053571	0.27707672	1	5026	tags=27%, list=23%, signal=35%
WILLIAMS_ESR1_TARGETS_UP	WILLIAMS_ESR1_TARGETS_UP		22	-0.4072405	-1.2794845	0.152542	0.2773865	1	3925	tags=41%, list=18%, signal=50%
ABE_VEGFA_TARGETS_2HR	ABE_VEGFA_TARGETS_2HR		18	-0.4662139	-1.278447	0.157895	0.27902833	1	2383	tags=22%, list=11%, signal=25%
BROWNE_HCMV_INFECTION_20HR_UP	BROWNE_HCMV_INFECTION_20HR_UP		220	-0.2808494	-1.2778435	0.070423	0.27952227	1	3371	tags=22%, list=15%, signal=26%
HOOI_ST7_TARGETS_DN	HOOI_ST7_TARGETS_DN		104	-0.307675	-1.2771107	0.078431	0.2802862	1	3891	tags=22%, list=18%, signal=27%
COULOUARN_TEMPORAL_TGFB1_SIGNATURE_UP	COULOUARN_TEMPORAL_TGFB1_SIGNATURE_UP		84	-0.3240051	-1.2766917	0.072727	0.2806282	1	5341	tags=31%, list=24%, signal=41%
JIANG_TIP30_TARGETS_UP	JIANG_TIP30_TARGETS_UP		41	-0.3914138	-1.2758199	0.137931	0.28173056	1	3882	tags=29%, list=18%, signal=35%
THUM_SYSTOLIC_HEART_FAILURE_UP	THUM_SYSTOLIC_HEART_FAILURE_UP		377	-0.2680234	-1.275545	0.014085	0.28191605	1	4010	tags=21%, list=18%, signal=26%

REACTOME_REGULATION_OF_APC_ACTIVATORS_BETWEEN	REACTOME_REGULATION_OF_APC_ACTIVATORS_BETWEEN_G1_S		69	-0.340773	-1.27524	0.116667	0.2821825	1	3678	tags=23%, list=17%, signal=28%
LOPEZ_EPITHELIOID_MESOTHELIOMA	LOPEZ_EPITHELIOID_MESOTHELIOMA		17	-0.4721843	-1.2749141	0.203704	0.28253785	1	2571	tags=29%, list=12%, signal=33%
GRADE_COLON_AND_RECTAL_CANCER_UP	GRADE_COLON_AND_RECTAL_CANCER_UP		215	-0.288529	-1.2747204	0.032787	0.28251937	1	5980	tags=32%, list=27%, signal=43%
WOOD_EBV_EBNA1_TARGETS_DN	WOOD_EBV_EBNA1_TARGETS_DN		40	-0.3585128	-1.2746434	0.101695	0.282298	1	5087	tags=35%, list=23%, signal=45%
HELLEBREKERS_SILENCED_DURING_TUMOR_ANGIOGENES	HELLEBREKERS_SILENCED_DURING_TUMOR_ANGIOGENESIS		54	-0.3621884	-1.274085	0.105263	0.2827507	1	4740	tags=31%, list=22%, signal=40%
REACTOME_PHASE_1_FUNCTIONALIZATION_OF_COMPOUN	REACTOME_PHASE_1_FUNCTIONALIZATION_OF_COMPOUNDS		59	-0.3443672	-1.27361	0.113208	0.283255	1	2250	tags=22%, list=10%, signal=24%
KEGG_BASAL_CELL_CARCINOMA	KEGG_BASAL_CELL_CARCINOMA		55	-0.349116	-1.2731613	0.061224	0.2836541	1	5356	tags=44%, list=24%, signal=58%
KEGG_RENAL_CELL_CARCINOMA	KEGG_RENAL_CELL_CARCINOMA		70	-0.3409561	-1.2731477	0.114754	0.28333208	1	4961	tags=30%, list=23%, signal=39%
TONKS_TARGETS_OF_RUNX1_RUNX1T1_FUSION_SUSTAIN	TONKS_TARGETS_OF_RUNX1_RUNX1T1_FUSION_SUSTAINDED_		41	-0.3904948	-1.272188	0.15	0.28482118	1	5633	tags=41%, list=26%, signal=56%
FRIDMAN_SENESCENCE_UP	FRIDMAN_SENESCENCE_UP		76	-0.3251492	-1.271854	0.09434	0.2850334	1	2676	tags=18%, list=12%, signal=21%
ELVIDGE_HYPOXIA_BY_DMOG_UP	ELVIDGE_HYPOXIA_BY_DMOG_UP		126	-0.3007344	-1.2715315	0.096774	0.2851272	1	4329	tags=23%, list=20%, signal=29%
DELYS_THYROID_CANCER_DN	DELYS_THYROID_CANCER_DN		201	-0.2890478	-1.2714657	0.061538	0.284822	1	3954	tags=22%, list=18%, signal=26%
VERRECCHIA_RESPONSE_TO_TGFB1_C5	VERRECCHIA_RESPONSE_TO_TGFB1_C5		20	-0.4528352	-1.2712766	0.188679	0.28469846	1	5218	tags=40%, list=24%, signal=52%
DEURIG_T_CELL_PROLYMPHOCYTIC_LEUKEMIA_UP	DEURIG_T_CELL_PROLYMPHOCYTIC_LEUKEMIA_UP		337	-0.2733051	-1.2712516	0.026667	0.28441072	1	4350	tags=24%, list=20%, signal=30%
MULLIGHAN_MLL_SIGNATURE_1_DN	MULLIGHAN_MLL_SIGNATURE_1_DN		223	-0.2882529	-1.2710643	0.070175	0.28445858	1	5083	tags=29%, list=23%, signal=37%
REACTOME_SIGNALLING_TO_RAS	REACTOME_SIGNALLING_TO_RAS		26	-0.399707	-1.2706594	0.16129	0.28482056	1	6090	tags=54%, list=28%, signal=74%
FOSTER_INFLAMMATORY_RESPONSE_LPS_DN	FOSTER_INFLAMMATORY_RESPONSE_LPS_DN		381	-0.2698329	-1.2703556	0.066667	0.28484654	1	4838	tags=24%, list=22%, signal=30%
HOFFMANN_IMMATURE_TO_MATURE_B_LYMPHOCYTE_DN	HOFFMANN_IMMATURE_TO_MATURE_B_LYMPHOCYTE_DN		30	-0.3825094	-1.2701738	0.227273	0.28482622	1	4803	tags=33%, list=22%, signal=43%
BIOCARTA_INTEGRIN_PATHWAY	BIOCARTA_INTEGRIN_PATHWAY		38	-0.3736136	-1.2694845	0.136364	0.28578034	1	5330	tags=45%, list=24%, signal=59%
RAMASWAMY_METASTASIS_DN	RAMASWAMY_METASTASIS_DN		57	-0.360797	-1.2687652	0.142857	0.28680953	1	3605	tags=28%, list=16%, signal=34%
TURASHVILI_BREAST_LOBULAR_CARCINOMA_VS_DUCTAL	TURASHVILI_BREAST_LOBULAR_CARCINOMA_VS_DUCTAL_NORM		85	-0.3221188	-1.26802	0.166667	0.2877272	1	3845	tags=24%, list=18%, signal=28%
FERRANDO_TAL1_NEIGHBORS	FERRANDO_TAL1_NEIGHBORS		16	-0.4927893	-1.2679981	0.121212	0.28742206	1	2692	tags=25%, list=12%, signal=28%
WEST_ADRENOCORTICAL_TUMOR_MARKERS_DN	WEST_ADRENOCORTICAL_TUMOR_MARKERS_DN		19	-0.4485069	-1.2677441	0.18	0.28735	1	2433	tags=37%, list=11%, signal=41%
SU_THYMUS	SU_THYMUS		17	-0.4536502	-1.26761	0.245614	0.28721017	1	4484	tags=47%, list=20%, signal=59%
HASLINGER_B_CLL_WITH_13Q14_DELETION	HASLINGER_B_CLL_WITH_13Q14_DELETION		17	-0.4349833	-1.2676039	0.18	0.28690618	1	5375	tags=47%, list=25%, signal=62%
SABATES_COLORECTAL_ADENOMA_DN	SABATES_COLORECTAL_ADENOMA_DN		260	-0.2807357	-1.2673476	0.047619	0.28694636	1	2180	tags=16%, list=10%, signal=17%
CAIRO_HEPATOBLASTOMA_DN	CAIRO_HEPATOBLASTOMA_DN		255	-0.283618	-1.267078	0.016949	0.2870705	1	4838	tags=28%, list=22%, signal=36%
LI_WILMS_TUMOR_VS_FETAL_KIDNEY_1_UP	LI_WILMS_TUMOR_VS_FETAL_KIDNEY_1_UP		176	-0.2924954	-1.2664412	0.078125	0.28775257	1	6368	tags=39%, list=29%, signal=55%
REACTOME_CYCLIN_E_ASSOCIATED_EVENTS_DURING_G1	REACTOME_CYCLIN_E_ASSOCIATED_EVENTS_DURING_G1_S_TR		57	-0.3496284	-1.2638223	0.1875	0.29169074	1	6215	tags=39%, list=28%, signal=54%
BROWNE_HCMV_INFECTION_14HR_DN	BROWNE_HCMV_INFECTION_14HR_DN		283	-0.2690668	-1.263366	0.015625	0.29221538	1	4206	tags=25%, list=19%, signal=30%
VALK_AML_WITH_11Q23_REARRANGED	VALK_AML_WITH_11Q23_REARRANGED		21	-0.4445531	-1.2633629	0.207547	0.29187557	1	2383	tags=24%, list=11%, signal=27%
REACTOME_GLUCONEOGENESIS	REACTOME_GLUCONEOGENESIS		31	-0.3984448	-1.2627914	0.172414	0.2926573	1	3769	tags=32%, list=17%, signal=39%
RASHI_RESPONSE_TO_IONIZING_RADIATION_1	RASHI_RESPONSE_TO_IONIZING_RADIATION_1		38	-0.3811998	-1.262743	0.08	0.29239857	1	2363	tags=18%, list=11%, signal=21%
BOYLAN_MULTIPLE_MYELOMA_D_CLUSTER_DN	BOYLAN_MULTIPLE_MYELOMA_D_CLUSTER_DN		37	-0.3777851	-1.261481	0.081967	0.29421043	1	4764	tags=30%, list=22%, signal=38%
RHEIN_ALL_GLUCOCORTICOID_THERAPY_DN	RHEIN_ALL_GLUCOCORTICOID_THERAPY_DN		354	-0.2715225	-1.2608986	0.068493	0.29469553	1	5470	tags=27%, list=25%, signal=35%
SCHEIDEREIT_IKK_INTERACTING_PROTEINS	SCHEIDEREIT_IKK_INTERACTING_PROTEINS		25	-0.4052677	-1.2603649	0.150943	0.29529846	1	6434	tags=40%, list=29%, signal=57%
SCIAN_INVERSED_TARGETS_OF_TP53_AND_TP73_DN	SCIAN_INVERSED_TARGETS_OF_TP53_AND_TP73_DN		26	-0.3919272	-1.26034	0.114754	0.29500562	1	6659	tags=50%, list=30%, signal=72%
BOYLAN_MULTIPLE_MYELOMA_PCA1_UP	BOYLAN_MULTIPLE_MYELOMA_PCA1_UP		100	-0.3155828	-1.2596463	0.114286	0.2956844	1	2216	tags=21%, list=10%, signal=23%
AIYAR_COBRA1_TARGETS_UP	AIYAR_COBRA1_TARGETS_UP		37	-0.3811334	-1.2579134	0.113208	0.2984195	1	1896	tags=14%, list=9%, signal=15%
IWANAGA_CARCINOGENESIS_BY_KRAS_PTEN_DN	IWANAGA_CARCINOGENESIS_BY_KRAS_PTEN_DN		326	-0.2656232	-1.2578628	0.047619	0.29818958	1	3083	tags=19%, list=14%, signal=21%
STEIN_ESR1_TARGETS	STEIN_ESR1_TARGETS		83	-0.3162818	-1.2561672	0.112903	0.30067727	1	6002	tags=37%, list=27%, signal=51%
NIKOLSKY_BREAST_CANCER_22Q13_AMPLICON	NIKOLSKY_BREAST_CANCER_22Q13_AMPLICON		15	-0.4849493	-1.2547557	0.12069	0.30298525	1	5816	tags=53%, list=27%, signal=73%
SHIPP_DLBCL_VS_FOLLICULAR_LYMPHOMA_UP	SHIPP_DLBCL_VS_FOLLICULAR_LYMPHOMA_UP		45	-0.3595027	-1.2546467	0.133333	0.3029118	1	1201	tags=11%, list=5%, signal=12%
GOLDRATH_IMMUNE_MEMORY	GOLDRATH_IMMUNE_MEMORY		57	-0.3450341	-1.2544025	0.130435	0.30308247	1	7694	tags=49%, list=35%, signal=75%
KEGG_ALLOGRAFT_REJECTION	KEGG_ALLOGRAFT_REJECTION		29	-0.4090883	-1.2539539	0.173077	0.30360124	1	3103	tags=28%, list=14%, signal=32%
BOGNI_TREATMENT_RELATED_MYELOID_LEUKEMIA_UP	BOGNI_TREATMENT_RELATED_MYELOID_LEUKEMIA_UP		28	-0.3863797	-1.2520052	0.125	0.30671808	1	2932	tags=25%, list=13%, signal=29%
REACTOME_CONVERSION_FROM_APC_CDC20_TO_APC_CD	REACTOME_CONVERSION_FROM_APC_CDC20_TO_APC_CDH1_IN		17	-0.4629164	-1.2519467	0.178571	0.3064468	1	1116	tags=18%, list=5%, signal=19%
REACTOME_SCF_SKP2_MEDIATED_DEGRADATION_OF_P27	REACTOME_SCF_SKP2_MEDIATED_DEGRADATION_OF_P27_P21		51	-0.3448517	-1.2516648	0.125	0.30659273	1	6834	tags=41%, list=31%, signal=60%
SIG_INSULIN_RECEPTOR_PATHWAY_IN_CARDIAC_MYOCYT	SIG_INSULIN_RECEPTOR_PATHWAY_IN_CARDIAC_MYOCYTES		49	-0.3400022	-1.2502744	0.114754	0.30869627	1	5837	tags=39%, list=27%, signal=53%
ZHANG_BREAST_CANCER_PROGENITORS_DN	ZHANG_BREAST_CANCER_PROGENITORS_DN		138	-0.3029249	-1.2499944	0.115942	0.30883917	1	5251	tags=30%, list=24%, signal=40%
LIU_PROSTATE_CANCER_UP	LIU_PROSTATE_CANCER_UP		88	-0.3146925	-1.2496232	0.148148	0.30906457	1	4928	tags=26%, list=22%, signal=34%
LASTOWSKA_NEUROBLASTOMA_COPY_NUMBER_UP	LASTOWSKA_NEUROBLASTOMA_COPY_NUMBER_UP		169	-0.3002352	-1.2494876	0.098361	0.30893865	1	3951	tags=21%, list=18%, signal=26%
AMIT_EGF_RESPONSE_240_MCF10A	AMIT_EGF_RESPONSE_240_MCF10A		19	-0.4534904	-1.2493482	0.173913	0.30881009	1	4072	tags=37%, list=19%, signal=45%
DAZARD_RESPONSE_TO_UV_SCC_UP	DAZARD_RESPONSE_TO_UV_SCC_UP		80	-0.3171689	-1.2491509	0.087719	0.3086513	1	1896	tags=15%, list=9%, signal=16%
OLSSON_E2F3_TARGETS_DN	OLSSON_E2F3_TARGETS_DN		25	-0.4257245	-1.2488059	0.145455	0.308699	1	3064	tags=36%, list=14%, signal=42%
SPIRA_SMOKERS_LUNG_CANCER_UP	SPIRA_SMOKERS_LUNG_CANCER_UP		37	-0.3778922	-1.2484661	0.150943	0.30890706	1	5	tags=3%, list=0%, signal=3%
KEGG_BLADDER_CANCER	KEGG_BLADDER_CANCER		41	-0.37468	-1.2473556	0.163265	0.31073073	1	3688	tags=34%, list=17%, signal=41%
REACTOME_PYRUVATE_METABOLISM_AND_TCA_CYCLE	REACTOME_PYRUVATE_METABOLISM_AND_TCA_CYCLE		36	-0.372027	-1.247303	0.132075	0.31050712	1	5961	tags=42%, list=27%, signal=57%
BIOCARTA_RHO_PATHWAY	BIOCARTA_RHO_PATHWAY		32	-0.386658	-1.2458163	0.196721	0.31274843	1	7494	tags=53%, list=34%, signal=81%
BHATI_G2M_ARREST_BY_2METHOXYESTRADIOL_UP	BHATI_G2M_ARREST_BY_2METHOXYESTRADIOL_UP		110	-0.3106532	-1.2453933	0.12069	0.31315443	1	4508	tags=28%, list=21%, signal=35%
VALK_AML_WITH_FLT3_ITD	VALK_AML_WITH_FLT3_ITD		36	-0.3694907	-1.2440993	0.209677	0.31494963	1	3079	tags=25%, list=14%, signal=29%
HELLER_HDAC_TARGETS_SILENCED_BY_METHYLATION_UP	HELLER_HDAC_TARGETS_SILENCED_BY_METHYLATION_UP		361	-0.2632967	-1.2426395	0.047619	0.31716645	1	4617	tags=27%, list=21%, signal=33%
GRAHAM_CML_DIVIDING_VS_NORMAL_QUIESCENT_DN	GRAHAM_CML_DIVIDING_VS_NORMAL_QUIESCENT_DN		81	-0.3312689	-1.2423811	0.181818	0.31723663	1	5470	tags=33%, list=25%, signal=44%
KEGG_ACUTE_MYELOID_LEUKEMIA	KEGG_ACUTE_MYELOID_LEUKEMIA		57	-0.3484002	-1.2417622	0.109091	0.31801605	1	2151	tags=19%, list=10%, signal=21%

BREDEMEYER_RAG_SIGNALING_VIA_ATM_NOT_VIA_NFKB	BREDEMEYER_RAG_SIGNALING_VIA_ATM_NOT_VIA_NFKB_DN		38	-0.3654379	-1.2410715	0.09375	0.3187643	1	4031	tags=34%, list=18%, signal=42%
NIKOLSKY_BREAST_CANCER_20Q11_AMPLICON	NIKOLSKY_BREAST_CANCER_20Q11_AMPLICON		28	-0.3980024	-1.240406	0.2	0.3195551	1	4541	tags=29%, list=21%, signal=36%
SASSON_RESPONSE_TO_FORSKOLIN_DN	SASSON_RESPONSE_TO_FORSKOLIN_DN		65	-0.3233666	-1.2375301	0.098361	0.32442	1	4922	tags=35%, list=22%, signal=45%
MORI_LARGE_PRE_BII_LYMPHOCYTE_DN	MORI_LARGE_PRE_BII_LYMPHOCYTE_DN		45	-0.3424629	-1.2370822	0.18	0.32488623	1	5468	tags=38%, list=25%, signal=50%
WOO_LIVER_CANCER_RECURRENCE_UP	WOO_LIVER_CANCER_RECURRENCE_UP		101	-0.3042041	-1.2362624	0.119403	0.32611805	1	5527	tags=32%, list=25%, signal=42%
PUJANA_BRCA_CENTERED_NETWORK	PUJANA_BRCA_CENTERED_NETWORK		93	-0.3129084	-1.2354212	0.072727	0.32723975	1	6683	tags=35%, list=30%, signal=51%
REACTOME_G_ALPHA_I_SIGNALLING_EVENTS	REACTOME_G_ALPHA_I_SIGNALLING_EVENTS		161	-0.285563	-1.2338932	0.127273	0.3296357	1	4097	tags=24%, list=19%, signal=29%
RICKMAN_HEAD_AND_NECK_CANCER_D	RICKMAN_HEAD_AND_NECK_CANCER_D		28	-0.394555	-1.2337537	0.214286	0.3295166	1	6561	tags=36%, list=30%, signal=51%
WALLACE_PROSTATE_CANCER_RACE_UP	WALLACE_PROSTATE_CANCER_RACE_UP		254	-0.2793409	-1.2332766	0.066667	0.33006704	1	4750	tags=30%, list=22%, signal=38%
PETRETTO_HEART_MASS_QTL_CIS_UP	PETRETTO_HEART_MASS_QTL_CIS_UP		23	-0.4119026	-1.2332524	0.176471	0.3297168	1	5779	tags=48%, list=26%, signal=65%
NIKOLSKY_BREAST_CANCER_12Q24_AMPLICON	NIKOLSKY_BREAST_CANCER_12Q24_AMPLICON		15	-0.4568021	-1.2327212	0.190476	0.33039278	1	6535	tags=47%, list=30%, signal=66%
YU_MYC_TARGETS_DN	YU_MYC_TARGETS_DN		46	-0.3338817	-1.2326638	0.1	0.33019763	1	5211	tags=37%, list=24%, signal=48%
CROONQUIST_NRAS_SIGNALING_UP	CROONQUIST_NRAS_SIGNALING_UP		28	-0.3768184	-1.2325946	0.137255	0.32995802	1	6250	tags=43%, list=29%, signal=60%
TSENG_IRS1_TARGETS_UP	TSENG_IRS1_TARGETS_UP		111	-0.3068214	-1.2320384	0.131148	0.3305569	1	5008	tags=30%, list=23%, signal=38%
BLUM_RESPONSE_TO_SALIRASIB_UP	BLUM_RESPONSE_TO_SALIRASIB_UP		239	-0.271241	-1.2317466	0.057971	0.33074868	1	6310	tags=33%, list=29%, signal=46%
BIOCARTA_TID_PATHWAY	BIOCARTA_TID_PATHWAY		19	-0.4277134	-1.2316196	0.20339	0.33052376	1	4360	tags=37%, list=20%, signal=46%
WONG_MITOCHONDRIA_GENE_MODULE	WONG_MITOCHONDRIA_GENE_MODULE		205	-0.2812344	-1.2312918	0.112903	0.33091626	1	7207	tags=42%, list=33%, signal=63%
WEINMANN_ADAPTATION_TO_HYPOXIA_UP	WEINMANN_ADAPTATION_TO_HYPOXIA_UP		24	-0.403446	-1.2308233	0.236364	0.331353	1	3198	tags=25%, list=15%, signal=29%
BIOCARTA_TOLL_PATHWAY	BIOCARTA_TOLL_PATHWAY		34	-0.3808802	-1.2301073	0.222222	0.33243328	1	4171	tags=29%, list=19%, signal=36%
BIOCARTA_UCALPAIN_PATHWAY	BIOCARTA_UCALPAIN_PATHWAY		18	-0.4458786	-1.2300485	0.16	0.33212972	1	3497	tags=39%, list=16%, signal=46%
REACTOME_GLYCOLYSIS	REACTOME_GLYCOLYSIS		22	-0.4243943	-1.229883	0.14	0.332058	1	2046	tags=23%, list=9%, signal=25%
HENDRICKS_SMARCA4_TARGETS_UP	HENDRICKS_SMARCA4_TARGETS_UP		43	-0.3589268	-1.2297314	0.163265	0.33206162	1	5592	tags=37%, list=26%, signal=50%
KEGG_FC_GAMMA_R_MEDIATED_PHAGOCYTOSIS	KEGG_FC_GAMMA_R_MEDIATED_PHAGOCYTOSIS		93	-0.3042214	-1.2293564	0.169811	0.33234164	1	4961	tags=32%, list=23%, signal=42%
TAVOR_CEBPA_TARGETS_DN	TAVOR_CEBPA_TARGETS_DN		23	-0.4247949	-1.2292342	0.25	0.33225736	1	5495	tags=48%, list=25%, signal=64%
FRIDMAN_IMMORTALIZATION_DN	FRIDMAN_IMMORTALIZATION_DN		32	-0.3797379	-1.2274345	0.189189	0.33506817	1	2619	tags=25%, list=12%, signal=28%
REACTOME_PD1_SIGNALING	REACTOME_PD1_SIGNALING		19	-0.4135685	-1.2273436	0.222222	0.33490333	1	3182	tags=32%, list=15%, signal=37%
WALLACE_JAK2_TARGETS_UP	WALLACE_JAK2_TARGETS_UP		21	-0.4473478	-1.2261783	0.24	0.33665192	1	6766	tags=52%, list=31%, signal=76%
KEGG_TOLL_LIKE_RECEPTOR_SIGNALING_PATHWAY	KEGG_TOLL_LIKE_RECEPTOR_SIGNALING_PATHWAY		92	-0.2916084	-1.2242553	0.140351	0.33994973	1	5102	tags=32%, list=23%, signal=41%
REACTOME_SYNTHESIS_AND_INTERCONVERSION_OF_NUC	REACTOME_SYNTHESIS_AND_INTERCONVERSION_OF_NUCLEOT		18	-0.431139	-1.2236415	0.178571	0.34090516	1	6842	tags=56%, list=31%, signal=81%
KEGG_JAK_STAT_SIGNALING_PATHWAY	KEGG_JAK_STAT_SIGNALING_PATHWAY		143	-0.2877596	-1.223103	0.142857	0.34170938	1	3393	tags=22%, list=15%, signal=26%
MELLMAN_TUT1_TARGETS_DN	MELLMAN_TUT1_TARGETS_DN		46	-0.3588021	-1.2227333	0.224138	0.3420367	1	3970	tags=28%, list=18%, signal=34%
REACTOME_ELECTRON_TRANSPORT_CHAIN	REACTOME_ELECTRON_TRANSPORT_CHAIN		68	-0.3243198	-1.2227148	0.151515	0.34169695	1	7242	tags=47%, list=33%, signal=70%
NATSUME_RESPONSE_TO_INTERFERON_BETA_DN	NATSUME_RESPONSE_TO_INTERFERON_BETA_DN		47	-0.3810349	-1.2219646	0.188679	0.3427635	1	2947	tags=21%, list=13%, signal=25%
PRAMOONJAGO_SOX4_TARGETS_DN	PRAMOONJAGO_SOX4_TARGETS_DN		49	-0.3444372	-1.2209109	0.169811	0.34448034	1	4621	tags=37%, list=21%, signal=46%
CHIANG_LIVER_CANCER_SUBCLASS_CTNNB1_UP	CHIANG_LIVER_CANCER_SUBCLASS_CTNNB1_UP		133	-0.2965688	-1.2206787	0.12069	0.34451917	1	4189	tags=25%, list=19%, signal=30%
REACTOME_CITRIC_ACID_CYCLE	REACTOME_CITRIC_ACID_CYCLE		19	-0.4368266	-1.220349	0.175439	0.34466073	1	6595	tags=58%, list=30%, signal=83%
MUNSHI_MULTIPLE_MYELOMA_UP	MUNSHI_MULTIPLE_MYELOMA_UP		53	-0.3561922	-1.2200198	0.206897	0.34488046	1	4686	tags=36%, list=21%, signal=45%
TURASHVILI_BREAST_DUCTAL_CARCINOMA_VS_DUCTAL_N	TURASHVILI_BREAST_DUCTAL_CARCINOMA_VS_DUCTAL_NORM		44	-0.3620566	-1.2192514	0.213115	0.34612185	1	3867	tags=27%, list=18%, signal=33%
ZHAN_EARLY_DIFFERENTIATION_GENES_DN	ZHAN_EARLY_DIFFERENTIATION_GENES_DN		37	-0.3674082	-1.2191756	0.177419	0.3459304	1	7193	tags=62%, list=33%, signal=92%
ST_JNK_MAPK_PATHWAY	ST_JNK_MAPK_PATHWAY		38	-0.354508	-1.218864	0.163934	0.34603676	1	3219	tags=29%, list=15%, signal=34%
YAGI_AML_WITH_T_8_21_TRANSLOCATION	YAGI_AML_WITH_T_8_21_TRANSLOCATION		340	-0.2580429	-1.2187102	0.061538	0.34598094	1	4941	tags=26%, list=23%, signal=33%
REACTOME_CELL_CELL_ADHESION_SYSTEMS	REACTOME_CELL_CELL_ADHESION_SYSTEMS		59	-0.3308104	-1.2180139	0.122807	0.34703666	1	3164	tags=27%, list=14%, signal=32%
REACTOME_BRANCHED_CHAIN_AMINO_ACID_CATABOLISM	REACTOME_BRANCHED_CHAIN_AMINO_ACID_CATABOLISM		17	-0.4708636	-1.2180037	0.183333	0.3466804	1	4621	tags=41%, list=21%, signal=52%
KEGG_PROXIMAL_TUBULE_BICARBONATE_RECLAMATION	KEGG_PROXIMAL_TUBULE_BICARBONATE_RECLAMATION		22	-0.3808825	-1.2167361	0.176471	0.34875536	1	2570	tags=27%, list=12%, signal=31%
UDAYAKUMAR_MED1_TARGETS_UP	UDAYAKUMAR_MED1_TARGETS_UP		129	-0.2811488	-1.2164214	0.087719	0.34902647	1	4932	tags=25%, list=22%, signal=32%
BREDEMEYER_RAG_SIGNALING_NOT_VIA_ATM_DN	BREDEMEYER_RAG_SIGNALING_NOT_VIA_ATM_DN		60	-0.3217086	-1.216243	0.122807	0.34898221	1	4723	tags=32%, list=22%, signal=40%
REACTOME_PYRIMIDINE_METABOLISM	REACTOME_PYRIMIDINE_METABOLISM		22	-0.3926465	-1.2162073	0.218182	0.34867057	1	6322	tags=55%, list=29%, signal=77%
FONTAINE_PAPILLARY_THYROID_CARCINOMA_DN	FONTAINE_PAPILLARY_THYROID_CARCINOMA_DN		65	-0.3287308	-1.2160187	0.192982	0.3485097	1	2999	tags=23%, list=14%, signal=27%
REACTOME_GLUTATHIONE_CONJUGATION	REACTOME_GLUTATHIONE_CONJUGATION		16	-0.4465374	-1.2158581	0.258621	0.34840813	1	4125	tags=44%, list=19%, signal=54%
FULCHER_INFLAMMATORY_RESPONSE_LECTIN_VS_LPS_DN	FULCHER_INFLAMMATORY_RESPONSE_LECTIN_VS_LPS_DN		409	-0.250178	-1.215857	0.014286	0.34803864	1	4950	tags=25%, list=23%, signal=32%
KEGG_LINOLEIC_ACID_METABOLISM	KEGG_LINOLEIC_ACID_METABOLISM		26	-0.3774244	-1.2155536	0.244898	0.3482055	1	3275	tags=19%, list=15%, signal=23%
KESHELAVA_MULTIPLE_DRUG_RESISTANCE	KESHELAVA_MULTIPLE_DRUG_RESISTANCE		78	-0.3181054	-1.2151886	0.172414	0.3482682	1	6428	tags=38%, list=29%, signal=54%
KEGG_PROGESTERONE_MEDIATED_OOCYTE_MATURATION	KEGG_PROGESTERONE_MEDIATED_OOCYTE_MATURATION		82	-0.3014401	-1.2148414	0.148148	0.3484964	1	2669	tags=20%, list=12%, signal=22%
CHAUHAN_RESPONSE_TO_METHOXYESTRADIOL_DN	CHAUHAN_RESPONSE_TO_METHOXYESTRADIOL_DN		97	-0.2972588	-1.2134392	0.166667	0.35073963	1	6392	tags=34%, list=29%, signal=48%
REACTOME_PHOSPHORYLATION_OF_CD3_AND_TCR_ZETA	REACTOME_PHOSPHORYLATION_OF_CD3_AND_TCR_ZETA_CHAI		16	-0.4157262	-1.2133971	0.211538	0.3505326	1	3182	tags=38%, list=15%, signal=44%
REACTOME_CTLA4_INHIBITORY_SIGNALING	REACTOME_CTLA4_INHIBITORY_SIGNALING		21	-0.4190523	-1.212769	0.216667	0.35137132	1	2266	tags=24%, list=10%, signal=27%
NIKOLSKY_BREAST_CANCER_7Q21_Q22_AMPLICON	NIKOLSKY_BREAST_CANCER_7Q21_Q22_AMPLICON		66	-0.3239316	-1.2125214	0.142857	0.35141763	1	4650	tags=32%, list=21%, signal=40%
ZHAN_MULTIPLE_MYELOMA_CD1_UP	ZHAN_MULTIPLE_MYELOMA_CD1_UP		41	-0.3623712	-1.211676	0.2	0.35302144	1	4924	tags=34%, list=22%, signal=44%
HSIAO_LIVER_SPECIFIC_GENES	HSIAO_LIVER_SPECIFIC_GENES		221	-0.2570766	-1.2113206	0.075758	0.35331318	1	4713	tags=24%, list=21%, signal=31%
BROWNE_HCMV_INFECTION_30MIN_UP	BROWNE_HCMV_INFECTION_30MIN_UP		48	-0.3314321	-1.2103484	0.125	0.35473645	1	4780	tags=29%, list=22%, signal=37%
ZHU_CMV_ALL_DN	ZHU_CMV_ALL_DN		71	-0.3370166	-1.210303	0.136364	0.35445258	1	4073	tags=31%, list=19%, signal=38%
PASQUALUCCI_LYMPHOMA_BY_GC_STAGE_DN	PASQUALUCCI_LYMPHOMA_BY_GC_STAGE_DN		152	-0.291152	-1.2101218	0.098039	0.35440445	1	2881	tags=20%, list=13%, signal=23%
NEWMAN_ERCC6_TARGETS_DN	NEWMAN_ERCC6_TARGETS_DN		33	-0.3676757	-1.2100506	0.169492	0.3541964	1	4887	tags=30%, list=22%, signal=39%
BRUECKNER_TARGETS_OF_MIRLET7A3_DN	BRUECKNER_TARGETS_OF_MIRLET7A3_DN		70	-0.3216757	-1.2097951	0.166667	0.35435557	1	7402	tags=43%, list=34%, signal=64%

BIOCARTA_NO1_PATHWAY	BIOCARTA_NO1_PATHWAY		29	-0.3731668	-1.2094966	0.177778	0.35449785	1	1373	tags=21%, list=6%, signal=22%
YAGI_AML_FAB_MARKERS	YAGI_AML_FAB_MARKERS		184	-0.2720724	-1.2086369	0.083333	0.35585737	1	6285	tags=34%, list=29%, signal=47%
BIOCARTA_ACH_PATHWAY	BIOCARTA_ACH_PATHWAY		16	-0.446258	-1.2083237	0.253968	0.35608676	1	5725	tags=44%, list=26%, signal=59%
GRAHAM_CML_QUIESCENT_VS_NORMAL_QUIESCENT_DN	GRAHAM_CML_QUIESCENT_VS_NORMAL_QUIESCENT_DN		38	-0.3662279	-1.2079011	0.157895	0.35648972	1	5416	tags=37%, list=25%, signal=49%
ASTIER_INTEGRIN_SIGNALING	ASTIER_INTEGRIN_SIGNALING		54	-0.3387628	-1.2073675	0.163636	0.357052	1	4760	tags=31%, list=22%, signal=40%
REACTOME_TIE2_SIGNALING	REACTOME_TIE2_SIGNALING		18	-0.4292857	-1.2065651	0.222222	0.3584046	1	4745	tags=44%, list=22%, signal=57%
BOYAULT_LIVER_CANCER_SUBCLASS_G56_DN	BOYAULT_LIVER_CANCER_SUBCLASS_G56_DN		16	-0.4795159	-1.2048858	0.266667	0.3611635	1	4239	tags=50%, list=19%, signal=62%
SASSON_RESPONSE_TO_FORSKOLIN_UP	SASSON_RESPONSE_TO_FORSKOLIN_UP		71	-0.305528	-1.2044219	0.112903	0.3616877	1	4327	tags=27%, list=20%, signal=33%
KEGG_WNT_SIGNALING_PATHWAY	KEGG_WNT_SIGNALING_PATHWAY		146	-0.2762505	-1.203894	0.074074	0.36229917	1	4318	tags=23%, list=20%, signal=28%
KOYAMA_SEMA3B_TARGETS_DN	KOYAMA_SEMA3B_TARGETS_DN		278	-0.2574912	-1.2035149	0.088608	0.3625186	1	5447	tags=31%, list=25%, signal=40%
MASRI_RESISTANCE_TO_TAMOXIFEN_AND_AROMATASE_IN	MASRI_RESISTANCE_TO_TAMOXIFEN_AND_AROMATASE_INHIBIT		19	-0.4216784	-1.2028524	0.26	0.36331844	1	1216	tags=16%, list=6%, signal=17%
KOYAMA_SEMA3B_TARGETS_UP	KOYAMA_SEMA3B_TARGETS_UP		211	-0.2701279	-1.2024174	0.083333	0.36384144	1	4041	tags=22%, list=18%, signal=26%
HOFFMANN_LARGE_TO_SMALL_PRE_BII_LYMPHOCYTE_DN	HOFFMANN_LARGE_TO_SMALL_PRE_BII_LYMPHOCYTE_DN		38	-0.3393022	-1.2021859	0.163934	0.36388767	1	3606	tags=26%, list=16%, signal=31%
KLEIN_PRIMARY_EFFUSION_LYMPHOMA_DN	KLEIN_PRIMARY_EFFUSION_LYMPHOMA_DN		55	-0.3148448	-1.2001894	0.209677	0.36740473	1	6346	tags=40%, list=29%, signal=56%
SIMBULAN_UV_RESPONSE_IMMORTALIZED_DN	SIMBULAN_UV_RESPONSE_IMMORTALIZED_DN		27	-0.4002572	-1.1985127	0.225806	0.3701011	1	4569	tags=41%, list=21%, signal=51%
TURASHVILI_BREAST_NORMAL_DUCTAL_VS_LOBULAR_UP	TURASHVILI_BREAST_NORMAL_DUCTAL_VS_LOBULAR_UP		61	-0.3329081	-1.1974932	0.132075	0.37174165	1	1774	tags=16%, list=8%, signal=18%
LINDGREN_BLADDER_CANCER_CLUSTER_2B	LINDGREN_BLADDER_CANCER_CLUSTER_2B		358	-0.2471011	-1.194856	0.047619	0.37641436	1	4180	tags=25%, list=19%, signal=31%
SHEDDEN_LUNG_CANCER_GOOD_SURVIVAL_A12	SHEDDEN_LUNG_CANCER_GOOD_SURVIVAL_A12		260	-0.2598171	-1.1947656	0.085714	0.37621343	1	2855	tags=16%, list=13%, signal=18%
ABE_VEGFA_TARGETS_30MIN	ABE_VEGFA_TARGETS_30MIN		19	-0.4176089	-1.194645	0.258065	0.376158	1	2383	tags=26%, list=11%, signal=29%
KAUFFMANN_MELANOMA_RELAPSE_UP	KAUFFMANN_MELANOMA_RELAPSE_UP		55	-0.3413764	-1.1934451	0.235294	0.3780313	1	6400	tags=35%, list=29%, signal=49%
REACTOME_INTEGRIN_CELL_SURFACE_INTERACTIONS	REACTOME_INTEGRIN_CELL_SURFACE_INTERACTIONS		80	-0.3091483	-1.1931452	0.2	0.37834463	1	2828	tags=24%, list=13%, signal=27%
BROWNE_HCMV_INFECTION_8HR_DN	BROWNE_HCMV_INFECTION_8HR_DN		45	-0.3450524	-1.1929045	0.18	0.3785023	1	4205	tags=31%, list=19%, signal=38%
DANG_REGULATED_BY_MYC_UP	DANG_REGULATED_BY_MYC_UP		65	-0.3236753	-1.1926373	0.145455	0.3784886	1	6322	tags=38%, list=29%, signal=54%
HALMOS_CEBPA_TARGETS_UP	HALMOS_CEBPA_TARGETS_UP		39	-0.3593672	-1.191945	0.258065	0.3796187	1	5822	tags=49%, list=27%, signal=66%
BIOCARTA_NFKB_PATHWAY	BIOCARTA_NFKB_PATHWAY		22	-0.4118456	-1.1911324	0.2	0.38090125	1	4177	tags=23%, list=19%, signal=28%
ROPERO_HDAC2_TARGETS	ROPERO_HDAC2_TARGETS		89	-0.300351	-1.1906295	0.119403	0.38140067	1	4388	tags=24%, list=20%, signal=29%
REACTOME_SCF_BETA_TRCP_MEDIATED_DEGRADATION_O	REACTOME_SCF_BETA_TRCP_MEDIATED_DEGRADATION_OF_EM		47	-0.3438078	-1.1901693	0.183333	0.38192615	1	3678	tags=23%, list=17%, signal=28%
RHODES_CANCER_META_SIGNATURE	RHODES_CANCER_META_SIGNATURE		51	-0.33389	-1.1900538	0.222222	0.38172504	1	6850	tags=37%, list=31%, signal=54%
RUGO_RESPONSE_TO_GAMMA_RADIATION	RUGO_RESPONSE_TO_GAMMA_RADIATION		43	-0.3286878	-1.1899748	0.218182	0.38139492	1	2673	tags=21%, list=12%, signal=24%
SUNG_METASTASIS_STROMA_DN	SUNG_METASTASIS_STROMA_DN		47	-0.3230509	-1.1894019	0.2	0.3820608	1	4838	tags=30%, list=22%, signal=38%
FONTAINE_FOLLICULAR_THYROID_ADENOMA_DN	FONTAINE_FOLLICULAR_THYROID_ADENOMA_DN		61	-0.3484342	-1.1893872	0.208333	0.3816881	1	6022	tags=41%, list=27%, signal=56%
NICK_RESPONSE_TO_PROC_TREATMENT_DN	NICK_RESPONSE_TO_PROC_TREATMENT_DN		24	-0.4095001	-1.1888207	0.258621	0.38249615	1	1486	tags=17%, list=7%, signal=18%
TOMIDA_METASTASIS_UP	TOMIDA_METASTASIS_UP		18	-0.4366862	-1.1879203	0.229508	0.38384813	1	5001	tags=28%, list=23%, signal=36%
BENPORATH_ES_CORE_NINE_CORRELATED	BENPORATH_ES_CORE_NINE_CORRELATED		98	-0.2915269	-1.187519	0.196429	0.38429925	1	6125	tags=34%, list=28%, signal=47%
REACTOME_M_G1_TRANSITION	REACTOME_M_G1_TRANSITION		61	-0.3213793	-1.1875119	0.234375	0.38395423	1	6215	tags=34%, list=28%, signal=48%
BARRIER_CANCER_RELAPSE_NORMAL_SAMPLE_UP	BARRIER_CANCER_RELAPSE_NORMAL_SAMPLE_UP		31	-0.3814436	-1.1873364	0.278689	0.38399047	1	258	tags=13%, list=1%, signal=13%
REACTOME_CLASS_B2_SECRETIN_FAMILY_RECEPTORS	REACTOME_CLASS_B2_SECRETIN_FAMILY_RECEPTORS		84	-0.2956121	-1.1850945	0.172414	0.3879873	1	5356	tags=38%, list=24%, signal=50%
REACTOME_COSTIMULATION_BY_THE_CD28_FAMILY	REACTOME_COSTIMULATION_BY_THE_CD28_FAMILY		60	-0.3282837	-1.184741	0.183333	0.38837242	1	3455	tags=23%, list=16%, signal=28%
TCGA_GLIOBLASTOMA_COPY_NUMBER_UP	TCGA_GLIOBLASTOMA_COPY_NUMBER_UP		66	-0.319036	-1.1844246	0.207547	0.3886027	1	4337	tags=29%, list=20%, signal=36%
SENGUPTA_EBNA1_ANTICORRELATED	SENGUPTA_EBNA1_ANTICORRELATED		136	-0.2810474	-1.1844	0.064516	0.38824078	1	5270	tags=32%, list=24%, signal=42%
RUTELLA_RESPONSE_TO_HGF_VS_CSF2RB_AND_IL4_DN	RUTELLA_RESPONSE_TO_HGF_VS_CSF2RB_AND_IL4_DN		234	-0.2633736	-1.1836126	0.106061	0.3894683	1	6029	tags=31%, list=27%, signal=43%
GARGALOVIC_RESPONSE_TO_OXIDIZED_PHOSPHOLIPIDS	GARGALOVIC_RESPONSE_TO_OXIDIZED_PHOSPHOLIPIDS_GREY		17	-0.4206448	-1.1834923	0.32	0.38930586	1	1284	tags=18%, list=6%, signal=19%
BOYAULT_LIVER_CANCER_SUBCLASS_G6_DN	BOYAULT_LIVER_CANCER_SUBCLASS_G6_DN		18	-0.4312213	-1.1832178	0.265306	0.38953364	1	2086	tags=22%, list=10%, signal=25%
REACTOME_SMOOTH_MUSCLE_CONTRACTION	REACTOME_SMOOTH_MUSCLE_CONTRACTION		23	-0.3925565	-1.183113	0.230769	0.38931453	1	6271	tags=43%, list=29%, signal=61%
KEGG_COMPLEMENT_AND_COAGULATION_CASCADES	KEGG_COMPLEMENT_AND_COAGULATION_CASCADES		65	-0.3180227	-1.182654	0.175439	0.38988242	1	4838	tags=32%, list=22%, signal=41%
ADDYA_ERYTHROID_DIFFERENTIATION_BY_HEMIN	ADDYA_ERYTHROID_DIFFERENTIATION_BY_HEMIN		57	-0.3246673	-1.1822964	0.16	0.390024	1	4625	tags=37%, list=21%, signal=47%
RIZKI_TUMOR_INVASIVENESS_3D_DN	RIZKI_TUMOR_INVASIVENESS_3D_DN		224	-0.2629622	-1.1817994	0.147541	0.3906403	1	4567	tags=25%, list=21%, signal=32%
REACTOME_IMMUNOREGULATORY_INTERACTIONS_BETWEE	REACTOME_IMMUNOREGULATORY_INTERACTIONS_BETWEEN_A		54	-0.3500784	-1.1809176	0.2	0.39190012	1	3894	tags=33%, list=18%, signal=40%
SCHWAB_TARGETS_OF_BMYB_I624M_DN	SCHWAB_TARGETS_OF_BMYB_I624M_DN		17	-0.416337	-1.1804142	0.25	0.39263076	1	144	tags=12%, list=1%, signal=12%
RUGO_STRESS_RESPONSE_SUBSET_H	RUGO_STRESS_RESPONSE_SUBSET_H		39	-0.3331563	-1.1801758	0.271186	0.39271486	1	3732	tags=26%, list=17%, signal=31%
FERRANDO_T_ALL_WITH_MLL_ENL_FUSION_DN	FERRANDO_T_ALL_WITH_MLL_ENL_FUSION_DN		76	-0.3033051	-1.1792887	0.148148	0.3942742	1	4263	tags=29%, list=19%, signal=36%
BIOCARTA_ACTINY_PATHWAY	BIOCARTA_ACTINY_PATHWAY		20	-0.3901294	-1.17852	0.271186	0.39555323	1	7347	tags=50%, list=34%, signal=75%
WU_SILENCED_BY_METHYLATION_IN_BLADDER_CANCER	WU_SILENCED_BY_METHYLATION_IN_BLADDER_CANCER		41	-0.3507973	-1.1777176	0.196429	0.39709428	1	5443	tags=37%, list=25%, signal=49%
HOSHIDA_LIVER_CANCER_LATE_RECURRENCE_UP	HOSHIDA_LIVER_CANCER_LATE_RECURRENCE_UP		53	-0.325436	-1.1775042	0.192982	0.39710584	1	4687	tags=36%, list=21%, signal=45%
BOYAULT_LIVER_CANCER_SUBCLASS_G3_DN	BOYAULT_LIVER_CANCER_SUBCLASS_G3_DN		51	-0.3146657	-1.1754944	0.20339	0.40080264	1	4366	tags=29%, list=20%, signal=37%
BIOCARTA_ETS_PATHWAY	BIOCARTA_ETS_PATHWAY		18	-0.4094989	-1.1748948	0.241379	0.40157184	1	4556	tags=39%, list=21%, signal=49%
AUNG_GASTRIC_CANCER	AUNG_GASTRIC_CANCER		36	-0.3468969	-1.1746607	0.224138	0.40161896	1	5603	tags=39%, list=26%, signal=52%
BASSO_CD40_SIGNALING_DN	BASSO_CD40_SIGNALING_DN		66	-0.3189058	-1.1735948	0.225806	0.4035187	1	2027	tags=17%, list=9%, signal=18%
REACTOME_TOLL_LIKE_RECEPTOR_3_CASCADE	REACTOME_TOLL_LIKE_RECEPTOR_3_CASCADE		56	-0.3148477	-1.1734862	0.206349	0.40341184	1	5102	tags=32%, list=23%, signal=42%
KEGG_MAPK_SIGNALING_PATHWAY	KEGG_MAPK_SIGNALING_PATHWAY		259	-0.2497956	-1.1733444	0.131148	0.4033189	1	5090	tags=27%, list=23%, signal=35%
REACTOME_TRKA_SIGNALLING_FROM_THE_PLASMA_MEMB	REACTOME_TRKA_SIGNALLING_FROM_THE_PLASMA_MEMBRANE		102	-0.2924931	-1.1731706	0.25	0.40329418	1	5767	tags=35%, list=26%, signal=48%
REACTOME_BIOLOGICAL_OXIDATIONS	REACTOME_BIOLOGICAL_OXIDATIONS		102	-0.30057	-1.1730589	0.169014	0.4030624	1	2829	tags=22%, list=13%, signal=25%
ZHAN_MULTIPLE_MYELOMA_CD1_VS_CD2_UP	ZHAN_MULTIPLE_MYELOMA_CD1_VS_CD2_UP		63	-0.3106833	-1.1726718	0.185185	0.40335545	1	4660	tags=30%, list=21%, signal=38%
AMIT_EGF_RESPONSE_40_HELA	AMIT_EGF_RESPONSE_40_HELA		41	-0.3387694	-1.1722227	0.216667	0.403851	1	4748	tags=29%, list=22%, signal=37%

AMIT_EGF_RESPONSE_40_MCF10A	AMIT_EGF_RESPONSE_40_MCF10A		19	-0.4003137	-1.1717772	0.288889	0.40450415	1	2181	tags=26%, list=10%, signal=29%
GAUSSMANN_MLL_AF4_FUSION_TARGETS_F_UP	GAUSSMANN_MLL_AF4_FUSION_TARGETS_F_UP		130	-0.270585	-1.1716871	0.136364	0.4043689	1	4554	tags=32%, list=21%, signal=40%
CAMPS_COLON_CANCER_COPY_NUMBER_UP	CAMPS_COLON_CANCER_COPY_NUMBER_UP		71	-0.3223884	-1.1705867	0.210526	0.40623486	1	5729	tags=42%, list=26%, signal=57%
DAIRKEE_TERT_TARGETS_DN	DAIRKEE_TERT_TARGETS_DN		85	-0.2942763	-1.1703881	0.189655	0.40619335	1	5211	tags=31%, list=24%, signal=40%
KEGG_T_CELL_RECEPTOR_SIGNALING_PATHWAY	KEGG_T_CELL_RECEPTOR_SIGNALING_PATHWAY		108	-0.2848914	-1.1699803	0.220339	0.4067298	1	4745	tags=26%, list=22%, signal=33%
CHANG_CORE_SERUM_RESPONSE_UP	CHANG_CORE_SERUM_RESPONSE_UP		66	-0.3227035	-1.1691611	0.222222	0.40818262	1	6085	tags=42%, list=28%, signal=59%
SA_B_CELL_RECEPTOR_COMPLEXES	SA_B_CELL_RECEPTOR_COMPLEXES		24	-0.3860786	-1.1688145	0.313726	0.40865895	1	4961	tags=42%, list=23%, signal=54%
PUJANA_BREAST_CANCER_WITH_BRCA1_MUTATED_UP	PUJANA_BREAST_CANCER_WITH_BRCA1_MUTATED_UP		53	-0.3394616	-1.168243	0.237288	0.40940794	1	6291	tags=36%, list=29%, signal=50%
REACTOME_GLUCOSE_REGULATION_OF_INSULIN_SECRETI	REACTOME_GLUCOSE_REGULATION_OF_INSULIN_SECRETION		151	-0.2765178	-1.168046	0.178571	0.4094606	1	6220	tags=34%, list=28%, signal=48%
JAZAG_TGFB1_SIGNALING_VIA_SMAD4_DN	JAZAG_TGFB1_SIGNALING_VIA_SMAD4_DN		59	-0.3145684	-1.1667536	0.257576	0.4116507	1	4526	tags=31%, list=21%, signal=38%
REACTOME_CAM_PATHWAY	REACTOME_CAM_PATHWAY		25	-0.3697802	-1.1663045	0.245902	0.4122189	1	5767	tags=44%, list=26%, signal=60%
REACTOME_UNFOLDED_PROTEIN_RESPONSE	REACTOME_UNFOLDED_PROTEIN_RESPONSE		19	-0.4137401	-1.1645734	0.264151	0.4156268	1	6596	tags=47%, list=30%, signal=68%
MAHADEVAN_IMATINIB_RESISTANCE_UP	MAHADEVAN_IMATINIB_RESISTANCE_UP		16	-0.3994749	-1.1644893	0.280702	0.41542813	1	3367	tags=31%, list=15%, signal=37%
BOYLAN_MULTIPLE_MYELOMA_PCA3_UP	BOYLAN_MULTIPLE_MYELOMA_PCA3_UP		41	-0.3277769	-1.1638514	0.171875	0.41636986	1	3375	tags=22%, list=15%, signal=26%
WINNEPENNINCKX_MELANOMA_METASTASIS_UP	WINNEPENNINCKX_MELANOMA_METASTASIS_UP		151	-0.2715727	-1.163166	0.245902	0.41742778	1	6839	tags=36%, list=31%, signal=53%
MENSSEN_MYC_TARGETS	MENSSEN_MYC_TARGETS		15	-0.4404308	-1.1630719	0.264151	0.41716105	1	5387	tags=53%, list=25%, signal=71%
SMID_BREAST_CANCER_RELAPSE_IN_BONE_UP	SMID_BREAST_CANCER_RELAPSE_IN_BONE_UP		88	-0.2988701	-1.1618136	0.190476	0.4191924	1	2571	tags=17%, list=12%, signal=19%
DE_YY1_TARGETS_UP	DE_YY1_TARGETS_UP		18	-0.4119417	-1.1608796	0.254545	0.42111287	1	693	tags=17%, list=3%, signal=17%
ODONNELL_TARGETS_OF_MYC_AND_TFRC_UP	ODONNELL_TARGETS_OF_MYC_AND_TFRC_UP		68	-0.3254518	-1.1606966	0.186441	0.4210966	1	2037	tags=18%, list=9%, signal=19%
MOHANKUMAR_TLX1_TARGETS_DN	MOHANKUMAR_TLX1_TARGETS_DN		141	-0.2786934	-1.1601694	0.16129	0.42187816	1	2176	tags=16%, list=10%, signal=17%
NOJIMA_SFRP2_TARGETS_DN	NOJIMA_SFRP2_TARGETS_DN		22	-0.3785217	-1.1601644	0.218182	0.42147332	1	3688	tags=36%, list=17%, signal=44%
CHIBA_RESPONSE_TO_TSA	CHIBA_RESPONSE_TO_TSA		37	-0.339721	-1.160146	0.229508	0.42108294	1	736	tags=14%, list=3%, signal=14%
BIOCARTA_STRESS_PATHWAY	BIOCARTA_STRESS_PATHWAY		25	-0.3825591	-1.159332	0.22807	0.42270836	1	828	tags=12%, list=4%, signal=12%
KORKOLA_CORRELATED_WITH_POU5F1	KORKOLA_CORRELATED_WITH_POU5F1		28	-0.3633581	-1.1588509	0.253968	0.42332608	1	3551	tags=32%, list=16%, signal=38%
KEGG_PANCREATIC_CANCER	KEGG_PANCREATIC_CANCER		70	-0.2987898	-1.158731	0.233333	0.42312264	1	3619	tags=21%, list=17%, signal=26%
JAZAG_TGFB1_SIGNALING_DN	JAZAG_TGFB1_SIGNALING_DN		32	-0.3709598	-1.1586343	0.222222	0.42291883	1	5920	tags=41%, list=27%, signal=56%
ZHOU_INFLAMMATORY_RESPONSE_LIVE_UP	ZHOU_INFLAMMATORY_RESPONSE_LIVE_UP		417	-0.2423883	-1.1582495	0.146667	0.4235082	1	4838	tags=25%, list=22%, signal=31%
ELVIDGE_HIF1A_TARGETS_DN	ELVIDGE_HIF1A_TARGETS_DN		87	-0.2978139	-1.1574078	0.213115	0.4250705	1	4941	tags=28%, list=23%, signal=35%
BASSO_HAIRY_CELL_LEUKEMIA_DN	BASSO_HAIRY_CELL_LEUKEMIA_DN		77	-0.3052785	-1.157132	0.214286	0.4252138	1	2749	tags=22%, list=13%, signal=25%
REACTOME_PLC_BETA_MEDIATED_EVENTS	REACTOME_PLC_BETA_MEDIATED_EVENTS		37	-0.3404312	-1.156997	0.296296	0.42513308	1	5767	tags=41%, list=26%, signal=55%
CHNG_MULTIPLE_MYELOMA_HYPERPLOID_DN	CHNG_MULTIPLE_MYELOMA_HYPERPLOID_DN		27	-0.3801334	-1.1568787	0.277778	0.425036	1	1383	tags=11%, list=6%, signal=12%
KEGG_GRAFT_VERSUS_HOST_DISEASE	KEGG_GRAFT_VERSUS_HOST_DISEASE		28	-0.3730434	-1.156241	0.258621	0.42594725	1	3103	tags=29%, list=14%, signal=33%
BIOCARTA_IL12_PATHWAY	BIOCARTA_IL12_PATHWAY		21	-0.3758725	-1.1560605	0.3	0.42595574	1	4508	tags=43%, list=21%, signal=54%
TONKS_TARGETS_OF_RUNX1_RUNX1T1_FUSION_GRANULC	TONKS_TARGETS_OF_RUNX1_RUNX1T1_FUSION_GRANULOCYTE		16	-0.4192576	-1.1559647	0.26	0.42576355	1	5175	tags=50%, list=24%, signal=65%
KEGG_NOD_LIKE_RECEPTOR_SIGNALING_PATHWAY	KEGG_NOD_LIKE_RECEPTOR_SIGNALING_PATHWAY		54	-0.3373308	-1.1552727	0.262295	0.42660952	1	5796	tags=35%, list=26%, signal=48%
MISHRA_CARCINOMA_ASSOCIATED_FIBROBLAST_UP	MISHRA_CARCINOMA_ASSOCIATED_FIBROBLAST_UP		20	-0.4215186	-1.1537708	0.262295	0.4295926	1	5527	tags=40%, list=25%, signal=53%
KIM_WT1_TARGETS_8HR_DN	KIM_WT1_TARGETS_8HR_DN		113	-0.2848313	-1.1532564	0.214286	0.43022004	1	6250	tags=35%, list=29%, signal=49%
DAVICIONI_TARGETS_OF_PAX_FOXO1_FUSIONS_UP	DAVICIONI_TARGETS_OF_PAX_FOXO1_FUSIONS_UP		249	-0.2543517	-1.152832	0.161765	0.4306754	1	3644	tags=18%, list=17%, signal=21%
VALK_AML_CLUSTER_4	VALK_AML_CLUSTER_4		27	-0.3936628	-1.1524324	0.288462	0.43093106	1	4965	tags=33%, list=23%, signal=43%
WOO_LIVER_CANCER_RECURRENCE_DN	WOO_LIVER_CANCER_RECURRENCE_DN		73	-0.3029127	-1.1523832	0.216667	0.43059155	1	4328	tags=26%, list=20%, signal=32%
MCBRYAN_PUBERTAL_BREAST_5_6WK_DN	MCBRYAN_PUBERTAL_BREAST_5_6WK_DN		127	-0.2781899	-1.1522183	0.206897	0.430595	1	4275	tags=20%, list=19%, signal=25%
REACTOME_CYTOSOLIC_TRNA_AMINOACYLATION	REACTOME_CYTOSOLIC_TRNA_AMINOACYLATION		23	-0.3864337	-1.1508048	0.24	0.43308762	1	6835	tags=43%, list=31%, signal=63%
PASQUALUCCI_LYMPHOMA_BY_GC_STAGE_UP	PASQUALUCCI_LYMPHOMA_BY_GC_STAGE_UP		262	-0.2509067	-1.149631	0.131148	0.43504003	1	5012	tags=27%, list=23%, signal=34%
BROWNE_HCMV_INFECTION_30MIN_DN	BROWNE_HCMV_INFECTION_30MIN_DN		141	-0.2630132	-1.1484321	0.169492	0.4370637	1	3415	tags=21%, list=16%, signal=25%
LEE_RECENT_THYMIC_EMIGRANT	LEE_RECENT_THYMIC_EMIGRANT		96	-0.2831673	-1.1483345	0.222222	0.43690428	1	4721	tags=29%, list=22%, signal=37%
CONCANNON_APOPTOSIS_BY_EPOXOMICIN_DN	CONCANNON_APOPTOSIS_BY_EPOXOMICIN_DN		162	-0.2631957	-1.1480967	0.169231	0.43685025	1	4131	tags=25%, list=19%, signal=30%
IZADPANAH_STEM_CELL_ADIPOSE_VS_BONE_DN	IZADPANAH_STEM_CELL_ADIPOSE_VS_BONE_DN		105	-0.2838234	-1.1469519	0.185185	0.43905306	1	4265	tags=28%, list=19%, signal=34%
KEGG_VEGF_SIGNALING_PATHWAY	KEGG_VEGF_SIGNALING_PATHWAY		74	-0.3053528	-1.1457336	0.210526	0.44141626	1	3318	tags=23%, list=15%, signal=27%
XU_HGF_TARGETS_REPRESSED_BY_AKT1_DN	XU_HGF_TARGETS_REPRESSED_BY_AKT1_DN		68	-0.2991538	-1.145375	0.192982	0.44183043	1	2696	tags=19%, list=12%, signal=22%
HAHTOLA_MYCOSIS_FUNGOIDES_DN	HAHTOLA_MYCOSIS_FUNGOIDES_DN		15	-0.4212198	-1.1411201	0.322034	0.4506231	1	5126	tags=33%, list=23%, signal=43%
MOOTHA_TCA	MOOTHA_TCA		15	-0.4532524	-1.1408453	0.326087	0.45075208	1	6595	tags=60%, list=30%, signal=86%
TIAN_TNF_SIGNALING_NOT_VIA_NFKB	TIAN_TNF_SIGNALING_NOT_VIA_NFKB		15	-0.4332274	-1.1407582	0.309524	0.4505806	1	7048	tags=60%, list=32%, signal=88%
KEGG_HUNTINGTONS_DISEASE	KEGG_HUNTINGTONS_DISEASE		167	-0.2617687	-1.1400869	0.171875	0.4516798	1	5204	tags=26%, list=24%, signal=34%
FINETTI_BREAST_CANCER_KINOME_GREEN	FINETTI_BREAST_CANCER_KINOME_GREEN		15	-0.4300916	-1.1396708	0.295082	0.45213693	1	2151	tags=27%, list=10%, signal=30%
BIDUS_METASTASIS_UP	BIDUS_METASTASIS_UP		207	-0.2507475	-1.1390673	0.295082	0.45303336	1	5640	tags=27%, list=26%, signal=36%
STOSSI_RESPONSE_TO_ESTRADIOL	STOSSI_RESPONSE_TO_ESTRADIOL		33	-0.3781023	-1.1386706	0.28	0.453394	1	3275	tags=24%, list=15%, signal=28%
RIGGINS_TAMOXIFEN_RESISTANCE_UP	RIGGINS_TAMOXIFEN_RESISTANCE_UP		65	-0.3025157	-1.138591	0.245614	0.45311627	1	5176	tags=37%, list=24%, signal=48%
BREUHAHN_GROWTH_FACTOR_SIGNALING_IN_LIVER_CAN	BREUHAHN_GROWTH_FACTOR_SIGNALING_IN_LIVER_CANCER		15	-0.4247312	-1.1385783	0.288462	0.4527361	1	5218	tags=40%, list=24%, signal=52%
YAGI_AML_WITH_11Q23_REARRANGED	YAGI_AML_WITH_11Q23_REARRANGED		334	-0.2363293	-1.138519	0.136364	0.45245972	1	4609	tags=25%, list=21%, signal=31%
BIOCARTA_MAPK_PATHWAY	BIOCARTA_MAPK_PATHWAY		86	-0.3118625	-1.1384993	0.266667	0.45211935	1	5090	tags=28%, list=23%, signal=36%
RUGO_UV_RESPONSE	RUGO_UV_RESPONSE		25	-0.364273	-1.1383979	0.196078	0.4519718	1	7002	tags=44%, list=32%, signal=65%
SIG_PIP3_SIGNALING_IN_B_LYMPHOCYTES	SIG_PIP3_SIGNALING_IN_B_LYMPHOCYTES		33	-0.3508933	-1.138242	0.215686	0.4518261	1	4751	tags=36%, list=22%, signal=46%
TENEDINI_MEGAKARYOCYTE_MARKERS	TENEDINI_MEGAKARYOCYTE_MARKERS		51	-0.3208056	-1.1378185	0.222222	0.45241895	1	4864	tags=31%, list=22%, signal=40%
XU_HGF_SIGNALING_NOT_VIA_AKT1_48HR_DN	XU_HGF_SIGNALING_NOT_VIA_AKT1_48HR_DN		15	-0.4358201	-1.1359073	0.327586	0.45620495	1	4331	tags=33%, list=20%, signal=42%

BIOCARTA_IL2RB_PATHWAY	BIOCARTA_IL2RB_PATHWAY		38	-0.3495163	-1.1357135	0.215686	0.4562496	1	6103	tags=37%, list=28%, signal=51%
MULLIGHAN_NPM1_MUTATED_SIGNATURE_2_DN	MULLIGHAN_NPM1_MUTATED_SIGNATURE_2_DN		72	-0.3059348	-1.1352099	0.258065	0.4568644	1	6432	tags=40%, list=29%, signal=57%
REACTOME_ADP_SIGNALLING_THROUGH_P2Y_PURINOCEP	REACTOME_ADP_SIGNALLING_THROUGH_P2Y_PURINOCEPTOR		25	-0.3510369	-1.1350433	0.238095	0.45672995	1	4073	tags=28%, list=19%, signal=34%
REACTOME_MAP_KINASES_ACTIVATION_IN_TLR_CASCADE	REACTOME_MAP_KINASES_ACTIVATION_IN_TLR_CASCADE		41	-0.3189526	-1.1338768	0.316667	0.4593012	1	1512	tags=17%, list=7%, signal=18%
WANG_LMO4_TARGETS_UP	WANG_LMO4_TARGETS_UP		328	-0.2383001	-1.133538	0.179104	0.45961565	1	2844	tags=14%, list=13%, signal=16%
LEONARD_HYPOXIA	LEONARD_HYPOXIA		34	-0.3497298	-1.1318045	0.288136	0.4630971	1	5221	tags=29%, list=24%, signal=39%
MORI_SMALL_PRE_BII_LYMPHOCYTE_UP	MORI_SMALL_PRE_BII_LYMPHOCYTE_UP		62	-0.2887429	-1.1314884	0.245614	0.463522	1	4626	tags=32%, list=21%, signal=41%
YAGI_AML_RELAPSE_PROGNOSIS	YAGI_AML_RELAPSE_PROGNOSIS		34	-0.3373585	-1.1313514	0.267857	0.4634079	1	4681	tags=32%, list=21%, signal=41%
RAMASWAMY_METASTASIS_UP	RAMASWAMY_METASTASIS_UP		64	-0.309401	-1.1312715	0.241935	0.46309996	1	3962	tags=27%, list=18%, signal=32%
NAKAYAMA_FRA2_TARGETS	NAKAYAMA_FRA2_TARGETS		39	-0.3206789	-1.1310472	0.269841	0.46326604	1	4002	tags=33%, list=18%, signal=41%
GAUSSMANN_MLL_AF4_FUSION_TARGETS_D_UP	GAUSSMANN_MLL_AF4_FUSION_TARGETS_D_UP		30	-0.3617974	-1.1310453	0.258621	0.4628562	1	7	tags=3%, list=0%, signal=3%
ST_INTERLEUKIN_4_PATHWAY	ST_INTERLEUKIN_4_PATHWAY		24	-0.3888547	-1.1303396	0.318182	0.4637543	1	2947	tags=29%, list=13%, signal=34%
SENESE_HDAC3_TARGETS_DN	SENESE_HDAC3_TARGETS_DN		495	-0.2349113	-1.1300088	0.192771	0.46407256	1	6803	tags=36%, list=31%, signal=51%
LUI_THYROID_CANCER_CLUSTER_2	LUI_THYROID_CANCER_CLUSTER_2		42	-0.326087	-1.1291834	0.31746	0.46562892	1	4680	tags=31%, list=21%, signal=39%
BIOCARTA_NKT_PATHWAY	BIOCARTA_NKT_PATHWAY		27	-0.3781787	-1.1291436	0.309091	0.46528146	1	4508	tags=37%, list=21%, signal=47%
REACTOME_CYTOCHROME_P450_ARRANGED_BY_SUBSTRA	REACTOME_CYTOCHROME_P450_ARRANGED_BY_SUBSTRATE_TY		44	-0.3196621	-1.1290542	0.321429	0.46508932	1	2080	tags=18%, list=9%, signal=20%
DITTMER_PTHLH_TARGETS_UP	DITTMER_PTHLH_TARGETS_UP		109	-0.2720862	-1.1285481	0.225806	0.46595216	1	7960	tags=50%, list=36%, signal=77%
GRAHAM_NORMAL_QUIESCENT_VS_NORMAL_DIVIDING_U	GRAHAM_NORMAL_QUIESCENT_VS_NORMAL_DIVIDING_UP		61	-0.31749	-1.1282376	0.255319	0.4660012	1	6466	tags=41%, list=29%, signal=58%
GAZDA_DIAMOND_BLACKFAN_ANEMIA_ERYTHROID_DN	GAZDA_DIAMOND_BLACKFAN_ANEMIA_ERYTHROID_DN		432	-0.2271405	-1.1276302	0.060606	0.4668377	1	4968	tags=21%, list=23%, signal=27%
ZHAN_MULTIPLE_MYELOMA_MS_UP	ZHAN_MULTIPLE_MYELOMA_MS_UP		45	-0.3262649	-1.1269511	0.254545	0.468001	1	4982	tags=27%, list=23%, signal=34%
BENPORATH_ES_2	BENPORATH_ES_2		38	-0.3482155	-1.1266915	0.254237	0.4679825	1	5908	tags=37%, list=27%, signal=50%
BIOCARTA_IGF1MTOR_PATHWAY	BIOCARTA_IGF1MTOR_PATHWAY		20	-0.3900415	-1.1266673	0.30303	0.46766087	1	1062	tags=15%, list=5%, signal=16%
SCIBETTA_KDM5B_TARGETS_UP	SCIBETTA_KDM5B_TARGETS_UP		16	-0.4208933	-1.1265464	0.333333	0.4675291	1	3977	tags=31%, list=18%, signal=38%
BREDEMEYER_RAG_SIGNALING_NOT_VIA_ATM_UP	BREDEMEYER_RAG_SIGNALING_NOT_VIA_ATM_UP		60	-0.310631	-1.1253487	0.258621	0.4693925	1	5441	tags=30%, list=25%, signal=40%
REACTOME_MYD88_CASCADE	REACTOME_MYD88_CASCADE		17	-0.3805456	-1.1250722	0.266667	0.46954986	1	3386	tags=35%, list=15%, signal=42%
UZONYI_RESPONSE_TO_LEUKOTRIENE_AND_THROMBIN	UZONYI_RESPONSE_TO_LEUKOTRIENE_AND_THROMBIN		36	-0.3320718	-1.1247967	0.311475	0.46980962	1	3200	tags=22%, list=15%, signal=26%
ENGELMANN_CANCER_PROGENITORS_UP	ENGELMANN_CANCER_PROGENITORS_UP		47	-0.3305772	-1.1240025	0.31746	0.47137094	1	4545	tags=32%, list=21%, signal=40%
HANN_RESISTANCE_TO_BCL2_INHIBITOR_UP	HANN_RESISTANCE_TO_BCL2_INHIBITOR_UP		30	-0.3474693	-1.123403	0.28125	0.47234777	1	1610	tags=20%, list=7%, signal=22%
FOURNIER_ACINAR_DEVELOPMENT_EARLY_UP	FOURNIER_ACINAR_DEVELOPMENT_EARLY_UP		21	-0.3793487	-1.1231138	0.314815	0.47256765	1	3271	tags=19%, list=15%, signal=22%
RIZ_ERYTHROID_DIFFERENTIATION_HEMGN	RIZ_ERYTHROID_DIFFERENTIATION_HEMGN		29	-0.3426228	-1.122379	0.283019	0.47381294	1	5741	tags=45%, list=26%, signal=61%
COLIN_PILOCYTIC_ASTROCYTOMA_VS_GLIOBLASTOMA_DN	COLIN_PILOCYTIC_ASTROCYTOMA_VS_GLIOBLASTOMA_DN		28	-0.3499328	-1.1218952	0.25	0.47443354	1	7540	tags=43%, list=34%, signal=65%
REACTOME_MAPK_TARGETS_NUCLEAR_EVENTS_MEDIATED	REACTOME_MAPK_TARGETS_NUCLEAR_EVENTS_MEDIATED_BY_		30	-0.3610506	-1.1217527	0.241379	0.4742983	1	925	tags=17%, list=4%, signal=17%
LEE_LIVER_CANCER_MYC_TGFA_DN	LEE_LIVER_CANCER_MYC_TGFA_DN		61	-0.3081426	-1.1215876	0.28	0.47430333	1	3028	tags=16%, list=14%, signal=19%
ST_TUMOR_NECROSIS_FACTOR_PATHWAY	ST_TUMOR_NECROSIS_FACTOR_PATHWAY		28	-0.3563881	-1.1211017	0.269231	0.4749059	1	4171	tags=25%, list=19%, signal=31%
CHEBOTAEV_GR_TARGETS_DN	CHEBOTAEV_GR_TARGETS_DN		123	-0.2680739	-1.1206216	0.25	0.47560918	1	4155	tags=24%, list=19%, signal=29%
SCHWAB_TARGETS_OF_BMYB_S427G_DN	SCHWAB_TARGETS_OF_BMYB_S427G_DN		17	-0.416337	-1.1204066	0.363636	0.47579712	1	144	tags=12%, list=1%, signal=12%
REACTOME_PLATELET_AGGREGATION_PLUG_FORMATION	REACTOME_PLATELET_AGGREGATION_PLUG_FORMATION		26	-0.3677419	-1.1202964	0.277778	0.47567323	1	6862	tags=46%, list=31%, signal=67%
NAKAMURA_METASTASIS_MODEL_UP	NAKAMURA_METASTASIS_MODEL_UP		41	-0.3209727	-1.1197699	0.32	0.4764103	1	6145	tags=37%, list=28%, signal=51%
CHENG_IMPRINTED_BY_ESTRADIOL	CHENG_IMPRINTED_BY_ESTRADIOL		100	-0.2806358	-1.1192524	0.301587	0.47737	1	4015	tags=23%, list=18%, signal=28%
VANTVEER_BREAST_CANCER_BRCA1_UP	VANTVEER_BREAST_CANCER_BRCA1_UP		31	-0.3533574	-1.118929	0.259259	0.47758526	1	2619	tags=23%, list=12%, signal=26%
KYNG_DNA_DAMAGE_BY_GAMMA_RADIATION	KYNG_DNA_DAMAGE_BY_GAMMA_RADIATION		43	-0.3286878	-1.118701	0.277778	0.47768217	1	2673	tags=21%, list=12%, signal=24%
YAGI_AML_WITH_INV_16_TRANSLOCATION	YAGI_AML_WITH_INV_16_TRANSLOCATION		395	-0.2324263	-1.118601	0.166667	0.47748235	1	3682	tags=14%, list=17%, signal=16%
BIOCARTA_ATM_PATHWAY	BIOCARTA_ATM_PATHWAY		20	-0.4042322	-1.1182705	0.315789	0.4777444	1	4741	tags=35%, list=22%, signal=45%
CHEN_HOXA5_TARGETS_9HR_DN	CHEN_HOXA5_TARGETS_9HR_DN		38	-0.3297061	-1.1181918	0.301887	0.47749415	1	4940	tags=29%, list=23%, signal=37%
ZHANG_PROLIFERATING_VS_QUIESCENT	ZHANG_PROLIFERATING_VS_QUIESCENT		48	-0.3106635	-1.1180375	0.240741	0.4774426	1	4686	tags=27%, list=21%, signal=34%
KIM_WT1_TARGETS_12HR_DN	KIM_WT1_TARGETS_12HR_DN		204	-0.2459333	-1.1179466	0.218182	0.4772303	1	4938	tags=25%, list=23%, signal=33%
KYNG_DNA_DAMAGE_BY_GAMMA_AND_UV_RADIATION	KYNG_DNA_DAMAGE_BY_GAMMA_AND_UV_RADIATION		39	-0.3331563	-1.1178415	0.327273	0.47701907	1	3732	tags=26%, list=17%, signal=31%
SANA_TNF_SIGNALING_DN	SANA_TNF_SIGNALING_DN		79	-0.2910969	-1.1174806	0.315789	0.47732827	1	4031	tags=27%, list=18%, signal=32%
PUJANA_XPRSS_INT_NETWORK	PUJANA_XPRSS_INT_NETWORK		162	-0.2553362	-1.1173228	0.258621	0.4772916	1	6683	tags=34%, list=30%, signal=48%
BONOME_OVARIAN_CANCER_SURVIVAL_SUBOPTIMAL_DEE	BONOME_OVARIAN_CANCER_SURVIVAL_SUBOPTIMAL_DEBULKI		479	-0.2279226	-1.1169852	0.136986	0.4777999	1	6298	tags=32%, list=29%, signal=44%
BROWNE_HCMV_INFECTION_6HR_UP	BROWNE_HCMV_INFECTION_6HR_UP		62	-0.2959841	-1.1167978	0.234375	0.47776267	1	3526	tags=19%, list=16%, signal=23%
BERTUCCI_MEDULLARY_VS_DUCTAL_BREAST_CANCER_DN	BERTUCCI_MEDULLARY_VS_DUCTAL_BREAST_CANCER_DN		154	-0.2562717	-1.1166167	0.2	0.4777602	1	4365	tags=23%, list=20%, signal=29%
VALK_AML_CLUSTER_15	VALK_AML_CLUSTER_15		29	-0.3698495	-1.1163545	0.315789	0.47800553	1	6145	tags=38%, list=28%, signal=53%
BROWNE_HCMV_INFECTION_20HR_DN	BROWNE_HCMV_INFECTION_20HR_DN		103	-0.2783467	-1.114863	0.234375	0.48111233	1	4785	tags=33%, list=22%, signal=42%
BOYAULT_LIVER_CANCER_SUBCLASS_G123_UP	BOYAULT_LIVER_CANCER_SUBCLASS_G123_UP		43	-0.3332658	-1.1139534	0.258621	0.48301503	1	6380	tags=44%, list=29%, signal=62%
BLALOCK_ALZHEIMERS_DISEASE_INCIPIENT_UP	BLALOCK_ALZHEIMERS_DISEASE_INCIPIENT_UP		365	-0.2366034	-1.1135617	0.19697	0.48350674	1	3963	tags=21%, list=18%, signal=25%
GENTILE_UV_RESPONSE_CLUSTER_D5	GENTILE_UV_RESPONSE_CLUSTER_D5		29	-0.3562261	-1.113361	0.297872	0.48346445	1	5411	tags=31%, list=25%, signal=41%
BILD_MYC_ONCOGENIC_SIGNATURE	BILD_MYC_ONCOGENIC_SIGNATURE		186	-0.2500598	-1.113187	0.234375	0.48338377	1	3963	tags=21%, list=18%, signal=25%
BIOCARTA_P38MAPK_PATHWAY	BIOCARTA_P38MAPK_PATHWAY		39	-0.3482769	-1.1125197	0.266667	0.48439822	1	4953	tags=31%, list=23%, signal=40%
LEE_LIVER_CANCER	LEE_LIVER_CANCER		39	-0.3140067	-1.1124791	0.28	0.48414564	1	5250	tags=33%, list=24%, signal=44%
REACTOME_DOWN_STREAM_SIGNAL_TRANSDUCTION	REACTOME_DOWN_STREAM_SIGNAL_TRANSDUCTION		35	-0.3440393	-1.1122732	0.315789	0.48426193	1	5725	tags=37%, list=26%, signal=50%
BIOCARTA_CHREBP2_PATHWAY	BIOCARTA_CHREBP2_PATHWAY		40	-0.3477535	-1.1116598	0.263158	0.485335	1	5391	tags=33%, list=25%, signal=43%
CHENG_RESPONSE_TO_NICKEL_ACETATE	CHENG_RESPONSE_TO_NICKEL_ACETATE		26	-0.3600242	-1.1110964	0.344262	0.48648852	1	3256	tags=27%, list=15%, signal=32%
YAO_TEMPORAL_RESPONSE_TO_PROGESTERONE_CLUSTER	YAO_TEMPORAL_RESPONSE_TO_PROGESTERONE_CLUSTER_0		70	-0.2983978	-1.1110649	0.233333	0.48611408	1	4016	tags=27%, list=18%, signal=33%

REACTOME_TOLL_RECEPTOR_CASCADES	REACTOME_TOLL_RECEPTOR_CASCADES		81	-0.297817	-1.1104116	0.275862	0.48703995	1	4171	tags=27%, list=19%, signal=33%
XU_CREBBP_TARGETS_UP	XU_CREBBP_TARGETS_UP		26	-0.3638474	-1.1098641	0.311475	0.48797908	1	8766	tags=62%, list=40%, signal=102%
REACTOME_ACTIVATION_OF_BH3_ONLY_PROTEINS	REACTOME_ACTIVATION_OF_BH3_ONLY_PROTEINS		16	-0.3979512	-1.1097852	0.365385	0.48776302	1	1062	tags=13%, list=5%, signal=13%
BENPORATH_MYC_TARGETS_WITH_EBOX	BENPORATH_MYC_TARGETS_WITH_EBOX		221	-0.2469177	-1.1096014	0.177419	0.48770463	1	4899	tags=24%, list=22%, signal=31%
YAO_TEMPORAL_RESPONSE_TO_PROGESTERONE_CLUSTEF	YAO_TEMPORAL_RESPONSE_TO_PROGESTERONE_CLUSTER_10		64	-0.3039707	-1.1094242	0.267857	0.4876732	1	4928	tags=30%, list=22%, signal=38%
KLEIN_PRIMARY_EFFUSION_LYMPHOMA_UP	KLEIN_PRIMARY_EFFUSION_LYMPHOMA_UP		48	-0.3081314	-1.1093208	0.35	0.48745814	1	6304	tags=35%, list=29%, signal=50%
DER_IFN_GAMMA_RESPONSE_UP	DER_IFN_GAMMA_RESPONSE_UP		55	-0.3169525	-1.1091772	0.258621	0.48735228	1	3244	tags=24%, list=15%, signal=28%
DITTMER_PTHLH_TARGETS_DN	DITTMER_PTHLH_TARGETS_DN		65	-0.3034619	-1.1078234	0.346154	0.4897467	1	2596	tags=18%, list=12%, signal=21%
BARRIER_COLON_CANCER_RECURRENCE_DN	BARRIER_COLON_CANCER_RECURRENCE_DN		19	-0.4240182	-1.107534	0.358491	0.48997644	1	1932	tags=26%, list=9%, signal=29%
BIOCARTA_IL3_PATHWAY	BIOCARTA_IL3_PATHWAY		15	-0.4081168	-1.1066754	0.327273	0.49167487	1	4961	tags=40%, list=23%, signal=52%
TURASHVILI_BREAST_DUCTAL_CARCINOMA_VS_LOBULAR	TURASHVILI_BREAST_DUCTAL_CARCINOMA_VS_LOBULAR_NORI		71	-0.2862393	-1.1063625	0.295082	0.49185723	1	3561	tags=21%, list=16%, signal=25%
KAYO_CALORIE_RESTRICTION_MUSCLE_DN	KAYO_CALORIE_RESTRICTION_MUSCLE_DN		66	-0.298094	-1.1057432	0.259259	0.49303076	1	3497	tags=18%, list=16%, signal=22%
FONTAINE_THYROID_TUMOR_UNCERTAIN_MALIGNANCY_D	FONTAINE_THYROID_TUMOR_UNCERTAIN_MALIGNANCY_DN		25	-0.3715679	-1.1050938	0.338983	0.49396682	1	5401	tags=48%, list=25%, signal=64%
MUELLER_PLURINET	MUELLER_PLURINET		282	-0.2409222	-1.1043065	0.267606	0.49541566	1	4049	tags=22%, list=18%, signal=26%
RODRIGUES_NTN1_AND_DCC_TARGETS	RODRIGUES_NTN1_AND_DCC_TARGETS		32	-0.3631116	-1.1037942	0.333333	0.4960753	1	3652	tags=25%, list=17%, signal=30%
BOYLAN_MULTIPLE_MYELOMA_C_CLUSTER_DN	BOYLAN_MULTIPLE_MYELOMA_C_CLUSTER_DN		29	-0.3498643	-1.1031365	0.303571	0.49714157	1	617	tags=10%, list=3%, signal=11%
SCHLOSSER_SERUM_RESPONSE_UP	SCHLOSSER_SERUM_RESPONSE_UP		122	-0.2603749	-1.1026626	0.333333	0.49773887	1	4941	tags=28%, list=23%, signal=36%
ZUCCHI_METASTASIS_DN	ZUCCHI_METASTASIS_DN		24	-0.3655298	-1.1026466	0.346154	0.49734876	1	4463	tags=29%, list=20%, signal=37%
ELVIDGE_HYPOXIA_DN	ELVIDGE_HYPOXIA_DN		140	-0.267049	-1.1025581	0.241379	0.49710375	1	6877	tags=39%, list=31%, signal=57%
HAN_SATB1_TARGETS_DN	HAN_SATB1_TARGETS_DN		309	-0.2440319	-1.1021408	0.180556	0.49768662	1	5504	tags=28%, list=25%, signal=37%
SASSON_RESPONSE_TO_GONADOTROPHINS_UP	SASSON_RESPONSE_TO_GONADOTROPHINS_UP		69	-0.2796336	-1.101626	0.313726	0.49848524	1	4327	tags=25%, list=20%, signal=31%
SCHLOSSER_MYC_TARGETS_AND_SERUM_RESPONSE_UP	SCHLOSSER_MYC_TARGETS_AND_SERUM_RESPONSE_UP		47	-0.3114125	-1.1008931	0.377359	0.49986956	1	3434	tags=19%, list=16%, signal=23%
REACTOME_DARPP32_EVENTS	REACTOME_DARPP32_EVENTS		25	-0.3382751	-1.1007594	0.282609	0.4997307	1	1504	tags=16%, list=7%, signal=17%
CHIANG_LIVER_CANCER_SUBCLASS_INTERFERON_UP	CHIANG_LIVER_CANCER_SUBCLASS_INTERFERON_UP		24	-0.3660907	-1.1002539	0.275862	0.50059706	1	3832	tags=33%, list=17%, signal=40%
ODONNELL_METASTASIS_DN	ODONNELL_METASTASIS_DN		23	-0.3620437	-1.0999922	0.333333	0.5008525	1	3844	tags=30%, list=18%, signal=37%
KEGG_GNRH_SIGNALING_PATHWAY	KEGG_GNRH_SIGNALING_PATHWAY		97	-0.2696066	-1.0996059	0.278689	0.5012136	1	5837	tags=33%, list=27%, signal=45%
LIANG_HEMATOPOIESIS_STEM_CELL_NUMBER_SMALL_VS	LIANG_HEMATOPOIESIS_STEM_CELL_NUMBER_SMALL_VS_HUGE		33	-0.3491724	-1.0994947	0.305085	0.50102663	1	1939	tags=15%, list=9%, signal=17%
KYNG_DNA_DAMAGE_BY_UV	KYNG_DNA_DAMAGE_BY_UV		25	-0.364273	-1.0989856	0.351852	0.5017478	1	7002	tags=44%, list=32%, signal=65%
KEGG_HOMOLOGOUS_RECOMBINATION	KEGG_HOMOLOGOUS_RECOMBINATION		27	-0.3571492	-1.098203	0.285714	0.5033123	1	2042	tags=19%, list=9%, signal=20%
BIOCARTA_TFF_PATHWAY	BIOCARTA_TFF_PATHWAY		20	-0.3917756	-1.0980775	0.333333	0.50323063	1	5218	tags=45%, list=24%, signal=59%
LUND_SILENCED_BY_METHYLATION	LUND_SILENCED_BY_METHYLATION		15	-0.434017	-1.0980589	0.314815	0.502828	1	4771	tags=33%, list=22%, signal=43%
WANG_BARRETTS_ESOPHAGUS_AND_ESOPHAGUS_CANCE	WANG_BARRETTS_ESOPHAGUS_AND_ESOPHAGUS_CANCER_UP		23	-0.3729123	-1.0980394	0.339286	0.5024256	1	4328	tags=43%, list=20%, signal=54%
BIOCARTA_CSK_PATHWAY	BIOCARTA_CSK_PATHWAY		21	-0.3706436	-1.0953887	0.333333	0.507958	1	3182	tags=29%, list=15%, signal=33%
SHAFFER_IRF4_TARGETS_IN_MYELOMA_VS_MATURE_B_LY	SHAFFER_IRF4_TARGETS_IN_MYELOMA_VS_MATURE_B_LYMPHO		95	-0.2829432	-1.0949651	0.206349	0.50865704	1	5004	tags=23%, list=23%, signal=30%
HAHTOLA_MYCOSIS_FUNGOIDES_CD4_UP	HAHTOLA_MYCOSIS_FUNGOIDES_CD4_UP		57	-0.2986428	-1.0948461	0.333333	0.5085006	1	5364	tags=37%, list=24%, signal=49%
KAUFFMANN_DNA_REPLICATION_GENES	KAUFFMANN_DNA_REPLICATION_GENES		124	-0.2723163	-1.0947305	0.268657	0.50836754	1	6813	tags=37%, list=31%, signal=54%
REACTOME_TRAF6_MEDIATED_INDUCTION_OF_THE_ANTIV	REACTOME_TRAF6_MEDIATED_INDUCTION_OF_THE_ANTIVIRAL		50	-0.3175628	-1.0939162	0.350877	0.5098487	1	4171	tags=28%, list=19%, signal=34%
REACTOME_GLUCAGON_SIGNALING_IN_METABOLIC_REGU	REACTOME_GLUCAGON_SIGNALING_IN_METABOLIC_REGULATIO		33	-0.3530351	-1.0936726	0.310345	0.50998694	1	5767	tags=42%, list=26%, signal=57%
HONMA_DOCETAXEL_RESISTANCE	HONMA_DOCETAXEL_RESISTANCE		30	-0.3322502	-1.0927941	0.303571	0.51169634	1	6747	tags=33%, list=31%, signal=48%
XU_HGF_SIGNALING_NOT_VIA_AKT1_48HR_UP	XU_HGF_SIGNALING_NOT_VIA_AKT1_48HR_UP		33	-0.3408117	-1.0918071	0.392857	0.5138176	1	1292	tags=15%, list=6%, signal=16%
TURASHVILI_BREAST_LOBULAR_CARCINOMA_VS_DUCTAL	TURASHVILI_BREAST_LOBULAR_CARCINOMA_VS_DUCTAL_NORM		61	-0.2786491	-1.0903804	0.277778	0.5165838	1	3561	tags=23%, list=16%, signal=27%
CONCANNON_APOPTOSIS_BY_EPOXOMICIN_UP	CONCANNON_APOPTOSIS_BY_EPOXOMICIN_UP		224	-0.2472235	-1.0903406	0.219178	0.51624477	1	4938	tags=25%, list=23%, signal=31%
KEGG_CHEMOKINE_SIGNALING_PATHWAY	KEGG_CHEMOKINE_SIGNALING_PATHWAY		170	-0.2528221	-1.0901452	0.285714	0.5162118	1	5837	tags=31%, list=27%, signal=41%
JAEGER_METASTASIS_UP	JAEGER_METASTASIS_UP		41	-0.3053177	-1.090007	0.365079	0.5162011	1	3463	tags=24%, list=16%, signal=29%
KEGG_NON_SMALL_CELL_LUNG_CANCER	KEGG_NON_SMALL_CELL_LUNG_CANCER		53	-0.2897608	-1.0896034	0.298246	0.51686233	1	5162	tags=36%, list=24%, signal=47%
PELLICCIOTTA_HDAC_IN_ANTIGEN_PRESENTATION_DN	PELLICCIOTTA_HDAC_IN_ANTIGEN_PRESENTATION_DN		48	-0.3058095	-1.0895723	0.358491	0.51652366	1	6834	tags=42%, list=31%, signal=60%
MCMURRAY_TP53_HRAS_COOPERATION_RESPONSE_DN	MCMURRAY_TP53_HRAS_COOPERATION_RESPONSE_DN		65	-0.2949371	-1.0890884	0.346154	0.51715887	1	3865	tags=25%, list=18%, signal=30%
HOEGERKORP_CD44_TARGETS_TEMPORAL_DN	HOEGERKORP_CD44_TARGETS_TEMPORAL_DN		21	-0.3895392	-1.0887053	0.360656	0.517723	1	3031	tags=33%, list=14%, signal=39%
TRAYNOR_RETT_SYNDROM_UP	TRAYNOR_RETT_SYNDROM_UP		22	-0.3424081	-1.0883728	0.295082	0.5180974	1	3502	tags=32%, list=16%, signal=38%
ROVERSI_GLIOMA_COPY_NUMBER_UP	ROVERSI_GLIOMA_COPY_NUMBER_UP		77	-0.2815209	-1.0881965	0.275862	0.5180883	1	6123	tags=39%, list=28%, signal=54%
LINDGREN_BLADDER_CANCER_CLUSTER_3_DN	LINDGREN_BLADDER_CANCER_CLUSTER_3_DN		213	-0.239219	-1.0880091	0.21875	0.51811355	1	6526	tags=32%, list=30%, signal=45%
SHEN_SMARCA2_TARGETS_DN	SHEN_SMARCA2_TARGETS_DN		295	-0.2356899	-1.0872153	0.237288	0.51950276	1	3111	tags=19%, list=14%, signal=22%
DACOSTA_UV_RESPONSE_VIA_ERCC3_XPCS_UP	DACOSTA_UV_RESPONSE_VIA_ERCC3_XPCS_UP		15	-0.4212952	-1.0862067	0.346154	0.52153695	1	6928	tags=33%, list=32%, signal=49%
MAHADEVAN_RESPONSE_TO_MP470_DN	MAHADEVAN_RESPONSE_TO_MP470_DN		15	-0.4169874	-1.0836647	0.4	0.5273134	1	2188	tags=27%, list=10%, signal=30%
KEGG_OXIDATIVE_PHOSPHORYLATION	KEGG_OXIDATIVE_PHOSPHORYLATION		120	-0.2737265	-1.0834842	0.339286	0.52731645	1	5204	tags=28%, list=24%, signal=36%
KYNG_DNA_DAMAGE_BY_4NQO_OR_UV	KYNG_DNA_DAMAGE_BY_4NQO_OR_UV		32	-0.3174984	-1.0828153	0.392857	0.5286943	1	825	tags=13%, list=4%, signal=13%
SAGIV_CD24_TARGETS_DN	SAGIV_CD24_TARGETS_DN		43	-0.3172804	-1.0824653	0.345455	0.5290943	1	4255	tags=30%, list=19%, signal=37%
NAKAJIMA_EOSINOPHIL	NAKAJIMA_EOSINOPHIL		20	-0.3807745	-1.0824103	0.357143	0.5288287	1	3090	tags=30%, list=14%, signal=35%
LEE_LIVER_CANCER_MYC_DN	LEE_LIVER_CANCER_MYC_DN		57	-0.3014134	-1.0822563	0.322034	0.5287396	1	4385	tags=26%, list=20%, signal=33%
AMIT_EGF_RESPONSE_240_HELA	AMIT_EGF_RESPONSE_240_HELA		60	-0.2812656	-1.0821689	0.3125	0.52851206	1	4227	tags=28%, list=19%, signal=35%
ZHANG_RESPONSE_TO_IKK_INHIBITOR_AND_TNF_DN	ZHANG_RESPONSE_TO_IKK_INHIBITOR_AND_TNF_DN		95	-0.2741821	-1.0814052	0.269841	0.52995855	1	2819	tags=18%, list=13%, signal=20%
GARGALOVIC_RESPONSE_TO_OXIDIZED_PHOSPHOLIPIDS	GARGALOVIC_RESPONSE_TO_OXIDIZED_PHOSPHOLIPIDS_RED_		17	-0.3914446	-1.0809883	0.327273	0.53049475	1	4578	tags=29%, list=21%, signal=37%
REACTOME_NUCLEAR_EVENTS_KINASE_AND_TRANSCRIPT	REACTOME_NUCLEAR_EVENTS_KINASE_AND_TRANSCRIPTION_F		24	-0.3699575	-1.0800959	0.37037	0.5321167	1	925	tags=17%, list=4%, signal=17%
VERNELL_RETINOBLASTOMA_PATHWAY_UP	VERNELL_RETINOBLASTOMA_PATHWAY_UP		39	-0.3231471	-1.0799053	0.310345	0.53222173	1	6002	tags=36%, list=27%, signal=49%

REACTOME_PHASE_II_CONJUGATION	REACTOME_PHASE_II_CONJUGATION		43	-0.3022342	-1.0797222	0.25	0.53219825	1	5489	tags=37%, list=25%, signal=50%
GEORGES_CELL_CYCLE_MIR192_TARGETS	GEORGES_CELL_CYCLE_MIR192_TARGETS		58	-0.3110187	-1.0793653	0.320755	0.53253645	1	6191	tags=38%, list=28%, signal=53%
ZHAN_LATE_DIFFERENTIATION_GENES_UP	ZHAN_LATE_DIFFERENTIATION_GENES_UP		23	-0.3691304	-1.0790707	0.259259	0.53280175	1	825	tags=9%, list=4%, signal=9%
REACTOME_ERK_MAPK_TARGETS	REACTOME_ERK_MAPK_TARGETS		21	-0.3757743	-1.0788661	0.333333	0.53294003	1	4961	tags=33%, list=23%, signal=43%
KEGG_ENDOCYTOSIS	KEGG_ENDOCYTOSIS		173	-0.2385844	-1.0788248	0.234375	0.5325947	1	6617	tags=36%, list=30%, signal=52%
KEGG_ALDOSTERONE_REGULATED_SODIUM_REABSORPTIO	KEGG_ALDOSTERONE_REGULATED_SODIUM_REABSORPTION		41	-0.3098832	-1.0777359	0.339623	0.5343183	1	1673	tags=15%, list=8%, signal=16%
WAMUNYOKOLI_OVARIAN_CANCER_GRADES_1_2_DN	WAMUNYOKOLI_OVARIAN_CANCER_GRADES_1_2_DN		60	-0.2926229	-1.0756298	0.290909	0.5393393	1	3272	tags=23%, list=15%, signal=27%
REACTOME_INACTIVATION_OF_APC_VIA_DIRECT_INHIBITI	REACTOME_INACTIVATION_OF_APC_VIA_DIRECT_INHIBITION_C		18	-0.3808409	-1.0752996	0.377049	0.5396348	1	3503	tags=22%, list=16%, signal=26%
RUGO_STRESS_RESPONSE_SUBSET_G	RUGO_STRESS_RESPONSE_SUBSET_G		32	-0.3174984	-1.0748612	0.333333	0.5400309	1	825	tags=13%, list=4%, signal=13%
HOUSTIS_ROS	HOUSTIS_ROS		30	-0.3319438	-1.0747206	0.297872	0.53993434	1	5892	tags=30%, list=27%, signal=41%
BIOCARTA_CASPASE_PATHWAY	BIOCARTA_CASPASE_PATHWAY		22	-0.3761261	-1.0738752	0.37037	0.54189277	1	5426	tags=41%, list=25%, signal=54%
SMITH_TERT_TARGETS_UP	SMITH_TERT_TARGETS_UP		126	-0.2616014	-1.0737163	0.344262	0.5417926	1	6136	tags=35%, list=28%, signal=48%
REACTOME_HOMOLOGOUS_RECOMBINATION_REPAIR	REACTOME_HOMOLOGOUS_RECOMBINATION_REPAIR		15	-0.4106888	-1.0727156	0.327273	0.5437013	1	1799	tags=20%, list=8%, signal=22%
CHARAFE_BREAST_CANCER_LUMINAL_VS_BASAL_UP	CHARAFE_BREAST_CANCER_LUMINAL_VS_BASAL_UP		350	-0.2279195	-1.0725185	0.344262	0.5437717	1	3677	tags=19%, list=17%, signal=23%
RIZKI_TUMOR_INVASIVENESS_2D_UP	RIZKI_TUMOR_INVASIVENESS_2D_UP		59	-0.2867225	-1.0722588	0.306122	0.54391307	1	5697	tags=32%, list=26%, signal=43%
VANTVEER_BREAST_CANCER_BRCA1_DN	VANTVEER_BREAST_CANCER_BRCA1_DN		36	-0.315985	-1.0719745	0.311475	0.5440303	1	4473	tags=31%, list=20%, signal=38%
RICKMAN_TUMOR_DIFFERENTIATED_MODERATELY_VS_PO	RICKMAN_TUMOR_DIFFERENTIATED_MODERATELY_VS_POORLY_		41	-0.3018262	-1.0719682	0.357143	0.5436334	1	7487	tags=49%, list=34%, signal=74%
GALE_APL_WITH_FLT3_MUTATED_DN	GALE_APL_WITH_FLT3_MUTATED_DN		16	-0.4048528	-1.0706735	0.318182	0.5464737	1	6018	tags=44%, list=27%, signal=60%
YANG_BREAST_CANCER_ESR1_LASER_UP	YANG_BREAST_CANCER_ESR1_LASER_UP		26	-0.3557883	-1.0704769	0.372549	0.54654425	1	5446	tags=42%, list=25%, signal=56%
HENDRICKS_SMARCA4_TARGETS_DN	HENDRICKS_SMARCA4_TARGETS_DN		39	-0.3224596	-1.069671	0.269231	0.5482511	1	4794	tags=28%, list=22%, signal=36%
REACTOME_TRNA_AMINOACYLATION	REACTOME_TRNA_AMINOACYLATION		40	-0.315167	-1.069363	0.333333	0.54845697	1	5701	tags=35%, list=26%, signal=47%
SCHUETZ_BREAST_CANCER_DUCTAL_INVASIVE_DN	SCHUETZ_BREAST_CANCER_DUCTAL_INVASIVE_DN		80	-0.2782233	-1.0685438	0.35	0.5500268	1	1705	tags=13%, list=8%, signal=14%
WANG_RECURRENT_LIVER_CANCER_DN	WANG_RECURRENT_LIVER_CANCER_DN		15	-0.4171384	-1.0669559	0.446429	0.5534184	1	6416	tags=53%, list=29%, signal=75%
LU_TUMOR_VASCULATURE_UP	LU_TUMOR_VASCULATURE_UP		26	-0.347516	-1.0668983	0.416667	0.55307436	1	5465	tags=38%, list=25%, signal=51%
MORI_MATURE_B_LYMPHOCYTE_UP	MORI_MATURE_B_LYMPHOCYTE_UP		72	-0.2738069	-1.0666625	0.318182	0.5531039	1	7194	tags=39%, list=33%, signal=58%
MONNIER_POSTRADIATION_TUMOR_ESCAPE_DN	MONNIER_POSTRADIATION_TUMOR_ESCAPE_DN		336	-0.2265863	-1.0665424	0.287879	0.55293834	1	5080	tags=24%, list=23%, signal=31%
KEGG_DRUG_METABOLISM_CYTOCHROME_P450	KEGG_DRUG_METABOLISM_CYTOCHROME_P450		51	-0.3072725	-1.0657809	0.272727	0.55428857	1	4787	tags=29%, list=22%, signal=38%
YAO_TEMPORAL_RESPONSE_TO_PROGESTERONE_CLUSTEF	YAO_TEMPORAL_RESPONSE_TO_PROGESTERONE_CLUSTER_4		16	-0.3744061	-1.0655402	0.44	0.5545076	1	1448	tags=19%, list=7%, signal=20%
REACTOME_AUTODEGRADATION_OF_CDH1_BY_CDH1_APC	REACTOME_AUTODEGRADATION_OF_CDH1_BY_CDH1_APC		57	-0.2817534	-1.0650767	0.338983	0.55508935	1	7004	tags=35%, list=32%, signal=51%
STEIN_ESRRA_TARGETS_RESPONSIVE_TO_ESTROGEN_UP	STEIN_ESRRA_TARGETS_RESPONSIVE_TO_ESTROGEN_UP		27	-0.3441127	-1.064813	0.387755	0.5553881	1	6751	tags=37%, list=31%, signal=53%
JISON_SICKLE_CELL_DISEASE_UP	JISON_SICKLE_CELL_DISEASE_UP		172	-0.2497617	-1.0646925	0.322034	0.5552239	1	4740	tags=26%, list=22%, signal=32%
CREIGHTON_ENDOCRINE_THERAPY_RESISTANCE_5	CREIGHTON_ENDOCRINE_THERAPY_RESISTANCE_5		441	-0.2222849	-1.0638629	0.275362	0.55662847	1	6136	tags=32%, list=28%, signal=44%
KEGG_RENIN_ANGIOTENSIN_SYSTEM	KEGG_RENIN_ANGIOTENSIN_SYSTEM		16	-0.3727856	-1.0618981	0.413043	0.5614776	1	3463	tags=31%, list=16%, signal=37%
LIU_SOX4_TARGETS_DN	LIU_SOX4_TARGETS_DN		296	-0.2290354	-1.0617957	0.285714	0.5612184	1	5177	tags=24%, list=24%, signal=31%
SU_TESTIS	SU_TESTIS		70	-0.2898315	-1.061683	0.326923	0.5610285	1	2920	tags=21%, list=13%, signal=25%
KEGG_HEDGEHOG_SIGNALING_PATHWAY	KEGG_HEDGEHOG_SIGNALING_PATHWAY		54	-0.2937443	-1.061192	0.327586	0.5618829	1	5356	tags=37%, list=24%, signal=49%
BIOCARTA_MAL_PATHWAY	BIOCARTA_MAL_PATHWAY		19	-0.3669736	-1.0611302	0.339286	0.5616685	1	2940	tags=21%, list=13%, signal=24%
WELCSH_BRCA1_TARGETS_1_UP	WELCSH_BRCA1_TARGETS_1_UP		164	-0.257855	-1.060909	0.315789	0.56190157	1	5194	tags=25%, list=24%, signal=33%
SHAFFER_IRF4_TARGETS_IN_ACTIVATED_DENDRITIC_CEL	SHAFFER_IRF4_TARGETS_IN_ACTIVATED_DENDRITIC_CELL		64	-0.2892998	-1.060662	0.380952	0.56205636	1	3622	tags=17%, list=17%, signal=21%
BIOCARTA_RELA_PATHWAY	BIOCARTA_RELA_PATHWAY		16	-0.3540251	-1.0605363	0.358491	0.5619327	1	131	tags=6%, list=1%, signal=6%
TAVOR_CEBPA_TARGETS_UP	TAVOR_CEBPA_TARGETS_UP		42	-0.3040169	-1.0605032	0.315789	0.5615517	1	4037	tags=24%, list=18%, signal=29%
LEE_LIVER_CANCER_MYC_UP	LEE_LIVER_CANCER_MYC_UP		50	-0.3018802	-1.0595657	0.360656	0.56355834	1	3407	tags=22%, list=16%, signal=26%
YOSHIOKA_LIVER_CANCER_EARLY_RECURRENCE_DN	YOSHIOKA_LIVER_CANCER_EARLY_RECURRENCE_DN		52	-0.3011907	-1.0594326	0.375	0.5634568	1	3483	tags=21%, list=16%, signal=25%
NIKOLSKY_BREAST_CANCER_17Q21_Q25_AMPLICON	NIKOLSKY_BREAST_CANCER_17Q21_Q25_AMPLICON		309	-0.2250151	-1.059324	0.230769	0.5632767	1	4822	tags=22%, list=22%, signal=28%
PRAMOONJAGO_SOX4_TARGETS_UP	PRAMOONJAGO_SOX4_TARGETS_UP		49	-0.3097427	-1.0592645	0.346154	0.5630526	1	6659	tags=43%, list=30%, signal=61%
HOSHIDA_LIVER_CANCER_SUBCLASS_S3	HOSHIDA_LIVER_CANCER_SUBCLASS_S3		252	-0.2375292	-1.0588087	0.344262	0.56375504	1	4391	tags=23%, list=20%, signal=29%
BIOCARTA_INSULIN_PATHWAY	BIOCARTA_INSULIN_PATHWAY		22	-0.3428324	-1.0585576	0.40678	0.56402105	1	2947	tags=23%, list=13%, signal=26%
RUIZ_TNC_TARGETS_UP	RUIZ_TNC_TARGETS_UP		147	-0.2444842	-1.0575554	0.290909	0.5658681	1	4331	tags=24%, list=20%, signal=29%
BIOCARTA_IGF1R_PATHWAY	BIOCARTA_IGF1R_PATHWAY		22	-0.3639564	-1.0563322	0.297872	0.56866693	1	5608	tags=45%, list=26%, signal=61%
LANDIS_ERBB2_BREAST_TUMORS_65_UP	LANDIS_ERBB2_BREAST_TUMORS_65_UP		22	-0.3668982	-1.0562702	0.375	0.5683725	1	6023	tags=36%, list=27%, signal=50%
REACTOME_CDC20_PHOSPHO_APC_MEDIATED_DEGRADAT	REACTOME_CDC20_PHOSPHO_APC_MEDIATED_DEGRADATION_C		62	-0.301687	-1.0562654	0.298246	0.5679453	1	3678	tags=21%, list=17%, signal=25%
REACTOME_G1_PHASE	REACTOME_G1_PHASE		16	-0.3847408	-1.0549372	0.411765	0.57082707	1	5162	tags=38%, list=24%, signal=49%
MITSIADES_RESPONSE_TO_APLIDIN_UP	MITSIADES_RESPONSE_TO_APLIDIN_UP		406	-0.2292341	-1.0548619	0.273973	0.5704869	1	4477	tags=20%, list=20%, signal=25%
NAKAMURA_METASTASIS_MODEL_DN	NAKAMURA_METASTASIS_MODEL_DN		42	-0.2896926	-1.0543274	0.408163	0.5714435	1	4150	tags=29%, list=19%, signal=35%
THEILGAARD_NEUTROPHIL_AT_SKIN_WOUND_UP	THEILGAARD_NEUTROPHIL_AT_SKIN_WOUND_UP		72	-0.2701542	-1.0537833	0.369231	0.5722668	1	4177	tags=21%, list=19%, signal=26%
REACTOME_ZINC_TRANSPORTATION	REACTOME_ZINC_TRANSPORTATION		17	-0.3880733	-1.053144	0.346939	0.57362735	1	6187	tags=35%, list=28%, signal=49%
JEON_SMAD6_TARGETS_UP	JEON_SMAD6_TARGETS_UP		22	-0.3489433	-1.0526845	0.410714	0.57439005	1	4838	tags=45%, list=22%, signal=58%
NIKOLSKY_BREAST_CANCER_20Q12_Q13_AMPLICON	NIKOLSKY_BREAST_CANCER_20Q12_Q13_AMPLICON		135	-0.2485177	-1.0526596	0.391304	0.57401484	1	5633	tags=27%, list=26%, signal=37%
WEIGEL_OXIDATIVE_STRESS_BY_TBH_AND_H2O2	WEIGEL_OXIDATIVE_STRESS_BY_TBH_AND_H2O2		35	-0.318824	-1.052163	0.344262	0.57498354	1	4864	tags=34%, list=22%, signal=44%
LUI_THYROID_CANCER_CLUSTER_3	LUI_THYROID_CANCER_CLUSTER_3		27	-0.3074956	-1.0517793	0.339286	0.5754587	1	7525	tags=44%, list=34%, signal=68%
KEGG_FATTY_ACID_METABOLISM	KEGG_FATTY_ACID_METABOLISM		39	-0.3121303	-1.0514485	0.409836	0.5758025	1	7010	tags=49%, list=32%, signal=71%
BERTUCCI_MEDULLARY_VS_DUCTAL_BREAST_CANCER_UP	BERTUCCI_MEDULLARY_VS_DUCTAL_BREAST_CANCER_UP		187	-0.240386	-1.0511683	0.377049	0.5761233	1	6728	tags=39%, list=31%, signal=55%
HOSHIDA_LIVER_CANCER_SURVIVAL_DN	HOSHIDA_LIVER_CANCER_SURVIVAL_DN		109	-0.2610702	-1.0506757	0.391304	0.57698953	1	4787	tags=29%, list=22%, signal=37%
ELVIDGE_HIF1A_AND_HIF2A_TARGETS_DN	ELVIDGE_HIF1A_AND_HIF2A_TARGETS_DN		100	-0.2631688	-1.0500764	0.383333	0.57798696	1	4941	tags=25%, list=23%, signal=32%

BIOCARTA_PYK2_PATHWAY	BIOCARTA_PYK2_PATHWAY		28	-0.3361805	-1.0497637	0.296296	0.578219	1	5725	tags=43%, list=26%, signal=58%
KIM_WT1_TARGETS_DN	KIM_WT1_TARGETS_DN		442	-0.2206325	-1.048572	0.347826	0.5807395	1	4334	tags=21%, list=20%, signal=26%
KOBAYASHI_RESPONSE_TO_ROMIDEPSIN	KOBAYASHI_RESPONSE_TO_ROMIDEPSIN		17	-0.3810666	-1.0476656	0.375	0.5826969	1	2530	tags=24%, list=12%, signal=27%
CHIARETTI_T_ALL_REFRACTORY_TO_THERAPY	CHIARETTI_T_ALL_REFRACTORY_TO_THERAPY		25	-0.3257793	-1.0476111	0.354167	0.5823719	1	4283	tags=32%, list=20%, signal=40%
LEE_LIVER_CANCER_TOP50	LEE_LIVER_CANCER_TOP50		39	-0.3140067	-1.0472484	0.358491	0.58273727	1	5250	tags=33%, list=24%, signal=44%
DUNNE_TARGETS_OF_AML1_MTG8_FUSION_UP	DUNNE_TARGETS_OF_AML1_MTG8_FUSION_UP		39	-0.3256688	-1.0465342	0.375	0.58428913	1	3603	tags=18%, list=16%, signal=21%
REACTOME_RHO_GTPASE_CYCLE	REACTOME_RHO_GTPASE_CYCLE		118	-0.2534037	-1.0463696	0.322034	0.58418345	1	4191	tags=25%, list=19%, signal=31%
GENTILE_UV_HIGH_DOSE_DN	GENTILE_UV_HIGH_DOSE_DN		242	-0.2293184	-1.0462714	0.387097	0.5839579	1	5871	tags=29%, list=27%, signal=39%
REACTOME_P53_INDEPENDENT_DNA_DAMAGE_RESPONSE	REACTOME_P53_INDEPENDENT_DNA_DAMAGE_RESPONSE		43	-0.2976988	-1.0456374	0.40625	0.5849875	1	6834	tags=37%, list=31%, signal=54%
RORIE_TARGETS_OF_EWSR1_FLI1_FUSION_UP	RORIE_TARGETS_OF_EWSR1_FLI1_FUSION_UP		24	-0.3591415	-1.0455606	0.44898	0.5847295	1	5079	tags=42%, list=23%, signal=54%
IWANAGA_CARCINOGENESIS_BY_KRAS_PTEN_UP	IWANAGA_CARCINOGENESIS_BY_KRAS_PTEN_UP		172	-0.2375302	-1.045326	0.359375	0.5849735	1	2172	tags=11%, list=10%, signal=12%
KEGG_MTOR_SIGNALING_PATHWAY	KEGG_MTOR_SIGNALING_PATHWAY		52	-0.2975173	-1.0450553	0.433962	0.5855103	1	2652	tags=17%, list=12%, signal=20%
CAIRO_LIVER_DEVELOPMENT_UP	CAIRO_LIVER_DEVELOPMENT_UP		158	-0.2355924	-1.0445459	0.360656	0.58656806	1	4518	tags=22%, list=21%, signal=27%
BIOCARTA_MITOCHONDRIA_PATHWAY	BIOCARTA_MITOCHONDRIA_PATHWAY		21	-0.3569687	-1.0441191	0.387755	0.58723146	1	7118	tags=48%, list=32%, signal=70%
KEGG_ARRHYTHMOGENIC_RIGHT_VENTRICULAR_CARDIOM	KEGG_ARRHYTHMOGENIC_RIGHT_VENTRICULAR_CARDIOMYOPA		73	-0.2838691	-1.0440706	0.344262	0.5869403	1	3732	tags=26%, list=17%, signal=31%
CORRE_MULTIPLE_MYELOMA_UP	CORRE_MULTIPLE_MYELOMA_UP		65	-0.2825615	-1.0440614	0.358491	0.5864863	1	3041	tags=23%, list=14%, signal=27%
KEGG_THYROID_CANCER	KEGG_THYROID_CANCER		29	-0.3231601	-1.0439954	0.32	0.58628005	1	5218	tags=38%, list=24%, signal=50%
REACTOME_PEPTIDE_LIGAND_BINDING_RECEPTORS	REACTOME_PEPTIDE_LIGAND_BINDING_RECEPTORS		156	-0.2474428	-1.0432777	0.349206	0.58726007	1	4097	tags=22%, list=19%, signal=27%
SMID_BREAST_CANCER_RELAPSE_IN_LUNG_UP	SMID_BREAST_CANCER_RELAPSE_IN_LUNG_UP		21	-0.3690715	-1.0432166	0.392857	0.58692694	1	236	tags=10%, list=1%, signal=10%
XU_GH1_AUTOCRINE_TARGETS_DN	XU_GH1_AUTOCRINE_TARGETS_DN		117	-0.2519194	-1.0431409	0.393443	0.5866918	1	3793	tags=22%, list=17%, signal=27%
KEGG_N_GLYCAN_BIOSYNTHESIS	KEGG_N_GLYCAN_BIOSYNTHESIS		45	-0.303705	-1.0424116	0.357143	0.58819515	1	6208	tags=44%, list=28%, signal=62%
HASLINGER_B_CLL_WITH_CHROMOSOME_12_TRISOMY	HASLINGER_B_CLL_WITH_CHROMOSOME_12_TRISOMY		16	-0.3915348	-1.042082	0.358491	0.5885075	1	7725	tags=63%, list=35%, signal=96%
ST_GA13_PATHWAY	ST_GA13_PATHWAY		32	-0.3282075	-1.0396774	0.5	0.5943594	1	4665	tags=31%, list=21%, signal=40%
CERVERA_SDHB_TARGETS_2	CERVERA_SDHB_TARGETS_2		105	-0.26127	-1.0395907	0.373134	0.5942158	1	3150	tags=22%, list=14%, signal=25%
NELSON_RESPONSE_TO_ANDROGEN_UP	NELSON_RESPONSE_TO_ANDROGEN_UP		79	-0.2703997	-1.0393414	0.448276	0.5944076	1	4118	tags=25%, list=19%, signal=31%
MORI_PLASMA_CELL_DN	MORI_PLASMA_CELL_DN		21	-0.3430447	-1.0391399	0.381818	0.5945348	1	7260	tags=57%, list=33%, signal=85%
GAUSSMANN_MLL_AF4_FUSION_TARGETS_E_DN	GAUSSMANN_MLL_AF4_FUSION_TARGETS_E_DN		19	-0.3512779	-1.0389184	0.352941	0.5947682	1	3615	tags=26%, list=16%, signal=31%
BERENJENO_ROCK_SIGNALING_NOT_VIA_RHOA_DN	BERENJENO_ROCK_SIGNALING_NOT_VIA_RHOA_DN		39	-0.3107958	-1.0389167	0.421053	0.59433377	1	4269	tags=26%, list=19%, signal=32%
KEGG_TYPE_I_DIABETES_MELLITUS	KEGG_TYPE_I_DIABETES_MELLITUS		35	-0.2985797	-1.0387176	0.340426	0.5944269	1	3103	tags=23%, list=14%, signal=27%
VANHARANTA_UTERINE_FIBROID_DN	VANHARANTA_UTERINE_FIBROID_DN		61	-0.2799613	-1.0383725	0.425532	0.5949308	1	5426	tags=33%, list=25%, signal=43%
CHIARETTI_ACUTE_LYMPHOBLASTIC_LEUKEMIA_ZAP70	CHIARETTI_ACUTE_LYMPHOBLASTIC_LEUKEMIA_ZAP70		63	-0.2954963	-1.0371002	0.42	0.59753925	1	5350	tags=27%, list=24%, signal=36%
DOANE_BREAST_CANCER_ESR1_UP	DOANE_BREAST_CANCER_ESR1_UP		103	-0.2604502	-1.0368283	0.403226	0.5977767	1	2238	tags=13%, list=10%, signal=14%
KEGG_DRUG_METABOLISM_OTHER_ENZYMES	KEGG_DRUG_METABOLISM_OTHER_ENZYMES		36	-0.3084143	-1.0368067	0.415094	0.59735245	1	5200	tags=33%, list=24%, signal=44%
REACTOME_AMINE_COMPOUND_SLC_TRANSPORTERS	REACTOME_AMINE_COMPOUND_SLC_TRANSPORTERS		29	-0.3232639	-1.0367891	0.425532	0.5969291	1	3402	tags=21%, list=16%, signal=24%
ROYLANCE_BREAST_CANCER_16Q_COPY_NUMBER_DN	ROYLANCE_BREAST_CANCER_16Q_COPY_NUMBER_DN		16	-0.3692961	-1.0356289	0.428571	0.5994778	1	1589	tags=25%, list=7%, signal=27%
BIOCARTA_P53HYPOXIA_PATHWAY	BIOCARTA_P53HYPOXIA_PATHWAY		22	-0.3600298	-1.0355366	0.34	0.5992355	1	2243	tags=23%, list=10%, signal=25%
NIKOLSKY_BREAST_CANCER_8Q23_Q24_AMPLICON	NIKOLSKY_BREAST_CANCER_8Q23_Q24_AMPLICON		142	-0.242018	-1.0355155	0.389831	0.5988543	1	6869	tags=42%, list=31%, signal=60%
TOYOTA_TARGETS_OF_MIR34B_AND_MIR34C	TOYOTA_TARGETS_OF_MIR34B_AND_MIR34C		409	-0.2206661	-1.0345267	0.390625	0.60150826	1	3765	tags=18%, list=17%, signal=21%
STREICHER_LSM1_TARGETS_UP	STREICHER_LSM1_TARGETS_UP		40	-0.2869392	-1.0338748	0.388889	0.6027809	1	4671	tags=33%, list=21%, signal=41%
VALK_AML_CLUSTER_9	VALK_AML_CLUSTER_9		34	-0.3031487	-1.0338426	0.369231	0.6023873	1	3252	tags=18%, list=15%, signal=21%
BENPORATH_OCT4_TARGETS	BENPORATH_OCT4_TARGETS		278	-0.2239512	-1.0323274	0.370968	0.6058821	1	4500	tags=24%, list=21%, signal=30%
REACTOME_SEMA4D_INDUCED_CELL_MIGRATION_AND_GI	REACTOME_SEMA4D_INDUCED_CELL_MIGRATION_AND_GROWT		24	-0.3396817	-1.0322218	0.442623	0.6056889	1	5580	tags=33%, list=25%, signal=45%
WEBER_METHYLATED_HCP_IN_FIBROBLAST_DN	WEBER_METHYLATED_HCP_IN_FIBROBLAST_DN		28	-0.3112247	-1.030701	0.421875	0.60904884	1	4008	tags=29%, list=18%, signal=35%
REACTOME_THROMBIN_SIGNALLING_THROUGH_PROTEINA	REACTOME_THROMBIN_SIGNALLING_THROUGH_PROTEINASE_A		27	-0.3449955	-1.0304087	0.446429	0.6092782	1	4066	tags=30%, list=19%, signal=36%
REACTOME_GLOBAL_GENOMIC_NER	REACTOME_GLOBAL_GENOMIC_NER		33	-0.2999915	-1.0303868	0.385965	0.6088701	1	2870	tags=18%, list=13%, signal=21%
KOKKINAKIS_METHIONINE_DEPRIVATION_48HR_DN	KOKKINAKIS_METHIONINE_DEPRIVATION_48HR_DN		60	-0.2799561	-1.0290285	0.370968	0.6119687	1	4283	tags=25%, list=20%, signal=31%
DAUER_STAT3_TARGETS_UP	DAUER_STAT3_TARGETS_UP		37	-0.2982979	-1.028236	0.413043	0.6133819	1	4201	tags=30%, list=19%, signal=37%
CREIGHTON_ENDOCRINE_THERAPY_RESISTANCE_1	CREIGHTON_ENDOCRINE_THERAPY_RESISTANCE_1		484	-0.2191423	-1.0274371	0.369231	0.61504924	1	5682	tags=26%, list=26%, signal=34%
BIOCARTA_NKCELLS_PATHWAY	BIOCARTA_NKCELLS_PATHWAY		19	-0.3701766	-1.0273882	0.409091	0.6147136	1	889	tags=16%, list=4%, signal=16%
BIOCARTA_VIP_PATHWAY	BIOCARTA_VIP_PATHWAY		25	-0.3109951	-1.0270951	0.431373	0.6147801	1	796	tags=8%, list=4%, signal=8%
PUJANA_BREAST_CANCER_LIT_INT_NETWORK	PUJANA_BREAST_CANCER_LIT_INT_NETWORK		99	-0.2635532	-1.0269116	0.47541	0.6148236	1	5591	tags=27%, list=25%, signal=36%
REACTOME_OPIOID_SIGNALLING	REACTOME_OPIOID_SIGNALLING		82	-0.2610719	-1.0267822	0.47541	0.6146898	1	5767	tags=30%, list=26%, signal=41%
KAAB_FAILED_HEART_VENTRICLE_DN	KAAB_FAILED_HEART_VENTRICLE_DN		38	-0.3289141	-1.0267524	0.3125	0.61430126	1	3595	tags=24%, list=16%, signal=28%
RASHI_RESPONSE_TO_IONIZING_RADIATION_5	RASHI_RESPONSE_TO_IONIZING_RADIATION_5		136	-0.2431925	-1.0261728	0.4	0.6152933	1	5273	tags=27%, list=24%, signal=36%
KAPOSI_LIVER_CANCER_POOR_SURVIVAL_UP	KAPOSI_LIVER_CANCER_POOR_SURVIVAL_UP		17	-0.3678994	-1.0246342	0.395349	0.61862934	1	6145	tags=35%, list=28%, signal=49%
HOLLMAN_APOPTOSIS_VIA_CD40_DN	HOLLMAN_APOPTOSIS_VIA_CD40_DN		240	-0.2279533	-1.0231594	0.439394	0.6217527	1	3349	tags=17%, list=15%, signal=19%
REACTOME_APCDC20_MEDIATED_DEGRADATION_OF_CYCL	REACTOME_APCDC20_MEDIATED_DEGRADATION_OF_CYCLIN_B		17	-0.3697936	-1.0228136	0.409836	0.622307	1	1116	tags=12%, list=5%, signal=12%
BIOCARTA_SHH_PATHWAY	BIOCARTA_SHH_PATHWAY		15	-0.3998916	-1.0224198	0.470588	0.62307423	1	5232	tags=53%, list=24%, signal=70%
REACTOME_GPCR_LIGAND_BINDING	REACTOME_GPCR_LIGAND_BINDING		364	-0.2090694	-1.0203078	0.368421	0.6277168	1	4211	tags=22%, list=19%, signal=26%
KEGG_ABC_TRANSPORTERS	KEGG_ABC_TRANSPORTERS		42	-0.3114193	-1.0197972	0.418182	0.6284586	1	4937	tags=33%, list=23%, signal=43%
BEGUM_TARGETS_OF_PAX3_FOXO1_FUSION_UP	BEGUM_TARGETS_OF_PAX3_FOXO1_FUSION_UP		49	-0.2928848	-1.0192343	0.425926	0.62937295	1	2734	tags=18%, list=12%, signal=21%
SA_TRKA_RECEPTOR	SA_TRKA_RECEPTOR		15	-0.3837302	-1.0192256	0.488889	0.6289238	1	4740	tags=47%, list=22%, signal=59%
KEGG_GLYCINE_SERINE_AND_THREONINE_METABOLISM	KEGG_GLYCINE_SERINE_AND_THREONINE_METABOLISM		31	-0.3147501	-1.017448	0.448276	0.6329989	1	5322	tags=26%, list=24%, signal=34%
OUYANG_PROSTATE_CANCER_PROGRESSION_DN	OUYANG_PROSTATE_CANCER_PROGRESSION_DN		19	-0.3503685	-1.0174148	0.491228	0.63258964	1	844	tags=16%, list=4%, signal=16%

BREDEMEYER_RAG_SIGNALING_VIA_ATM_NOT_VIA_NFKB	BREDEMEYER_RAG_SIGNALING_VIA_ATM_NOT_VIA_NFKB_UP		48	-0.2873085	-1.01663	0.377778	0.63404936	1	4521	tags=29%, list=21%, signal=37%
IVANOVA_HEMATOPOIESIS_STEM_CELL_LONG_TERM	IVANOVA_HEMATOPOIESIS_STEM_CELL_LONG_TERM		82	-0.2633832	-1.0151429	0.412698	0.6373125	1	2898	tags=20%, list=13%, signal=22%
BIOCARTA_CDMAC_PATHWAY	BIOCARTA_CDMAC_PATHWAY		16	-0.3729501	-1.0147798	0.42623	0.63772553	1	4961	tags=38%, list=23%, signal=48%
KEGG_CELL_ADHESION_MOLECULES_CAMS	KEGG_CELL_ADHESION_MOLECULES_CAMS		121	-0.2460446	-1.0145766	0.387097	0.63780385	1	3264	tags=21%, list=15%, signal=24%
BROWNE_HCMV_INFECTION_14HR_UP	BROWNE_HCMV_INFECTION_14HR_UP		145	-0.2385156	-1.0143529	0.52459	0.63792485	1	4108	tags=26%, list=19%, signal=32%
REACTOME_SEMA4D_IN_SEMAPHORIN_SIGNALING	REACTOME_SEMA4D_IN_SEMAPHORIN_SIGNALING		29	-0.3342319	-1.0141475	0.418182	0.6380445	1	7813	tags=45%, list=36%, signal=70%
KYNG_DNA_DAMAGE_DN	KYNG_DNA_DAMAGE_DN		98	-0.2454336	-1.0132715	0.438597	0.6395596	1	3070	tags=17%, list=14%, signal=20%
VANTVEER_BREAST_CANCER_ESR1_UP	VANTVEER_BREAST_CANCER_ESR1_UP		139	-0.24042	-1.0126992	0.508475	0.6405214	1	5276	tags=25%, list=24%, signal=33%
BIOCARTA_BAD_PATHWAY	BIOCARTA_BAD_PATHWAY		25	-0.3497379	-1.011982	0.483333	0.6421147	1	5176	tags=32%, list=24%, signal=42%
BIOCARTA_FCER1_PATHWAY	BIOCARTA_FCER1_PATHWAY		38	-0.2968261	-1.0119479	0.423729	0.6417637	1	4980	tags=26%, list=23%, signal=34%
YAO_TEMPORAL_RESPONSE_TO_PROGESTERONE_CLUSTER	YAO_TEMPORAL_RESPONSE_TO_PROGESTERONE_CLUSTER_11		94	-0.2655278	-1.0113833	0.428571	0.64288133	1	8291	tags=48%, list=38%, signal=77%
BIOCARTA_FMLP_PATHWAY	BIOCARTA_FMLP_PATHWAY		35	-0.3108808	-1.0110861	0.42	0.6432464	1	4980	tags=29%, list=23%, signal=37%
REACTOME_SIGNALING_BY_WNT	REACTOME_SIGNALING_BY_WNT		57	-0.2729425	-1.0098543	0.467742	0.6464386	1	6834	tags=35%, list=31%, signal=51%
CASORELLI_ACUTE_PROMYELOCYTIC_LEUKEMIA_UP	CASORELLI_ACUTE_PROMYELOCYTIC_LEUKEMIA_UP		154	-0.2303323	-1.00959	0.5	0.6466013	1	2414	tags=12%, list=11%, signal=14%
REACTOME_BASIGIN_INTERACTIONS	REACTOME_BASIGIN_INTERACTIONS		25	-0.3355561	-1.0089928	0.470588	0.6477298	1	3964	tags=28%, list=18%, signal=34%
BIOCARTA_NO2IL12_PATHWAY	BIOCARTA_NO2IL12_PATHWAY		17	-0.3592376	-1.0087575	0.423729	0.6478414	1	4508	tags=41%, list=21%, signal=52%
BYSTRYKH_HEMATOPOIESIS_STEM_CELL_AND_BRAIN_QTL	BYSTRYKH_HEMATOPOIESIS_STEM_CELL_AND_BRAIN_QTL_CIS		59	-0.2720259	-1.0084552	0.446429	0.6479723	1	4721	tags=22%, list=22%, signal=28%
KEGG_PYRUVATE_METABOLISM	KEGG_PYRUVATE_METABOLISM		39	-0.3029753	-1.0079881	0.442308	0.64867514	1	4594	tags=31%, list=21%, signal=39%
KEGG_OOCYTE_MEIOSIS	KEGG_OOCYTE_MEIOSIS		107	-0.2502793	-1.0078855	0.428571	0.6484564	1	5560	tags=27%, list=25%, signal=36%
REACTOME_INTEGRIN_ALPHAIIBBETA3_SIGNALING	REACTOME_INTEGRIN_ALPHAIIBBETA3_SIGNALING		23	-0.360734	-1.0078406	0.37037	0.64804065	1	6862	tags=48%, list=31%, signal=70%
KEGG_PORPHYRIN_AND_CHLOROPHYLL_METABOLISM	KEGG_PORPHYRIN_AND_CHLOROPHYLL_METABOLISM		30	-0.3198793	-1.0075473	0.428571	0.6484015	1	7821	tags=53%, list=36%, signal=83%
RICKMAN_TUMOR_DIFFERENTIATED_WELL_VS_POORLY_U	RICKMAN_TUMOR_DIFFERENTIATED_WELL_VS_POORLY_UP		222	-0.2253428	-1.0057856	0.454545	0.65281504	1	6744	tags=30%, list=31%, signal=43%
KEGG_VALINE_LEUCINE_AND_ISOLEUCINE_DEGRADATION	KEGG_VALINE_LEUCINE_AND_ISOLEUCINE_DEGRADATION		44	-0.2970163	-1.0054789	0.380952	0.6531907	1	5022	tags=30%, list=23%, signal=38%
LIU_TARGETS_OF_VMYB_VS_CMYB_UP	LIU_TARGETS_OF_VMYB_VS_CMYB_UP		17	-0.3627273	-1.0047716	0.407407	0.6547452	1	1475	tags=12%, list=7%, signal=13%
SNIJDERS_AMPLIFIED_IN_HEAD_AND_NECK_TUMORS	SNIJDERS_AMPLIFIED_IN_HEAD_AND_NECK_TUMORS		32	-0.3010567	-1.0046208	0.396552	0.65465623	1	6288	tags=47%, list=29%, signal=66%
ODONNELL_TFRC_TARGETS_UP	ODONNELL_TFRC_TARGETS_UP		348	-0.2173655	-1.0042771	0.426471	0.65506077	1	4748	tags=25%, list=22%, signal=31%
BIOCARTA_ATRBRCA_PATHWAY	BIOCARTA_ATRBRCA_PATHWAY		21	-0.3538194	-1.004237	0.482143	0.6547057	1	4881	tags=29%, list=22%, signal=37%
ODONNELL_METASTASIS_UP	ODONNELL_METASTASIS_UP		75	-0.2613972	-1.0030509	0.40678	0.65720713	1	3240	tags=17%, list=15%, signal=20%
KEGG_NOTCH_SIGNALING_PATHWAY	KEGG_NOTCH_SIGNALING_PATHWAY		47	-0.2925122	-1.0025312	0.483333	0.6582935	1	3242	tags=19%, list=15%, signal=22%
YAO_TEMPORAL_RESPONSE_TO_PROGESTERONE_CLUSTER	YAO_TEMPORAL_RESPONSE_TO_PROGESTERONE_CLUSTER_12		72	-0.257796	-1.0014193	0.471698	0.660576	1	6739	tags=36%, list=31%, signal=52%
BIOCARTA_CDC42RAC_PATHWAY	BIOCARTA_CDC42RAC_PATHWAY		16	-0.3808921	-1.0013512	0.490909	0.66023743	1	7347	tags=50%, list=34%, signal=75%
OSWALD_HEMATOPOIETIC_STEM_CELL_IN_COLLAGEN_GE	OSWALD_HEMATOPOIETIC_STEM_CELL_IN_COLLAGEN_GEL_UP		215	-0.2202325	-1.0010431	0.45	0.66057503	1	5001	tags=24%, list=23%, signal=31%
PROVENZANI_METASTASIS_UP	PROVENZANI_METASTASIS_UP		187	-0.2237368	-1.0008203	0.409091	0.6607479	1	7037	tags=36%, list=32%, signal=53%
GRATIAS_RETINOBLASTOMA_16Q24	GRATIAS_RETINOBLASTOMA_16Q24		17	-0.3720784	-1.0006227	0.457627	0.6608381	1	3041	tags=24%, list=14%, signal=27%
KEGG_PRIMARY_IMMUNODEFICIENCY	KEGG_PRIMARY_IMMUNODEFICIENCY		35	-0.3035319	-0.999204	0.442623	0.66397333	1	2928	tags=23%, list=13%, signal=26%
REACTOME_REGULATION_OF_INSULIN_SECRETION	REACTOME_REGULATION_OF_INSULIN_SECRETION		202	-0.2197529	-0.9989721	0.396825	0.66410404	1	5221	tags=27%, list=24%, signal=35%
SCHLOSSER_MYC_TARGETS_AND_SERUM_RESPONSE_DN	SCHLOSSER_MYC_TARGETS_AND_SERUM_RESPONSE_DN		47	-0.2926294	-0.9986382	0.482143	0.6646227	1	2440	tags=11%, list=11%, signal=12%
CHIANG_LIVER_CANCER_SUBCLASS_PROLIFERATION_DN	CHIANG_LIVER_CANCER_SUBCLASS_PROLIFERATION_DN		143	-0.2373232	-0.9985788	0.465517	0.66428304	1	4564	tags=24%, list=21%, signal=30%
TOOKER_RESPONSE_TO_BEXAROTENE_DN	TOOKER_RESPONSE_TO_BEXAROTENE_DN		75	-0.2604038	-0.9983115	0.466667	0.66449255	1	4829	tags=29%, list=22%, signal=37%
REACTOME_SIGNALLING_TO_ERKS	REACTOME_SIGNALLING_TO_ERKS		34	-0.3038591	-0.9982578	0.403846	0.6641334	1	6090	tags=41%, list=28%, signal=57%
REACTOME_PLC_GAMMA1_SIGNALLING	REACTOME_PLC_GAMMA1_SIGNALLING		34	-0.2930704	-0.9980838	0.483333	0.6641601	1	5767	tags=35%, list=26%, signal=48%
TONKS_TARGETS_OF_RUNX1_RUNX1T1_FUSION_GRANUL	TONKS_TARGETS_OF_RUNX1_RUNX1T1_FUSION_GRANULOCYTE		53	-0.2852118	-0.9978835	0.483871	0.664125	1	4161	tags=25%, list=19%, signal=30%
WEST_ADRENOCORTICAL_CARCINOMA_VS_ADENOMA_DN	WEST_ADRENOCORTICAL_CARCINOMA_VS_ADENOMA_DN		19	-0.3651991	-0.9973722	0.454545	0.66483307	1	4938	tags=37%, list=23%, signal=48%
BILBAN_B_CLL_LPL_DN	BILBAN_B_CLL_LPL_DN		39	-0.3074634	-0.9964184	0.509434	0.66682744	1	3833	tags=23%, list=17%, signal=28%
KEGG_NATURAL_KILLER_CELL_MEDIATED_CYTOTOXICITY	KEGG_NATURAL_KILLER_CELL_MEDIATED_CYTOTOXICITY		107	-0.2515909	-0.9960575	0.457627	0.66717607	1	4780	tags=28%, list=22%, signal=36%
CHEBOTAEV_GR_TARGETS_UP	CHEBOTAEV_GR_TARGETS_UP		71	-0.2693498	-0.9957127	0.425926	0.6674729	1	2734	tags=17%, list=12%, signal=19%
BROWNE_HCMV_INFECTION_18HR_UP	BROWNE_HCMV_INFECTION_18HR_UP		174	-0.2296106	-0.9955614	0.405797	0.66735697	1	4756	tags=24%, list=22%, signal=30%
BIOCARTA_BCR_PATHWAY	BIOCARTA_BCR_PATHWAY		34	-0.3058463	-0.9950563	0.488372	0.66821253	1	4980	tags=32%, list=23%, signal=42%
CUI_GLUCOSE_DEPRIVATION	CUI_GLUCOSE_DEPRIVATION		46	-0.3012122	-0.9947382	0.45	0.66851014	1	6199	tags=41%, list=28%, signal=57%
KEGG_REGULATION_OF_ACTIN_CYTOSKELETON	KEGG_REGULATION_OF_ACTIN_CYTOSKELETON		209	-0.2313443	-0.9938296	0.469697	0.67064947	1	5187	tags=28%, list=24%, signal=37%
MASSARWEH_RESPONSE_TO_ESTRADIOL	MASSARWEH_RESPONSE_TO_ESTRADIOL		59	-0.2733351	-0.9938121	0.480769	0.6702172	1	6004	tags=34%, list=27%, signal=47%
CAFFAREL_RESPONSE_TO_THC_24HR_5_UP	CAFFAREL_RESPONSE_TO_THC_24HR_5_UP		25	-0.3086567	-0.9931302	0.458333	0.6714255	1	1806	tags=16%, list=8%, signal=17%
GOUYER_TATI_TARGETS_DN	GOUYER_TATI_TARGETS_DN		17	-0.3591564	-0.9930618	0.423077	0.6711246	1	6827	tags=47%, list=31%, signal=68%
RICKMAN_TUMOR_DIFFERENTIATED_MODERATELY_VS_PO	RICKMAN_TUMOR_DIFFERENTIATED_MODERATELY_VS_POORLY		41	-0.3018262	-0.993061	0.413043	0.67064345	1	7487	tags=49%, list=34%, signal=74%
YAUCH_HEDGEHOG_SIGNALING_PARACRINE_UP	YAUCH_HEDGEHOG_SIGNALING_PARACRINE_UP		147	-0.2314303	-0.9930553	0.451613	0.6701732	1	4658	tags=24%, list=21%, signal=31%
BIOCARTA_GH_PATHWAY	BIOCARTA_GH_PATHWAY		27	-0.3196898	-0.992352	0.446429	0.6716009	1	4961	tags=33%, list=23%, signal=43%
BANDRES_RESPONSE_TO_CARMUSTIN_WITHOUT_MGMT_4	BANDRES_RESPONSE_TO_CARMUSTIN_WITHOUT_MGMT_48HR_		24	-0.3251588	-0.9916987	0.508772	0.67286843	1	4391	tags=29%, list=20%, signal=36%
ICHIBA_GRAFT_VERSUS_HOST_DISEASE_35D_DN	ICHIBA_GRAFT_VERSUS_HOST_DISEASE_35D_DN		55	-0.2699873	-0.9903365	0.519231	0.67577076	1	2581	tags=15%, list=12%, signal=16%
DIRMEIER_LMP1_RESPONSE_EARLY	DIRMEIER_LMP1_RESPONSE_EARLY		53	-0.2766321	-0.9896187	0.464286	0.67731345	1	6029	tags=32%, list=27%, signal=44%
GOTTWEIN_TARGETS_OF_KSHV_MIR_K12_11	GOTTWEIN_TARGETS_OF_KSHV_MIR_K12_11		60	-0.2528184	-0.9893552	0.377359	0.67769104	1	3908	tags=22%, list=18%, signal=26%
TAKEDA_TARGETS_OF_NUP98_HOXA9_FUSION_16D_UP	TAKEDA_TARGETS_OF_NUP98_HOXA9_FUSION_16D_UP		152	-0.2274796	-0.9885976	0.45	0.67912173	1	3037	tags=16%, list=14%, signal=18%
BIOCARTA_TH1TH2_PATHWAY	BIOCARTA_TH1TH2_PATHWAY		19	-0.3537307	-0.9883211	0.469388	0.67948914	1	4508	tags=32%, list=21%, signal=40%
AMIT_SERUM_RESPONSE_20_MCF10A	AMIT_SERUM_RESPONSE_20_MCF10A		20	-0.3440098	-0.9874284	0.392857	0.68127775	1	4002	tags=25%, list=18%, signal=31%
BROWNE_HCMV_INFECTION_1HR_DN	BROWNE_HCMV_INFECTION_1HR_DN		207	-0.2242729	-0.9873912	0.540984	0.6809115	1	4329	tags=21%, list=20%, signal=26%

FERREIRA_EWINGS_SARCOMA_UNSTABLE_VS_STABLE_UP	FERREIRA_EWINGS_SARCOMA_UNSTABLE_VS_STABLE_UP		143	-0.2295247	-0.9873723	0.492064	0.6804773	1	5312	tags=29%, list=24%, signal=39%
CHOW_RASSF1_TARGETS_DN	CHOW_RASSF1_TARGETS_DN		29	-0.3109906	-0.986617	0.465517	0.68187404	1	3852	tags=28%, list=18%, signal=33%
FARMER_BREAST_CANCER_CLUSTER_6	FARMER_BREAST_CANCER_CLUSTER_6		15	-0.3930496	-0.9865482	0.537037	0.68150926	1	914	tags=13%, list=4%, signal=14%
BENPORATH_NOS_TARGETS	BENPORATH_NOS_TARGETS		171	-0.2231222	-0.9862924	0.448276	0.6816926	1	4480	tags=24%, list=20%, signal=30%
RASHI_RESPONSE_TO_IONIZING_RADIATION_3	RASHI_RESPONSE_TO_IONIZING_RADIATION_3		45	-0.2816092	-0.9856967	0.380952	0.6827222	1	6627	tags=36%, list=30%, signal=51%
REACTOME_REGULATION_OF_ORNITHINE_DECARBOXYLAS	REACTOME_REGULATION_OF_ORNITHINE_DECARBOXYLASE		47	-0.2848726	-0.9853705	0.442308	0.6831936	1	6834	tags=38%, list=31%, signal=56%
HOEBEKE_LYMPHOID_STEM_CELL_DN	HOEBEKE_LYMPHOID_STEM_CELL_DN		83	-0.2519033	-0.9848019	0.508475	0.68432254	1	4029	tags=19%, list=18%, signal=24%
KAYO_AGING_MUSCLE_DN	KAYO_AGING_MUSCLE_DN		105	-0.2445208	-0.9847177	0.491525	0.68404627	1	5477	tags=24%, list=25%, signal=32%
IVANOVA_HEMATOPOIESIS_INTERMEDIATE_PROGENITOR	IVANOVA_HEMATOPOIESIS_INTERMEDIATE_PROGENITOR		30	-0.3115519	-0.9845387	0.530612	0.68401843	1	5387	tags=33%, list=25%, signal=44%
REACTOME_STABILIZATION_OF_P53	REACTOME_STABILIZATION_OF_P53		46	-0.2953661	-0.9822188	0.508475	0.6896226	1	6834	tags=37%, list=31%, signal=54%
JIANG_TIP30_TARGETS_DN	JIANG_TIP30_TARGETS_DN		23	-0.320412	-0.979835	0.525424	0.69580066	1	4274	tags=26%, list=19%, signal=32%
BIOCARTA_DC_PATHWAY	BIOCARTA_DC_PATHWAY		22	-0.3273628	-0.9798271	0.490909	0.69532984	1	3386	tags=27%, list=15%, signal=32%
REACTOME_DOWNSTREAM_EVENTS_IN_GPCR_SIGNALING	REACTOME_DOWNSTREAM_EVENTS_IN_GPCR_SIGNALING		423	-0.2021834	-0.9791101	0.565217	0.69667995	1	4181	tags=21%, list=19%, signal=25%
AKL_HTLV1_INFECTION_UP	AKL_HTLV1_INFECTION_UP		27	-0.3068658	-0.9783796	0.392157	0.6981471	1	1960	tags=15%, list=9%, signal=16%
REACTOME_TCR_SIGNALING	REACTOME_TCR_SIGNALING		53	-0.2667907	-0.9778409	0.528302	0.69927096	1	3425	tags=21%, list=16%, signal=25%
NIKOLSKY_BREAST_CANCER_17Q11_Q21_AMPLICON	NIKOLSKY_BREAST_CANCER_17Q11_Q21_AMPLICON		112	-0.2279574	-0.9777666	0.482759	0.6989462	1	5016	tags=28%, list=23%, signal=36%
KEGG_COLORECTAL_CANCER	KEGG_COLORECTAL_CANCER		62	-0.2591699	-0.9771977	0.517857	0.6999939	1	1486	tags=13%, list=7%, signal=14%
CREIGHTON_ENDOCRINE_THERAPY_RESISTANCE_4	CREIGHTON_ENDOCRINE_THERAPY_RESISTANCE_4		281	-0.2124383	-0.9766032	0.535211	0.7010398	1	3925	tags=19%, list=18%, signal=22%
KAUFFMANN_DNA_REPAIR_GENES	KAUFFMANN_DNA_REPAIR_GENES		197	-0.2201849	-0.976572	0.552239	0.7006171	1	4881	tags=23%, list=22%, signal=30%
TOOKER_GEMCITABINE_RESISTANCE_UP	TOOKER_GEMCITABINE_RESISTANCE_UP		75	-0.2604038	-0.9761945	0.444444	0.70111907	1	4829	tags=29%, list=22%, signal=37%
ZHANG_ANTIVIRAL_RESPONSE_TO_RIBAVIRIN_UP	ZHANG_ANTIVIRAL_RESPONSE_TO_RIBAVIRIN_UP		22	-0.3174706	-0.9759551	0.483333	0.70115864	1	5948	tags=36%, list=27%, signal=50%
KEGG_MELANOGENESIS	KEGG_MELANOGENESIS		98	-0.2487455	-0.975193	0.516667	0.7026659	1	5356	tags=33%, list=24%, signal=43%
GAUSSMANN_MLL_AF4_FUSION_TARGETS_B_UP	GAUSSMANN_MLL_AF4_FUSION_TARGETS_B_UP		18	-0.3445169	-0.9750298	0.525424	0.7024784	1	762	tags=17%, list=3%, signal=17%
LU_IL4_SIGNALING	LU_IL4_SIGNALING		61	-0.2711579	-0.9740565	0.537037	0.7046858	1	4448	tags=30%, list=20%, signal=37%
NAM_FXYD5_TARGETS_DN	NAM_FXYD5_TARGETS_DN		18	-0.3396436	-0.9729413	0.5	0.7070479	1	6032	tags=39%, list=28%, signal=54%
CADWELL_ATG16L1_TARGETS_DN	CADWELL_ATG16L1_TARGETS_DN		60	-0.2612409	-0.9728212	0.607143	0.7068974	1	4101	tags=25%, list=19%, signal=31%
OHM_METHYLATED_IN_ADULT_CANCERS	OHM_METHYLATED_IN_ADULT_CANCERS		27	-0.3223814	-0.9726444	0.489796	0.70691144	1	4752	tags=41%, list=22%, signal=52%
RUTELLA_RESPONSE_TO_HGF_DN	RUTELLA_RESPONSE_TO_HGF_DN		220	-0.2158832	-0.9721836	0.571429	0.707623	1	6175	tags=31%, list=28%, signal=43%
EHLERS_ANEUPLOIDY_UP	EHLERS_ANEUPLOIDY_UP		36	-0.3035193	-0.9719219	0.473684	0.7078434	1	3603	tags=22%, list=16%, signal=27%
KEGG_FRUCTOSE_AND_MANNOSE_METABOLISM	KEGG_FRUCTOSE_AND_MANNOSE_METABOLISM		34	-0.320185	-0.9718544	0.530612	0.707526	1	7950	tags=47%, list=36%, signal=74%
REACTOME_PYRUVATE_METABOLISM	REACTOME_PYRUVATE_METABOLISM		18	-0.3538223	-0.9715927	0.581818	0.7077274	1	3735	tags=22%, list=17%, signal=27%
GARGALOVIC_RESPONSE_TO_OXIDIZED_PHOSPHOLIPIDS	GARGALOVIC_RESPONSE_TO_OXIDIZED_PHOSPHOLIPIDS_TURC		67	-0.2527143	-0.971291	0.52459	0.7079941	1	2628	tags=13%, list=12%, signal=15%
DIRMEIER_LMP1_RESPONSE_LATE_UP	DIRMEIER_LMP1_RESPONSE_LATE_UP		46	-0.2832316	-0.9703583	0.482759	0.70998895	1	4108	tags=26%, list=19%, signal=32%
BIOCARTA_BCELLSURVIVAL_PATHWAY	BIOCARTA_BCELLSURVIVAL_PATHWAY		16	-0.3700907	-0.9696153	0.571429	0.71162236	1	2345	tags=25%, list=11%, signal=28%
OSWALD_HEMATOPOIETIC_STEM_CELL_IN_COLLAGEN_GE	OSWALD_HEMATOPOIETIC_STEM_CELL_IN_COLLAGEN_GEL_DN		215	-0.2202325	-0.9688969	0.485714	0.7128529	1	5001	tags=24%, list=23%, signal=31%
HOSHIDA_LIVER_CANCER_LATE_RECURRENCE_DN	HOSHIDA_LIVER_CANCER_LATE_RECURRENCE_DN		65	-0.2646989	-0.9674519	0.509091	0.7163051	1	4782	tags=29%, list=22%, signal=37%
KEGG_TGF_BETA_SIGNALING_PATHWAY	KEGG_TGF_BETA_SIGNALING_PATHWAY		85	-0.2389484	-0.9670876	0.482759	0.7167515	1	4227	tags=22%, list=19%, signal=28%
LEE_EARLY_T_LYMPHOCYTE_DN	LEE_EARLY_T_LYMPHOCYTE_DN		39	-0.2902157	-0.9667009	0.542373	0.7171618	1	3813	tags=28%, list=17%, signal=34%
BIOCARTA_IL2_PATHWAY	BIOCARTA_IL2_PATHWAY		22	-0.3323805	-0.9663974	0.519231	0.71742296	1	4961	tags=36%, list=23%, signal=47%
KEGG_NEUROTROPHIN_SIGNALING_PATHWAY	KEGG_NEUROTROPHIN_SIGNALING_PATHWAY		125	-0.2264513	-0.9656915	0.448276	0.71868503	1	5074	tags=26%, list=23%, signal=33%
TONKS_TARGETS_OF_RUNX1_RUNX1T1_FUSION_HSC_UP	TONKS_TARGETS_OF_RUNX1_RUNX1T1_FUSION_HSC_UP		173	-0.2247531	-0.9650431	0.483333	0.7198224	1	3787	tags=20%, list=17%, signal=24%
KEGG_UBIQUITIN_MEDIATED_PROTEOLYSIS	KEGG_UBIQUITIN_MEDIATED_PROTEOLYSIS		131	-0.2238432	-0.9639373	0.616667	0.722335	1	4840	tags=21%, list=22%, signal=26%
OSADA_ASCL1_TARGETS_UP	OSADA_ASCL1_TARGETS_UP		44	-0.2875396	-0.9635994	0.578947	0.72284496	1	1832	tags=16%, list=8%, signal=17%
BIOCARTA_HCMV_PATHWAY	BIOCARTA_HCMV_PATHWAY		17	-0.3439691	-0.9635073	0.6	0.72264487	1	4961	tags=29%, list=23%, signal=38%
TOMLINS_PROSTATE_CANCER_DN	TOMLINS_PROSTATE_CANCER_DN		39	-0.2913606	-0.9632784	0.48	0.72279584	1	4971	tags=31%, list=23%, signal=40%
SANA_RESPONSE_TO_IFNG_DN	SANA_RESPONSE_TO_IFNG_DN		76	-0.2504409	-0.9627568	0.516667	0.72356284	1	6380	tags=34%, list=29%, signal=48%
WHITEHURST_PACLITAXEL_SENSITIVITY	WHITEHURST_PACLITAXEL_SENSITIVITY		30	-0.3159481	-0.9624358	0.468085	0.723867	1	3678	tags=30%, list=17%, signal=36%
MONNIER_POSTRADIATION_TUMOR_ESCAPE_UP	MONNIER_POSTRADIATION_TUMOR_ESCAPE_UP		357	-0.2056619	-0.961333	0.569444	0.72645557	1	5892	tags=29%, list=27%, signal=39%
REACTOME_LAGGING_STRAND_SYNTHESIS	REACTOME_LAGGING_STRAND_SYNTHESIS		20	-0.3544641	-0.9606072	0.54	0.7280727	1	4086	tags=30%, list=19%, signal=37%
BOYLAN_MULTIPLE_MYELOMA_D_UP	BOYLAN_MULTIPLE_MYELOMA_D_UP		82	-0.2423212	-0.9599444	0.529412	0.729337	1	6575	tags=35%, list=30%, signal=50%
WOTTON_RUNX_TARGETS_DN	WOTTON_RUNX_TARGETS_DN		27	-0.3003961	-0.9594721	0.529412	0.7300742	1	5422	tags=37%, list=25%, signal=49%
REACTOME_G_PROTEIN_ACTIVATION	REACTOME_G_PROTEIN_ACTIVATION		28	-0.2991764	-0.9586276	0.518519	0.7317672	1	3226	tags=21%, list=15%, signal=25%
KEGG_HISTIDINE_METABOLISM	KEGG_HISTIDINE_METABOLISM		29	-0.2932723	-0.9584926	0.492064	0.7315645	1	4418	tags=28%, list=20%, signal=35%
REACTOME_LYSOSOME_VESICLE_BIOGENESIS	REACTOME_LYSOSOME_VESICLE_BIOGENESIS		24	-0.334254	-0.9581327	0.488372	0.7319663	1	4967	tags=38%, list=23%, signal=48%
SEMENZA_HIF1_TARGETS	SEMENZA_HIF1_TARGETS		34	-0.2938152	-0.9577473	0.571429	0.73246485	1	1376	tags=12%, list=6%, signal=13%
ZHOU_INFLAMMATORY_RESPONSE_LIVE_DN	ZHOU_INFLAMMATORY_RESPONSE_LIVE_DN		344	-0.2031163	-0.9575517	0.552239	0.73249155	1	5990	tags=29%, list=27%, signal=40%
REACTOME_DIABETES_PATHWAYS	REACTOME_DIABETES_PATHWAYS		367	-0.2004821	-0.9574635	0.623188	0.73211604	1	5477	tags=23%, list=25%, signal=31%
BONOME_OVARIAN_CANCER_SURVIVAL_OPTIMAL_DEBULK	BONOME_OVARIAN_CANCER_SURVIVAL_OPTIMAL_DEBULKING		226	-0.2056354	-0.9572651	0.507692	0.7321656	1	5068	tags=25%, list=23%, signal=32%
KAAB_HEART_ATRIUM_VS_VENTRICLE_UP	KAAB_HEART_ATRIUM_VS_VENTRICLE_UP		237	-0.2145968	-0.95688	0.607143	0.732701	1	3770	tags=19%, list=17%, signal=23%
ICHIBA_GRAFT_VERSUS_HOST_DISEASE_D7_DN	ICHIBA_GRAFT_VERSUS_HOST_DISEASE_D7_DN		46	-0.2640109	-0.9562588	0.611111	0.7337694	1	2815	tags=20%, list=13%, signal=22%
BIOCARTA_RACCYCD_PATHWAY	BIOCARTA_RACCYCD_PATHWAY		26	-0.3275026	-0.9556713	0.520833	0.73493767	1	6663	tags=42%, list=30%, signal=61%
IVANOVA_HEMATOPOIESIS_STEM_CELL	IVANOVA_HEMATOPOIESIS_STEM_CELL		82	-0.2556872	-0.9556481	0.392857	0.7344652	1	3305	tags=17%, list=15%, signal=20%
LIEN_BREAST_CARCINOMA_METAPLASTIC	LIEN_BREAST_CARCINOMA_METAPLASTIC		32	-0.3031886	-0.9550528	0.574074	0.7356866	1	3852	tags=28%, list=18%, signal=34%
GARGALOVIC_RESPONSE_TO_OXIDIZED_PHOSPHOLIPIDS	GARGALOVIC_RESPONSE_TO_OXIDIZED_PHOSPHOLIPIDS_BLAC		31	-0.29655	-0.9549539	0.534483	0.735539	1	7281	tags=48%, list=33%, signal=72%

BIOCARTA_ERK5_PATHWAY	BIOCARTA_ERK5_PATHWAY		17	-0.3352721	-0.954253	0.52381	0.7370892	1	4961	tags=35%, list=23%, signal=46%
NIKOLSKY_BREAST_CANCER_11Q12_Q14_AMPLICON	NIKOLSKY_BREAST_CANCER_11Q12_Q14_AMPLICON		146	-0.2176651	-0.9541412	0.567164	0.73683655	1	5394	tags=32%, list=25%, signal=42%
GINESTIER_BREAST_CANCER_20Q13_AMPLIFICATION_DN	GINESTIER_BREAST_CANCER_20Q13_AMPLIFICATION_DN		157	-0.2226116	-0.9537175	0.596774	0.7373352	1	5064	tags=25%, list=23%, signal=32%
LIAO_HAVE_SOX4_BINDING_SITES	LIAO_HAVE_SOX4_BINDING_SITES		38	-0.2843819	-0.9535044	0.571429	0.7373021	1	5221	tags=29%, list=24%, signal=38%
ZHOU_INFLAMMATORY_RESPONSE_LPS_DN	ZHOU_INFLAMMATORY_RESPONSE_LPS_DN		344	-0.2031163	-0.9532999	0.606557	0.7373521	1	5990	tags=29%, list=27%, signal=40%
CHIANG_LIVER_CANCER_SUBCLASS_POLYSOMY7_DN	CHIANG_LIVER_CANCER_SUBCLASS_POLYSOMY7_DN		20	-0.3247115	-0.9528235	0.576923	0.7382222	1	2577	tags=15%, list=12%, signal=17%
BIOCARTA_PTEN_PATHWAY	BIOCARTA_PTEN_PATHWAY		18	-0.3340447	-0.9523507	0.563636	0.73898757	1	5330	tags=39%, list=24%, signal=51%
GALE_APL_WITH_FLT3_MUTATED_UP	GALE_APL_WITH_FLT3_MUTATED_UP		53	-0.2762804	-0.9506396	0.538462	0.7432641	1	4299	tags=23%, list=20%, signal=28%
DANG_MYC_TARGETS_UP	DANG_MYC_TARGETS_UP		125	-0.230078	-0.950572	0.537313	0.7429887	1	5530	tags=26%, list=25%, signal=35%
REACTOME_SIGNALLING_BY_NGF	REACTOME_SIGNALLING_BY_NGF		214	-0.2146481	-0.9503292	0.546875	0.74302065	1	5837	tags=29%, list=27%, signal=39%
VALK_AML_CLUSTER_7	VALK_AML_CLUSTER_7		24	-0.3228205	-0.9494445	0.470588	0.74485403	1	6138	tags=29%, list=28%, signal=40%
NEWMAN_ERCC6_TARGETS_UP	NEWMAN_ERCC6_TARGETS_UP		23	-0.3177264	-0.9494309	0.545455	0.7443608	1	2530	tags=26%, list=12%, signal=29%
STARK_PREFRONTAL_CORTEX_22Q11_DELETION_DN	STARK_PREFRONTAL_CORTEX_22Q11_DELETION_DN		449	-0.1993871	-0.9492934	0.597222	0.74426615	1	6913	tags=33%, list=32%, signal=47%
FRASOR_RESPONSE_TO_ESTRADIOL_DN	FRASOR_RESPONSE_TO_ESTRADIOL_DN		59	-0.2646874	-0.9492734	0.509434	0.7438495	1	5646	tags=31%, list=26%, signal=41%
JAIN_NFKB_SIGNALING	JAIN_NFKB_SIGNALING		69	-0.2539645	-0.9486718	0.52459	0.74475574	1	6674	tags=39%, list=30%, signal=56%
LAU_APOPTOSIS_CDKN2A_UP	LAU_APOPTOSIS_CDKN2A_UP		53	-0.2463436	-0.9484846	0.491803	0.74476445	1	7167	tags=34%, list=33%, signal=50%
BIOCARTA_IL6_PATHWAY	BIOCARTA_IL6_PATHWAY		22	-0.3420986	-0.9472283	0.5	0.7475475	1	4961	tags=36%, list=23%, signal=47%
SCHLESINGER_H3K27ME3_IN_NORMAL_AND_METHYLATED	SCHLESINGER_H3K27ME3_IN_NORMAL_AND_METHYLATED_IN_(		23	-0.3079094	-0.9471997	0.535714	0.7471579	1	3196	tags=30%, list=15%, signal=36%
SAMOLS_TARGETS_OF_KHSV_MIRNAS_DN	SAMOLS_TARGETS_OF_KHSV_MIRNAS_DN		57	-0.2503766	-0.9470662	0.515152	0.74697524	1	3667	tags=23%, list=17%, signal=27%
LEE_LIVER_CANCER_E2F1_DN	LEE_LIVER_CANCER_E2F1_DN		57	-0.2669322	-0.947043	0.528302	0.7465499	1	4056	tags=21%, list=18%, signal=26%
LE_SKI_TARGETS_UP	LE_SKI_TARGETS_UP		16	-0.3572875	-0.9464808	0.566038	0.74768615	1	368	tags=13%, list=2%, signal=13%
KEGG_GLYCOLYSIS_GLUCONEOGENESIS	KEGG_GLYCOLYSIS_GLUCONEOGENESIS		57	-0.2599534	-0.9452626	0.6	0.7501666	1	2596	tags=16%, list=12%, signal=18%
IVANOVA_HEMATOPOIESIS_STEM_CELL_AND_PROGENITOR	IVANOVA_HEMATOPOIESIS_STEM_CELL_AND_PROGENITOR		211	-0.2137225	-0.9451816	0.666667	0.74987096	1	4554	tags=20%, list=21%, signal=25%
SENESE_HDAC1_TARGETS_UP	SENESE_HDAC1_TARGETS_UP		417	-0.2036388	-0.9451444	0.615385	0.7494725	1	4548	tags=19%, list=21%, signal=24%
GRESHOCK_CANCER_COPY_NUMBER_DN	GRESHOCK_CANCER_COPY_NUMBER_DN		330	-0.1993861	-0.9450043	0.640625	0.7492533	1	5235	tags=24%, list=24%, signal=31%
MODY_HIPPOCAMPUS_PRENATAL	MODY_HIPPOCAMPUS_PRENATAL		27	-0.319665	-0.9443438	0.534483	0.75058866	1	1732	tags=15%, list=8%, signal=16%
MASSARWEH_TAMOXIFEN_RESISTANCE_DN	MASSARWEH_TAMOXIFEN_RESISTANCE_DN		225	-0.2088142	-0.9434241	0.666667	0.7523129	1	5608	tags=27%, list=26%, signal=35%
REACTOME_THROMBOXANE_SIGNALLING_THROUGH_TP_R	REACTOME_THROMBOXANE_SIGNALLING_THROUGH_TP_RECEPT		23	-0.3217752	-0.9421165	0.5	0.75508654	1	3226	tags=22%, list=15%, signal=25%
REACTOME_CDT1_ASSOCIATION_WITH_THE_CDC6_ORC_C	REACTOME_CDT1_ASSOCIATION_WITH_THE_CDC6_ORC_ORIGI		52	-0.2599738	-0.9414799	0.457627	0.75621873	1	6834	tags=35%, list=31%, signal=50%
RIZ_ERYTHROID_DIFFERENTIATION_6HR	RIZ_ERYTHROID_DIFFERENTIATION_6HR		40	-0.2908847	-0.9408593	0.462687	0.7572473	1	1746	tags=15%, list=8%, signal=16%
FOSTER_INFLAMMATORY_RESPONSE_LPS_UP	FOSTER_INFLAMMATORY_RESPONSE_LPS_UP		163	-0.2205498	-0.9402719	0.538462	0.75834	1	4654	tags=26%, list=21%, signal=32%
REACTOME_STEROID_METABOLISM	REACTOME_STEROID_METABOLISM		61	-0.2549139	-0.9392752	0.539683	0.76019794	1	4684	tags=31%, list=21%, signal=39%
SIG_REGULATION_OF_THE_ACTIN_CYTOSKELETON_BY_RH	SIG_REGULATION_OF_THE_ACTIN_CYTOSKELETON_BY_RHO_GT		35	-0.2701131	-0.9390453	0.58	0.76032823	1	3425	tags=17%, list=16%, signal=20%
STEIN_ESRRA_TARGETS_DN	STEIN_ESRRA_TARGETS_DN		98	-0.2385861	-0.9384416	0.6	0.76126957	1	7196	tags=40%, list=33%, signal=59%
BIOCARTA_PTDINS_PATHWAY	BIOCARTA_PTDINS_PATHWAY		23	-0.315618	-0.9377253	0.553571	0.7625934	1	7632	tags=57%, list=35%, signal=87%
ZHAN_MULTIPLE_MYELOMA_SUBGROUPS	ZHAN_MULTIPLE_MYELOMA_SUBGROUPS		27	-0.3088026	-0.9374638	0.516667	0.76296574	1	4188	tags=22%, list=19%, signal=27%
BANDRES_RESPONSE_TO_CARMUSTIN_MGMT_48HR_DN	BANDRES_RESPONSE_TO_CARMUSTIN_MGMT_48HR_DN		130	-0.2257896	-0.9362834	0.5625	0.7654036	1	4072	tags=20%, list=19%, signal=24%
REACTOME_NCAM1_INTERACTIONS	REACTOME_NCAM1_INTERACTIONS		43	-0.2643054	-0.9362243	0.483333	0.7650266	1	1853	tags=16%, list=8%, signal=18%
LIU_LIVER_CANCER	LIU_LIVER_CANCER		24	-0.3080229	-0.9353996	0.54717	0.76664853	1	6956	tags=46%, list=32%, signal=67%
ELVIDGE_HYPOXIA_BY_DMOG_DN	ELVIDGE_HYPOXIA_BY_DMOG_DN		59	-0.2511031	-0.9350046	0.535714	0.76717407	1	6877	tags=39%, list=31%, signal=57%
YANG_BREAST_CANCER_ESR1_BULK_UP	YANG_BREAST_CANCER_ESR1_BULK_UP		18	-0.3598078	-0.9347898	0.559322	0.7672125	1	5634	tags=33%, list=26%, signal=45%
SARRIO_EPITHELIAL_MESENCHYMAL_TRANSITION_UP	SARRIO_EPITHELIAL_MESENCHYMAL_TRANSITION_UP		15	-0.372057	-0.9347028	0.563636	0.7669662	1	5939	tags=47%, list=27%, signal=64%
REACTOME_G_BETA_GAMMA_SIGNALLING_THROUGH_PI3K	REACTOME_G_BETA_GAMMA_SIGNALLING_THROUGH_PI3KGAMI		25	-0.309431	-0.9343355	0.584906	0.7676458	1	3226	tags=24%, list=15%, signal=28%
VANTVEER_BREAST_CANCER_POOR_PROGNOSIS	VANTVEER_BREAST_CANCER_POOR_PROGNOSIS		46	-0.2650628	-0.9341964	0.481482	0.7675992	1	7187	tags=46%, list=33%, signal=68%
CHUNG_BLISTER_CYTOTOXICITY_DN	CHUNG_BLISTER_CYTOTOXICITY_DN		40	-0.2932378	-0.9338319	0.576271	0.76820534	1	4676	tags=25%, list=21%, signal=32%
POMEROY_MEDULLOBLASTOMA_PROGNOSIS_UP	POMEROY_MEDULLOBLASTOMA_PROGNOSIS_UP		34	-0.3016675	-0.9334536	0.559322	0.7686696	1	2194	tags=18%, list=10%, signal=20%
FIRESTEIN_CTNNB1_PATHWAY	FIRESTEIN_CTNNB1_PATHWAY		30	-0.2931604	-0.9331396	0.482143	0.7688844	1	6958	tags=50%, list=32%, signal=73%
CHANDRAN_METASTASIS_TOP50_UP	CHANDRAN_METASTASIS_TOP50_UP		16	-0.3290202	-0.9331304	0.529412	0.7684143	1	6779	tags=38%, list=31%, signal=54%
SU_LIVER	SU_LIVER		52	-0.2668138	-0.9331173	0.558824	0.76796335	1	2446	tags=17%, list=11%, signal=19%
HEIDENBLAD_AMPLICON_8Q24_UP	HEIDENBLAD_AMPLICON_8Q24_UP		26	-0.2877104	-0.9327804	0.517241	0.7683277	1	1512	tags=12%, list=7%, signal=12%
TSENG_ADIPOGENIC_POTENTIAL_UP	TSENG_ADIPOGENIC_POTENTIAL_UP		30	-0.286789	-0.9324861	0.595745	0.7685677	1	4688	tags=27%, list=21%, signal=34%
KEGG_AUTOIMMUNE_THYROID_DISEASE	KEGG_AUTOIMMUNE_THYROID_DISEASE		39	-0.2621816	-0.9320537	0.489796	0.76926357	1	3103	tags=21%, list=14%, signal=24%
GENTILE_UV_RESPONSE_CLUSTER_D9	GENTILE_UV_RESPONSE_CLUSTER_D9		19	-0.3382795	-0.9317913	0.509434	0.7693666	1	2634	tags=16%, list=12%, signal=18%
DEURIG_T_CELL_PROLYMPHOCYTIC_LEUKEMIA_DN	DEURIG_T_CELL_PROLYMPHOCYTIC_LEUKEMIA_DN		279	-0.2056054	-0.9311934	0.714286	0.77032715	1	3746	tags=16%, list=17%, signal=19%
NATSUME_RESPONSE_TO_INTERFERON_BETA_UP	NATSUME_RESPONSE_TO_INTERFERON_BETA_UP		67	-0.2501501	-0.9296063	0.490566	0.77425224	1	4342	tags=25%, list=20%, signal=32%
WANG_CISPLATIN_RESPONSE_AND_XPC_DN	WANG_CISPLATIN_RESPONSE_AND_XPC_DN		135	-0.2184908	-0.9294407	0.62069	0.7741495	1	5892	tags=31%, list=27%, signal=42%
PENG_RAPAMYCIN_RESPONSE_UP	PENG_RAPAMYCIN_RESPONSE_UP		37	-0.2726388	-0.9289488	0.563636	0.7749097	1	4218	tags=24%, list=19%, signal=30%
CHEN_LVAD_SUPPORT_OF_FAILING_HEART_DN	CHEN_LVAD_SUPPORT_OF_FAILING_HEART_DN		41	-0.2713153	-0.9288878	0.64	0.77458733	1	3266	tags=20%, list=15%, signal=23%
LU_TUMOR_ENDOTHELIAL_MARKERS_UP	LU_TUMOR_ENDOTHELIAL_MARKERS_UP		19	-0.3277852	-0.9284124	0.578947	0.7753116	1	2999	tags=21%, list=14%, signal=24%
PYEON_CANCER_HEAD_AND_NECK_VS_CERVICAL_UP	PYEON_CANCER_HEAD_AND_NECK_VS_CERVICAL_UP		170	-0.2213161	-0.9282221	0.629032	0.77522516	1	2957	tags=15%, list=13%, signal=17%
GRESHOCK_CANCER_COPY_NUMBER_UP	GRESHOCK_CANCER_COPY_NUMBER_UP		316	-0.1994152	-0.9279885	0.623188	0.7754329	1	4939	tags=22%, list=23%, signal=29%
COLDREN_GEFITINIB_RESISTANCE_UP	COLDREN_GEFITINIB_RESISTANCE_UP		85	-0.2392743	-0.9276995	0.55	0.77564204	1	4219	tags=25%, list=19%, signal=30%
ZHAN_MULTIPLE_MYELOMA_CD1_AND_CD2_DN	ZHAN_MULTIPLE_MYELOMA_CD1_AND_CD2_DN		50	-0.2593793	-0.9276302	0.666667	0.77537435	1	5281	tags=28%, list=24%, signal=37%
YAGI_AML_SURVIVAL	YAGI_AML_SURVIVAL		119	-0.2173247	-0.9273955	0.625	0.77545595	1	6608	tags=34%, list=30%, signal=49%

REACTOME_MITOCHONDRIAL_TRNA_AMINOACYLATION	REACTOME_MITOCHONDRIAL_TRNA_AMINOACYLATION		20	-0.3252601	-0.9273101	0.574074	0.7751245	1	5701	tags=40%, list=26%, signal=54%
GRADE_METASTASIS_DN	GRADE_METASTASIS_DN		41	-0.2662526	-0.9268448	0.660714	0.7758987	1	4436	tags=24%, list=20%, signal=31%
IWANAGA_CARCINOGENESIS_BY_KRAS_UP	IWANAGA_CARCINOGENESIS_BY_KRAS_UP		159	-0.2162056	-0.9268348	0.566667	0.77541167	1	5787	tags=33%, list=26%, signal=44%
RIZ_ERYTHROID_DIFFERENTIATION_HBZ	RIZ_ERYTHROID_DIFFERENTIATION_HBZ		37	-0.2666397	-0.9267547	0.6	0.775116	1	5741	tags=35%, list=26%, signal=48%
MASRI_RESISTANCE_TO_TAMOXIFEN_AND_AROMATASE_IN	MASRI_RESISTANCE_TO_TAMOXIFEN_AND_AROMATASE_INHIBIT		18	-0.3168426	-0.9267346	0.527273	0.7746859	1	2080	tags=11%, list=9%, signal=12%
SHEDDEN_LUNG_CANCER_GOOD_SURVIVAL_A5	SHEDDEN_LUNG_CANCER_GOOD_SURVIVAL_A5		64	-0.2578667	-0.9263587	0.612903	0.7751227	1	4941	tags=28%, list=23%, signal=36%
KEGG_ENDOMETRIAL_CANCER	KEGG_ENDOMETRIAL_CANCER		52	-0.2583916	-0.9241705	0.537313	0.78070974	1	5218	tags=33%, list=24%, signal=43%
SIG_BCR_SIGNALING_PATHWAY	SIG_BCR_SIGNALING_PATHWAY		46	-0.257741	-0.9214928	0.606557	0.7873867	1	5837	tags=37%, list=27%, signal=50%
LEE_NEURAL_CREST_STEM_CELL_DN	LEE_NEURAL_CREST_STEM_CELL_DN		110	-0.2217445	-0.9206185	0.701754	0.789307	1	3022	tags=15%, list=14%, signal=18%
MANN_RESPONSE_TO_AMIFOSTINE_UP	MANN_RESPONSE_TO_AMIFOSTINE_UP		19	-0.3230191	-0.9202263	0.516667	0.79000455	1	8167	tags=63%, list=37%, signal=101%
SESTO_RESPONSE_TO_UV_C8	SESTO_RESPONSE_TO_UV_C8		71	-0.2345311	-0.9193128	0.578125	0.7916689	1	4838	tags=21%, list=22%, signal=27%
LANDIS_BREAST_CANCER_PROGRESSION_UP	LANDIS_BREAST_CANCER_PROGRESSION_UP		44	-0.2822965	-0.9182508	0.534483	0.7937187	1	6441	tags=36%, list=29%, signal=51%
MARTINEZ_RESPONSE_TO_TRABECTEDIN_UP	MARTINEZ_RESPONSE_TO_TRABECTEDIN_UP		58	-0.2470134	-0.918097	0.619048	0.793523	1	3833	tags=21%, list=17%, signal=25%
REACTOME_GOLGI_ASSOCIATED_VESICLE_BIOGENESIS	REACTOME_GOLGI_ASSOCIATED_VESICLE_BIOGENESIS		53	-0.2561971	-0.9179449	0.706897	0.7933815	1	6626	tags=38%, list=30%, signal=54%
SENESE_HDAC1_TARGETS_DN	SENESE_HDAC1_TARGETS_DN		231	-0.2169555	-0.9178011	0.638889	0.79327047	1	5784	tags=29%, list=26%, signal=38%
REACTOME_NUCLEOTIDE_LIKE_PURINERGIC_RECEPTORS	REACTOME_NUCLEOTIDE_LIKE_PURINERGIC_RECEPTORS		15	-0.3698303	-0.9176927	0.617021	0.79309434	1	5908	tags=40%, list=27%, signal=55%
AGUIRRE_PANCREATIC_CANCER_COPY_NUMBER_DN	AGUIRRE_PANCREATIC_CANCER_COPY_NUMBER_DN		228	-0.1997424	-0.916879	0.654545	0.7945764	1	6806	tags=30%, list=31%, signal=43%
HEIDENBLAD_AMPLICON_12P11_12_DN	HEIDENBLAD_AMPLICON_12P11_12_DN		21	-0.3026441	-0.9160026	0.473684	0.79649824	1	7678	tags=62%, list=35%, signal=95%
MULLIGHAN_MLL_SIGNATURE_2_DN	MULLIGHAN_MLL_SIGNATURE_2_DN		257	-0.2000377	-0.9155504	0.704225	0.79714286	1	4965	tags=22%, list=23%, signal=28%
HU_ANGIOGENESIS_DN	HU_ANGIOGENESIS_DN		30	-0.2904232	-0.9152585	0.637931	0.79739743	1	6064	tags=40%, list=28%, signal=55%
REACTOME_SYNTHESIS_OF_GLYCOSYLPHOSPHATIDYLINOS	REACTOME_SYNTHESIS_OF_GLYCOSYLPHOSPHATIDYLINOSITOL		17	-0.3242828	-0.914394	0.5	0.7991205	1	8806	tags=53%, list=40%, signal=88%
SENESE_HDAC1_AND_HDAC2_TARGETS_UP	SENESE_HDAC1_AND_HDAC2_TARGETS_UP		211	-0.2131475	-0.9134228	0.698413	0.8010251	1	6178	tags=33%, list=28%, signal=46%
REACTOME_CLATHRIN_DERIVED_VESICLE_BUDDING	REACTOME_CLATHRIN_DERIVED_VESICLE_BUDDING		60	-0.2593295	-0.9132834	0.539683	0.80077016	1	6626	tags=37%, list=30%, signal=52%
CHIANG_LIVER_CANCER_SUBCLASS_UNANNOTATED_UP	CHIANG_LIVER_CANCER_SUBCLASS_UNANNOTATED_UP		24	-0.3079684	-0.9124901	0.603448	0.8021734	1	2785	tags=25%, list=13%, signal=29%
BORCZUK_MALIGNANT_MESOTHELIOMA_UP	BORCZUK_MALIGNANT_MESOTHELIOMA_UP		293	-0.2038834	-0.9116252	0.69697	0.80391294	1	5194	tags=22%, list=24%, signal=29%
HASLINGER_B_CLL_WITH_11Q23_DELETION	HASLINGER_B_CLL_WITH_11Q23_DELETION		19	-0.3189214	-0.9113595	0.603774	0.80408067	1	2676	tags=21%, list=12%, signal=24%
REACTOME_DOUBLE_STRAND_BREAK_REPAIR	REACTOME_DOUBLE_STRAND_BREAK_REPAIR		21	-0.2930977	-0.9105366	0.557692	0.8056443	1	1799	tags=14%, list=8%, signal=16%
HINATA_NFKB_TARGETS_KERATINOCYTE_DN	HINATA_NFKB_TARGETS_KERATINOCYTE_DN		15	-0.3363796	-0.9103367	0.581818	0.80554354	1	4597	tags=47%, list=21%, signal=59%
LANDEMAINE_LUNG_METASTASIS	LANDEMAINE_LUNG_METASTASIS		21	-0.3285289	-0.9098483	0.6	0.8064185	1	5336	tags=38%, list=24%, signal=50%
BOYAULT_LIVER_CANCER_SUBCLASS_G1_UP	BOYAULT_LIVER_CANCER_SUBCLASS_G1_UP		106	-0.2253052	-0.9096424	0.618182	0.8064226	1	3587	tags=17%, list=16%, signal=20%
KEGG_GALACTOSE_METABOLISM	KEGG_GALACTOSE_METABOLISM		25	-0.2913219	-0.9096136	0.64	0.80601525	1	6670	tags=52%, list=30%, signal=75%
DAVICIONI_RHABDOMYOSARCOMA_PAX_FOXO1_FUSION_	DAVICIONI_RHABDOMYOSARCOMA_PAX_FOXO1_FUSION_UP		62	-0.2393585	-0.9090633	0.706897	0.80691266	1	2057	tags=15%, list=9%, signal=16%
BIOCARTA_G2_PATHWAY	BIOCARTA_G2_PATHWAY		23	-0.3007744	-0.9090514	0.561404	0.8064332	1	4740	tags=30%, list=22%, signal=39%
BOYAULT_LIVER_CANCER_SUBCLASS_G123_DN	BOYAULT_LIVER_CANCER_SUBCLASS_G123_DN		50	-0.2448276	-0.9083769	0.642857	0.807659	1	4075	tags=30%, list=19%, signal=37%
LIU_VAV3_PROSTATE_CARCINOGENESIS_DN	LIU_VAV3_PROSTATE_CARCINOGENESIS_DN		17	-0.3169575	-0.9072733	0.535714	0.8099705	1	2161	tags=24%, list=10%, signal=26%
BIOCARTA_PDGF_PATHWAY	BIOCARTA_PDGF_PATHWAY		32	-0.2790172	-0.9066865	0.6	0.81073195	1	2947	tags=19%, list=13%, signal=22%
ST_B_CELL_ANTIGEN_RECEPTOR	ST_B_CELL_ANTIGEN_RECEPTOR		37	-0.2634765	-0.9063639	0.603175	0.8109681	1	5837	tags=43%, list=27%, signal=59%
DORSEY_GAB2_TARGETS	DORSEY_GAB2_TARGETS		22	-0.3166473	-0.9058691	0.62	0.81160635	1	3374	tags=36%, list=15%, signal=43%
BIOCARTA_CCR3_PATHWAY	BIOCARTA_CCR3_PATHWAY		23	-0.2992218	-0.904784	0.576923	0.8135253	1	2988	tags=22%, list=14%, signal=25%
DER_IFN_ALPHA_RESPONSE_UP	DER_IFN_ALPHA_RESPONSE_UP		53	-0.2576357	-0.9040263	0.65	0.8150446	1	2425	tags=17%, list=11%, signal=19%
MURAKAMI_UV_RESPONSE_6HR_DN	MURAKAMI_UV_RESPONSE_6HR_DN		19	-0.315625	-0.9037529	0.618182	0.8152681	1	4718	tags=32%, list=22%, signal=40%
AMUNDSON_RESPONSE_TO_ARSENITE	AMUNDSON_RESPONSE_TO_ARSENITE		170	-0.2021865	-0.9034863	0.69697	0.8152318	1	5251	tags=24%, list=24%, signal=31%
MCCABE_BOUND_BY_HOXC6	MCCABE_BOUND_BY_HOXC6		379	-0.1859484	-0.9023706	0.822581	0.81749505	1	5276	tags=26%, list=24%, signal=34%
YANG_BREAST_CANCER_ESR1_UP	YANG_BREAST_CANCER_ESR1_UP		25	-0.2976022	-0.9013809	0.611111	0.8194242	1	4787	tags=28%, list=22%, signal=36%
REACTOME_INTRINSIC_PATHWAY_FOR_APOPTOSIS	REACTOME_INTRINSIC_PATHWAY_FOR_APOPTOSIS		29	-0.2933758	-0.9008647	0.666667	0.8200281	1	6598	tags=34%, list=30%, signal=49%
FLOTHO_PEDIATRIC_ALL_THERAPY_RESPONSE_DN	FLOTHO_PEDIATRIC_ALL_THERAPY_RESPONSE_DN		27	-0.2988308	-0.9004568	0.5625	0.8207361	1	6814	tags=37%, list=31%, signal=54%
KORKOLA_YOLK_SAC_TUMOR_UP	KORKOLA_YOLK_SAC_TUMOR_UP		19	-0.3189365	-0.9003803	0.545455	0.8204485	1	8329	tags=47%, list=38%, signal=76%
WANG_SMARCE1_TARGETS_DN	WANG_SMARCE1_TARGETS_DN		318	-0.1933571	-0.8994948	0.743243	0.82200617	1	5539	tags=24%, list=25%, signal=32%
BIOCARTA_CREB_PATHWAY	BIOCARTA_CREB_PATHWAY		26	-0.2780106	-0.8986623	0.553571	0.8236289	1	5176	tags=35%, list=24%, signal=45%
ST_PHOSPHOINOSITIDE_3_KINASE_PATHWAY	ST_PHOSPHOINOSITIDE_3_KINASE_PATHWAY		33	-0.2830415	-0.8979117	0.627907	0.8248996	1	5837	tags=30%, list=27%, signal=41%
CHUANG_OXIDATIVE_STRESS_RESPONSE_UP	CHUANG_OXIDATIVE_STRESS_RESPONSE_UP		25	-0.2942229	-0.8978451	0.642857	0.824564	1	3337	tags=20%, list=15%, signal=24%
HUANG_FOXA2_TARGETS_UP	HUANG_FOXA2_TARGETS_UP		44	-0.2702406	-0.8973668	0.673469	0.8253992	1	3383	tags=20%, list=15%, signal=24%
PETRETTO_CARDIAC_HYPERTROPHY	PETRETTO_CARDIAC_HYPERTROPHY		33	-0.2644135	-0.8966441	0.666667	0.82661426	1	3905	tags=21%, list=18%, signal=26%
ZHOU_INFLAMMATORY_RESPONSE_FIMA_DN	ZHOU_INFLAMMATORY_RESPONSE_FIMA_DN		248	-0.2002595	-0.8966356	0.671429	0.8261024	1	5834	tags=31%, list=27%, signal=41%
FINETTI_BREAST_CANCERS_KINOME_BLUE	FINETTI_BREAST_CANCERS_KINOME_BLUE		19	-0.3139309	-0.8946552	0.557377	0.8306402	1	1382	tags=16%, list=6%, signal=17%
KUUSELO_PANCREATIC_CANCER_19Q13_AMPLIFICATION	KUUSELO_PANCREATIC_CANCER_19Q13_AMPLIFICATION		20	-0.3295777	-0.8945249	0.557377	0.8304001	1	7007	tags=45%, list=32%, signal=66%
WATANABE_COLON_CANCER_MSI_VS_MSS_DN	WATANABE_COLON_CANCER_MSI_VS_MSS_DN		64	-0.2266155	-0.8935747	0.583333	0.83207494	1	4198	tags=20%, list=19%, signal=25%
WANG_CLIM2_TARGETS_UP	WANG_CLIM2_TARGETS_UP		209	-0.1988859	-0.8932264	0.7	0.8324448	1	5184	tags=24%, list=24%, signal=31%
OUELLET_CULTURED_OVARIAN_CANCER_INVASIVE_VS_LM	OUELLET_CULTURED_OVARIAN_CANCER_INVASIVE_VS_LMP_DN		30	-0.2711429	-0.893207	0.640625	0.83195704	1	4305	tags=23%, list=20%, signal=29%
HALMOS_CEBPA_TARGETS_DN	HALMOS_CEBPA_TARGETS_DN		38	-0.2674711	-0.8930515	0.666667	0.8318656	1	3652	tags=29%, list=17%, signal=35%
BERENJENO_TRANSFORMED_BY_RHOA_REVERSIBLY_DN	BERENJENO_TRANSFORMED_BY_RHOA_REVERSIBLY_DN		27	-0.290297	-0.892685	0.583333	0.8322772	1	7409	tags=44%, list=34%, signal=67%
TAKEDA_TARGETS_OF_NUP98_HOXA9_FUSION_8D_UP	TAKEDA_TARGETS_OF_NUP98_HOXA9_FUSION_8D_UP		143	-0.2176112	-0.8926601	0.690909	0.8317731	1	2896	tags=16%, list=13%, signal=18%
REACTOME_SIGNAL_AMPLIFICATION	REACTOME_SIGNAL_AMPLIFICATION		31	-0.27614	-0.8920245	0.616667	0.8327771	1	5755	tags=32%, list=26%, signal=44%
SCHEIDEREIT_IKK_TARGETS	SCHEIDEREIT_IKK_TARGETS		16	-0.3339057	-0.8918285	0.5625	0.83272153	1	6284	tags=38%, list=29%, signal=53%

BASAKI_YBX1_TARGETS_DN	BASAKI_YBX1_TARGETS_DN		336	-0.1956885	-0.8912365	0.776316	0.8336757	1	5263	tags=24%, list=24%, signal=30%
REACTOME_NUCLEAR_RECEPTOR_TRANSCRIPTION_PATHW	REACTOME_NUCLEAR_RECEPTOR_TRANSCRIPTION_PATHWAY		50	-0.2591171	-0.8911827	0.631579	0.8332769	1	3118	tags=22%, list=14%, signal=26%
KIM_MYCN_AMPLIFICATION_TARGETS_UP	KIM_MYCN_AMPLIFICATION_TARGETS_UP		69	-0.2402999	-0.8904495	0.65	0.83445174	1	2249	tags=13%, list=10%, signal=14%
AMUNDSON_DNA_DAMAGE_RESPONSE_TP53	AMUNDSON_DNA_DAMAGE_RESPONSE_TP53		15	-0.3374594	-0.8903621	0.54386	0.83415854	1	4740	tags=27%, list=22%, signal=34%
KEGG_PYRIMIDINE_METABOLISM	KEGG_PYRIMIDINE_METABOLISM		94	-0.2259919	-0.8902975	0.701754	0.83377016	1	6421	tags=35%, list=29%, signal=49%
ST_GAQ_PATHWAY	ST_GAQ_PATHWAY		26	-0.2934303	-0.8894586	0.571429	0.835248	1	6284	tags=50%, list=29%, signal=70%
KEGG_PROSTATE_CANCER	KEGG_PROSTATE_CANCER		88	-0.2356979	-0.8892452	0.609375	0.8352097	1	5538	tags=32%, list=25%, signal=42%
KORKOLA_SEMINOMA_UP	KORKOLA_SEMINOMA_UP		42	-0.2621588	-0.88885	0.66129	0.83572453	1	4700	tags=24%, list=21%, signal=30%
KEGG_TERPENOID_BACKBONE_BIOSYNTHESIS	KEGG_TERPENOID_BACKBONE_BIOSYNTHESIS		15	-0.3519926	-0.8875358	0.641509	0.8383554	1	4604	tags=27%, list=21%, signal=34%
TIEN_INTESTINE_PROBIOTICS_6HR_DN	TIEN_INTESTINE_PROBIOTICS_6HR_DN		163	-0.2041317	-0.8874601	0.684211	0.83800864	1	5682	tags=26%, list=26%, signal=35%
JAZAG_TGFB1_SIGNALING_VIA_SMAD4_UP	JAZAG_TGFB1_SIGNALING_VIA_SMAD4_UP		97	-0.2273543	-0.8872017	0.660714	0.83816755	1	6223	tags=33%, list=28%, signal=46%
BERENJENO_ROCK_SIGNALING_NOT_VIA_RHOA_UP	BERENJENO_ROCK_SIGNALING_NOT_VIA_RHOA_UP		27	-0.2937761	-0.8871917	0.637931	0.837673	1	2832	tags=22%, list=13%, signal=25%
HUMMEL_BURKITTS_LYMPHOMA_DN	HUMMEL_BURKITTS_LYMPHOMA_DN		15	-0.3497682	-0.8860307	0.673469	0.84013325	1	4171	tags=33%, list=19%, signal=41%
HOOI_ST7_TARGETS_UP	HOOI_ST7_TARGETS_UP		76	-0.2399206	-0.8858311	0.704225	0.8400489	1	2692	tags=16%, list=12%, signal=18%
MOREIRA_RESPONSE_TO_TSA_DN	MOREIRA_RESPONSE_TO_TSA_DN		18	-0.3234022	-0.8843909	0.659574	0.84299016	1	1581	tags=17%, list=7%, signal=18%
SIG_CHEMOTAXIS	SIG_CHEMOTAXIS		44	-0.2533384	-0.8842256	0.684211	0.84286004	1	3865	tags=20%, list=18%, signal=25%
ALCALA_APOPTOSIS	ALCALA_APOPTOSIS		86	-0.2302129	-0.8826432	0.690909	0.84640837	1	6629	tags=35%, list=30%, signal=50%
MIDORIKAWA_AMPLIFIED_IN_LIVER_CANCER	MIDORIKAWA_AMPLIFIED_IN_LIVER_CANCER		37	-0.2622872	-0.8819978	0.526316	0.8474354	1	5301	tags=27%, list=24%, signal=36%
REACTOME_GS_ALPHA_MEDIATED_EVENTS_IN_GLUCAGON	REACTOME_GS_ALPHA_MEDIATED_EVENTS_IN_GLUCAGON_SIG		27	-0.3023684	-0.8813128	0.644444	0.8483821	1	5767	tags=41%, list=26%, signal=55%
SHIPP_DLBCL_CURED_VS_FATAL_UP	SHIPP_DLBCL_CURED_VS_FATAL_UP		35	-0.2623716	-0.8792023	0.607143	0.8527567	1	766	tags=11%, list=3%, signal=12%
MARZEC_IL2_SIGNALING_DN	MARZEC_IL2_SIGNALING_DN		33	-0.2703152	-0.8782153	0.666667	0.8545252	1	2165	tags=18%, list=10%, signal=20%
ZHU_CMV_24_HR_UP	ZHU_CMV_24_HR_UP		38	-0.2683207	-0.8782007	0.641791	0.85404944	1	6288	tags=39%, list=29%, signal=55%
REACTOME_PEROXISOMAL_LIPID_METABOLISM	REACTOME_PEROXISOMAL_LIPID_METABOLISM		20	-0.3021515	-0.8780795	0.596154	0.8538173	1	3006	tags=20%, list=14%, signal=23%
KEGG_ALANINE_ASPARTATE_AND_GLUTAMATE_METABOLIS	KEGG_ALANINE_ASPARTATE_AND_GLUTAMATE_METABOLISM		31	-0.2771162	-0.8774442	0.62069	0.8548156	1	3788	tags=29%, list=17%, signal=35%
WONG_PROTEASOME_GENE_MODULE	WONG_PROTEASOME_GENE_MODULE		46	-0.2405344	-0.8771372	0.692308	0.85493565	1	4025	tags=22%, list=18%, signal=27%
VALK_AML_CLUSTER_13	VALK_AML_CLUSTER_13		29	-0.2704048	-0.8753595	0.588235	0.8584364	1	5068	tags=34%, list=23%, signal=45%
BIOCARTA_AGR_PATHWAY	BIOCARTA_AGR_PATHWAY		36	-0.2704275	-0.8751187	0.692308	0.8585215	1	5251	tags=33%, list=24%, signal=44%
GARGALOVIC_RESPONSE_TO_OXIDIZED_PHOSPHOLIPIDS	GARGALOVIC_RESPONSE_TO_OXIDIZED_PHOSPHOLIPIDS_MAGI		25	-0.288724	-0.8746074	0.615385	0.85918486	1	2811	tags=16%, list=13%, signal=18%
REACTOME_MEMBRANE_TRAFFICKING	REACTOME_MEMBRANE_TRAFFICKING		77	-0.2206769	-0.873578	0.644068	0.86104226	1	6161	tags=29%, list=28%, signal=40%
KEGG_STARCH_AND_SUCROSE_METABOLISM	KEGG_STARCH_AND_SUCROSE_METABOLISM		35	-0.269331	-0.8721948	0.6	0.86367804	1	4303	tags=26%, list=20%, signal=32%
REACTOME_RNA_POLYMERASE_I_III_AND_MITOCHONDRIA	REACTOME_RNA_POLYMERASE_I_III_AND_MITOCHONDRIAL_TRA		94	-0.2230962	-0.8720416	0.807018	0.8635624	1	5584	tags=31%, list=25%, signal=41%
SEITZ_NEOPLASTIC_TRANSFORMATION_BY_8P_DELETION	SEITZ_NEOPLASTIC_TRANSFORMATION_BY_8P_DELETION_UP		68	-0.2284831	-0.8714693	0.754386	0.8645295	1	4215	tags=25%, list=19%, signal=31%
GRAHAM_CML_QUIESCENT_VS_CML_DIVIDING_UP	GRAHAM_CML_QUIESCENT_VS_CML_DIVIDING_UP		21	-0.3109371	-0.8712714	0.625	0.8644122	1	2427	tags=19%, list=11%, signal=21%
ROSS_AML_WITH_MLL_FUSIONS	ROSS_AML_WITH_MLL_FUSIONS		72	-0.2369685	-0.8712112	0.660377	0.8640628	1	5161	tags=28%, list=24%, signal=36%
MARIADASON_RESPONSE_TO_CURCUMIN_SULINDAC_5	MARIADASON_RESPONSE_TO_CURCUMIN_SULINDAC_5		23	-0.2911633	-0.8710804	0.68	0.8637746	1	4215	tags=22%, list=19%, signal=27%
RICKMAN_TUMOR_DIFFERENTIATED_WELL_VS_MODERATE	RICKMAN_TUMOR_DIFFERENTIATED_WELL_VS_MODERATELY_UF		103	-0.2160738	-0.8705631	0.737705	0.8644301	1	6621	tags=32%, list=30%, signal=46%
CAIRO_HEPATOBLASTOMA_UP	CAIRO_HEPATOBLASTOMA_UP		202	-0.1924222	-0.8697454	0.777778	0.86571985	1	6385	tags=29%, list=29%, signal=41%
TIEN_INTESTINE_PROBIOTICS_24HR_DN	TIEN_INTESTINE_PROBIOTICS_24HR_DN		204	-0.2035274	-0.8693897	0.716418	0.86603177	1	6790	tags=30%, list=31%, signal=44%
HASLINGER_B_CLL_WITH_17P13_DELETION	HASLINGER_B_CLL_WITH_17P13_DELETION		15	-0.3312419	-0.8685808	0.612245	0.86707103	1	4974	tags=40%, list=23%, signal=52%
RAMALHO_STEMNESS_UP	RAMALHO_STEMNESS_UP		197	-0.1938736	-0.8683765	0.779661	0.86699516	1	5518	tags=24%, list=25%, signal=32%
BROWNE_HCMV_INFECTION_12HR_DN	BROWNE_HCMV_INFECTION_12HR_DN		97	-0.2125035	-0.8678693	0.764706	0.86760396	1	4261	tags=20%, list=19%, signal=24%
VECCHI_GASTRIC_CANCER_ADVANCED_VS_EARLY_UP	VECCHI_GASTRIC_CANCER_ADVANCED_VS_EARLY_UP		156	-0.2014508	-0.8677297	0.838235	0.86738205	1	3561	tags=20%, list=16%, signal=24%
WOTTON_RUNX_TARGETS_UP	WOTTON_RUNX_TARGETS_UP		19	-0.3012358	-0.8671376	0.62069	0.86807984	1	4379	tags=26%, list=20%, signal=33%
BIOCARTA_WNT_PATHWAY	BIOCARTA_WNT_PATHWAY		25	-0.2948838	-0.8660728	0.666667	0.8700255	1	5218	tags=24%, list=24%, signal=31%
TONKS_TARGETS_OF_RUNX1_RUNX1T1_FUSION_MONOCY	TONKS_TARGETS_OF_RUNX1_RUNX1T1_FUSION_MONOCYTE_DN		47	-0.2458065	-0.8657834	0.705882	0.87018895	1	2411	tags=15%, list=11%, signal=17%
LUI_THYROID_CANCER_PAX8_PPARG_DN	LUI_THYROID_CANCER_PAX8_PPARG_DN		43	-0.2417386	-0.8649797	0.711864	0.87156236	1	7603	tags=40%, list=35%, signal=60%
BIOCARTA_IGF1_PATHWAY	BIOCARTA_IGF1_PATHWAY		21	-0.3041812	-0.862885	0.694915	0.8759479	1	7565	tags=52%, list=34%, signal=80%
BIOCARTA_AKT_PATHWAY	BIOCARTA_AKT_PATHWAY		21	-0.2926234	-0.862156	0.595745	0.8770842	1	4171	tags=24%, list=19%, signal=29%
SIG_CD40PATHWAYMAP	SIG_CD40PATHWAYMAP		33	-0.2680069	-0.8609231	0.694915	0.8794175	1	6284	tags=45%, list=29%, signal=64%
RODRIGUES_DCC_TARGETS_DN	RODRIGUES_DCC_TARGETS_DN		117	-0.2160865	-0.8605588	0.796875	0.87962425	1	4626	tags=25%, list=21%, signal=31%
ONDER_CDH1_TARGETS_2_UP	ONDER_CDH1_TARGETS_2_UP		248	-0.192042	-0.8605449	0.833333	0.8791075	1	4143	tags=22%, list=19%, signal=27%
WANG_CLIM2_TARGETS_DN	WANG_CLIM2_TARGETS_DN		162	-0.192823	-0.859134	0.803279	0.8820162	1	3911	tags=19%, list=18%, signal=23%
LIU_COMMON_CANCER_GENES	LIU_COMMON_CANCER_GENES		38	-0.2445251	-0.8587135	0.734694	0.88255936	1	5516	tags=29%, list=25%, signal=39%
BROWNE_HCMV_INFECTION_12HR_UP	BROWNE_HCMV_INFECTION_12HR_UP		100	-0.2210569	-0.8583618	0.781818	0.88299227	1	5832	tags=31%, list=27%, signal=42%
BIOCARTA_AMI_PATHWAY	BIOCARTA_AMI_PATHWAY		19	-0.2874904	-0.8582277	0.673077	0.8828415	1	4075	tags=26%, list=19%, signal=32%
BALDWIN_PRKCI_TARGETS_UP	BALDWIN_PRKCI_TARGETS_UP		32	-0.2766113	-0.8580963	0.660714	0.8826817	1	3155	tags=16%, list=14%, signal=18%
RIZ_ERYTHROID_DIFFERENTIATION	RIZ_ERYTHROID_DIFFERENTIATION		71	-0.2161055	-0.8574672	0.73913	0.8834479	1	4831	tags=24%, list=22%, signal=31%
VALK_AML_CLUSTER_1	VALK_AML_CLUSTER_1		27	-0.2735032	-0.8574192	0.692308	0.88299876	1	2626	tags=19%, list=12%, signal=21%
KEGG_RIBOFLAVIN_METABOLISM	KEGG_RIBOFLAVIN_METABOLISM		16	-0.3245801	-0.8572547	0.650794	0.88284713	1	5970	tags=44%, list=27%, signal=60%
OUELLET_OVARIAN_CANCER_INVASIVE_VS_LMP_UP	OUELLET_OVARIAN_CANCER_INVASIVE_VS_LMP_UP		114	-0.2050331	-0.8568767	0.791045	0.88313586	1	8313	tags=42%, list=38%, signal=67%
ELVIDGE_HIF1A_TARGETS_UP	ELVIDGE_HIF1A_TARGETS_UP		67	-0.2324996	-0.8567044	0.688525	0.8830516	1	6877	tags=36%, list=31%, signal=52%
KEGG_METABOLISM_OF_XENOBIOTICS_BY_CYTOCHROME	KEGG_METABOLISM_OF_XENOBIOTICS_BY_CYTOCHROME_P450		51	-0.234242	-0.8562698	0.71875	0.8835519	1	1683	tags=10%, list=8%, signal=11%
BOGNI_TREATMENT_RELATED_MYELOID_LEUKEMIA_DN	BOGNI_TREATMENT_RELATED_MYELOID_LEUKEMIA_DN		29	-0.2843	-0.8561975	0.740741	0.88321465	1	6989	tags=48%, list=32%, signal=71%
BHATTACHARYA_EMBRYONIC_STEM_CELL	BHATTACHARYA_EMBRYONIC_STEM_CELL		71	-0.2232918	-0.8561409	0.741936	0.88277656	1	5164	tags=24%, list=24%, signal=31%

KIM_LRRC3B_TARGETS	KIM_LRRC3B_TARGETS		26	-0.2923695	-0.8560472	0.666667	0.88248116	1	4715	tags=35%, list=22%, signal=44%
KRASNOSELSKAYA_ILF3_TARGETS_DN	KRASNOSELSKAYA_ILF3_TARGETS_DN		38	-0.2603527	-0.8554133	0.787234	0.88336843	1	5211	tags=34%, list=24%, signal=45%
SU_PANCREAS	SU_PANCREAS		46	-0.2569679	-0.8551204	0.723077	0.8835727	1	4453	tags=24%, list=20%, signal=30%
BONOME_OVARIAN_CANCER_POOR_SURVIVAL_DN	BONOME_OVARIAN_CANCER_POOR_SURVIVAL_DN		19	-0.2904455	-0.8548936	0.666667	0.8835394	1	4108	tags=26%, list=19%, signal=32%
TARTE_PLASMA_CELL_VS_B_LYMPHOCYTE_DN	TARTE_PLASMA_CELL_VS_B_LYMPHOCYTE_DN		35	-0.2522679	-0.8546916	0.775862	0.883387	1	7154	tags=46%, list=33%, signal=68%
STARK_HYPPOCAMPUS_22Q11_DELETION_DN	STARK_HYPPOCAMPUS_22Q11_DELETION_DN		18	-0.3057131	-0.8545404	0.709091	0.88317764	1	1185	tags=11%, list=5%, signal=12%
YAGI_AML_WITH_T_9_11_TRANSLOCATION	YAGI_AML_WITH_T_9_11_TRANSLOCATION		126	-0.2052587	-0.8539842	0.769231	0.88387614	1	2546	tags=11%, list=12%, signal=12%
ST_FAS_SIGNALING_PATHWAY	ST_FAS_SIGNALING_PATHWAY		56	-0.2364315	-0.8493797	0.730769	0.8936622	1	4171	tags=23%, list=19%, signal=29%
REACTOME_NEF_MEDIATES_DOWN_MODULATION_OF_CEL	REACTOME_NEF_MEDIATES_DOWN_MODULATION_OF_CELL_SUI		21	-0.2909386	-0.8493463	0.75	0.8932103	1	7413	tags=43%, list=34%, signal=65%
BOHN_PRIMARY_IMMUNODEFICIENCY_SYNDROM_DN	BOHN_PRIMARY_IMMUNODEFICIENCY_SYNDROM_DN		24	-0.2795916	-0.8492728	0.65	0.8928342	1	4037	tags=29%, list=18%, signal=36%
OKAWA_NEUROBLASTOMA_1P36_31_DELETION	OKAWA_NEUROBLASTOMA_1P36_31_DELETION		22	-0.2726314	-0.8485667	0.660714	0.89380616	1	5955	tags=41%, list=27%, signal=56%
REACTOME_P130CAS_LINKAGE_TO_MAPK_SIGNALING_FO	REACTOME_P130CAS_LINKAGE_TO_MAPK_SIGNALING_FOR_INT		15	-0.3168119	-0.8485586	0.649123	0.89329755	1	6862	tags=47%, list=31%, signal=68%
REACTOME_POST_TRANSLATIONAL_PROTEIN_MODIFICATI	REACTOME_POST_TRANSLATIONAL_PROTEIN_MODIFICATION		40	-0.2633735	-0.847503	0.766667	0.89504063	1	3996	tags=23%, list=18%, signal=27%
REACTOME_SIGNALING_BY_EGFR	REACTOME_SIGNALING_BY_EGFR		48	-0.2414824	-0.8471142	0.733333	0.89534485	1	4961	tags=29%, list=23%, signal=38%
SU_PLACENTA	SU_PLACENTA		25	-0.2842621	-0.8468491	0.711864	0.89543635	1	1089	tags=12%, list=5%, signal=13%
REACTOME_MRNA_SPLICING	REACTOME_MRNA_SPLICING		104	-0.2187712	-0.845781	0.772727	0.89703035	1	3613	tags=17%, list=16%, signal=21%
KEGG_AMINOACYL_TRNA_BIOSYNTHESIS	KEGG_AMINOACYL_TRNA_BIOSYNTHESIS		41	-0.249003	-0.8454328	0.684211	0.8972831	1	7821	tags=44%, list=36%, signal=68%
BROWNE_HCMV_INFECTION_24HR_DN	BROWNE_HCMV_INFECTION_24HR_DN		142	-0.2006958	-0.8450995	0.818182	0.8975588	1	5401	tags=25%, list=25%, signal=33%
AMIT_DELAYED_EARLY_GENES	AMIT_DELAYED_EARLY_GENES		17	-0.3171588	-0.8449484	0.709091	0.8973507	1	6742	tags=41%, list=31%, signal=59%
REACTOME_TOLL_LIKE_RECEPTOR_9_CASCADE	REACTOME_TOLL_LIKE_RECEPTOR_9_CASCADE		21	-0.2901215	-0.8441558	0.72	0.8984973	1	3386	tags=29%, list=15%, signal=34%
BIOCARTA_GSK3_PATHWAY	BIOCARTA_GSK3_PATHWAY		27	-0.2918257	-0.8439952	0.77551	0.89837193	1	5218	tags=33%, list=24%, signal=44%
REACTOME_ACTIVATED_TLR4_SIGNALLING	REACTOME_ACTIVATED_TLR4_SIGNALLING		22	-0.2769612	-0.8439598	0.688525	0.89790416	1	3386	tags=27%, list=15%, signal=32%
YAO_TEMPORAL_RESPONSE_TO_PROGESTERONE_CLUSTE	YAO_TEMPORAL_RESPONSE_TO_PROGESTERONE_CLUSTER_8		47	-0.2426676	-0.8414769	0.711864	0.9021323	1	7602	tags=45%, list=35%, signal=68%
BOYAULT_LIVER_CANCER_SUBCLASS_G1_DN	BOYAULT_LIVER_CANCER_SUBCLASS_G1_DN		39	-0.2524195	-0.8411372	0.755556	0.9023288	1	4405	tags=26%, list=20%, signal=32%
KEGG_CHRONIC_MYELOID_LEUKEMIA	KEGG_CHRONIC_MYELOID_LEUKEMIA		73	-0.2192521	-0.8408259	0.706897	0.90238214	1	5162	tags=26%, list=24%, signal=34%
REACTOME_DOWNSTREAM_TCR_SIGNALING	REACTOME_DOWNSTREAM_TCR_SIGNALING		36	-0.2592594	-0.8400301	0.716667	0.90360206	1	3182	tags=19%, list=15%, signal=23%
TAKEDA_TARGETS_OF_NUP98_HOXA9_FUSION_6HR_UP	TAKEDA_TARGETS_OF_NUP98_HOXA9_FUSION_6HR_UP		81	-0.2139218	-0.8381071	0.724138	0.907557	1	3868	tags=22%, list=18%, signal=27%
HATADA_METHYLATED_IN_LUNG_CANCER_UP	HATADA_METHYLATED_IN_LUNG_CANCER_UP		345	-0.1818373	-0.8376877	0.953125	0.90797514	1	4222	tags=20%, list=19%, signal=24%
ZHAN_MULTIPLE_MYELOMA_HP_DN	ZHAN_MULTIPLE_MYELOMA_HP_DN		44	-0.2504427	-0.8375894	0.721311	0.90770423	1	3272	tags=20%, list=15%, signal=24%
REACTOME_STEROID_HORMONES	REACTOME_STEROID_HORMONES		21	-0.2875078	-0.8368828	0.729167	0.908568	1	4117	tags=33%, list=19%, signal=41%
KEGG_PROPANOATE_METABOLISM	KEGG_PROPANOATE_METABOLISM		31	-0.2730111	-0.8357207	0.686275	0.9104228	1	7006	tags=39%, list=32%, signal=57%
AMBROSINI_FLAVOPIRIDOL_TREATMENT_TP53	AMBROSINI_FLAVOPIRIDOL_TREATMENT_TP53		104	-0.2023707	-0.8349952	0.830509	0.9114938	1	1809	tags=11%, list=8%, signal=11%
BOYAULT_LIVER_CANCER_SUBCLASS_G12_UP	BOYAULT_LIVER_CANCER_SUBCLASS_G12_UP		38	-0.2535932	-0.8347867	0.754717	0.91131276	1	7168	tags=39%, list=33%, signal=59%
BIOCARTA_NFAT_PATHWAY	BIOCARTA_NFAT_PATHWAY		51	-0.2273707	-0.8343692	0.758065	0.91165257	1	2073	tags=14%, list=9%, signal=15%
COLIN_PILOCYTIC_ASTROCYTOMA_VS_GLIOBLASTOMA_UP	COLIN_PILOCYTIC_ASTROCYTOMA_VS_GLIOBLASTOMA_UP		34	-0.2498188	-0.8341911	0.704918	0.9114802	1	3264	tags=21%, list=15%, signal=24%
SIG_PIP3_SIGNALING_IN_CARDIAC_MYOCTES	SIG_PIP3_SIGNALING_IN_CARDIAC_MYOCTES		63	-0.2333865	-0.8330721	0.721311	0.91299665	1	5837	tags=29%, list=27%, signal=39%
OKUMURA_INFLAMMATORY_RESPONSE_LPS	OKUMURA_INFLAMMATORY_RESPONSE_LPS		177	-0.1956693	-0.8322016	0.848485	0.9141618	1	4301	tags=21%, list=20%, signal=26%
ZHANG_ANTIVIRAL_RESPONSE_TO_RIBAVIRIN_DN	ZHANG_ANTIVIRAL_RESPONSE_TO_RIBAVIRIN_DN		40	-0.2291795	-0.832149	0.791045	0.913739	1	3367	tags=20%, list=15%, signal=24%
MAHADEVAN_RESPONSE_TO_MP470_UP	MAHADEVAN_RESPONSE_TO_MP470_UP		17	-0.2868087	-0.8321125	0.696429	0.91325176	1	4187	tags=24%, list=19%, signal=29%
REACTOME_E2F_TRANSCRIPTIONAL_TARGETS_AT_G1_S	REACTOME_E2F_TRANSCRIPTIONAL_TARGETS_AT_G1_S		20	-0.2914988	-0.8318408	0.722222	0.91340524	1	6421	tags=45%, list=29%, signal=64%
MCCABE_HOXC6_TARGETS_DN	MCCABE_HOXC6_TARGETS_DN		21	-0.2919293	-0.8312655	0.666667	0.91383946	1	4559	tags=29%, list=21%, signal=36%
BRUECKNER_TARGETS_OF_MIRLET7A3_UP	BRUECKNER_TARGETS_OF_MIRLET7A3_UP		108	-0.2083786	-0.8310642	0.859649	0.91372323	1	3652	tags=20%, list=17%, signal=24%
BIOCARTA_EIF4_PATHWAY	BIOCARTA_EIF4_PATHWAY		24	-0.2993087	-0.8300693	0.745763	0.9149732	1	5608	tags=33%, list=26%, signal=45%
BIOCARTA_MTOR_PATHWAY	BIOCARTA_MTOR_PATHWAY		23	-0.2799578	-0.828597	0.527273	0.9172714	1	1062	tags=9%, list=5%, signal=9%
IGARASHI_ATF4_TARGETS_DN	IGARASHI_ATF4_TARGETS_DN		82	-0.2144232	-0.8285172	0.851852	0.91689986	1	4672	tags=20%, list=21%, signal=25%
WATANABE_ULCERATIVE_COLITIS_WITH_CANCER_UP	WATANABE_ULCERATIVE_COLITIS_WITH_CANCER_UP		18	-0.3037974	-0.8260577	0.693878	0.9213435	1	4472	tags=28%, list=20%, signal=35%
SILIGAN_BOUND_BY_EWS_FLT1_FUSION	SILIGAN_BOUND_BY_EWS_FLT1_FUSION		35	-0.2577989	-0.8257101	0.666667	0.9214691	1	6356	tags=40%, list=29%, signal=56%
MARTORIATI_MDM4_TARGETS_NEUROEPITHELIUM_UP	MARTORIATI_MDM4_TARGETS_NEUROEPITHELIUM_UP		94	-0.2096856	-0.8256603	0.793103	0.92096287	1	5424	tags=32%, list=25%, signal=42%
REACTOME_TRANSLATION	REACTOME_TRANSLATION		117	-0.2052363	-0.8255912	0.793103	0.92056435	1	1849	tags=9%, list=8%, signal=9%
SAKAI_CHRONIC_HEPATITIS_VS_LIVER_CANCER_UP	SAKAI_CHRONIC_HEPATITIS_VS_LIVER_CANCER_UP		78	-0.2133305	-0.8254374	0.833333	0.92023903	1	3894	tags=18%, list=18%, signal=22%
FAELT_B_CLL_WITH_VH_REARRANGEMENTS_UP	FAELT_B_CLL_WITH_VH_REARRANGEMENTS_UP		48	-0.2273703	-0.8245907	0.777778	0.9212268	1	7717	tags=42%, list=35%, signal=64%
BIOCARTA_MYOSIN_PATHWAY	BIOCARTA_MYOSIN_PATHWAY		31	-0.2611694	-0.8243753	0.674419	0.9210504	1	3410	tags=23%, list=16%, signal=27%
BROWNE_HCMV_INFECTION_4HR_DN	BROWNE_HCMV_INFECTION_4HR_DN		235	-0.1850696	-0.8242472	0.873016	0.92061144	1	2104	tags=10%, list=10%, signal=11%
MORI_EMU_MYC_LYMPHOMA_BY_ONSET_TIME_UP	MORI_EMU_MYC_LYMPHOMA_BY_ONSET_TIME_UP		96	-0.2067737	-0.8234046	0.885714	0.9217643	1	2869	tags=14%, list=13%, signal=16%
CASORELLI_APL_SECONDARY_VS_DE_NOVO_UP	CASORELLI_APL_SECONDARY_VS_DE_NOVO_UP		37	-0.2590862	-0.8233908	0.733333	0.92125183	1	5741	tags=38%, list=26%, signal=51%
BROWNE_HCMV_INFECTION_24HR_UP	BROWNE_HCMV_INFECTION_24HR_UP		142	-0.2033071	-0.8231394	0.847458	0.9212311	1	5350	tags=26%, list=24%, signal=34%
KEGG_PURINE_METABOLISM	KEGG_PURINE_METABOLISM		155	-0.1954034	-0.8218052	0.852941	0.9234161	1	6036	tags=30%, list=28%, signal=41%
GINESTIER_BREAST_CANCER_20Q13_AMPLIFICATION_UP	GINESTIER_BREAST_CANCER_20Q13_AMPLIFICATION_UP		101	-0.2041322	-0.8208067	0.824561	0.9249696	1	3868	tags=16%, list=18%, signal=19%
GARCIA_TARGETS_OF_FLI1_AND_DAX1_UP	GARCIA_TARGETS_OF_FLI1_AND_DAX1_UP		42	-0.2363274	-0.8196542	0.672727	0.9265777	1	5094	tags=33%, list=23%, signal=43%
REACTOME_PI3K_AKT_SIGNALLING	REACTOME_PI3K_AKT_SIGNALLING		37	-0.2453337	-0.8192725	0.8	0.9267583	1	5608	tags=30%, list=26%, signal=40%
BROWNE_HCMV_INFECTION_2HR_UP	BROWNE_HCMV_INFECTION_2HR_UP		34	-0.2508742	-0.818466	0.732143	0.92769086	1	4205	tags=24%, list=19%, signal=29%
BIOCARTA_TNFR2_PATHWAY	BIOCARTA_TNFR2_PATHWAY		18	-0.3017655	-0.8182746	0.655172	0.92747855	1	6434	tags=28%, list=29%, signal=39%
MENSE_HYPOXIA_UP	MENSE_HYPOXIA_UP		92	-0.2121063	-0.8172024	0.785714	0.9289435	1	5364	tags=27%, list=24%, signal=36%
KEGG_CYTOSOLIC_DNA_SENSING_PATHWAY	KEGG_CYTOSOLIC_DNA_SENSING_PATHWAY		49	-0.2344301	-0.8171772	0.72549	0.9284542	1	2784	tags=16%, list=13%, signal=19%

REACTOME_NCAM_SIGNALING_FOR_NEURITE_OUT_GROW	REACTOME_NCAM_SIGNALING_FOR_NEURITE_OUT_GROWTH		68	-0.2110205	-0.8169576	0.796875	0.92840683	1	5029	tags=28%, list=23%, signal=36%
GRABARCZYK_BCL11B_TARGETS_UP	GRABARCZYK_BCL11B_TARGETS_UP		69	-0.2225542	-0.816914	0.777778	0.92796695	1	1764	tags=12%, list=8%, signal=13%
GUTIERREZ_MULTIPLE_MYELOMA_DN	GUTIERREZ_MULTIPLE_MYELOMA_DN		34	-0.2476001	-0.8164929	0.678571	0.92836314	1	6156	tags=32%, list=28%, signal=45%
IVANOVA_HEMATOPOIESIS_EARLY_PROGENITOR	IVANOVA_HEMATOPOIESIS_EARLY_PROGENITOR		104	-0.209771	-0.8163844	0.868852	0.9280126	1	4195	tags=19%, list=19%, signal=24%
WATANABE_COLON_CANCER_MSI_VS_MSS_UP	WATANABE_COLON_CANCER_MSI_VS_MSS_UP		27	-0.2547629	-0.8163015	0.851852	0.92762274	1	3874	tags=30%, list=18%, signal=36%
KUROKAWA_LIVER_CANCER_CHEMOTHERAPY_DN	KUROKAWA_LIVER_CANCER_CHEMOTHERAPY_DN		17	-0.288387	-0.8162699	0.660377	0.92714316	1	6193	tags=41%, list=28%, signal=57%
REACTOME_PHOSPHORYLATION_OF_THE_APC	REACTOME_PHOSPHORYLATION_OF_THE_APC		16	-0.2973107	-0.8160209	0.673077	0.92709327	1	9404	tags=63%, list=43%, signal=109%
SENESE_HDAC3_TARGETS_UP	SENESE_HDAC3_TARGETS_UP		459	-0.1695777	-0.8153036	0.942029	0.9278888	1	4556	tags=19%, list=21%, signal=24%
KEGG_MISMATCH_REPAIR	KEGG_MISMATCH_REPAIR		22	-0.2646439	-0.8149771	0.686275	0.927912	1	1799	tags=14%, list=8%, signal=15%
KEGG_PROTEASOME	KEGG_PROTEASOME		44	-0.2379303	-0.8138681	0.75	0.92939377	1	6834	tags=36%, list=31%, signal=53%
TONG_INTERACT_WITH_PTTG1	TONG_INTERACT_WITH_PTTG1		53	-0.231721	-0.8137994	0.807018	0.9289717	1	5548	tags=30%, list=25%, signal=40%
NGUYEN_NOTCH1_TARGETS_DN	NGUYEN_NOTCH1_TARGETS_DN		64	-0.2230842	-0.8129653	0.8	0.929853	1	7064	tags=36%, list=32%, signal=53%
KAAB_FAILED_HEART_ATRIUM_DN	KAAB_FAILED_HEART_ATRIUM_DN		139	-0.1931612	-0.811655	0.938462	0.93162996	1	6917	tags=32%, list=32%, signal=46%
KEGG_PENTOSE_AND_GLUCURONATE_INTERCONVERSIONS	KEGG_PENTOSE_AND_GLUCURONATE_INTERCONVERSIONS		16	-0.2757146	-0.8106474	0.630435	0.9330007	1	7757	tags=50%, list=35%, signal=77%
MULLIGHAN_NPM1_SIGNATURE_3_UP	MULLIGHAN_NPM1_SIGNATURE_3_UP		319	-0.1724286	-0.8098544	0.932203	0.9337714	1	5809	tags=26%, list=26%, signal=35%
BIOCARTA_STATHMIN_PATHWAY	BIOCARTA_STATHMIN_PATHWAY		17	-0.2830781	-0.8097042	0.68	0.93344426	1	980	tags=12%, list=4%, signal=12%
ZHAN_MULTIPLE_MYELOMA_CD1_DN	ZHAN_MULTIPLE_MYELOMA_CD1_DN		39	-0.2505432	-0.808586	0.666667	0.93489826	1	3384	tags=21%, list=15%, signal=24%
LIU_TARGETS_OF_VMYB_VS_CMYB_DN	LIU_TARGETS_OF_VMYB_VS_CMYB_DN		42	-0.2447123	-0.8082731	0.75	0.9348203	1	3710	tags=21%, list=17%, signal=26%
HADDAD_T_LYMPHOCYTE_AND_NK_PROGENITOR_UP	HADDAD_T_LYMPHOCYTE_AND_NK_PROGENITOR_UP		76	-0.2136562	-0.8081188	0.795455	0.93463916	1	4143	tags=18%, list=19%, signal=23%
PUIFFE_INVASION_INHIBITED_BY_ASCITES_UP	PUIFFE_INVASION_INHIBITED_BY_ASCITES_UP		75	-0.2080954	-0.8069837	0.84127	0.9361563	1	5302	tags=25%, list=24%, signal=33%
REACTOME_METABOLISM_OF_PROTEINS	REACTOME_METABOLISM_OF_PROTEINS		209	-0.1762815	-0.8063341	0.984375	0.9368583	1	3996	tags=14%, list=18%, signal=17%
REACTOME_RNA_POLYMERASE_I_CHAIN_ELONGATION	REACTOME_RNA_POLYMERASE_I_CHAIN_ELONGATION		25	-0.2447687	-0.80597	0.730769	0.9369886	1	1385	tags=12%, list=6%, signal=13%
BASSO_B_LYMPHOCYTE_NETWORK	BASSO_B_LYMPHOCYTE_NETWORK		134	-0.1873179	-0.8049929	0.898551	0.93812615	1	4317	tags=19%, list=20%, signal=23%
WALLACE_PROSTATE_CANCER_RACE_DN	WALLACE_PROSTATE_CANCER_RACE_DN		72	-0.2076814	-0.8043616	0.781818	0.93871075	1	1472	tags=10%, list=7%, signal=10%
BOYAULT_LIVER_CANCER_SUBCLASS_G3_UP	BOYAULT_LIVER_CANCER_SUBCLASS_G3_UP		181	-0.185536	-0.804008	0.916667	0.93876934	1	6119	tags=25%, list=28%, signal=34%
KEGG_ETHER_LIPID_METABOLISM	KEGG_ETHER_LIPID_METABOLISM		32	-0.2500686	-0.8026141	0.792453	0.9406936	1	3976	tags=22%, list=18%, signal=27%
FLECHNER_PBL_KIDNEY_TRANSPLANT_OK_VS_DONOR_UP	FLECHNER_PBL_KIDNEY_TRANSPLANT_OK_VS_DONOR_UP		145	-0.1876034	-0.8022655	0.851852	0.9408308	1	6106	tags=29%, list=28%, signal=40%
LIU_VMYB_TARGETS_UP	LIU_VMYB_TARGETS_UP		122	-0.1884423	-0.8021563	0.932203	0.94050986	1	6702	tags=34%, list=31%, signal=48%
GALLUZZI_PERMEABILIZE_MITOCHONDRIA	GALLUZZI_PERMEABILIZE_MITOCHONDRIA		38	-0.2552432	-0.8021495	0.75	0.93997365	1	7575	tags=42%, list=35%, signal=64%
CHOI_ATL_CHRONIC_VS_ACUTE_DN	CHOI_ATL_CHRONIC_VS_ACUTE_DN		15	-0.3122307	-0.8014532	0.759259	0.9407021	1	6009	tags=27%, list=27%, signal=37%
BIOCARTA_RAS_PATHWAY	BIOCARTA_RAS_PATHWAY		23	-0.2818841	-0.8009082	0.745455	0.941043	1	2073	tags=13%, list=9%, signal=14%
GUENTHER_GROWTH_SPHERICAL_VS_ADHERENT_DN	GUENTHER_GROWTH_SPHERICAL_VS_ADHERENT_DN		25	-0.2665934	-0.8002099	0.706897	0.9416107	1	4938	tags=28%, list=23%, signal=36%
REACTOME_VIF_MEDIATED_DEGRADATION_OF_APOBEC3G	REACTOME_VIF_MEDIATED_DEGRADATION_OF_APOBEC3G		46	-0.2391181	-0.7990056	0.827586	0.9428646	1	6834	tags=33%, list=31%, signal=47%
REACTOME_GENERATION_OF_SECOND_MESSENGER_MOLE	REACTOME_GENERATION_OF_SECOND_MESSENGER_MOLECULE		27	-0.2480529	-0.7989782	0.758621	0.9424086	1	3425	tags=22%, list=16%, signal=26%
ALONSO_METASTASIS_UP	ALONSO_METASTASIS_UP		152	-0.1822903	-0.7988065	0.901961	0.9421671	1	7006	tags=36%, list=32%, signal=52%
CHIANG_LIVER_CANCER_SUBCLASS_UNANNOTATED_DN	CHIANG_LIVER_CANCER_SUBCLASS_UNANNOTATED_DN		150	-0.1829526	-0.7967119	0.982143	0.9449907	1	6834	tags=32%, list=31%, signal=46%
RIGGINS_TAMOXIFEN_RESISTANCE_DN	RIGGINS_TAMOXIFEN_RESISTANCE_DN		209	-0.1774152	-0.7948613	0.985507	0.9473572	1	1494	tags=7%, list=7%, signal=8%
SABATES_COLORECTAL_ADENOMA_SIZE_UP	SABATES_COLORECTAL_ADENOMA_SIZE_UP		18	-0.2872609	-0.7946876	0.787234	0.94713163	1	7823	tags=50%, list=36%, signal=78%
KEGG_ANTIGEN_PROCESSING_AND_PRESENTATION	KEGG_ANTIGEN_PROCESSING_AND_PRESENTATION		61	-0.231906	-0.7944332	0.85	0.9470091	1	4803	tags=28%, list=22%, signal=36%
KEGG_VASOPRESSIN_REGULATED_WATER_REABSORPTION	KEGG_VASOPRESSIN_REGULATED_WATER_REABSORPTION		43	-0.2348324	-0.7941151	0.827586	0.94694227	1	6310	tags=37%, list=29%, signal=52%
LIANG_HEMATOPOIESIS_STEM_CELL_NUMBER_SMALL_VS	LIANG_HEMATOPOIESIS_STEM_CELL_NUMBER_SMALL_VS_HUGE		37	-0.2496869	-0.7940275	0.75	0.94655734	1	5489	tags=30%, list=25%, signal=40%
LIANG_HEMATOPOIESIS_STEM_CELL_NUMBER_LARGE_VS	LIANG_HEMATOPOIESIS_STEM_CELL_NUMBER_LARGE_VS_TINY		41	-0.2392015	-0.7930578	0.822581	0.94760936	1	4616	tags=24%, list=21%, signal=31%
KENNY_CTNNB1_TARGETS_DN	KENNY_CTNNB1_TARGETS_DN		41	-0.2308109	-0.792715	0.813559	0.9476754	1	5183	tags=27%, list=24%, signal=35%
KEGG_EPITHELIAL_CELL_SIGNALING_IN_HELICOBACTER_I	KEGG_EPITHELIAL_CELL_SIGNALING_IN_HELICOBACTER_PYLOR		65	-0.2114591	-0.7924512	0.825397	0.9476002	1	4171	tags=20%, list=19%, signal=25%
KEGG_PHENYLALANINE_METABOLISM	KEGG_PHENYLALANINE_METABOLISM		18	-0.2853712	-0.7915289	0.706897	0.9483441	1	5022	tags=28%, list=23%, signal=36%
HANN_RESISTANCE_TO_BCL2_INHIBITOR_DN	HANN_RESISTANCE_TO_BCL2_INHIBITOR_DN		43	-0.2495933	-0.7901373	0.745763	0.94979864	1	4270	tags=28%, list=19%, signal=35%
ABE_INNER_EAR	ABE_INNER_EAR		22	-0.2756822	-0.7885792	0.767857	0.95160264	1	3302	tags=18%, list=15%, signal=21%
SUH_COEXPRESSED_WITH_ID1_AND_ID2_UP	SUH_COEXPRESSED_WITH_ID1_AND_ID2_UP		17	-0.270285	-0.7882605	0.77551	0.95158935	1	6659	tags=53%, list=30%, signal=76%
HSIAO_HOUSEKEEPING_GENES	HSIAO_HOUSEKEEPING_GENES		377	-0.1663514	-0.7881166	1	0.9512839	1	6317	tags=26%, list=29%, signal=36%
RICKMAN_HEAD_AND_NECK_CANCER_A	RICKMAN_HEAD_AND_NECK_CANCER_A		90	-0.2009935	-0.7880164	0.870968	0.95091295	1	343	tags=6%, list=2%, signal=6%
ZHANG_TARGETS_OF_EWSR1_FLI1_FUSION	ZHANG_TARGETS_OF_EWSR1_FLI1_FUSION		81	-0.2020121	-0.7878149	0.807018	0.95071745	1	6349	tags=33%, list=29%, signal=47%
BROWNE_HCMV_INFECTION_10HR_UP	BROWNE_HCMV_INFECTION_10HR_UP		90	-0.1965548	-0.7856816	0.932432	0.9535226	1	4248	tags=20%, list=19%, signal=25%
KORKOLA_TERATOMA_UP	KORKOLA_TERATOMA_UP		15	-0.2957552	-0.7856044	0.6875	0.95311266	1	5413	tags=33%, list=25%, signal=44%
KEGG_LYSOSOME	KEGG_LYSOSOME		119	-0.1912434	-0.7854718	0.909091	0.95278955	1	6161	tags=27%, list=28%, signal=37%
HEDENFALK_BREAST_CANCER_BRACX_UP	HEDENFALK_BREAST_CANCER_BRACX_UP		17	-0.2750542	-0.7848352	0.696429	0.95338607	1	1474	tags=12%, list=7%, signal=13%
POMEROY_MEDULLOBLASTOMA_PROGNOSIS_DN	POMEROY_MEDULLOBLASTOMA_PROGNOSIS_DN		39	-0.2371112	-0.7838242	0.765625	0.95433104	1	2825	tags=13%, list=13%, signal=15%
WEBER_METHYLATED_LCP_IN_SPERM_UP	WEBER_METHYLATED_LCP_IN_SPERM_UP		15	-0.3042732	-0.7831159	0.803279	0.95484924	1	3694	tags=27%, list=17%, signal=32%
SUZUKI_AMPLIFIED_IN_ORAL_CANCER	SUZUKI_AMPLIFIED_IN_ORAL_CANCER		16	-0.3026075	-0.7819848	0.783333	0.9559592	1	5025	tags=31%, list=23%, signal=41%
BIOCARTA_ERK_PATHWAY	BIOCARTA_ERK_PATHWAY		28	-0.2427814	-0.7805024	0.875	0.95764124	1	4961	tags=29%, list=23%, signal=37%
GARGALOVIC_RESPONSE_TO_OXIDIZED_PHOSPHOLIPIDS	GARGALOVIC_RESPONSE_TO_OXIDIZED_PHOSPHOLIPIDS_BLUE		119	-0.1885327	-0.7804924	0.854839	0.9571124	1	6073	tags=30%, list=28%, signal=42%
BERNARD_PPAPDC1B_TARGETS_UP	BERNARD_PPAPDC1B_TARGETS_UP		34	-0.2390873	-0.7796943	0.827586	0.957865	1	5424	tags=24%, list=25%, signal=31%
ST_GA12_PATHWAY	ST_GA12_PATHWAY		22	-0.2740861	-0.7781247	0.72093	0.9596129	1	5725	tags=32%, list=26%, signal=43%
MURAKAMI_UV_RESPONSE_24HR	MURAKAMI_UV_RESPONSE_24HR		17	-0.2726384	-0.7778959	0.843137	0.9593517	1	4718	tags=35%, list=22%, signal=45%
REACTOME_HORMONE_BIOSYNTHESIS	REACTOME_HORMONE_BIOSYNTHESIS		50	-0.2362012	-0.7762516	0.875	0.96108973	1	4117	tags=24%, list=19%, signal=29%

CHO_NR4A1_TARGETS	CHO_NR4A1_TARGETS		22	-0.2624453	-0.7747107	0.830509	0.9628204	1	6596	tags=41%, list=30%, signal=58%
BIOCARTA_CHEMICAL_PATHWAY	BIOCARTA_CHEMICAL_PATHWAY		22	-0.268662	-0.7738731	0.763636	0.9634262	1	3021	tags=18%, list=14%, signal=21%
REACTOME_MRNA_3_END_PROCESSING	REACTOME_MRNA_3_END_PROCESSING		34	-0.2418858	-0.770244	0.803571	0.96808267	1	3296	tags=18%, list=15%, signal=21%
TSUNODA_CISPLATIN_RESISTANCE_DN	TSUNODA_CISPLATIN_RESISTANCE_DN		26	-0.2591547	-0.7701459	0.847458	0.96764433	1	5374	tags=38%, list=25%, signal=51%
ZHU_CMV_ALL_UP	ZHU_CMV_ALL_UP		57	-0.2042528	-0.770117	0.87234	0.96712834	1	6288	tags=33%, list=29%, signal=47%
KOBAYASHI_EGFR_SIGNALING_24HR_UP	KOBAYASHI_EGFR_SIGNALING_24HR_UP		85	-0.2016449	-0.7688417	0.875	0.9683556	1	4876	tags=26%, list=22%, signal=33%
GRUETZMANN_PANCREATIC_CANCER_DN	GRUETZMANN_PANCREATIC_CANCER_DN		191	-0.1760018	-0.7680783	0.970149	0.9689008	1	4017	tags=16%, list=18%, signal=19%
REACTOME_SIGNALING_BY_NOTCH	REACTOME_SIGNALING_BY_NOTCH		16	-0.279795	-0.7674263	0.767857	0.9691633	1	3494	tags=25%, list=16%, signal=30%
FARMER_BREAST_CANCER_CLUSTER_3	FARMER_BREAST_CANCER_CLUSTER_3		15	-0.2817868	-0.7665151	0.784314	0.969709	1	2740	tags=20%, list=12%, signal=23%
BECKER_TAMOXIFEN_RESISTANCE_DN	BECKER_TAMOXIFEN_RESISTANCE_DN		45	-0.2246423	-0.7663046	0.84375	0.9694423	1	4899	tags=27%, list=22%, signal=34%
SEIDEN_MET_SIGNALING	SEIDEN_MET_SIGNALING		18	-0.2589815	-0.7657418	0.781818	0.9696135	1	9103	tags=56%, list=42%, signal=95%
ROYLANCE_BREAST_CANCER_16Q_COPY_NUMBER_UP	ROYLANCE_BREAST_CANCER_16Q_COPY_NUMBER_UP		32	-0.2279395	-0.7654257	0.842105	0.9695036	1	5107	tags=31%, list=23%, signal=41%
REACTOME_GRB2_SOS_PROVIDES_LINKAGE_TO_MAPK_SI	REACTOME_GRB2_SOS_PROVIDES_LINKAGE_TO_MAPK_SIGNAL		15	-0.2753952	-0.7653787	0.767857	0.96901226	1	6862	tags=47%, list=31%, signal=68%
LEE_METASTASIS_AND_ALTERNATIVE_SPLICING_DN	LEE_METASTASIS_AND_ALTERNATIVE_SPLICING_DN		43	-0.2235265	-0.7647567	0.716667	0.96923095	1	4962	tags=28%, list=23%, signal=36%
REACTOME_GENE_EXPRESSION	REACTOME_GENE_EXPRESSION		419	-0.1575175	-0.7627149	0.984615	0.97137874	1	3307	tags=12%, list=15%, signal=14%
GOLUB_ALL_VS_AML_UP	GOLUB_ALL_VS_AML_UP		19	-0.2735997	-0.7625378	0.827586	0.97114336	1	1653	tags=16%, list=8%, signal=17%
KORKOLA_YOLK_SAC_TUMOR	KORKOLA_YOLK_SAC_TUMOR		45	-0.2135268	-0.7622846	0.886793	0.97093725	1	3891	tags=22%, list=18%, signal=27%
REACTOME_TOLL_LIKE_RECEPTOR_4_CASCADE	REACTOME_TOLL_LIKE_RECEPTOR_4_CASCADE		26	-0.250882	-0.7620865	0.890909	0.97063285	1	3743	tags=27%, list=17%, signal=32%
ZHAN_MULTIPLE_MYELOMA_SPIKED	ZHAN_MULTIPLE_MYELOMA_SPIKED		18	-0.2655551	-0.7620027	0.877551	0.97025955	1	1876	tags=17%, list=9%, signal=18%
SESTO_RESPONSE_TO_UV_C2	SESTO_RESPONSE_TO_UV_C2		53	-0.2146532	-0.7619382	0.861538	0.9697928	1	3948	tags=17%, list=18%, signal=21%
GENTILE_UV_RESPONSE_CLUSTER_D2	GENTILE_UV_RESPONSE_CLUSTER_D2		34	-0.2352583	-0.7613983	0.821429	0.97000855	1	3946	tags=21%, list=18%, signal=25%
BONCI_TARGETS_OF_MIR15A_AND_MIR16_1	BONCI_TARGETS_OF_MIR15A_AND_MIR16_1		79	-0.1903112	-0.7600294	0.896552	0.97123444	1	3848	tags=18%, list=18%, signal=21%
JAATINEN_HEMATOPOIETIC_STEM_CELL_UP	JAATINEN_HEMATOPOIETIC_STEM_CELL_UP		295	-0.1612332	-0.758179	1	0.9729552	1	4767	tags=18%, list=22%, signal=23%
LUI_THYROID_CANCER_PAX8_PPARG_UP	LUI_THYROID_CANCER_PAX8_PPARG_UP		42	-0.2303069	-0.75499	0.716981	0.97618246	1	5258	tags=29%, list=24%, signal=38%
ST_INTEGRIN_SIGNALING_PATHWAY	ST_INTEGRIN_SIGNALING_PATHWAY		78	-0.1983306	-0.7546628	0.90625	0.97603106	1	5837	tags=31%, list=27%, signal=42%
GENTILE_UV_RESPONSE_CLUSTER_D6	GENTILE_UV_RESPONSE_CLUSTER_D6		30	-0.2470905	-0.7546033	0.886793	0.9755709	1	5822	tags=33%, list=27%, signal=45%
KIM_MYCN_AMPLIFICATION_TARGETS_DN	KIM_MYCN_AMPLIFICATION_TARGETS_DN		76	-0.2022346	-0.7543794	0.827586	0.9753349	1	4071	tags=18%, list=19%, signal=23%
IIZUKA_LIVER_CANCER_PROGRESSION_G1_G2_DN	IIZUKA_LIVER_CANCER_PROGRESSION_G1_G2_DN		22	-0.253559	-0.7540523	0.86	0.9751473	1	6446	tags=32%, list=29%, signal=45%
BIOCARTA_GLEEVEC_PATHWAY	BIOCARTA_GLEEVEC_PATHWAY		23	-0.241922	-0.7537822	0.792453	0.9748957	1	2073	tags=13%, list=9%, signal=14%
REACTOME_G_ALPHA_12_13_SIGNALLING_EVENTS	REACTOME_G_ALPHA_12_13_SIGNALLING_EVENTS		54	-0.2182191	-0.7534472	0.836735	0.97469836	1	6663	tags=37%, list=30%, signal=53%
AKL_HTLV1_INFECTION_DN	AKL_HTLV1_INFECTION_DN		66	-0.1998172	-0.7521976	0.913793	0.9755823	1	4218	tags=20%, list=19%, signal=24%
TAKAO_RESPONSE_TO_UVB_RADIATION_DN	TAKAO_RESPONSE_TO_UVB_RADIATION_DN		89	-0.196558	-0.7511671	0.925373	0.97620225	1	6310	tags=27%, list=29%, signal=38%
REACTOME_NA_CL_DEPENDENT_NEUROTRANSMITTER_TRA	REACTOME_NA_CL_DEPENDENT_NEUROTRANSMITTER_TRANSPO		18	-0.259252	-0.7492639	0.857143	0.978141	1	2996	tags=17%, list=14%, signal=19%
REACTOME_G_PROTEIN_BETA_GAMMA_SIGNALLING	REACTOME_G_PROTEIN_BETA_GAMMA_SIGNALLING		28	-0.2441061	-0.74869	0.803279	0.9783823	1	4665	tags=29%, list=21%, signal=36%
SENESE_HDAC2_TARGETS_UP	SENESE_HDAC2_TARGETS_UP		106	-0.182867	-0.74707	0.92	0.97966856	1	5834	tags=28%, list=27%, signal=38%
BIOCARTA_IL22BP_PATHWAY	BIOCARTA_IL22BP_PATHWAY		16	-0.287328	-0.746573	0.815385	0.9797394	1	3255	tags=25%, list=15%, signal=29%
PUIFFE_INVASION_INHIBITED_BY_ASCITES_DN	PUIFFE_INVASION_INHIBITED_BY_ASCITES_DN		141	-0.1789708	-0.7448273	0.96	0.98131627	1	2529	tags=11%, list=12%, signal=13%
KEGG_PEROXISOME	KEGG_PEROXISOME		77	-0.1899288	-0.7445654	0.882353	0.98109347	1	5109	tags=22%, list=23%, signal=29%
SIG_IL4RECEPTOR_IN_B_LYPHOCYTES	SIG_IL4RECEPTOR_IN_B_LYPHOCYTES		27	-0.2481162	-0.7434136	0.809524	0.98200667	1	5837	tags=41%, list=27%, signal=55%
DAZARD_UV_RESPONSE_CLUSTER_G6	DAZARD_UV_RESPONSE_CLUSTER_G6		123	-0.1729045	-0.7433137	0.935484	0.9815549	1	4046	tags=16%, list=18%, signal=20%
KORKOLA_EMBRYONIC_CARCINOMA_VS_SEMINOMA_UP	KORKOLA_EMBRYONIC_CARCINOMA_VS_SEMINOMA_UP		21	-0.2566887	-0.7406334	0.784314	0.98397905	1	406	tags=10%, list=2%, signal=10%
SMITH_LIVER_CANCER	SMITH_LIVER_CANCER		30	-0.2343235	-0.7392288	0.857143	0.98510873	1	9278	tags=53%, list=42%, signal=92%
YEGNASUBRAMANIAN_PROSTATE_CANCER	YEGNASUBRAMANIAN_PROSTATE_CANCER		114	-0.182434	-0.7389135	0.968254	0.984954	1	2260	tags=11%, list=10%, signal=13%
SEKI_INFLAMMATORY_RESPONSE_LPS_DN	SEKI_INFLAMMATORY_RESPONSE_LPS_DN		24	-0.2495345	-0.7387906	0.867925	0.9845702	1	5110	tags=33%, list=23%, signal=43%
HOFMANN_MYELODYSPLASTIC_SYNDROM_LOW_RISK_UP	HOFMANN_MYELODYSPLASTIC_SYNDROM_LOW_RISK_UP		19	-0.2605816	-0.7386796	0.77193	0.98416334	1	5248	tags=32%, list=24%, signal=41%
HOEBEKE_LYMPHOID_STEM_CELL_UP	HOEBEKE_LYMPHOID_STEM_CELL_UP		86	-0.1896533	-0.7384512	0.919355	0.9839024	1	6261	tags=28%, list=29%, signal=39%
BIOCARTA_RARRXR_PATHWAY	BIOCARTA_RARRXR_PATHWAY		15	-0.2807707	-0.7383217	0.82	0.9835335	1	3830	tags=20%, list=17%, signal=24%
SEIDEN_ONCOGENESIS_BY_MET	SEIDEN_ONCOGENESIS_BY_MET		85	-0.1843122	-0.736242	0.949153	0.985314	1	2123	tags=9%, list=10%, signal=10%
XU_GH1_AUTOCRINE_TARGETS_UP	XU_GH1_AUTOCRINE_TARGETS_UP		159	-0.1699432	-0.7359889	0.970149	0.9851838	1	4729	tags=21%, list=22%, signal=26%
XU_HGF_TARGETS_INDUCED_BY_AKT1_48HR_DN	XU_HGF_TARGETS_INDUCED_BY_AKT1_48HR_DN		17	-0.2562335	-0.7350355	0.75	0.9857087	1	3848	tags=24%, list=18%, signal=29%
MOREAUX_B_LYMPHOCYTE_MATURATION_BY_TACI_DN	MOREAUX_B_LYMPHOCYTE_MATURATION_BY_TACI_DN		44	-0.2194805	-0.7349828	0.872727	0.98522633	1	7573	tags=45%, list=35%, signal=69%
ROME_INSULIN_TARGETS_IN_MUSCLE_DN	ROME_INSULIN_TARGETS_IN_MUSCLE_DN		45	-0.2320818	-0.7344333	0.894737	0.98533225	1	2522	tags=13%, list=12%, signal=15%
FERRARI_RESPONSE_TO_FENRETINIDE_UP	FERRARI_RESPONSE_TO_FENRETINIDE_UP		17	-0.2728234	-0.7328126	0.779661	0.9865317	1	4329	tags=35%, list=20%, signal=44%
REACTOME_DEADENYLATION_OF_MRNA	REACTOME_DEADENYLATION_OF_MRNA		22	-0.2450608	-0.7321104	0.844444	0.9866916	1	4275	tags=23%, list=19%, signal=28%
RASHI_RESPONSE_TO_IONIZING_RADIATION_4	RASHI_RESPONSE_TO_IONIZING_RADIATION_4		52	-0.203881	-0.7317486	0.934426	0.98652846	1	8336	tags=52%, list=38%, signal=84%
ZHU_CMV_8_HR_DN	ZHU_CMV_8_HR_DN		30	-0.2392854	-0.7317309	0.814815	0.986024	1	4073	tags=23%, list=19%, signal=29%
BIOCARTA_TGFB_PATHWAY	BIOCARTA_TGFB_PATHWAY		18	-0.2573818	-0.7293832	0.867925	0.9880201	1	4961	tags=33%, list=23%, signal=43%
BIOCARTA_ECM_PATHWAY	BIOCARTA_ECM_PATHWAY		24	-0.2474304	-0.7291027	0.85	0.9878123	1	3410	tags=25%, list=16%, signal=30%
NIKOLSKY_BREAST_CANCER_16Q24_AMPLICON	NIKOLSKY_BREAST_CANCER_16Q24_AMPLICON		46	-0.1930066	-0.7274469	0.910714	0.9890019	1	3949	tags=17%, list=18%, signal=21%
KEGG_AMYOTROPHIC_LATERAL_SCLEROSIS_ALS	KEGG_AMYOTROPHIC_LATERAL_SCLEROSIS_ALS		53	-0.2126743	-0.7259341	0.934426	0.99007773	1	6826	tags=40%, list=31%, signal=57%
MCGARVEY_SILENCED_BY_METHYLATION_IN_COLON_CAN	MCGARVEY_SILENCED_BY_METHYLATION_IN_COLON_CANCER		41	-0.2161332	-0.7253231	0.90566	0.9902246	1	4318	tags=27%, list=20%, signal=33%
HORIUCHI_WTAP_TARGETS_UP	HORIUCHI_WTAP_TARGETS_UP		273	-0.1607016	-0.724817	1	0.99023527	1	3913	tags=17%, list=18%, signal=21%
WANG_RECURRENT_LIVER_CANCER_UP	WANG_RECURRENT_LIVER_CANCER_UP		16	-0.2636285	-0.7245007	0.833333	0.9901334	1	5301	tags=31%, list=24%, signal=41%
JI_RESPONSE_TO_FSH_DN	JI_RESPONSE_TO_FSH_DN		47	-0.2047983	-0.7239162	0.893939	0.9901198	1	2371	tags=11%, list=11%, signal=12%

SHIPP_DLBCL_CURED_VS_FATAL_DN	SHIPP_DLBCL_CURED_VS_FATAL_DN		42	-0.2173176	-0.7235966	0.903226	0.9898586	1	2446	tags=14%, list=11%, signal=16%
SA_G1_AND_S_PHASES	SA_G1_AND_S_PHASES		15	-0.2765807	-0.7214794	0.83871	0.99121654	1	6018	tags=40%, list=27%, signal=55%
FLECHNER_PBL_KIDNEY_TRANSPLANT_REJECTED_VS_OK	FLECHNER_PBL_KIDNEY_TRANSPLANT_REJECTED_VS_OK_DN		49	-0.1969652	-0.7204398	0.934426	0.9916867	1	7333	tags=37%, list=33%, signal=55%
BOYLAN_MULTIPLE_MYELOMA_C_D_UP	BOYLAN_MULTIPLE_MYELOMA_C_D_UP		119	-0.1777703	-0.7196916	0.967213	0.9918257	1	5596	tags=24%, list=26%, signal=31%
PACHER_TARGETS_OF_IGF1_AND_IGF2_UP	PACHER_TARGETS_OF_IGF1_AND_IGF2_UP		31	-0.2344886	-0.7183209	0.862069	0.9927529	1	3685	tags=23%, list=17%, signal=27%
WIKMAN_ASBESTOS_LUNG_CANCER_DN	WIKMAN_ASBESTOS_LUNG_CANCER_DN		27	-0.2312715	-0.7179976	0.903846	0.9924904	1	7668	tags=44%, list=35%, signal=68%
REACTOME_CD28_CO_STIMULATION	REACTOME_CD28_CO_STIMULATION		29	-0.2398199	-0.7165993	0.814815	0.9931952	1	6884	tags=34%, list=31%, signal=50%
KEGG_NICOTINATE_AND_NICOTINAMIDE_METABOLISM	KEGG_NICOTINATE_AND_NICOTINAMIDE_METABOLISM		23	-0.24698	-0.7152786	0.808511	0.9938551	1	6991	tags=43%, list=32%, signal=64%
MULLIGHAN_NPM1_MUTATED_SIGNATURE_1_UP	MULLIGHAN_NPM1_MUTATED_SIGNATURE_1_UP		253	-0.1582294	-0.7145763	1	0.99406064	1	5809	tags=25%, list=26%, signal=33%
BIOCARTA_SPRY_PATHWAY	BIOCARTA_SPRY_PATHWAY		18	-0.2486454	-0.7141448	0.862069	0.99397165	1	5725	tags=44%, list=26%, signal=60%
LIN_MELANOMA_COPY_NUMBER_UP	LIN_MELANOMA_COPY_NUMBER_UP		63	-0.1979178	-0.7137699	0.965517	0.99381346	1	6768	tags=35%, list=31%, signal=50%
KEGG_GLYCOSAMINOGLYCAN_BIOSYNTHESIS_CHONDROIT	KEGG_GLYCOSAMINOGLYCAN_BIOSYNTHESIS_CHONDROITIN_S		22	-0.2335947	-0.7131141	0.864407	0.9938441	1	4868	tags=27%, list=22%, signal=35%
VALK_AML_CLUSTER_11	VALK_AML_CLUSTER_11		36	-0.2240754	-0.7125494	0.923077	0.9937348	1	2798	tags=17%, list=13%, signal=19%
BIOCARTA_EIF_PATHWAY	BIOCARTA_EIF_PATHWAY		16	-0.2762094	-0.7110013	0.803571	0.9945187	1	4463	tags=25%, list=20%, signal=31%
REACTOME_TRANSCRIPTION_COUPLED_NER	REACTOME_TRANSCRIPTION_COUPLED_NER		44	-0.2069293	-0.7102416	0.883333	0.99468017	1	2870	tags=16%, list=13%, signal=18%
PICCALUGA_ANGIOIMMUNOBLASTIC_LYMPHOMA_DN	PICCALUGA_ANGIOIMMUNOBLASTIC_LYMPHOMA_DN		123	-0.1692714	-0.7102185	1	0.9941632	1	4561	tags=20%, list=21%, signal=26%
LIU_SOX4_TARGETS_UP	LIU_SOX4_TARGETS_UP		131	-0.1705403	-0.7066356	0.982759	0.9966415	1	1765	tags=9%, list=8%, signal=10%
KEGG_ALPHA_LINOLENIC_ACID_METABOLISM	KEGG_ALPHA_LINOLENIC_ACID_METABOLISM		18	-0.2424963	-0.7064339	0.896552	0.99623615	1	3275	tags=17%, list=15%, signal=20%
MATTIOLI_MULTIPLE_MYELOMA_WITH_14Q32_TRANSLOCA	MATTIOLI_MULTIPLE_MYELOMA_WITH_14Q32_TRANSLOCATIONS		35	-0.2197582	-0.705116	0.968254	0.996749	1	7168	tags=49%, list=33%, signal=72%
WATANABE_RECTAL_CANCER_RADIOTHERAPY_RESPONSIV	WATANABE_RECTAL_CANCER_RADIOTHERAPY_RESPONSIVE_UP		98	-0.1687053	-0.704649	0.979167	0.99655366	1	5661	tags=27%, list=26%, signal=36%
BACOLOD_RESISTANCE_TO_ALKYLATING_AGENTS_UP	BACOLOD_RESISTANCE_TO_ALKYLATING_AGENTS_UP		17	-0.2465386	-0.7022361	0.803571	0.9978923	1	925	tags=12%, list=4%, signal=12%
BROWNE_HCMV_INFECTION_16HR_UP	BROWNE_HCMV_INFECTION_16HR_UP		211	-0.1642717	-0.702177	1	0.9973905	1	4418	tags=19%, list=20%, signal=24%
KEGG_PANTOTHENATE_AND_COA_BIOSYNTHESIS	KEGG_PANTOTHENATE_AND_COA_BIOSYNTHESIS		16	-0.2587651	-0.7010214	0.901961	0.9979566	1	4932	tags=38%, list=22%, signal=48%
REACTOME_SIGNALING_BY_BMP	REACTOME_SIGNALING_BY_BMP		23	-0.2299267	-0.7002859	0.866667	0.99798465	1	3557	tags=22%, list=16%, signal=26%
KEGG_NUCLEOTIDE_EXCISION_REPAIR	KEGG_NUCLEOTIDE_EXCISION_REPAIR		43	-0.2001281	-0.7000611	0.923077	0.9976695	1	3492	tags=16%, list=16%, signal=19%
REACTOME_GTP_HYDROLYSIS_AND_JOINING_OF_THE_60S	REACTOME_GTP_HYDROLYSIS_AND_JOINING_OF_THE_60S_RIB		103	-0.1694523	-0.6991591	0.982759	0.99780375	1	1849	tags=7%, list=8%, signal=7%
RIZ_ERYTHROID_DIFFERENTIATION_CCNE1	RIZ_ERYTHROID_DIFFERENTIATION_CCNE1		40	-0.2102044	-0.6989982	0.911111	0.99739295	1	3069	tags=15%, list=14%, signal=17%
JAERVINEN_AMPLIFIED_IN_LARYNGEAL_CANCER	JAERVINEN_AMPLIFIED_IN_LARYNGEAL_CANCER		39	-0.2194297	-0.6964254	0.964286	0.99889314	1	7662	tags=46%, list=35%, signal=71%
MULLIGHAN_NPM1_MUTATED_SIGNATURE_2_UP	MULLIGHAN_NPM1_MUTATED_SIGNATURE_2_UP		132	-0.1634941	-0.6955172	0.981818	0.99904186	1	6588	tags=30%, list=30%, signal=43%
GAZDA_DIAMOND_BLACKFAN_ANEMIA_PROGENITOR_UP	GAZDA_DIAMOND_BLACKFAN_ANEMIA_PROGENITOR_UP		37	-0.2095467	-0.6955095	0.90566	0.9985113	1	2312	tags=11%, list=11%, signal=12%
ELLWOOD_MYC_TARGETS_DN	ELLWOOD_MYC_TARGETS_DN		36	-0.2022225	-0.6949247	0.912281	0.998434	1	5750	tags=33%, list=26%, signal=45%
YANAGIHARA_ESX1_TARGETS	YANAGIHARA_ESX1_TARGETS		23	-0.2369798	-0.6938686	0.912281	0.9987243	1	2239	tags=13%, list=10%, signal=15%
BIOCARTA_NGF_PATHWAY	BIOCARTA_NGF_PATHWAY		18	-0.2519137	-0.6918483	0.843137	0.9999393	1	4961	tags=28%, list=23%, signal=36%
CHIBA_RESPONSE_TO_TSA_DN	CHIBA_RESPONSE_TO_TSA_DN		18	-0.2484065	-0.6910138	0.84	1	1	3688	tags=17%, list=17%, signal=20%
KEGG_BUTANOATE_METABOLISM	KEGG_BUTANOATE_METABOLISM		34	-0.2070285	-0.6907455	0.918367	0.99976534	1	3483	tags=15%, list=16%, signal=17%
RODRIGUES_THYROID_CARCINOMA_ANAPLASTIC_DN	RODRIGUES_THYROID_CARCINOMA_ANAPLASTIC_DN		474	-0.1430251	-0.6899573	1	0.99977696	1	4989	tags=20%, list=23%, signal=26%
MCCLUNG_CREB1_TARGETS_DN	MCCLUNG_CREB1_TARGETS_DN		52	-0.1846961	-0.6885958	0.966102	1	1	4623	tags=21%, list=21%, signal=27%
REACTOME_ELONGATION_AND_PROCESSING_OF_CAPPED	REACTOME_ELONGATION_AND_PROCESSING_OF_CAPPED_TRAN		131	-0.1672786	-0.6876419	0.983333	1	1	3296	tags=14%, list=15%, signal=16%
BIOCARTA_HDAC_PATHWAY	BIOCARTA_HDAC_PATHWAY		28	-0.2200952	-0.6870301	0.929825	1	1	7845	tags=46%, list=36%, signal=72%
KEGG_INTESTINAL_IMMUNE_NETWORK_FOR_IGA_PRODU	KEGG_INTESTINAL_IMMUNE_NETWORK_FOR_IGA_PRODUCTION		39	-0.2140135	-0.6846579	0.884615	1	1	2928	tags=13%, list=13%, signal=15%
FARMER_BREAST_CANCER_CLUSTER_4	FARMER_BREAST_CANCER_CLUSTER_4		16	-0.2445661	-0.6844204	0.92	1	1	2740	tags=19%, list=12%, signal=21%
ZHONG_RESPONSE_TO_AZACITIDINE_AND_TSA_DN	ZHONG_RESPONSE_TO_AZACITIDINE_AND_TSA_DN		65	-0.1854087	-0.683581	0.983607	1	1	5961	tags=28%, list=27%, signal=38%
CHEOK_RESPONSE_TO_MERCAPTOPURINE_AND_LD_MTX_	CHEOK_RESPONSE_TO_MERCAPTOPURINE_AND_LD_MTX_DN		15	-0.2362903	-0.6826648	0.777778	1	1	4655	tags=27%, list=21%, signal=34%
BIOCARTA_PGC1A_PATHWAY	BIOCARTA_PGC1A_PATHWAY		22	-0.2339819	-0.6822136	0.965517	1	1	1836	tags=9%, list=8%, signal=10%
HSC_MATURE_FETAL	HSC_MATURE_FETAL		17	-0.2370726	-0.6810607	0.947368	1	1	4372	tags=24%, list=20%, signal=29%
NELSON_RESPONSE_TO_ANDROGEN_DN	NELSON_RESPONSE_TO_ANDROGEN_DN		17	-0.2436576	-0.6787323	0.833333	1	1	3796	tags=18%, list=17%, signal=21%
LEE_DOUBLE_POLAR_THYMOCYTE	LEE_DOUBLE_POLAR_THYMOCYTE		17	-0.2416358	-0.675906	0.944444	1	1	4502	tags=29%, list=21%, signal=37%
SAKAI_TUMOR_INFILTRATING_MONOCYTES_DN	SAKAI_TUMOR_INFILTRATING_MONOCYTES_DN		78	-0.1767476	-0.674608	1	1	1	2281	tags=9%, list=10%, signal=10%
GARGALOVIC_RESPONSE_TO_OXIDIZED_PHOSPHOLIPIDS	GARGALOVIC_RESPONSE_TO_OXIDIZED_PHOSPHOLIPIDS_GREE		21	-0.2474819	-0.6743506	0.919355	1	1	6822	tags=43%, list=31%, signal=62%
KEGG_GLIOMA	KEGG_GLIOMA		63	-0.1862621	-0.6736229	0.978723	1	1	5162	tags=27%, list=24%, signal=35%
REACTOME_ASSOCIATION_OF_TRIC_CCT_WITH_TARGET_F	REACTOME_ASSOCIATION_OF_TRIC_CCT_WITH_TARGET_PROTE		29	-0.2112862	-0.6729559	0.96	1	1	7116	tags=38%, list=32%, signal=56%
KEGG_SELENOAMINO_ACID_METABOLISM	KEGG_SELENOAMINO_ACID_METABOLISM		26	-0.2250514	-0.6728153	0.90566	1	1	5778	tags=35%, list=26%, signal=47%
HOSHIDA_LIVER_CANCER_SUBCLASS_S2	HOSHIDA_LIVER_CANCER_SUBCLASS_S2		114	-0.1633198	-0.6683977	1	1	1	5713	tags=23%, list=26%, signal=31%
BIOCARTA_EGF_PATHWAY	BIOCARTA_EGF_PATHWAY		31	-0.2159983	-0.6641938	0.928571	1	1	2947	tags=16%, list=13%, signal=19%
KANG_FLUOROURACIL_RESISTANCE_UP	KANG_FLUOROURACIL_RESISTANCE_UP		20	-0.2329649	-0.6629865	0.953488	1	1	155	tags=5%, list=1%, signal=5%
BOYLAN_MULTIPLE_MYELOMA_C_UP	BOYLAN_MULTIPLE_MYELOMA_C_UP		31	-0.200471	-0.6627119	0.936508	1	1	4548	tags=26%, list=21%, signal=33%
GRADE_COLON_AND_RECTAL_CANCER_DN	GRADE_COLON_AND_RECTAL_CANCER_DN		73	-0.1625971	-0.6625201	0.96875	1	1	1763	tags=8%, list=8%, signal=9%
LU_AGING_BRAIN_DN	LU_AGING_BRAIN_DN		247	-0.1483937	-0.6623836	1	1	1	6380	tags=26%, list=29%, signal=37%
BIOCARTA_TPO_PATHWAY	BIOCARTA_TPO_PATHWAY		24	-0.2047723	-0.6618052	0.9375	1	1	4961	tags=25%, list=23%, signal=32%
NIKOLSKY_MUTATED_AND_AMPLIFIED_IN_BREAST_CANCE	NIKOLSKY_MUTATED_AND_AMPLIFIED_IN_BREAST_CANCER		88	-0.1645907	-0.6617164	0.981482	1	1	7	tags=1%, list=0%, signal=1%
BECKER_TAMOXIFEN_RESISTANCE_UP	BECKER_TAMOXIFEN_RESISTANCE_UP		35	-0.1982945	-0.6583425	0.934426	1	1	7344	tags=46%, list=33%, signal=69%
REACTOME_TRANSCRIPTION	REACTOME_TRANSCRIPTION		171	-0.1511946	-0.6569201	1	1	1	5697	tags=23%, list=26%, signal=31%
DOUGLAS_BMI1_TARGETS_DN	DOUGLAS_BMI1_TARGETS_DN		267	-0.1437002	-0.6568029	1	1	1	6803	tags=31%, list=31%, signal=44%
RADAEVA_RESPONSE_TO_IFNA1_UP	RADAEVA_RESPONSE_TO_IFNA1_UP		30	-0.2111385	-0.6565921	0.95	1	1	6059	tags=33%, list=28%, signal=46%

SESTO_RESPONSE_TO_UV_C0	SESTO_RESPONSE_TO_UV_C0		105	-0.16354	-0.6555281	1	1	1	5135	tags=19%, list=23%, signal=25%
SPIRA_SMOKERS_LUNG_CANCER_DN	SPIRA_SMOKERS_LUNG_CANCER_DN		17	-0.2502589	-0.6539282	0.90566	1	1	5591	tags=29%, list=25%, signal=39%
LU_TUMOR_ANGIOGENESIS_UP	LU_TUMOR_ANGIOGENESIS_UP		25	-0.2288108	-0.6529447	0.961538	1	1	6733	tags=40%, list=31%, signal=58%
CHNG_MULTIPLE_MYELOMA_HYPERPLOID_UP	CHNG_MULTIPLE_MYELOMA_HYPERPLOID_UP		50	-0.1847163	-0.6519502	0.953846	1	1	3316	tags=12%, list=15%, signal=14%
REACTOME_MTOR_SIGNALLING	REACTOME_MTOR_SIGNALLING		27	-0.2109238	-0.6515106	0.960784	1	1	973	tags=7%, list=4%, signal=8%
CORRE_MULTIPLE_MYELOMA_DN	CORRE_MULTIPLE_MYELOMA_DN		57	-0.1852185	-0.650878	0.982143	1	1	4555	tags=26%, list=21%, signal=33%
REACTOME_THE_ROLE_OF_NEF_IN_HIV1_REPLICATION_AI	REACTOME_THE_ROLE_OF_NEF_IN_HIV1_REPLICATION_AND_DI		28	-0.2143036	-0.6502983	0.951613	1	1	6884	tags=36%, list=31%, signal=52%
ROVERSI_GLIOMA_COPY_NUMBER_DN	ROVERSI_GLIOMA_COPY_NUMBER_DN		46	-0.1830091	-0.6474845	0.964286	1	1	3882	tags=20%, list=18%, signal=24%
REACTOME_PROCESSING_OF_CAPPED_INTRON_CONTAINII	REACTOME_PROCESSING_OF_CAPPED_INTRON_CONTAINING_PI		135	-0.1513063	-0.6460269	1	1	1	3296	tags=13%, list=15%, signal=15%
ENK_UV_RESPONSE_KERATINOCYTE_DN	ENK_UV_RESPONSE_KERATINOCYTE_DN		470	-0.1296704	-0.6457089	1	1	1	5912	tags=24%, list=27%, signal=32%
HAHTOLA_MYCOSIS_FUNGOIDES_SKIN_UP	HAHTOLA_MYCOSIS_FUNGOIDES_SKIN_UP		174	-0.1499403	-0.6447422	1	1	1	5795	tags=24%, list=26%, signal=33%
BIOCARTA_G1_PATHWAY	BIOCARTA_G1_PATHWAY		27	-0.209123	-0.643723	0.946429	1	1	5538	tags=30%, list=25%, signal=40%
CHOI_ATL_STAGE_PREDICTOR	CHOI_ATL_STAGE_PREDICTOR		34	-0.2072933	-0.642884	0.924528	1	1	8655	tags=50%, list=39%, signal=82%
TAKAO_RESPONSE_TO_UVB_RADIATION_UP	TAKAO_RESPONSE_TO_UVB_RADIATION_UP		73	-0.1799721	-0.642095	1	1	1	5135	tags=22%, list=23%, signal=29%
NIKOLSKY_OVERCONNECTED_IN_BREAST_CANCER	NIKOLSKY_OVERCONNECTED_IN_BREAST_CANCER		17	-0.2394675	-0.6415758	0.962264	1	1	6284	tags=41%, list=29%, signal=58%
VALK_AML_CLUSTER_8	VALK_AML_CLUSTER_8		22	-0.2127082	-0.6413476	0.896552	1	1	3682	tags=18%, list=17%, signal=22%
WAKASUGI_HAVE_ZNF143_BINDING_SITES	WAKASUGI_HAVE_ZNF143_BINDING_SITES		55	-0.1700684	-0.6387678	1	1	1	6400	tags=27%, list=29%, signal=38%
KORKOLA_EMBRYONAL_CARCINOMA_UP	KORKOLA_EMBRYONAL_CARCINOMA_UP		41	-0.1902661	-0.6384701	0.980769	1	1	4700	tags=27%, list=21%, signal=34%
CERVERA_SDHB_TARGETS_1_DN	CERVERA_SDHB_TARGETS_1_DN		32	-0.188338	-0.6346858	0.87037	1	1	5784	tags=31%, list=26%, signal=42%
REACTOME_MYOGENESSIS	REACTOME_MYOGENESSIS		29	-0.1988703	-0.6340001	0.95082	1	1	2669	tags=14%, list=12%, signal=16%
KEGG_GLYCOSAMINOGLYCAN_BIOSYNTHESIS_KERATAN_S	KEGG_GLYCOSAMINOGLYCAN_BIOSYNTHESIS_KERATAN_SULFAT		15	-0.2333229	-0.6339354	0.914894	1	1	6895	tags=40%, list=31%, signal=58%
BIOCARTA_PITX2_PATHWAY	BIOCARTA_PITX2_PATHWAY		15	-0.2418441	-0.6333784	0.928571	1	1	5218	tags=33%, list=24%, signal=44%
REACTOME_NUCLEOTIDE_EXCISION_REPAIR	REACTOME_NUCLEOTIDE_EXCISION_REPAIR		49	-0.1823847	-0.6317558	1	1	1	2870	tags=14%, list=13%, signal=16%
KEGG_BASE_EXCISION_REPAIR	KEGG_BASE_EXCISION_REPAIR		34	-0.1987848	-0.6305178	0.983607	1	1	8168	tags=47%, list=37%, signal=75%
BIOCARTA_RAC1_PATHWAY	BIOCARTA_RAC1_PATHWAY		23	-0.1980471	-0.6304598	0.961538	1	1	3410	tags=13%, list=16%, signal=15%
YANG_BREAST_CANCER_ESR1_DN	YANG_BREAST_CANCER_ESR1_DN		19	-0.2179016	-0.6295722	0.96	1	1	224	tags=5%, list=1%, signal=5%
LINDGREN_BLADDER_CANCER_CLUSTER_1_UP	LINDGREN_BLADDER_CANCER_CLUSTER_1_UP		114	-0.1520441	-0.6290717	1	1	1	2108	tags=8%, list=10%, signal=9%
BIOCARTA_GPCR_PATHWAY	BIOCARTA_GPCR_PATHWAY		33	-0.2007253	-0.6286202	0.962963	1	1	5176	tags=21%, list=24%, signal=28%
WONG_IFNA2_RESISTANCE_DN	WONG_IFNA2_RESISTANCE_DN		16	-0.2188224	-0.6281274	1	1	1	7513	tags=44%, list=34%, signal=67%
WEBER_METHYLATED_HCP_IN_SPERM_DN	WEBER_METHYLATED_HCP_IN_SPERM_DN		22	-0.2188423	-0.6272323	0.962963	1	1	3742	tags=18%, list=17%, signal=22%
BIOCARTA_TCR_PATHWAY	BIOCARTA_TCR_PATHWAY		44	-0.172645	-0.6255482	0.982456	1	1	7568	tags=41%, list=35%, signal=62%
GAZDA_DIAMOND_BLACKFAN_ANEMIA_ERYTHROID_UP	GAZDA_DIAMOND_BLACKFAN_ANEMIA_ERYTHROID_UP		24	-0.2091091	-0.6212004	0.928571	1	1	5694	tags=38%, list=26%, signal=51%
KEGG_SNARE_INTERACTIONS_IN_VESICULAR_TRANSPORT	KEGG_SNARE_INTERACTIONS_IN_VESICULAR_TRANSPORT		36	-0.1854916	-0.6208312	0.943396	1	1	6669	tags=28%, list=30%, signal=40%
FARMER_BREAST_CANCER_CLUSTER_5	FARMER_BREAST_CANCER_CLUSTER_5		19	-0.2238545	-0.6179658	0.941177	1	1	7002	tags=47%, list=32%, signal=70%
GOLDRATH_HOMEOSTATIC_PROLIFERATION	GOLDRATH_HOMEOSTATIC_PROLIFERATION		161	-0.1398794	-0.6177162	1	1	1	5238	tags=21%, list=24%, signal=28%
GAUSSMANN_MLL_AF4_FUSION_TARGETS_G_DN	GAUSSMANN_MLL_AF4_FUSION_TARGETS_G_DN		27	-0.2064735	-0.616181	0.943396	1	1	5742	tags=26%, list=26%, signal=35%
BIOCARTA_CCR5_PATHWAY	BIOCARTA_CCR5_PATHWAY		17	-0.2134193	-0.6094259	0.951613	1	1	4980	tags=29%, list=23%, signal=38%
BIOCARTA_MET_PATHWAY	BIOCARTA_MET_PATHWAY		37	-0.1762853	-0.6055316	0.952381	1	1	2940	tags=14%, list=13%, signal=16%
BIOCARTA_PAR1_PATHWAY	BIOCARTA_PAR1_PATHWAY		37	-0.1697113	-0.6029627	1	1	1	7517	tags=43%, list=34%, signal=66%
VANHARANTA_UTERINE_FIBROID_WITH_7Q_DELETION_UP	VANHARANTA_UTERINE_FIBROID_WITH_7Q_DELETION_UP		67	-0.1605404	-0.6023677	1	1	1	3580	tags=16%, list=16%, signal=20%
MCCABE_HOXC6_TARGETS_CANCER_UP	MCCABE_HOXC6_TARGETS_CANCER_UP		29	-0.1947126	-0.6020266	0.954545	1	1	5235	tags=34%, list=24%, signal=45%
REACTOME_FORMATION_OF_A_POOL_OF_FREE_40S_SUBU	REACTOME_FORMATION_OF_A_POOL_OF_FREE_40S_SUBUNITS		92	-0.151139	-0.6010867	1	1	1	3761	tags=11%, list=17%, signal=13%
MMS_MOUSE_LYMPH_HIGH_4HRS_UP	MMS_MOUSE_LYMPH_HIGH_4HRS_UP		34	-0.1836804	-0.600821	0.96	1	1	5972	tags=29%, list=27%, signal=40%
OSMAN_BLADDER_CANCER_UP	OSMAN_BLADDER_CANCER_UP		370	-0.1263529	-0.6002301	1	1	1	5377	tags=20%, list=25%, signal=26%
REACTOME_GLUCOSE_TRANSPORT	REACTOME_GLUCOSE_TRANSPORT		38	-0.1822703	-0.6000432	0.933333	1	1	2186	tags=8%, list=10%, signal=9%
KEGG_DORSO_VENTRAL_AXIS_FORMATION	KEGG_DORSO_VENTRAL_AXIS_FORMATION		22	-0.1985561	-0.5980643	0.964286	1	1	5837	tags=36%, list=27%, signal=50%
HEIDENBLAD_AMPLIFIED_IN_PANCREATIC_CANCER	HEIDENBLAD_AMPLIFIED_IN_PANCREATIC_CANCER		30	-0.1954498	-0.5975386	0.966667	1	1	5140	tags=30%, list=23%, signal=39%
BIOCARTA_CTCF_PATHWAY	BIOCARTA_CTCF_PATHWAY		23	-0.1947818	-0.5897359	0.982456	1	1	4938	tags=22%, list=23%, signal=28%
MARTINEZ_RESPONSE_TO_TRABECTEDIN_DN	MARTINEZ_RESPONSE_TO_TRABECTEDIN_DN		216	-0.1333185	-0.5892134	1	1	1	4803	tags=17%, list=22%, signal=22%
PELLICCIOTTA_HDAC_IN_ANTIGEN_PRESENTATION_UP	PELLICCIOTTA_HDAC_IN_ANTIGEN_PRESENTATION_UP		61	-0.164408	-0.5875238	0.981818	1	1	6347	tags=25%, list=29%, signal=35%
YAO_HOXA10_TARGETS_VIA_PROGESTERONE_DN	YAO_HOXA10_TARGETS_VIA_PROGESTERONE_DN		17	-0.2157324	-0.5822589	0.964286	1	1	6976	tags=41%, list=32%, signal=60%
NIKOLSKY_BREAST_CANCER_7P22_AMPLICON	NIKOLSKY_BREAST_CANCER_7P22_AMPLICON		35	-0.1636798	-0.5819172	1	1	1	2072	tags=11%, list=9%, signal=13%
REACTOME_TRANSPORT_OF_MATURE_MRNA_DERIVED_FR	REACTOME_TRANSPORT_OF_MATURE_MRNA_DERIVED_FROM_A		51	-0.1497838	-0.5798239	1	1	1	2453	tags=10%, list=11%, signal=11%
ROSS_AML_OF_FAB_M7_TYPE	ROSS_AML_OF_FAB_M7_TYPE		67	-0.1568908	-0.5790831	1	1	1	6500	tags=28%, list=30%, signal=40%
REACTOME_FORMATION_AND_MATURATION_OF_MRNA_TR	REACTOME_FORMATION_AND_MATURATION_OF_MRNA_TRANSC		149	-0.1362978	-0.5785459	1	1	1	3296	tags=12%, list=15%, signal=14%
FAELT_B_CLL_WITH_VH_REARRANGEMENTS_DN	FAELT_B_CLL_WITH_VH_REARRANGEMENTS_DN		45	-0.1638136	-0.5755706	0.981132	1	1	6407	tags=33%, list=29%, signal=47%
VALK_AML_CLUSTER_16	VALK_AML_CLUSTER_16		26	-0.1869305	-0.5739119	1	1	1	6951	tags=35%, list=32%, signal=51%
ST_MYOCYTE_AD_PATHWAY	ST_MYOCYTE_AD_PATHWAY		23	-0.2016604	-0.5709112	0.967742	1	1	3956	tags=26%, list=18%, signal=32%
RIZ_ERYTHROID_DIFFERENTIATION_APOBEC2	RIZ_ERYTHROID_DIFFERENTIATION_APOBEC2		26	-0.1785346	-0.5708209	0.963636	1	1	8852	tags=50%, list=40%, signal=84%
BARIS_THYROID_CANCER_UP	BARIS_THYROID_CANCER_UP		20	-0.1974234	-0.5681928	0.957447	1	1	5171	tags=30%, list=24%, signal=39%
REACTOME_SYNTHESIS_OF_GPI_ANCHORED_PROTEINS	REACTOME_SYNTHESIS_OF_GPI_ANCHORED_PROTEINS		26	-0.1775936	-0.5625261	0.981818	1	1	3996	tags=19%, list=18%, signal=23%
GAZDA_DIAMOND_BLACKFAN_ANEMIA_PROGENITOR_DN	GAZDA_DIAMOND_BLACKFAN_ANEMIA_PROGENITOR_DN		60	-0.1572204	-0.5610192	1	1	1	4056	tags=15%, list=18%, signal=18%
SESTO_RESPONSE_TO_UV_C5	SESTO_RESPONSE_TO_UV_C5		46	-0.1562715	-0.5555167	0.984615	1	1	8082	tags=46%, list=37%, signal=72%
REACTOME_ADP_SIGNALLING_THROUGH_P2Y_PURINOCEP	REACTOME_ADP_SIGNALLING_THROUGH_P2Y_PURINOCEPTOR_		21	-0.2008424	-0.5540335	1	1	1	3226	tags=19%, list=15%, signal=22%

YANG_BREAST_CANCER_ESR1_LASER_DN	YANG_BREAST_CANCER_ESR1_LASER_DN		36	-0.1792623	-0.5527563	1	1	1	4622	tags=19%, list=21%, signal=25%
KANNAN_TP53_TARGETS_DN	KANNAN_TP53_TARGETS_DN		16	-0.1938524	-0.551561	1	1	1	7166	tags=44%, list=33%, signal=65%
SEITZ_NEOPLASTIC_TRANSFORMATION_BY_8P_DELETION	SEITZ_NEOPLASTIC_TRANSFORMATION_BY_8P_DELETION_DN		30	-0.165138	-0.5499606	0.980769	1	1	4740	tags=23%, list=22%, signal=30%
KEGG_BASAL_TRANSCRIPTION_FACTORS	KEGG_BASAL_TRANSCRIPTION_FACTORS		34	-0.1728705	-0.5482864	0.976744	1	1	18145	tags=100%, list=83%, signal=579%
AMIT_EGF_RESPONSE_60_HELA	AMIT_EGF_RESPONSE_60_HELA		43	-0.1624627	-0.5464001	0.969231	1	1	450	tags=5%, list=2%, signal=5%
KEGG_GLYCOSAMINOGLYCAN_DEGRADATION	KEGG_GLYCOSAMINOGLYCAN_DEGRADATION		21	-0.1886707	-0.5445534	1	1	1	2865	tags=14%, list=13%, signal=16%
FAELT_B_CLL_WITH_VH3_21_DN	FAELT_B_CLL_WITH_VH3_21_DN		46	-0.1452557	-0.5436329	1	1	1	7717	tags=41%, list=35%, signal=64%
REACTOME_E2F_MEDIATED_REGULATION_OF_DNA_REPLIC	REACTOME_E2F_MEDIATED_REGULATION_OF_DNA_REPLICATIOI		31	-0.1781604	-0.5428092	1	1	1	6421	tags=32%, list=29%, signal=46%
MAYBURD_RESPONSE_TO_L663536_DN	MAYBURD_RESPONSE_TO_L663536_DN		50	-0.1559573	-0.5401844	1	1	1	5304	tags=20%, list=24%, signal=26%
LUI_TARGETS_OF_PAX8_PPARG_FUSION	LUI_TARGETS_OF_PAX8_PPARG_FUSION		32	-0.1662969	-0.5376997	1	1	1	3159	tags=13%, list=14%, signal=15%
REACTOME_TRANSLATION_INITIATION_COMPLEX_FORMAT	REACTOME_TRANSLATION_INITIATION_COMPLEX_FORMATION		53	-0.1507048	-0.5376694	0.981818	1	1	1584	tags=8%, list=7%, signal=8%
VALK_AML_CLUSTER_6	VALK_AML_CLUSTER_6		32	-0.1743376	-0.5357568	0.982143	1	1	2175	tags=9%, list=10%, signal=10%
BIOCARTA_AT1R_PATHWAY	BIOCARTA_AT1R_PATHWAY		32	-0.1622713	-0.5354224	1	1	1	4980	tags=25%, list=23%, signal=32%
BIOCARTA_CXCR4_PATHWAY	BIOCARTA_CXCR4_PATHWAY		24	-0.1811062	-0.5331937	0.964286	1	1	6751	tags=38%, list=31%, signal=54%
ZHAN_MULTIPLE_MYELOMA_CD2_DN	ZHAN_MULTIPLE_MYELOMA_CD2_DN		44	-0.1490619	-0.5270324	0.981132	1	1	4077	tags=18%, list=19%, signal=22%
GARY_CD5_TARGETS_DN	GARY_CD5_TARGETS_DN		412	-0.1080288	-0.5232692	1	1	1	6779	tags=25%, list=31%, signal=36%
HEIDENBLAD_AMPLICON_8Q24_DN	HEIDENBLAD_AMPLICON_8Q24_DN		30	-0.1585079	-0.5221495	0.967742	1	1	5026	tags=20%, list=23%, signal=26%
BOYAULT_LIVER_CANCER_SUBCLASS_G6_UP	BOYAULT_LIVER_CANCER_SUBCLASS_G6_UP		62	-0.1377316	-0.5220298	1	1	1	4065	tags=18%, list=19%, signal=22%
REACTOME_P75_NTR_RECEPTOR_MEDIATED_SIGNALLING	REACTOME_P75_NTR_RECEPTOR_MEDIATED_SIGNALLING		82	-0.1351408	-0.5088612	1	1	1	6682	tags=30%, list=30%, signal=44%
REACTOME_MRNA_SPLICING_MINOR_PATHWAY	REACTOME_MRNA_SPLICING_MINOR_PATHWAY		39	-0.1430541	-0.5008285	1	1	1	7487	tags=31%, list=34%, signal=47%
REACTOME_G2_M_TRANSITION	REACTOME_G2_M_TRANSITION		81	-0.1288089	-0.5007597	1	1	1	2795	tags=10%, list=13%, signal=11%
REACTOME_CHAPERONIN_MEDIATED_PROTEIN_FOLDING	REACTOME_CHAPERONIN_MEDIATED_PROTEIN_FOLDING		47	-0.1412659	-0.4976558	1	1	1	7116	tags=34%, list=32%, signal=50%
HOFFMANN_IMMATURE_TO_MATURE_B_LYMPHOCYTE_UP	HOFFMANN_IMMATURE_TO_MATURE_B_LYMPHOCYTE_UP		24	-0.1642365	-0.4953184	1	1	1	6313	tags=29%, list=29%, signal=41%
GENTILE_UV_RESPONSE_CLUSTER_D7	GENTILE_UV_RESPONSE_CLUSTER_D7		31	-0.1455497	-0.4849764	1	1	1	7244	tags=39%, list=33%, signal=58%
BIOCARTA_CTLA4_PATHWAY	BIOCARTA_CTLA4_PATHWAY		19	-0.1717657	-0.483637	0.979592	1	1	3182	tags=16%, list=15%, signal=18%
REACTOME_NRAGE_SIGNALS_DEATH_THROUGH_JNK	REACTOME_NRAGE_SIGNALS_DEATH_THROUGH_JNK		47	-0.1250235	-0.4562973	1	1	1	6663	tags=30%, list=30%, signal=43%
REACTOME_HOST_INTERACTIONS_OF_HIV_FACTORS	REACTOME_HOST_INTERACTIONS_OF_HIV_FACTORS		116	-0.1106674	-0.4529107	1	1	1	8758	tags=39%, list=40%, signal=64%
TOMLINS_PROSTATE_CANCER_UP	TOMLINS_PROSTATE_CANCER_UP		34	-0.1345047	-0.4434083	1	1	1	5808	tags=24%, list=26%, signal=32%
STEIN_ESTROGEN_RESPONSE_NOT_VIA_ESRRA	STEIN_ESTROGEN_RESPONSE_NOT_VIA_ESRRA		18	-0.1424709	-0.4058165	1	1	1	5211	tags=22%, list=24%, signal=29%
REACTOME_DUAL_INCISION_REACTION_IN_GG_NER	REACTOME_DUAL_INCISION_REACTION_IN_GG_NER		20	-0.136428	-0.3892816	1	0.99984586	1	18941	tags=100%, list=86%, signal=733%
