# Supplementary material for: Reduced Retinal Microvascular Density, Improved Forepaw Reach, Comparative Microarray and Gene Set Enrichment Analysis with c-jun Targeting DNA Enzyme
Source: PLoS One. 2012 Jul 17;7(7):e39160. doi: 10.1371/journal.pone.0039160 (PMC3398922; doi:10.1371/journal.pone.0039160)
Supplement: Table S2 — Summarized curated gene set terms with a Q-value (<0.05) downregulated by Dz13. Since names given to gene sets can be uninformative summarizing themes in collections of gene sets can be difficult. Here we used unique words in the names of each gene set and summarized the frequency of word occurrence. A complete list of all curated downregulated gene sets identified in GSEA is provided in Table S1. (PDF) [file pone.0039160.s003.pdf]

Supplementary Table 2. **Summarized curated gene set terms with a Q-value (<0.05) downregulated by Dz13.** Since names given to gene sets can be uninformative summarizing themes in collections of gene sets can be difficult. Here we used unique words in the names of each gene set and summarized the frequency of word occurrence. A complete list of all curated downregulated gene sets identified in GSEA is provided in the Supplementary Table 1.

Term in Gene Set	Frequency
Cancer	46
Luminal or Basal	12
Tumour/s	7
Metastasis	5
Carcinoma	4
Myeloma	3
